# HiMMe: using genetic patterns as a proxy for genome assembly reliability assessment

**DOI:** 10.1186/s12864-017-3965-2

**Published:** 2017-09-05

**Authors:** Jordi Abante, Noushin Ghaffari, Charles D. Johnson, Aniruddha Datta

**Affiliations:** 1 Whitaker Biomedical Engineering Institute, Johns Hopkins University, 3400 N Charles St, Baltimore, MD USA; 2 Center for Bioinformatics and Genomic Systems Engineering (CBGSE), 101 Gateway Blvd., College Station, TX USA; 3 Dwight Look College of Engineering, Texas A&M University, 400 Bizzell St, College Station, TX USA; 4 AgriLife Genomics and Bioinformatics, Texas A&M AgriLife Research, 101 Gateway, Suite A, College Station, TX USA

**Keywords:** Genome assemblies, *de novo* assemblies, Sequence analysis, Hidden Markov models, Markov chains, Stochastic processes, Supervised learning

## Abstract

**Background:**

The information content of genomes plays a crucial role in the existence and proper development of living organisms. Thus, tremendous effort has been dedicated to developing DNA sequencing technologies that provide a better understanding of the underlying mechanisms of cellular processes. Advances in the development of sequencing technology have made it possible to sequence genomes in a relatively fast and inexpensive way. However, as with any measurement technology, there is noise involved and this needs to be addressed to reach conclusions based on the resulting data. In addition, there are multiple intermediate steps and degrees of freedom when constructing genome assemblies that lead to ambiguous and inconsistent results among assemblers.

**Methods:**

Here we introduce HiMMe, an HMM-based tool that relies on genetic patterns to score genome assemblies. Through a Markov chain, the model is able to detect characteristic genetic patterns, while, by introducing emission probabilities, the noise involved in the process is taken into account. Prior knowledge can be used by training the model to fit a given organism or sequencing technology.

**Results:**

Our results show that the method presented is able to recognize patterns even with relatively small k-mer size choices and limited computational resources.

**Conclusions:**

Our methodology provides an individual quality metric per contig in addition to an overall genome assembly score, with a time complexity well below that of an aligner. Ultimately, HiMMe provides meaningful statistical insights that can be leveraged by researchers to better select contigs and genome assemblies for downstream analysis.

**Electronic supplementary material:**

The online version of this article (doi:10.1186/s12864-017-3965-2) contains supplementary material, which is available to authorized users.

## Introduction

When constructing genome assemblies there are multiple intermediate steps that can significantly impact the results obtained. For instance, DNA sequencing inherently carries uncertainty in the signal that is known to depend on the position of the nucleotide in the read. Furthermore, all currently available assemblers use heuristics that rely on arbitrary thresholds that must be defined by the user. As a result, even when using the same input data, an assembler will tend to lead to different genome assemblies depending on its particular configuration. This will also be the case when comparing the output of different assemblers. Thus, it is very common for researchers to use the same assembler multiple times with different parameter choices and, at the same time, to generate assemblies with multiple assemblers. Therefore, it is of paramount importance to provide researchers with a rigorous methodology to compare results and make sure that the assembly (or subset of contigs) chosen for downstream analysis is as reliable as possible.

Here we introduce HiMMe [1], a tool that allows researchers to compare results of different assembly runs and provides them with meaningful statistical insight to filter, compare, and choose between them based on genetic patterns. To this end, we take advantage of a hidden Markov model (HMM). This probabilistic model is an appealing choice since it allows one to model noisy signals (i.e. the sequences or contigs observed after a given workflow). Given its flexibility, this model has been extensively used in computational biology. For instance, hhmSeq [2] is a model-based hierarchical Bayesian technique that was conceived to detect differentially expressed genes. This tool uses HMMs to account for potential co-expression of neighboring genes. Another precedent was established by CodingQuarry [3], a tool that implements HMMs to predict genes in fungal genomes. Finally, a prominent tool in computational biology is HMMER [4], which also relies on HMMs. More specifically, HMMER is based on profile HMMs, and it is currently used as an alternative to BLAST [5]. Its main purpose is searching sequence databases for sequence homologs and making multiple sequence alignments.

HiMMe relies on HMMs and aims to provide a new set of quality metrics for genome assembly assessment based on genetic content. Given some prior knowledge about the organism, this metrics quantify how similar the genetic patterns found in the input are to that of the training set of sequences. A very common choice for genome assembly assessment is BUSCO [6] and, although its goal is the same as HiMMe’s, the approach used by this method is very different. BUSCO bases its scores on a homologous gene database search performed using BLAST and HMMER. Thus, it depends on a homologous gene database and inherently implies multiple sequence alignment. According to HMMER’s authors, the current release (HMMER3), is essentially as fast as BLAST [7]. Although we have not been able to find theoretical time complexities for either of them, we estimate that they are similar to that of the well-known Smith-Waterman algorithm (SWA) [8]. Therefore, we can assume that the time complexities of HMMER and BLAST are close to *O*(*MN*), where *M* is the number of nucleotides in the query sequence and *N* is the number of nucleotides in the database sequence. It follows that, considering that both sequences have the same length, the complexity of these algorithms has a quadratic relationship with the length of the sequences being aligned. Another common choice for genome assembly assessment is QUAST [9], which generates a very complete report with various quality metrics (e.g. NX, genome coverage). In addition, this tool also reports the NAX metric, which has the same meaning as the NX metric but only considers aligned contigs. However, when no reference genome is available, the number of metrics available is fairly limited.

Although HiMMe also relies on HMMs, the approach followed to model and process the data is completely different than that used by BUSCO. Our method involves no alignment, making it potentially faster than BUSCO, which will have at least the same time complexity as HMMER and BLAST, as well as QUAST. The algorithm proposed here takes advantage of the well-known *forward algorithm* and, as a result, its time complexity is linearly related to the product of the length of the sequence and the square of the state space cardinality. Consequently, even when not many computational resources are available, this method can still be used.

Furthermore, our method does not rely on heuristics in contrast to BLAST and other multiple alignment tools. Additionally, the user can employ different types of data to train the model. In a classical setting, the user will most likely choose the reference genome if available. On the other hand, in a *de-novo* genome assembly setting, i.e. when no reference genome is available, this is a scenario that can be handled well by our method too. It is important to note that under these conditions the researcher would not be able to align the assembly back to the reference to assess its quality. However, HiMMe would allow the user to employ some other prior knowledge to train the model, such as a closely related species genome or a database made up of homologous genes.

Finally, our method provides not only a general metric for the entire assembly, but also an individual metric for each contig present in the assembly as opposed to BUSCO and other tools. In turn, the user can filter contigs based on a cut-off in order to improve reliability of the assembly for downstream analysis. Moreover, distributions can be drawn for each assembly and significant differences can be found between these through a suitable statistical test.

In conclusion, our methodology provides the user with three different ways to assess the quality of a genome assembly (even in a *de-novo* setting): (i) by looking at individual scores of each contig, (ii) by looking at score distributions of the entire assembly, and (iii) by looking at the HiMMe coefficient computed for the entire genome assembly.

## Background

This section formally defines Markov chains and hidden Markov models. It also provides the necessary background material needed for developing our method.

### Markov chain

In a Markov chain (MC) each state *X*_*i*_ is a random variable that takes values in a set *E*, i.e. the state space of the Markov chain. We denote a general sequence of states as:

1 $$  X=\{X_{1},X_{2},\ldots,X_{m}\}  $$

where *m* is the number of states in the sequence. We denote the set of all possible sequences of states of length *m* as *Ω*_*m*_. The stochastic process $X=\{X_{n};n\in \mathbb {N}\}$ is called a Markov chain provided that:

2 $$ P\left[X_{n+1}=j|X_{0},\ldots,X_{n}\right]=P\left[X_{n+1}=j|X_{n}\right]  $$

for all *j*∈*E* and $n \in \mathbb {N}$ [10]. It turns out that the probability of any given path can be computed as the product of the initial state probability *π*_0_(·) followed by the respective transition probabilities:

3 $$ P[X]=\pi_{0}(x_{1})\prod_{i=2}^{m}P\left[X_{i}=x_{i}|X_{i-1}=x_{i-1}\right]  $$

### Hidden Markov models

Hidden Markov models (HMMs) can describe the behavior of a system based on observable events. For instance, one could try to infer the position of the upstairs neighbor based on the sound of his or her steps. The observable events (i.e. the noise produced by the steps in this case) are called symbols. On the other hand, the underlying or invisible factor one is trying to understand (i.e. the position of the neighbor) is referred to as state. The probability distribution of the symbols depends on the underlying states while the latter form an MC. Thus, given that the present state is known, the future states are conditionally independent of the past.

#### Emission probabilities

Let us define the emission probabilities, which bridge the gap between the symbols and the underlying sequence of states. Each symbol *Y*_*i*_ is a random variable that takes on a set of possible observations *O* based on a probability distribution conditioned on the current underlying state. We denote a sequence of symbols as follows:

4 $$  Y=\{Y_{1},Y_{2},\ldots,Y_{m}\}  $$

where *m* is the number of observations in the sequence. We denote the set of all possible sequences of observations of length *m* as *Λ*_*m*_. Since the random variable *Y*_*i*_ takes values in *O* based on the current state only, we define the emission probabilities as follows:

5 $$ e(Y_{n}|X_{n}) = P\left[Y_{n}|X_{n}\right]   $$

#### Complete model

The HMM is completely specified by the probabilities *π*_0_(*x*_1_), *P*[*x*_*n*_|*x*_*n*−1_] and *e*(*y*_*n*_|*x*_*n*_). Here, we denote the set of these probabilities by *Θ* for convenience. For a realization *Y* and *X*, we have:

6 $$\begin{array}{*{20}l} P\left[Y,X;\Theta\right]&=P\left[Y|X;\Theta\right]P\left[X;\Theta\right] \end{array} $$

where

7 $$  P\left[Y|X;\Theta\right]=e(Y_{1}|X_{1})e(Y_{2}|X_{2}) \cdot\cdot\cdot e(Y_{m}|X_{m})  $$

and

8 $$  P\left[X;\Theta\right]=\pi_{0}(X_{1})P\left[X_{2}|X_{1}\right] \cdot\cdot\cdot P\left[X_{m}|X_{m-1}\right]  $$

Thus, when the underlying sequence of states is known, the probability of observing a given sequence of symbols can be computed readily. Note that by the Law of Total Probability (LTP):

9 $$  P\left[Y;\Theta\right]=\sum\limits_{x \in \Omega_{m}}^{}P\left[Y,X=x;\Theta\right]  $$

One could potentially go through every single possible hidden state sequence to find the distribution of the observation given the model. However, depending on how large the state space is and the number of elements in the chain, this might not even be feasible. Taking advantage of Bayes’ rule, Eq. (9) can be expanded in the following way:

10 $$  P\left[Y;\Theta\right]=\sum\limits_{x \in \Omega_{m}}^{}P\left[Y|X=x;\Theta\right]P\left[X=x;\Theta\right]  $$

and by using Eqs. (7) and (8), Eq. (10) can be written in the following way:

11 $$  \sum\limits_{x \in \Omega_{m}}^{}\pi_{0}(x_{1})e(Y_{1}|x_{1})\prod_{i=2}^{m}e(Y_{i}|x_{i})P\left[x_{i}|x_{i-1}\right]  $$

## Methods

This section presents our novel HMM-based methodology that uses the genetic content as a proxy for the reliability of an assembly.

### Modeling the state sequences

The probability of any hidden state sequence *X*={*X*_1_,*X*_2_,…,*X*_*m*_} can be computed as follows:

12 $$ P\left[X;\Theta\right]=\pi_{0}(X_{1})\prod_{i=2}^{m}P\left[X_{i}|X_{i-1}\right]  $$

Here, we consider that the hidden state space *E* will contain all the combinations that can be formed from the set of nucleotides *N*={*A,C,G,T*}. These combinations are sequences of *k* elements and are usually referred to as *k-mers*. Note however that in this case we consider non-overlapping k-mers given that we are in an HMM setting. It should also be noted that k-mers, in general, are derived from sequenced reads and can be overlapping. The cardinality of the hidden state space will have an exponential relationship with the k-mer size used to model the data:

13 $$  |E|=|N|^{k}=4^{k}  $$

As an example, let us consider the following sequence: *S*=*ACTAGACAG*. Furthermore, let us assume that the k-mer size chosen to break down sequence *S* is three. The probability of observing such a sequence of states would be:

14 $$ P\left[S;\Theta\right]=\pi_{0}(ACT)P\left[AGA|ACT\right]P\left[CAG|AGA\right]  $$

These probabilities can be found in the transition matrix which is learned by parsing the training set of sequences and computing the frequency of each transition.

### Modeling the symbol sequences

Each sequence present in the genome assembly being analyzed will be regarded as a sequence of symbols. The emission probabilities will be used then to evaluate how likely is to observe each element in the observation chain given the corresponding hidden state.

Let us assume that the set of possible observations and the hidden state space are the same, i.e. *O*=*E*. Consider k-mers *X*_*i*_={*X*_*i*1_,…,*X*_*ik*_} and *Y*_*i*_={*Y*_*i*1_,…,*Y*_*ik*_}. The emission probability of observing *Y*_*i*_ provided that *X*_*i*_ is the hidden state has been introduced in Eq. (5). The method introduced is based on the assumption that only when the underlying state *X*_*i*_ is given, the random variables *Y*_*ij*_ are independent. That is, the observed adjacent nucleotides are conditionally independent when the underlying state is given. As a result, the set of possible observations becomes the set of nucleotides, i.e. *O*=*N*, and its cardinality is significantly reduced when *k*≥2. This assumption allows one to express the emission probability of observing k-mer *Y*_*i*_ given the hidden state *X*_*i*_ as the product of the different emission probabilities at a single base resolution:

15 $$ e(Y_{i}|X_{i})=\prod_{j=1}^{k}e(Y_{ij}|X_{ij})  $$

For instance, if we observe the symbol *Y*_*i*_={*ACT*} and we know that the corresponding hidden state is *X*_*i*_={*AGT*}, the emission probability would be:

16 $$ P\left[Y_{i}=ACT|X_{i}=AGT\right]=e(ACT|AGT)  $$

Then, assuming conditional independence, this emission probability could be computed in the following way:

17 $$ e(ACT|AGT)=e(A|A)e(C|G)e(T|T)  $$

These probabilities are stored in the emission matrix. We suggest using a SNP database might be a good way to estimate such probabilities. However, the user is completely free to decide what values are given to the entries of this matrix. Note that in the limit, where we have complete confidence in our data, i.e. when the emission matrix is diagonal (e(y|x) = 1 when y = x and zero otherwise), then the HMM becomes an MC. On the other hand, if the emission matrix is sparse to the point where all the entries are the same, then maximum uncertainty is imposed on the emission of the states. Thus, we consider this should be a user choice since the certainty about the data will depend on a case-by-case basis (e.g. quality of original reads, assembler used).

### Information in a k-mer

**Fig. 1:**
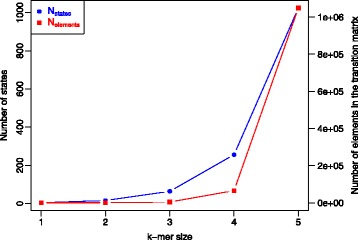
Size of the transition matrix and its implications. The number of states and the size of the transition matrix have been plotted as a function of the *k-mer* size. Note how the size of the transition matrix grows quadratically with the number of states. These, in turn, grow exponentially with the k-mer size

When breaking down a nucleic acid sequence into sub-strings, different k-mer sizes can be used. As previously introduced, the number of possible states in the hidden layer depends on the choice of k-mer size, since there would be as many as 4^*k*^ different states. Note that the size of the transition matrix grows quadratically with the number of states as Fig. 1 illustrates. Therefore, the memory requirements also grow quadratically with the size of k-mer used. In addition, there are processing time implications when using large k-mer sizes. However, one can benefit from using large k-mers. From an Information Theory perspective, the amount of information obtained from observing an event *A* with probability *p*_*A*_ is:

18 $$ I(A)=-\text{log}(p_{A})  $$

Therefore, as the cardinality of the state space grows, more information can be obtained. The average amount of information can be quantified by the well-known Shannon entropy:

19 $$ S=-\sum\limits_{A \in E}^{}p_{A}\text{log}(p_{A})  $$

When all k-mers in *E* are equally likely, (i.e. *p*_*A*_=|*E*|^−1^∀*A*∈*E*), we have that:

20 $$\begin{array}{*{20}l} S &=-\text{log}(|E|^{-1}) =-\text{log}(|N|^{-k}) \propto k \end{array} $$

Therefore, the amount of information is proportional to the k-mer size. This shows that, in fact, one can learn more from the sequence when larger k-mer’s are used. However, a trade-off between amount of information and computational requirements is often required since larger k-mer sizes imply more computational power.

### Adaptation of the forward algorithm

It is of interest to compute the probability of observing a given sequence of symbols given a HMM:

21 $$  P\left[Y=y;\Theta\right]  $$

This problem is referred to as the *scoring problem* [11]. In this case, the corresponding underlying sequence of states is not known. However, recall that by the LTP:

22 $$ P\left[Y=y;\Theta\right]=\sum\limits_{x \in \Omega_{m}}^{}P\left[Y=y,X=x;\Theta\right]  $$

Therefore, by considering all possible underlying sequences of states, one could eventually find the marginal probability of a given observation *P*[*Y*=*y*;*Θ*]. Nevertheless, this would not be feasible for long sequences of observations. As mentioned above, there would be as many as |*E*|^*m*^ possible combinations, where |*E*| is the cardinality of the state space and *m* the number of elements in a given symbol sequence. Therefore, this number grows exponentially with the length of the observation under consideration.

The forward algorithm was conceived to deal with this type of problem efficiently. It is based on dynamic programming, and can compute the probability of interest in a rather efficient way [12]. This computational approach consists in solving a complex problem by breaking it down into smaller problems that are much simpler, solving these and storing the solutions to finally find the answer to the larger problem by combining all these solutions. In this case, the following recursive variable is defined:

23 $$  \alpha(n,i)=P\left[Y_{1}=y_{1},\ldots, Y_{n}=y_{n},X_{n}=i;\Theta\right]  $$

and this variable is recursively computed (Eq. 24):

24 $$\begin{array}{@{}rcl@{}} \alpha(n,i)=\left\{\begin{array}{l} \pi_{0}(i)e(y_{1}|i), \; n=1 \\ \sum\limits_{s \in E}^{}\alpha(n-1,s)p_{si}e(y_{n}|i), \; n \geq 2 \end{array}\right. \end{array} $$

Where *p*_*si*_ is the transition probability from state *s* to *i*. Note that the cardinality of the state space *E* defines the number of recursive variables *α*(*n*,·). In addition, the number of iterations is equivalent to the number of k-mers the symbol sequence of interest has. Once the recursions are completed, the probability of interest can be computed as follows:

25 $$  P\left[Y=y;\Theta\right]=\sum\limits_{s \in E}\alpha(m,s)  $$

As a result, the probability *P*[*Y*=*y*;*Θ*] is obtained with a complexity of *O*(*m*|*E*|^2^). Note that the complexity of the algorithm is linear with respect to the number of symbols *m* in the sequence observed and quadratic with respect to the cardinality of the state space *E*. Comparing with the original computations, which complexity grows exponentially with the length of the sequence of symbols, this approach is significantly more efficient, especially when dealing with genome assemblies.

### Numerical stability

It can be seen in Eq. (24) that when computing the probability of a given observation, each recursive variable *α*(·,·) will be equal to the sum of very small numbers. As *m* becomes large, these numbers tend to zero and the same is true for the sum. For sufficiently large *m*, the dynamic range of the recursive variables computation will even exceed double-precision range [12].

There are two different ways of dealing with this problem [13], namely (i) by employing a log-transformation or (ii) by using scaling factors. Here, we will follow the scaling method proposed by [12]. Thus, we propose the following normalized score (Additional file 1):

26 $$  \hat{s} =-\frac{\sum\limits_{t=1}^{m}log(c_{t})}{k \cdot m}  $$

**Fig. 2:**
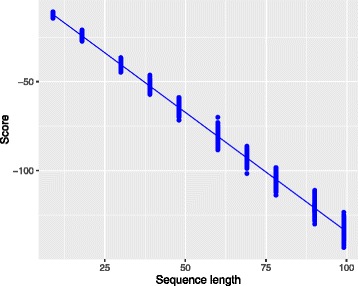
Linear relationship between contig length and non-normalized score. The non-normalized scores of the true sequences were plotted as a function of the sequence length and a linear model was fitted. A clear linear relationship between the non-normalized scores and the length of the sequences can be observed

**Fig. 3:**
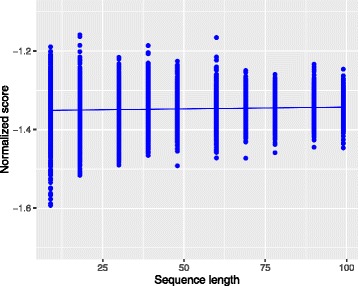
Scores after proposed normalization. The normalized scores of the true sequences were plotted as a function of the sequence length and a linear model was fitted. After the normalization, there is no longer correlation between the score and the sequence length

That way one can compare scores coming from sequences with different lengths since the length bias (see Fig. 2) is resolved. This score is usually referred to as *per-frame log-likelihood* in the speech processing field, where it is extensively used. The greater the score in Eq. (26), the closer to the reference the sequence is in terms of genetic patterns independently of its length. Note that Fig. 3 shows that, after normalizing the scores in Fig. 2, there is no longer correlation between the length of the sequence and the score.

### HiMMe’s workflow and genome assembly metric

**Fig. 4:**
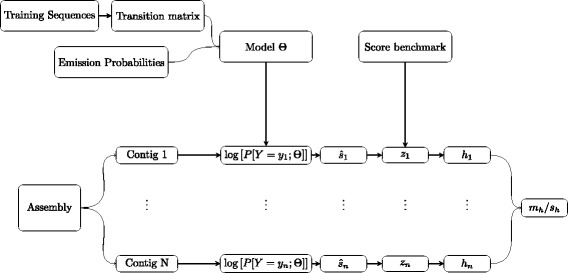
Flow chart of the methodology proposed

The workflow that we propose consists of four steps: (i) learning the transition matrix from the training set of sequences, (ii) defining the matrix of emission probabilities, (iii) scoring each contig in the assembly and (iv) computing the overall assembly metric. This workflow has been represented schematically in Fig. 4. Our package includes the following tools:

- himme_transition_matrix: builds the transition matrix from the training set of sequences. The output is a compressed tabulated text file that contains all transition probabilities.
- himme_emission_matrix: builds the matrix of emission probabilities. The output is a compressed tabulated text file that contains all emission probabilities. A SNP database in VCF format is expected as input. The emission probabilities can also be set manually based on base-calling precision or other metrics.
- himme_scoring: computes $\hat {s}$ for each contig in the assembly. The output is a compressed tabulated text file that contains the following information for each contig: ID of the contig, length of the contig, non-normalized score and normalized score.
- himme_summary: summarizes the output of himme_scoring. The following metrics are included in the output file: k-mer size used, number of contigs, median length, length variance, mean score, score variance, score interval C.I. 95%, mean corrected score, corrected score variance, corrected score 95% C.I. and HiMMe’s genome assembly coefficient.

The genome assembly coefficient provided by HiMMe is computed such that those assemblies with higher average normalized scores are rewarded. On the other hand, those assemblies with high normalized score variance are penalized. To that end, we take the z-score for each contig using a reference distribution drawn from real sequences. This reference distribution has been derived from a 30,000 normalized scores benchmark. Once the z-score for each contig is obtained, a logistic function is applied to map all scores to the unit interval [0,1], and finally the HiMMe coefficient is computed as follows:

27 $$  HiMMe_{coeff}=\frac{m_{h}}{s_{h}}  $$

where *m*_*h*_ and *s*_*h*_ denote respectively the average and the standard deviation of the logistic score obtained for a given assembly (Additional file 1). Note that, the numerator rewards those assemblies with high normalized scores, whereas the denominator rewards those assemblies with low standard deviations in their normalized scores distributions. Even though this metric represents an overall score for the assembly and makes it straightforward to rank them, we want to stress the importance of looking at each contig score individually as well as taking advantage of the fact that a distribution can be drawn from the output. Since a normalized score (or per-frame log-likelihood) is provided for each individual contig in the assembly, the user can filter the contigs based on this score in order to improve the reliability of the assembly for downstream analysis. In addition, statistical tests to compare distributions can be performed to find significant differences between assemblies.

## Results and discussion

We applied our novel method to two cases. In the first case, we considered simulated data to show how the algorithm is able to recognize genetic patterns in the input data. The results suggest that the proposed method is able to distinguish true sequences from random sequences with an accuracy that grows with the choice of k-mer size.

In the second case, we used our method to study the genome assemblies generated by the Genome Assembly Gold-Standard Evaluations (GAGE) project [14]. Several standard metrics have been computed for each assembly and are included in Table 5, along with the coefficient introduced in Eq. (27).

### Simulated data-set

The proposed algorithm has been used to score randomly generated sequences as well as sequences derived from a reference used to learn the transition matrix (Additional file 2). As genome reference, we chose the GRCh37 version of the human genome assembly [15]. A human SNP database was used to define the emission probabilities [16]. In this section, we show the results for sequences from length 10 nt to 100 nt with increments of 10 nt. The purpose of this comparison is to see whether the algorithm gives higher scores to those sequences that belong to the reference used to obtain the transition matrix.

#### Potential base composition biases

In order to avoid potential base composition biases, we measured the base composition of the reference used to draw sequences, the sequences drawn from it and the random sequences generated. This information can be found in Table 1. Differences in terms of base composition between the reference and the random sequences were found to be insignificant. Therefore, we consider this setting the worst-case scenario for our algorithm, since if there were a case where it would be favorable, this would be when the base composition significantly differed. That is, we intend to show that our method is not based in k-mer proportions, but in k-mer transitions, as opposed to other methods available.

**Table 1:** Base composition of the reference, the sequences drawn from the reference and the random sequences that were generated

Base composition simulated data-set			
Nucleotide	Reference	Sequences drawn	Random sequences
A	25.72%	25.68%	24.74%
C	24.86%	25.14%	25.06%
G	25.35%	25.15%	25.21%
T	24.08%	24.03%	24.98%

#### Discriminant power increases with k-mer size

**Fig. 5:**
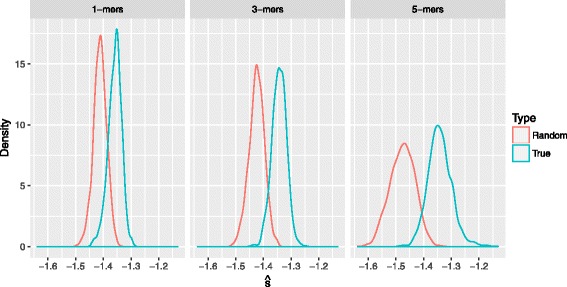
Random sequences and true sequences. The densities of the normalized scores using different k-mer sizes for simulated random sequences and true sequences have been plotted for length 100 nt. Note that the variance of both distributions grows with the k-mer size since the state space becomes larger. In addition, the distance between the medians grows as the k-mer size grows

Intuitively, one would expect to encounter more differences between scores as the k-mer size increases, since the algorithm should be able to identify more genetic patterns in the sequences. This is mainly due to the fact that the amount of information that can be contained in a k-mer, is proportional to *k*, as previously shown. As Fig. 5 demonstrates, the score distribution for the true sequences is always closer to zero compared to that of the random sequences (see the HiMMe coefficient in Table 2). It can be seen that, in general, the algorithm produced higher scores for those sequences that were sampled from the reference. However, some random sequences scored higher than some true sequences by chance. In addition, the score distributions deviate from each other more and more as the k-mer size used increases. Thus, the discriminant power of the method tends to increase with the k-mer size as we previously anticipated.

**Table 2:** HiMMe coefficient for sequences in Fig. 5

HiMMe coefficient		
k-mer size	True	Random
1	3.161	2.064
3	3.507	1.692
5	2.490	0.884

As expected, the coefficient is significantly greater for the true sequences than for the random sequences for all k-mer sizes

#### Effect of sequence length

**Fig. 6:**
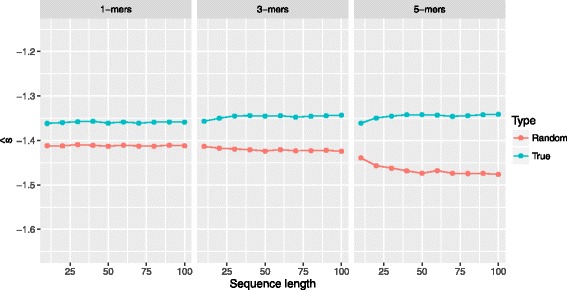
Dynamic range random and true sequences. The average normalized scores for the random and true sequences have been plotted for lengths between 10 nt and 100 nt. The sequence length does not have an effect on the normalized scores after these converge. In addition, when using larger k-mers to analyze the data, the difference in means becomes larger as well

Although we normalize the score based on the sequence length, we wondered whether this metric contributes to, or penalizes in any way, the discriminant power of our method. To that end, we run HiMMe on sequences from length 10 nt to length 100 nt and we plotted the average normalized score in Fig. 6. We verified that the discriminant power of our method increases with the k-mer size, since the lines diverge more with larger k-mer choices. However, we did not observe an effect of sequence length on differences between scores after the average normalized scores converged. We thus conclude that the sequence length neither benefits nor penalizes the discriminant power of our algorithm in any way.

#### Training a naive Bayes classifier with HiMMe’s output

In order to measure the discriminant power and understand the way it behaves in relation to the k-mer size choice, we fitted each obtained distribution to a Gaussian distribution. Once we obtained the distribution parameters, we were able to compute the naive Bayes classification error; i.e., the classification error if we were to use those distributions as likelihood functions in a classification problem setting (Table 3). As expected, the classification error decreased with the k-mer size. We observed a significant improvement when we used 3-mers instead of 1-mers, with the error dropping from 13.43*%* to 6.63*%*. This is consistent with the fact that, when larger k-mer sizes are used, more information can be captured by the algorithm.

**Table 3:** Classification errors if the densities obtained were to be used as likelihood functions in a naive Bayes classification problem setting

Naive Bayes classifier error		
1-mers	3-mers	5-mers
13.43%	6.63%	6.31%

#### Significance of discriminant power

Finally, a t-test was performed in order to compare the score distributions for k-mer sizes one, three, and five. The results are summarized in Table 4. The true sequences performed significantly better than the random sequences for all k-mer sizes. Note that all differences were found to be significant, even when the base composition was the same. In addition, larger k-mer sizes resulted in a more prominent difference in score distributions as expected.

**Table 4:** Statistics for the simulated data-set

t-test summary		
k-mer size	Difference in means (95% C.I.)	p-value
1	[0.0511,0.0553]	< 2.2e-16
3	[0.0788,0.0835]	< 2.2e-16
5	[0.1313,0.1390]	< 2.2e-16

For all the k-mer sizes the distributions are significantly different. The difference in means becomes larger as the k-mer size increases

### Analysis of the Genome Assembly Gold-Standard Evaluations data-set

**Fig. 7:**
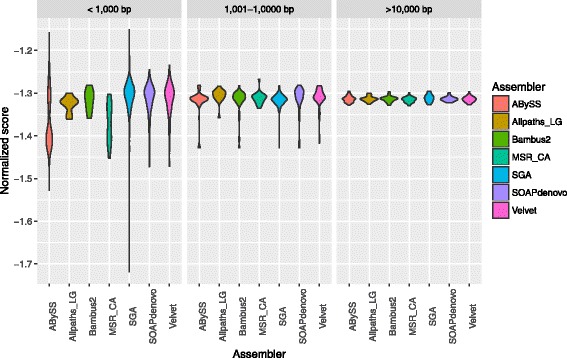
GAGE genome assemblies assessment. Violin plot of the normalized scores when analyzing the assemblies with k-mer size 3 for the seven assemblies generated in [14]. The scores have been binned by considering the size of the contigs

We used the proposed algorithm to score the seven *Staphylococcus Aureus* genome assemblies, generated by [14]. The first reference genome used to train the model pertained to the same species, i.e. *Staphylococcus Aureus*, and was downloaded from NCBI [17]. To show how our method performs in a *de-novo* setting, we also trained our algorithm with a related reference genome (same lineage). In this case we chose the reference genome of *Staphylococcus Saprophyticus*, also obtained from NCBI [18]. Regarding the emission matrix, we considered that the probability of observing the hidden nucleotide was 85% and the probability of finding a variant was 15% evenly distributed among the remaining three nucleotides (i.e. no bias). The score distributions have been plotted in Fig. 7 and have been binned into three different length subgroups.

We compared the following assemblers: ABySS [19], Allpaths-LG [20], Bambus2 [21], MSR-CA [22], SGA [23], SOAPdenovo [24] and Velvet [25]. For each assembly available, we summarized several metrics in Table 5 along with the coefficient generated by our method. The metrics included were: number of contigs, total length of the assembly, N50, NA50 [9], the percentage of the assembly that aligned back to the reference, the percentage of the genome covered by the assembly, BUSCO’s output (bacteria database available on their website), and the coefficient generated by HiMMe (Additional file 3).

**Table 5:** Metrics for the each of the assemblers generated in [14] when using Staphylococcus Aureus as reference

Metrics summary (*Staphylococcus Aureus*)							
Metric	ABySS	Allpaths-LG	Bambus2	MSR-CA	SGA	SOAP denovo	Velvet
*N*	5,065	51	105	98	6,854	182	301
*L*_*total*_ (bp)	3,489,706	1,674,547	2,277,291	2,862,552	3,448,095	2,631,096	2,860,307
*N*50 (kbp)	23.1	96.7	40.1	59.1	3.1	239.8	52.7
*NA*50 (kbp)	13.3	41.2	31.2	31.5	2.0	44.5	31.5
Aligned %	75%	83%	80%	83%	69%	81%	82%
Genome %	82%	51%	67%	87%	84%	78%	86%
*BUSCO*	15%	0%	2.5%	0%	40%	0%	2.5%
*HiMMe*_*coeff*_	1.412	9.053	6.928	4.217	5.036	3.953	4.606

*N* is the number of contigs in the assembler output. *L*_*total*_ represents the total length of the assemblers’ output. *N*50 is largest length *L* such that 50% of all nucleotides are contained in contigs of size at least *L* kbp. NA50 is a corrected version of N50 provided by QUAST [9]. Aligned % is the percentage of the assembly generated that was aligned back to the reference. Genome % is the fraction of the genome covered by the assembly generated. BUSCO’s score is the percentage of complete genes found in the assemblies from the bacteria database provided by BUSCO. Finally, *HiMMe*_*coeff*_ is the metric introduced in Eq. (27)

Previous studies suggest that it is very hard for an assembly to perform consistently well when assessed by multiple metrics [26]. Discrepancies might exist due to partial or complete orthogonality between the metrics themselves. For instance, the metric N50 measures the contiguity of an assembly but does not take into account the genetic content. Thus, it is perfectly plausible that an assembly has a high N50 even though the alignment rate is really low when mapping it to the reference.

Our method found Allpaths-LG’s output to be the best assembly by a wide margin. This assembly has very few contigs when compared to the average, its total length is the smallest and its median contig length is substantially above the average. In addition, it has the highest percentage alignment to the reference genome, an above average N50 and a rather superior NA50 compared to the other assemblies. When looking at BUSCO’s output, Allpaths-LG did not contain any homologous genes from the bacteria database in its output. As Fig. 7 shows, Allpaths-LG is the most consistent assembler throughout all the dynamic range of contig length (small variance in normalized score). The only caveat about the assembly generated by this tool is the fact that it only covers 51% of the reference genome. However, this is an orthogonal metric to the output of our algorithm and can be independently verified.

Bambus2 came in second after Allpaths-LG when looking at the coefficient generated by HiMMe. Similarly to Allpaths-LG, Bambus2 generated a rather short assembly with very few contigs in it and the contigs were found to be rather consistent throughout the whole length range as well (small variance in normalized score). Although its median contig length was rather high, clearly above average, its N50 was not as high as with Allpaths-LG. On the other hand, the percentage of the genome covered was higher, and its NA50 and aligned percentage were among the best. In this case, BUSCO was able to find 2.5% of the genes in the bacteria database, which is not as high as expected considering the other metrics.

While far from Allpaths-LG and Bambus2, the HiMMe coefficient was also above average for SGA. In this case, BUSCO was able to find 40% of the homologous genes in its output, being the highest percentage across all assemblies, and 84% of the reference genome was recovered by the assembly. However, SGA was the assembly with the highest number of contigs, a really small median contig length and, as a consequence, the smallest N50 and NA50, as well as smallest fraction of the assembly aligned. Figure 7 reveals a rather high variance for contigs smaller than 1000 bp with quite an important fraction of contigs scoring very low. This observation would allow the researcher to filter some of those contigs in order to obtain a higher quality assembly. For contigs larger than 1000 bp, SGA generated rather reliable contigs.

Both SOAPdenovo and Velvet performed similarly in all metrics except for N50. Although Velvet’s N50 was quiet low, SOAPdenovo’s N50 was well above average. Nevertheless, both NA50’s were among the highest. The fraction of the assembly aligned was rather high in both cases, and so was the percentage of the genome covered by both assemblies. As with SGA, the distribution of normalized scores is rather sparse for contigs smaller than 1000 bp with some low scores. This again suggests that these assemblies could be improved by filtering out these contigs of small size and with low normalized scores. As for BUSCO, no matches were identified for SOAPdenovo, while only 2.5% of the genes in the database were identified in the assembly generated by Velvet.

MSR-CA generated an assembly of high quality specially when looking at contigs over 1000 bp. The total length of the assembly generated was very close to the length of the reference genome, and almost all the metrics were among the best achieving the highest fraction of the genome covered. However, the HiMMe coefficient was slightly below average, mainly due to the issue with short contigs which represent a 30% of the total assembly. Nevertheless, as Fig. 7 shows, although the distribution of normalized scores for short contigs (under 1000 bp) was rather poor, when looking at larger contigs, MSR-CA appeared to be among the best. As for BUSCO, the tool was not able to find any homologous genes in this case.

Finally, the assembly generated by ABySS scored very low in multiple facets of the analysis. Its N50 and NA50 were among the lowest (2nd lowest in both cases). The fraction of the assembly that was aligned back to the reference was the lowest one (only 75%), although the fraction of the genome covered by the assembly was high. As for HiMMe, both the coefficient generated and the distribution of normalized scores (see Fig. 7) suggest that the assembly generated contains a fair amount of contigs of rather low quality. On the other hand, BUSCO was able to find about 15% of homologous genes, being this the second highest percentage.

We also proceeded to run our algorithm training it with a relatively close reference genome (*Staphylococcus Saprophyticus* instead of *Staphylococcus Aureus*). The goal of this experiment was to show that, while the alignment rates are misleading when not using the exact reference, our algorithm can still be used and the results obtained are close to those obtained when using the exact reference. As Table 6 shows, the genome coverage obtained when aligning the assembly to the reference was very low (under 1% in all cases). On the other hand, the scores produced by HiMMe were rather close to those obtained with the exact reference genome, leading to the same conclusions. This is due to the fact that, although the genetic content might be very similar, the way the genome is arranged can be rather different. On the other hand, the transition matrices were surprisingly close when comparing the one obtained from the true reference (i.e. *Staphylococcus Aureus*) to that of the relatively close reference (i.e. *Staphylococcus Saprophyticus*). That allowed our method to produce reliable metrics in a simulated *de-novo* setting.

**Table 6:** Percentage of the genome covered by the assembly (using QUAST) and *HIMME*_*coeff*_ obtained when using a relatively close reference (Staphylococcus Saprophyticus instead of Staphylococcus Aureus)

Using a different reference: *Staphylococcus Saprophyticus*							
Metric	ABySS	Allpaths-LG	Bambus2	MSR-CA	SGA	SOAP denovo	Velvet
Genome %	0.241%	0.120%	0.231%	0.637%	0.354%	0.254%	0.322%
*HiMMe*_*coeff*_	1.399	9.025	6.936	4.126	4.986	3.973	4.588

## Conclusions

The method presented in this article is capable of identifying genetic patterns in data regardless of the underlying base composition. The most frequent transitions are learned from the training set of sequences. By adding emission probabilities in the formulation, the method allows for biological variability and errors in all intermediate steps of the process (e.g. sequencing errors, assembler errors).

The transition matrix can contain as much previous knowledge about a certain organism as one desires and it is not required to be learned strictly from the reference genome of an organism. For instance, the transition matrix could be learned from the reference genome of a closely related species or even from a gene database. In addition, uncertainty can be introduced to the system by using a sparse emission matrix.

We have shown that our algorithm has a significant discriminant power even when using 1-mers, with the power increasing for large k-mer sizes. Appreciable improvement was observed when using 3-mers instead of 1-mers, reducing the naive Bayes classifier error by a half. However, we did not observe much improvement when using 5-mers instead of 3-mers. Therefore, k-mer size three was a good trade-off between performance and computational requirements in this case. Nevertheless, other k-mer sizes might be a better fit in other cases, depending on the complexity of the organisms and the nature of the study.

In addition to a score for each contig, our method also provides an overall metric for the entire assembly. After analyzing the assemblies from [14] we have found Allpaths-LG’s output to be the best assembly when looking exclusively at our coefficient. These results were consistent with standard metrics. On the other hand, our method showed ABYSS’ assembly to be the worst one. These results are consistent with the fact that both the fraction of assembly aligned and the resulting NA50 were rather low.

Our method presents several advantages when compared to other genome assembly assessment alternatives. Besides an overall score for the entire assembly, our method includes an individual score for each contig (per-frame log-likelihood). Thus, our method allows the use of a cut-off value to filter contigs, which can be used to improve the reliability of the assembly for downstream analysis. We want to emphasize again the importance of looking at the normalized score distribution and using this distribution to perform statistical tests to find significant differences between assemblies. For instance, ABySS’ assembly could be improved by filtering out those contigs with low scores (specially short contigs) and, as a result, improve the overall quality of the assembly.

In addition, the time complexity of our method is significantly smaller than that of an aligner such as BLAST or HMMER and, in turn, much smaller than BUSCO’s. Moreover, our method does not use any heuristics as opposed to BLAST and other multiple sequence aligners. However, we want to stress the fact that the purpose of our work was not to program a fast tool at this point, but instead demonstrate that HMMs provide an excellent framework for assessing genome assemblies without a need for alignment tools and with limited computational resources.

Finally, our model can be trained not only by using a reference genome but by employing any FASTA file. Thus, even when the reference genome is not available (i.e. in a *de-novo* genome assembly setting) our tool could still be trained with a set of homologous genes or with the reference genome of a closely related species and still provide the researcher with well-founded metrics. Under these circumstances, it would not be possible to assess the quality of a genome assembly via alignment rate, since there would be no reference genome available and a closely related genome would be problematic.

The scoring method for genome assemblies proposed in this article must be regarded as complementary to well-known standards used by the scientific community for evaluation (e.g., N50, median contig length, etc.). It is also complementary to the scoring methods that software like BUSCO or QUAST provide. Each metric focuses on a particular aspect of the problem and, thus, we recommend using as many metrics as possible when assessing genome assemblies so that more factors can be taken into account. In agreement with [26], we recommend not to place much faith in a single metric and instead take several into account when possible.

Finally, the main objective of the method introduced in this article is to use the genetic patterns as a proxy for genome assembly reliability. As a future direction, we will explore other potential applications of the proposed model. For instance, using the well-known Viterbi algorithm errors could be identified, reported and fixed. In addition, the same scoring method could be used to assess transcriptome assemblies or to classify meta-genomics data, considering the potential of our method for classification. However, the discriminant power requirements will depend on the application, and a trade-off between discriminant power and computational requirements might be required in some cases.

## Additional files

Additional file 1 ’Supplementary_file1.pdf’. Title: ’Derivation normalized score & HiMMe coefficient’. Description: derivation of the normalized score as well as the HiMMe coefficient. (PDF 94 kb)

Additional file 2 ’Supplementary_file2.zip’, Title: ’Data from simulations’. Description: contains all simulated data as well as the pertinent results. (ZIP 1187 kb)

Additional file 3 ’Supplementary_file3.zip’, Title: ’Data from GAGE’. Description: contains all real data as well as the pertinent results. (ZIP 18,698 kb)

## Abbreviations

A: Adenine
bp: Base pair
C: Cytosine
cDNA: Complementary deoxyribonucleic acid
DNA: Deoxyribonucleic acid
G: Guanine
HMM: Hidden Markov model
kb: Kilobase
LTP: Law of total probability
NCBI: National center for biotechnology information
nt: Nucleotides
SNP: Single nucleotide polymorphism
RNA: Ribonucleic acid
T: Thymine
U: Uracil
VCF: Variant call format
