# Supplementary material for: HiMMe: using genetic patterns as a proxy for genome assembly reliability assessment
Source: BMC Genomics. 2017 Sep 5;18:694. doi: 10.1186/s12864-017-3965-2 (PMC5584555; doi:10.1186/s12864-017-3965-2)
Supplement: Supplementary file 1 — ’Supplementary_file1.pdf’. Title: ’Derivation normalized score & HiMMe coefficient’. Description: derivation of the normalized score as well as the HiMMe coefficient. (PDF 94 kb) [file 12864_2017_3965_MOESM1_ESM.pdf]

Supplementary File 1

Forward Algorithm

The following coefficient c_t is used at each iteration to scale the recursive variable α:

$$c_t = \frac{1}{\sum\limits_{i \in E} \alpha(t,i)} \tag{1}$$

Then, for a given iteration step, we first calculate:

$$\alpha(t,i) = \sum_{s \in E} \hat{\alpha}(t-1,s) p_{si} e(y_t|i) \tag{2}$$

The scaled recursive variable is:

$$\hat{\alpha}(t,i) = \frac{\sum\limits_{s \in E} \hat{\alpha}(t-1,s) p_{si} e(y_t|i)}{\sum\limits_{j \in E} \sum\limits_{s \in E} \hat{\alpha}(t-1,s) p_{sj} e(y_t|j)} \tag{3}$$

By induction, $\hat{\alpha}(t-1,s)$ can be written as:

$$\begin{aligned} \hat{\alpha}(t-1,s) &= \left[\prod_{\tau=1}^{t-1} c_\tau \right] \alpha(t-1,s) \\ &= C_{t-1} \alpha(t-1,s) \end{aligned} \tag{4}$$

Hence, the scaled recursive variable can be written as:

$$\hat{\alpha}(t,i) = \frac{\sum\limits_{s \in E} C_{t-1} \alpha(t-1,s) p_{si} e(y_t|i)}{\sum\limits_{j \in E} \sum\limits_{s \in E} C_{t-1} \alpha(t-1,s) p_{sj} e(y_t|j)} \tag{5}$$

which can be simplified as:

$$\hat{\alpha}(t,i) = \frac{\alpha(t,i)}{\sum\limits_{j \in E} \alpha(t,j)} \tag{6}$$

Note that:

$$\sum_{i\in E}\hat{\alpha}(m,i) = \sum_{i\in E}\left[\prod_{t=1}^{m} c_t\right]\alpha(m,i)$$
$$= \prod_{t=1}^{m} c_t \sum_{i\in E}\alpha(m,i) = 1 \tag{7}$$

Hence, the marginal probability of a given observation $Y = y$ can be expressed as:

$$P\left[Y = y;\Theta\right] = \left(\prod_{t=1}^{m} c_t\right)^{-1} \tag{8}$$

Since this number would still be too small even for double-precision, it is suggested in to use:

$$\log\left[P\left[Y = y;\Theta\right]\right] = -\sum_{t=1}^{m} log(c_t) \tag{9}$$

As the sequence being analyzed becomes larger, this score decreases since the state space grows and it is less likely to observe any given sequence. Nevertheless, a linear relationship exists between this quantity and the length of the sequence as we experimentally show. Thus, we propose the following normalized score:

$$\hat{s} = -\frac{\sum_{t=1}^{m} log(c_t)}{k\cdot m} \tag{10}$$

HiMMe coefficient

A reference distribution has been derived from a 30,000 normalized scores benchmark. Using this distribution as a reference, a z-score is computed for each contig in the assembly. The logistic function $h(z) = 1/(1+\exp(-z))$ is used in order to map all z-scores to the interval $[0, 1]$. Note that this is a monotonic transformation and, as a result, does not alter the

rankings based on z-scores. The HiMMe coefficient is then obtained by:

$$m_h = \frac{1}{N_c} \sum_{i=1}^{N_c} h_i$$

$$s_h = \sqrt{\frac{1}{N_c - 1} \sum_{i=1}^{N_c} (h_i - m_h)^2}$$

$$HiMMe_{coeff} = \frac{m_h}{s_h}$$

where N_c is the number of contigs available for a given assembly. This coefficient is the reciprocal of the coefficient of variation, which in turn is vastly used in engineering, physics and other fields to measure the dispersion or volatility of a given process.
