# Supplementary material for: HiMMe: using genetic patterns as a proxy for genome assembly reliability assessment
Source: BMC Genomics. 2017 Sep 5;18:694. doi: 10.1186/s12864-017-3965-2 (PMC5584555; doi:10.1186/s12864-017-3965-2)
Supplement: Supplementary file 3 — ’Supplementary_file3.zip’, Title: ’Data from GAGE’. Description: contains all real data as well as the pertinent results. (ZIP 18,698 kb) [file 12864_2017_3965_MOESM3_ESM.zip › Real data/QUAST/lineage/SGA/report.html]

|  |
| --- |
| QUAST **Quality Assessment Tool for Genome Assemblies** by CAB |

Loading...

Aligned to
""

Combined reference
Estimated reference size:
 bp
|
 bp
|
 references
|
 fragments
|
 % G+C
|
 chromosomes
  
 reads
|
 mapped
|
 properly paired
  
 genes
|
 operons

Unfortunately, JavaScript in your
browser is disabled or is not supported.
We need JavaScript to build report and plots.

Worst
Median
Best

Show heatmap

|  |  |
| --- | --- |
|  |  |
|  | |  |
| Contigs are ordered from largest (contig #1) to smallest.  FRCurve: Y is the total number of aligned bases divided by the reference length, in the contigs having the total number of  at most X.  Contigs are broken into nonoverlapping 100 bp windows. Plot shows number of windows for each GC percentage.  Plot shows number of contigs with GC percentage in a certain range. | | |

{"subreports":[],"report":[["Genome statistics",[{"values":["0.354"],"quality":"More is better","isMain":true,"metricName":"Genome fraction (%)"},{"values":["2.991"],"quality":"Less is better","isMain":true,"metricName":"Duplication ratio"},{"values":[2981],"quality":"More is better","isMain":true,"metricName":"Largest alignment"},{"values":[25704],"quality":"More is better","isMain":true,"metricName":"Total aligned length"},{"values":[4404],"quality":"More is better","isMain":false,"metricName":"NG50"},{"values":[2585],"quality":"More is better","isMain":false,"metricName":"NG75"},{"values":[null],"quality":"More is better","isMain":true,"metricName":"NGA50"},{"values":[188],"quality":"Less is better","isMain":false,"metricName":"LG50"},{"values":[378],"quality":"Less is better","isMain":false,"metricName":"LG75"}]],["Misassemblies",[{"values":[0],"quality":"Less is better","isMain":true,"metricName":"# misassemblies"},{"values":[0],"quality":"Less is better","isMain":false,"metricName":" # relocations"},{"values":[0],"quality":"Less is better","isMain":false,"metricName":" # translocations"},{"values":[0],"quality":"Less is better","isMain":false,"metricName":" # inversions"},{"values":[0],"quality":"Less is better","isMain":false,"metricName":"# misassembled contigs"},{"values":[0],"quality":"Less is better","isMain":true,"metricName":"Misassembled contigs length"},{"values":[0],"quality":"Less is better","isMain":false,"metricName":"# local misassemblies"},{"values":[0],"quality":"Less is better","isMain":false,"metricName":"# unaligned mis. contigs"}]],["Unaligned",[{"values":[6660],"quality":"Less is better","isMain":false,"metricName":"# fully unaligned contigs"},{"values":[3406979],"quality":"Less is better","isMain":false,"metricName":"Fully unaligned length"},{"values":[4],"quality":"Less is better","isMain":false,"metricName":"# partially unaligned contigs"},{"values":[13785],"quality":"Less is better","isMain":false,"metricName":"Partially unaligned length"}]],["Mismatches",[{"values":[192],"quality":"Less is better","isMain":false,"metricName":"# mismatches"},{"values":[19],"quality":"Less is better","isMain":false,"metricName":"# indels"},{"values":[23],"quality":"Less is better","isMain":false,"metricName":"Indels length"},{"values":["2101.12"],"quality":"Less is better","isMain":true,"metricName":"# mismatches per 100 kbp"},{"values":["207.92"],"quality":"Less is better","isMain":true,"metricName":"# indels per 100 kbp"},{"values":[19],"quality":"Less is better","isMain":false,"metricName":" # indels (<= 5 bp)"},{"values":[0],"quality":"Less is better","isMain":false,"metricName":" # indels (> 5 bp)"},{"values":[0],"quality":"Less is better","isMain":false,"metricName":"# N's"},{"values":["0.00"],"quality":"Less is better","isMain":true,"metricName":"# N's per 100 kbp"}]],["Statistics without reference",[{"values":[6854],"quality":"Equal","isMain":true,"metricName":"# contigs"},{"values":[6854],"quality":"Equal","isMain":false,"metricName":"# contigs (>= 0 bp)"},{"values":[764],"quality":"Equal","isMain":false,"metricName":"# contigs (>= 1000 bp)"},{"values":[147],"quality":"Equal","isMain":false,"metricName":"# contigs (>= 5000 bp)"},{"values":[20],"quality":"Equal","isMain":false,"metricName":"# contigs (>= 10000 bp)"},{"values":[0],"quality":"Equal","isMain":false,"metricName":"# contigs (>= 25000 bp)"},{"values":[0],"quality":"Equal","isMain":false,"metricName":"# contigs (>= 50000 bp)"},{"values":[16870],"quality":"More is better","isMain":true,"metricName":"Largest contig"},{"values":[3448095],"quality":"More is better","isMain":true,"metricName":"Total length"},{"values":[3448095],"quality":"More is better","isMain":false,"metricName":"Total length (>= 0 bp)"},{"values":[2583186],"quality":"More is better","isMain":true,"metricName":"Total length (>= 1000 bp)"},{"values":[1097906],"quality":"More is better","isMain":false,"metricName":"Total length (>= 5000 bp)"},{"values":[264524],"quality":"More is better","isMain":true,"metricName":"Total length (>= 10000 bp)"},{"values":[0],"quality":"More is better","isMain":false,"metricName":"Total length (>= 25000 bp)"},{"values":[0],"quality":"More is better","isMain":true,"metricName":"Total length (>= 50000 bp)"},{"values":[3144],"quality":"More is better","isMain":false,"metricName":"N50"},{"values":[991],"quality":"More is better","isMain":false,"metricName":"N75"},{"values":[304],"quality":"Less is better","isMain":false,"metricName":"L50"},{"values":[767],"quality":"Less is better","isMain":false,"metricName":"L75"},{"values":["31.99"],"quality":"Equal","isMain":false,"metricName":"GC (%)"}]],["Predicted genes",[]],["Similarity statistics",[{"values":[0],"quality":"Equal","isMain":false,"metricName":"# similar correct contigs"},{"values":[0],"quality":"Equal","isMain":false,"metricName":"# similar misassembled blocks"}]],["Reference statistics",[{"values":[2577899],"quality":"Equal","isMain":false,"metricName":"Reference length"},{"values":[3],"quality":"Equal","isMain":false,"metricName":"Reference fragments"},{"values":["33.19"],"quality":"Equal","isMain":false,"metricName":"Reference GC (%)"}]]],"assembliesWithNs":null,"referenceName":"staphylococcus\_saprophyticus","date":"19 June 2017, Monday, 19:18:19","subreferences":[],"minContig":0,"order":[0],"assembliesNames":["genome.ctg.filtered"]}

{{ qualities }}

{{ mainMetrics }}

{"lists\_of\_lengths":[[65237,59023,51538,46344,42382,38446,36702,34655,33834,32599,31643,30884,29984,29071,28236,27197,26897,26570,26110,25832,25298,24658,24181,23872,23622,23138,22849,22561,22140,21954,21717,21270,20863,20750,20527,20233,20078,19718,19352,19192,19038,18729,18404,18227,18050,17874,17667,17540,17358,17204,17036,16729,16560,16449,16391,16267,16025,15728,15557,15432,15243,15073,14932,14690,14457,14215,14037,13867,13606,13443,13254,13149,13047,12799,12737,12651,12545,12267,12011,11813,11730,11669,11608,11565,11455,11349,11256,11172,11062,10834,10719,10637,10527,10427,10332,10254,10033,9902,9783,9738,9675,9583,9378,9329,9247,9215,9149,9099,9001,8924,8848,8675,8587,8534,8430,8300,8233,8145,8087,8040,7978,7924,7855,7790,7655,7629,7562,7525,7456,7377,7271,7139,7076,6998,6944,6914,6886,6840,6812,6718,6692,6646,6591,6552,6515,6462,6411,6330,6250,6207,6109,6004,5966,5943,5887,5817,5774,5704,5657,5602,5508,5450,5396,5363,5322,5295,5241,5191,5166,5130,5065,4990,4917,4881,4859,4805,4742,4706,4667,4535,4472,4440,4379,4358,4288,4240,4194,4148,4103,4056,4022,3977,3936,3906,3867,3810,3783,3758,3729,3689,3654,3622,3598,3540,3490,3467,3428,3396,3381,3345,3298,3258,3231,3210,3156,3123,3087,3029,2991,2947,2927,2900,2872,2807,2770,2738,2694,2634,2601,2578,2566,2538,2506,2449,2423,2384,2340,2306,2276,2246,2216,2178,2133,2083,2057,2043,2000,1974,1959,1945,1911,1881,1831,1803,1772,1745,1720,1686,1653,1638,1630,1607,1592,1577,1561,1548,1520,1506,1482,1456,1447,1427,1375,1323,1281,1264,1244,1219,1195,1176,1155,1138,1123,1108,1099,1082,1064,1044,1036,1031,1021,1009,998,985,973,959,948,933,928,923,915,902,894,881,867,858,847,841,832,824,818,812,803,794,789,786,778,776,770,762,753,745,737,736,730,727,724,722,715,711,707,701,694,692,689,685,682,680,680,676,673,672,668,667,664,662,660,655,652,652,651,648,644,642,637,633,632,630,628,628,628,627,624,624,624,623,620,620,620,620,616,616,616,616,612,612,612,612,612,612,608,608,608,606,604,604,604,604,603,600,600,600,597,596,596,595,592,592,592,589,588,588,588,588,586,584,584,584,584,582,580,580,580,577,576,576,576,575,572,572,569,568,568,568,568,566,564,564,564,562,560,560,560,560,560,560,556,556,556,556,552,552,552,552,552,548,548,548,548,548,548,548,544,544,544,544,544,542,540,540,540,540,540,539,536,536,536,536,533,532,532,532,531,528,528,528,526,524,524,524,523,520,520,520,520,519,516,516,516,516,516,515,512,512,512,512,512,509,508,508,507,504,504,504,504,504,501,500,500,500,499,496,496,496,495,492,492,492,492,492,492,492,492,488,488,488,488,488,487,484,484,484,484,484,484,484,480,480,480,480,480,480,478,476,476,476,476,476,475,472,472,472,472,472,471,468,468,468,468,468,468,465,464,464,464,464,464,464,464,463,460,460,460,460,460,460,460,457,456,456,456,456,456,456,456,455,452,452,452,452,452,452,452,452,452,451,448,448,448,448,448,448,448,448,448,445,444,444,444,444,444,444,444,444,444,444,444,444,440,440,440,440,440,440,440,440,440,440,440,440,440,437,436,436,436,436,436,436,436,436,436,436,436,436,436,436,434,432,432,432,432,432,432,432,432,432,432,432,432,432,432,432,432,432,428,428,428,428,428,428,428,428,428,428,428,428,428,428,428,428,428,428,428,428,428,428,428,425,424,424,424,424,424,424,424,424,424,424,424,424,424,424,424,424,424,424,424,424,424,424,424,424,424,424,424,424,424,424,422,420,420,420,420,420,420,420,420,420,420,420,420,420,420,420,420,420,420,420,420,420,420,420,420,420,420,420,420,420,420,420,420,420,420,420,420,419,416,416,416,416,416,416,416,416,416,416,416,416,416,416,416,416,416,416,416,416,416,416,416,416,416,416,416,416,416,416,416,416,416,416,416,416,416,416,416,416,416,416,416,416,416,416,416,416,416,412,412,412,412,412,412,412,412,412,412,412,412,412,412,412,412,412,412,412,412,412,412,412,412,412,412,412,412,412,412,412,412,412,412,412,412,412,412,412,412,412,412,412,412,412,412,412,412,412,412,412,412,412,412,412,412,412,412,412,412,412,412,412,412,408,408,408,408,408,408,408,408,408,408,408,408,408,408,408,408,408,408,408,408,408,408,408,408,408,408,408,408,408,408,408,408,408,408,408,408,408,408,408,408,408,408,408,408,408,408,408,408,408,408,408,408,408,408,408,408,408,408,408,408,408,408,408,408,408,408,408,408,408,408,408,408,408,408,408,408,408,408,408,408,408,408,408,408,408,408,408,408,408,408,408,408,408,408,408,408,408,408,408,408,408,408,408,408,408,408,408,408,404,404,404,404,404,404,404,404,404,404,404,404,404,404,404,404,404,404,404,404,404,404,404,404,404,404,404,404,404,404,404,404,404,404,404,404,404,404,404,404,404,404,404,404,404,404,404,404,404,404,404,404,404,404,404,404,404,404,404,404,404,404,404,404,404,404,404,404,404,404,404,404,404,404,404,404,404,404,404,404,404,404,404,404,404,404,404,404,404,404,404,404,404,404,404,404,404,404,404,404,404,404,404,404,404,404,404,404,404,404,404,404,404,404,404,404,404,404,404,404,404,404,404,404,404,404,404,404,404,404,404,404,404,404,404,404,404,404,404,404,404,404,404,404,404,404,404,404,404,404,404,404,404,404,404,404,404,404,404,404,404,404,404,404,404,404,404,404,404,404,404,404,404,404,404,404,404,404,404,404,404,404,404,404,404,404,404,404,404,404,404,404,404,404,404,404,404,404,404,404,404,404,404,404,404,404,404,404,404,404,404,404,404,404,404,404,404,404,404,404,404,404,404,404,404,404,404,404,404,404,404,404,404,404,404,404,404,404,404,404,404,404,404,404,404,404,404,404,404,404,404,404,404,404,404,404,404,404,404,404,404,404,404,404,404,404,404,404,404,404,404,404,404,404,404,404,404,404,404,404,404,404,404,404,404,404,404,404,404,404,404,404,404,404,404,404,404,404,404,404,404,404,404,404,404,404,404,404,404,404,404,404,404,404,404,404,404,404,404,404,404,404,404,404,404,404,404,404,404,404,404,404,404,404,404,404,404,404,404,404,404,404,404,404,404,404,404,404,404,404,404,404,404,404,404,404,404,404,404,404,404,404,404,404,404,404,404,404,404,404,404,404,404,404,404,404,404,404,404,404,404,404,404,404,404,404,404,404,404,404,404,404,404,404,404,404,404,404,404,404,404,404,404,404,404,404,404,404,404,404,404,404,404,404,404,404,404,404,404,404,404,404,404,404,404,404,404,404,404,404,404,404,404,404,404,404,404,404,404,404,404,404,404,404,404,404,404,404,404,404,404,404,404,404,404,404,404,404,404,404,404,404,404,404,404,404,404,404,404,404,404,404,404,404,404,404,404,404,404,404,404,404,404,404,404,404,404,404,404,404,404,404,404,404,404,404,404,404,404,404,404,404,404,404,404,404,404,404,404,404,404,404,404,404,404,404,404,404,404,404,404,404,404,404,404,404,404,404,404,404,404,404,404,404,404,404,404,404,404,404,404,404,404,404,404,404,404,404,404,404,404,404,404,404,404,404,404,404,404,404,404,404,404,404,404,404,404,404,404,404,404,404,404,404,404,404,404,404,404,404,404,404,404,404,404,404,404,404,404,404,404,404,404,404,404,404,404,404,404,404,404,404,404,404,404,404,404,404,404,404,404,404,404,404,404,404,404,404,404,404,404,404,404,404,404,404,404,404,404,404,404,404,404,404,404,404,404,404,404,404,404,404,404,404,404,404,404,404,404,404,404,404,404,404,404,404,404,404,404,404,404,404,404,404,404,404,404,404,404,404,404,404,404,404,404,404,404,404,404,404,404,404,404,404,404,404,404,404,404,404,404,404,404,404,404,404,404,404,404,404,404,404,404,404,404,404,404,404,404,404,404,404,404,404,404,404,404,404,404,404,404,404,404,404,404,404,404,404,404,404,404,404,404,404,404,404]],"filenames":["genome.ctg.filtered"]}

{"assemblies\_lengths":[3448095],"filenames":["genome.ctg.filtered"]}

{"reflen":[2577899,0,0,0,0,0,0,0,0,0,0,0,0,0,0,0,0,0,0,0,0,0,0,0,0,0,0,0,0,0,0,0,0,0,0,0,0,0,0,0,0,0,0,0,0,0,0,0,0,0,0,0,0,0,0,0,0,0,0,0,0,0,0,0,0,0,0,0,0,0,0,0,0,0,0,0,0,0,0,0,0,0,0,0,0,0,0,0,0,0,0,0,0,0,0,0,0,0,0,0,0,0,0,0,0,0,0,0,0,0,0,0,0,0,0,0,0,0,0,0,0,0,0,0,0,0,0,0,0,0,0,0,0,0,0,0,0,0,0,0,0,0,0,0,0,0,0,0,0,0,0,0,0,0,0,0,0,0,0,0,0,0,0,0,0,0,0,0,0,0,0,0,0,0,0,0,0,0,0,0,0,0,0,0,0,0,0,0,0,0,0,0,0,0,0,0,0,0,0,0,0,0,0,0,0,0,0,0,0,0,0,0,0,0,0,0,0,0,0,0,0,0,0,0,0,0,0,0,0,0,0,0,0,0,0,0,0,0,0,0,0,0,0,0,0,0,0,0,0,0,0,0,0,0,0,0,0,0,0,0,0,0,0,0,0,0,0,0,0,0,0,0,0,0,0,0,0,0,0,0,0,0,0,0,0,0,0,0,0,0,0,0,0,0,0,0,0,0,0,0,0,0,0,0,0,0,0,0,0,0,0,0,0,0,0,0,0,0,0,0,0,0,0,0,0,0,0,0,0,0,0,0,0,0,0,0,0,0,0,0,0,0,0,0,0,0,0,0,0,0,0,0,0,0,0,0,0,0,0,0,0,0,0,0,0,0,0,0,0,0,0,0,0,0,0,0,0,0,0,0,0,0,0,0,0,0,0,0,0,0,0,0,0,0,0,0,0,0,0,0,0,0,0,0,0,0,0,0,0,0,0,0,0,0,0,0,0,0,0,0,0,0,0,0,0,0,0,0,0,0,0,0,0,0,0,0,0,0,0,0,0,0,0,0,0,0,0,0,0,0,0,0,0,0,0,0,0,0,0,0,0,0,0,0,0,0,0,0,0,0,0,0,0,0,0,0,0,0,0,0,0,0,0,0,0,0,0,0,0,0,0,0,0,0,0,0,0,0,0,0,0,0,0,0,0,0,0,0,0,0,0,0,0,0,0,0,0,0,0,0,0,0,0,0,0,0,0,0,0,0,0,0,0,0,0,0,0,0,0,0,0,0,0,0,0,0,0,0,0,0,0,0,0,0,0,0,0,0,0,0,0,0,0,0,0,0,0,0,0,0,0,0,0,0,0,0,0,0,0,0,0,0,0,0,0,0,0,0,0,0,0,0,0,0,0,0,0,0,0,0,0,0,0,0,0,0,0,0,0,0,0,0,0,0,0,0,0,0,0,0,0,0,0,0,0,0,0,0,0,0,0,0,0,0,0,0,0,0,0,0,0,0,0,0,0,0,0,0,0,0,0,0,0,0,0,0,0,0,0,0,0,0,0,0,0,0,0,0,0,0,0,0,0,0,0,0,0,0,0,0,0,0,0,0,0,0,0,0,0,0,0,0,0,0,0,0,0,0,0,0,0,0,0,0,0,0,0,0,0,0,0,0,0,0,0,0,0,0,0,0,0,0,0,0,0,0,0,0,0,0,0,0,0,0,0,0,0,0,0,0,0,0,0,0,0,0,0,0,0,0,0,0,0,0,0,0,0,0,0,0,0,0,0,0,0,0,0,0,0,0,0,0,0,0,0,0,0,0,0,0,0,0,0,0,0,0,0,0,0,0,0,0,0,0,0,0,0,0,0,0,0,0,0,0,0,0,0,0,0,0,0,0,0,0,0,0,0,0,0,0,0,0,0,0,0,0,0,0,0,0,0,0,0,0,0,0,0,0,0,0,0,0,0,0,0,0,0,0,0,0,0,0,0,0,0,0,0,0,0,0,0,0,0,0,0,0,0,0,0,0,0,0,0,0,0,0,0,0,0,0,0,0,0,0,0,0,0,0,0,0,0,0,0,0,0,0,0,0,0,0,0,0,0,0,0,0,0,0,0,0,0,0,0,0,0,0,0,0,0,0,0,0,0,0,0,0,0,0,0,0,0,0,0,0,0,0,0,0,0,0,0,0,0,0,0,0,0,0,0,0,0,0,0,0,0,0,0,0,0,0,0,0,0,0,0,0,0,0,0,0,0,0,0,0,0,0,0,0,0,0,0,0,0,0,0,0,0,0,0,0,0,0,0,0,0,0,0,0,0,0,0,0,0,0,0,0,0,0,0,0,0,0,0,0,0,0,0,0,0,0,0,0,0,0,0,0,0,0,0,0,0,0,0,0,0,0,0,0,0,0,0,0,0,0,0,0,0,0,0,0,0,0,0,0,0,0,0,0,0,0,0,0,0,0,0,0,0,0,0,0,0,0,0,0,0,0,0,0,0,0,0,0,0,0,0,0,0,0,0,0,0,0,0,0,0,0,0,0,0,0,0,0,0,0,0,0,0,0,0,0,0,0,0,0,0,0,0,0,0,0,0,0,0,0,0,0,0,0,0,0,0,0,0,0,0,0,0,0,0,0,0,0,0,0,0,0,0,0,0,0,0,0,0,0,0,0,0,0,0,0,0,0,0,0,0,0,0,0,0,0,0,0,0,0,0,0,0,0,0,0,0,0,0,0,0,0,0,0,0,0,0,0,0,0,0,0,0,0,0,0,0,0,0,0,0,0,0,0,0,0,0,0,0,0,0,0,0,0,0,0,0,0,0,0,0,0,0,0,0,0,0,0,0,0,0,0,0,0,0,0,0,0,0,0,0,0,0,0,0,0,0,0,0,0,0,0,0,0,0,0,0,0,0,0,0,0,0,0,0,0,0,0,0,0,0,0,0,0,0,0,0,0,0,0,0,0,0,0,0,0,0,0,0,0,0,0,0,0,0,0,0,0,0,0,0,0,0,0,0,0,0,0,0,0,0,0,0,0,0,0,0,0,0,0,0,0,0,0,0,0,0,0,0,0,0,0,0,0,0,0,0,0,0,0,0,0,0,0,0,0,0,0,0,0,0,0,0,0,0,0,0,0,0,0,0,0,0,0,0,0,0,0,0,0,0,0,0,0,0,0,0,0,0,0,0,0,0,0,0,0,0,0,0,0,0,0,0,0,0,0,0,0,0,0,0,0,0,0,0,0,0,0,0,0,0,0,0,0,0,0,0,0,0,0,0,0,0,0,0,0,0,0,0,0,0,0,0,0,0,0,0,0,0,0,0,0,0,0,0,0,0,0,0,0,0,0,0,0,0,0,0,0,0,0,0,0,0,0,0,0,0,0,0,0,0,0,0,0,0,0,0,0,0,0,0,0,0,0,0,0,0,0,0,0,0,0,0,0,0,0,0,0,0,0,0,0,0,0,0,0,0,0,0,0,0,0,0,0,0,0,0,0,0,0,0,0,0,0,0,0,0,0,0,0,0,0,0,0,0,0,0,0,0,0,0,0,0,0,0,0,0,0,0,0,0,0,0,0,0,0,0,0,0,0,0,0,0,0,0,0,0,0,0,0,0,0,0,0,0,0,0,0,0,0,0,0,0,0,0,0,0,0,0,0,0,0,0,0,0,0,0,0,0,0,0,0,0,0,0,0,0,0,0,0,0,0,0,0,0,0,0,0,0,0,0,0,0,0,0,0,0,0,0,0,0,0,0,0,0,0,0,0,0,0,0,0,0,0,0,0,0,0,0,0,0,0,0,0,0,0,0,0,0,0,0,0,0,0,0,0,0,0,0,0,0,0,0,0,0,0,0,0,0,0,0,0,0,0,0,0,0,0,0,0,0,0,0,0,0,0,0,0,0,0,0,0,0,0,0,0,0,0,0]}

{"tickX":4}

{"coord\_y":[[16870,16870,16870,16684,16684,16131,16131,15552,15552,15029,15029,14955,14955,14538,14538,14501,14501,14210,14210,12773,12773,12462,12462,12093,12093,12041,12041,11690,11690,11572,11572,11041,11041,10876,10876,10638,10638,10499,10499,10369,10369,9721,9721,9717,9717,9552,9552,9456,9456,9360,9360,9188,9188,9142,9142,9012,9012,8816,8816,8630,8630,8608,8608,8601,8601,8558,8558,8478,8478,8442,8442,8356,8356,8246,8246,8198,8198,8138,8138,8017,8017,7944,7944,7908,7908,7896,7896,7895,7895,7794,7794,7769,7769,7689,7689,7632,7632,7628,7628,7483,7483,7464,7464,7409,7409,7360,7360,7264,7264,7233,7233,7214,7214,7158,7158,7154,7154,7085,7085,6839,6839,6836,6836,6801,6801,6790,6790,6770,6770,6767,6767,6758,6758,6704,6704,6668,6668,6650,6650,6648,6648,6639,6639,6633,6633,6559,6559,6547,6547,6502,6502,6489,6489,6483,6483,6445,6445,6415,6415,6399,6399,6337,6337,6300,6300,6262,6262,6216,6216,6182,6182,6141,6141,6119,6119,6096,6096,6056,6056,6048,6048,5981,5981,5977,5977,5967,5967,5964,5964,5941,5941,5912,5912,5908,5908,5861,5861,5841,5841,5783,5783,5760,5760,5754,5754,5741,5741,5738,5738,5686,5686,5684,5684,5683,5683,5665,5665,5610,5610,5603,5603,5571,5571,5538,5538,5523,5523,5508,5508,5501,5501,5496,5496,5487,5487,5470,5470,5466,5466,5426,5426,5420,5420,5405,5405,5365,5365,5332,5332,5320,5320,5253,5253,5230,5230,5218,5218,5214,5214,5201,5201,5196,5196,5190,5190,5184,5184,5180,5180,5150,5150,5141,5141,5095,5095,5085,5085,5059,5059,5046,5046,5043,5043,5041,5041,5026,5026,5022,5022,4989,4989,4988,4988,4935,4935,4904,4904,4891,4891,4874,4874,4830,4830,4828,4828,4820,4820,4818,4818,4809,4809,4786,4786,4779,4779,4768,4768,4758,4758,4754,4754,4744,4744,4680,4680,4662,4662,4643,4643,4633,4633,4592,4592,4590,4590,4589,4589,4586,4586,4558,4558,4557,4557,4526,4526,4520,4520,4513,4513,4511,4511,4506,4506,4488,4488,4473,4473,4461,4461,4452,4452,4430,4430,4427,4427,4406,4406,4404,4404,4392,4392,4387,4387,4381,4381,4380,4380,4353,4353,4342,4342,4336,4336,4327,4327,4317,4317,4299,4299,4297,4297,4291,4291,4275,4275,4272,4272,4253,4253,4236,4236,4186,4186,4179,4179,4178,4178,4157,4157,4154,4154,4130,4130,4119,4119,4118,4118,4108,4108,4105,4105,4104,4104,4095,4095,4087,4087,4082,4082,4080,4080,4067,4067,4038,4038,4013,4013,4012,4012,4005,4005,3995,3995,3969,3969,3938,3938,3915,3915,3906,3906,3890,3890,3889,3889,3882,3882,3854,3854,3852,3852,3844,3844,3840,3840,3826,3826,3803,3803,3774,3774,3773,3773,3766,3766,3761,3761,3741,3741,3738,3738,3730,3730,3723,3723,3683,3683,3680,3680,3677,3677,3650,3650,3638,3638,3634,3634,3597,3597,3588,3588,3571,3571,3557,3557,3553,3553,3534,3534,3527,3527,3516,3516,3513,3513,3481,3481,3471,3471,3462,3462,3453,3453,3420,3420,3415,3415,3386,3386,3385,3385,3373,3373,3364,3364,3353,3353,3336,3336,3316,3316,3304,3304,3298,3298,3292,3292,3288,3288,3287,3287,3282,3282,3274,3274,3272,3272,3265,3265,3236,3236,3208,3208,3205,3205,3193,3193,3192,3192,3183,3183,3182,3182,3180,3180,3179,3179,3166,3166,3162,3162,3144,3144,3139,3139,3138,3138,3135,3135,3133,3133,3084,3084,3074,3074,3068,3068,3041,3041,3031,3031,3003,3003,2990,2990,2987,2987,2975,2975,2959,2959,2941,2941,2938,2938,2937,2937,2932,2932,2931,2931,2930,2930,2929,2929,2923,2923,2909,2909,2908,2908,2906,2906,2901,2901,2900,2900,2899,2899,2896,2896,2893,2893,2877,2877,2876,2876,2870,2870,2859,2859,2850,2850,2848,2848,2839,2839,2832,2832,2830,2830,2828,2828,2821,2821,2804,2804,2803,2803,2799,2799,2796,2796,2794,2794,2783,2783,2779,2779,2776,2776,2761,2761,2746,2746,2726,2726,2717,2717,2700,2700,2691,2691,2689,2689,2684,2684,2676,2676,2670,2670,2664,2664,2657,2657,2652,2652,2645,2645,2619,2619,2618,2618,2615,2615,2612,2612,2601,2601,2599,2599,2592,2592,2585,2585,2583,2583,2572,2572,2570,2570,2567,2567,2566,2566,2551,2551,2543,2543,2515,2515,2489,2489,2486,2486,2482,2482,2480,2480,2477,2477,2463,2463,2449,2449,2445,2445,2444,2444,2433,2433,2431,2431,2430,2430,2423,2423,2413,2413,2409,2409,2407,2407,2402,2402,2401,2401,2373,2373,2351,2351,2345,2345,2342,2342,2340,2340,2336,2336,2335,2335,2332,2332,2326,2326,2320,2320,2313,2313,2307,2307,2301,2301,2300,2300,2293,2293,2287,2287,2286,2286,2283,2283,2281,2281,2277,2277,2271,2271,2270,2270,2260,2260,2253,2253,2248,2248,2240,2240,2233,2233,2229,2229,2222,2222,2221,2221,2218,2218,2210,2210,2199,2199,2181,2181,2169,2169,2168,2168,2157,2157,2153,2153,2151,2151,2142,2142,2141,2141,2139,2139,2137,2137,2131,2131,2127,2127,2115,2115,2108,2108,2105,2105,2102,2102,2095,2095,2074,2074,2068,2068,2063,2063,2062,2062,2061,2061,2057,2057,2053,2053,2047,2047,2026,2026,2025,2025,2023,2023,2022,2022,2021,2021,2020,2020,2014,2014,2008,2008,1998,1998,1997,1997,1996,1996,1988,1988,1986,1986,1982,1982,1978,1978,1974,1974,1954,1954,1953,1953,1950,1950,1949,1949,1942,1942,1918,1918,1914,1914,1913,1913,1910,1910,1908,1908,1907,1907,1906,1906,1892,1892,1890,1890,1888,1888,1883,1883,1881,1881,1878,1878,1877,1877,1864,1864,1859,1859,1856,1856,1851,1851,1848,1848,1841,1841,1837,1837,1830,1830,1826,1826,1809,1809,1806,1806,1797,1797,1783,1783,1782,1782,1777,1777,1772,1772,1766,1766,1755,1755,1753,1753,1747,1747,1743,1743,1739,1739,1737,1737,1734,1734,1731,1731,1728,1728,1727,1727,1726,1726,1723,1723,1720,1720,1717,1717,1712,1712,1711,1711,1709,1709,1708,1708,1707,1707,1706,1706,1705,1705,1694,1694,1681,1681,1680,1680,1677,1677,1675,1675,1672,1672,1670,1670,1667,1667,1663,1663,1659,1659,1657,1657,1653,1653,1649,1649,1647,1647,1642,1642,1640,1640,1638,1638,1637,1637,1634,1634,1630,1630,1626,1626,1625,1625,1621,1621,1619,1619,1614,1614,1608,1608,1604,1604,1603,1603,1601,1601,1590,1590,1589,1589,1577,1577,1574,1574,1567,1567,1563,1563,1560,1560,1558,1558,1552,1552,1549,1549,1548,1548,1535,1535,1528,1528,1511,1511,1506,1506,1502,1502,1500,1500,1496,1496,1495,1495,1491,1491,1490,1490,1488,1488,1486,1486,1483,1483,1477,1477,1467,1467,1466,1466,1455,1455,1454,1454,1453,1453,1447,1447,1446,1446,1442,1442,1439,1439,1435,1435,1426,1426,1424,1424,1419,1419,1416,1416,1414,1414,1408,1408,1406,1406,1403,1403,1402,1402,1391,1391,1386,1386,1379,1379,1372,1372,1371,1371,1368,1368,1362,1362,1361,1361,1359,1359,1354,1354,1350,1350,1348,1348,1344,1344,1343,1343,1340,1340,1336,1336,1334,1334,1327,1327,1326,1326,1321,1321,1319,1319,1310,1310,1302,1302,1301,1301,1299,1299,1296,1296,1295,1295,1292,1292,1291,1291,1288,1288,1286,1286,1280,1280,1276,1276,1272,1272,1269,1269,1266,1266,1258,1258,1254,1254,1249,1249,1246,1246,1241,1241,1233,1233,1232,1232,1228,1228,1224,1224,1223,1223,1220,1220,1219,1219,1218,1218,1215,1215,1207,1207,1205,1205,1203,1203,1194,1194,1193,1193,1184,1184,1183,1183,1182,1182,1178,1178,1176,1176,1170,1170,1169,1169,1165,1165,1164,1164,1155,1155,1128,1128,1126,1126,1121,1121,1118,1118,1117,1117,1116,1116,1113,1113,1107,1107,1098,1098,1096,1096,1093,1093,1092,1092,1090,1090,1089,1089,1087,1087,1082,1082,1073,1073,1067,1067,1066,1066,1063,1063,1058,1058,1056,1056,1048,1048,1042,1042,1039,1039,1035,1035,1033,1033,1030,1030,1021,1021,1019,1019,1015,1015,1014,1014,1013,1013,1009,1009,1008,1008,1005,1005,1000,1000,999,999,998,998,991,991,989,989,988,988,983,983,982,982,980,980,978,978,976,976,972,972,969,969,968,968,967,967,963,963,962,962,953,953,948,948,947,947,946,946,944,944,941,941,940,940,937,937,934,934,933,933,932,932,930,930,927,927,924,924,920,920,918,918,917,917,914,914,909,909,908,908,907,907,905,905,902,902,901,901,900,900,899,899,898,898,891,891,886,886,882,882,881,881,877,877,876,876,869,869,868,868,867,867,865,865,863,863,860,860,854,854,851,851,850,850,848,848,847,847,845,845,841,841,840,840,839,839,835,835,831,831,830,830,824,824,823,823,821,821,820,820,817,817,811,811,810,810,809,809,808,808,806,806,805,805,800,800,796,796,788,788,787,787,785,785,781,781,780,780,778,778,772,772,770,770,767,767,761,761,759,759,756,756,753,753,752,752,749,749,746,746,744,744,741,741,737,737,735,735,734,734,733,733,732,732,729,729,725,725,724,724,722,722,720,720,719,719,718,718,715,715,709,709,705,705,698,698,695,695,694,694,693,693,692,692,691,691,687,687,683,683,681,681,678,678,668,668,667,667,661,661,660,660,658,658,655,655,654,654,653,653,648,648,646,646,644,644,643,643,641,641,637,637,635,635,634,634,632,632,630,630,629,629,628,628,619,619,614,614,612,612,611,611,610,610,607,607,605,605,601,601,599,599,594,594,590,590,586,586,585,585,583,583,578,578,577,577,574,574,573,573,570,570,569,569,564,564,563,563,562,562,559,559,557,557,554,554,553,553,552,552,551,551,544,544,543,543,540,540,537,537,533,533,530,530,529,529,522,522,516,516,514,514,513,513,511,511,510,510,509,509,507,507,499,499,498,498,496,496,494,494,493,493,491,491,490,490,489,489,487,487,486,486,483,483,481,481,479,479,478,478,473,473,471,471,469,469,468,468,461,461,458,458,456,456,455,455,452,452,448,448,447,447,446,446,440,440,439,439,438,438,435,435,433,433,432,432,430,430,429,429,426,426,422,422,419,419,415,415,413,413,412,412,411,411,408,408,407,407,403,403,401,401,400,400,398,398,396,396,395,395,394,394,393,393,390,390,389,389,388,388,387,387,385,385,381,381,380,380,379,379,378,378,377,377,374,374,373,373,371,371,367,367,365,365,363,363,362,362,361,361,360,360,359,359,355,355,353,353,346,346,345,345,342,342,338,338,332,332,329,329,324,324,323,323,321,321,320,320,317,317,316,316,314,314,312,312,311,311,310,310,308,308,306,306,305,305,300,300,298,298,297,297,296,296,295,295,293,293,292,292,289,289,288,288,287,287,284,284,283,283,281,281,280,280,279,279,278,278,276,276,275,275,274,274,273,273,269,269,267,267,266,266,265,265,263,263,261,261,260,260,259,259,258,258,257,257,255,255,254,254,253,253,250,250,249,249,247,247,246,246,245,245,244,244,243,243,242,242,240,240,239,239,238,238,237,237,236,236,234,234,233,233,232,232,231,231,230,230,229,229,228,228,226,226,225,225,224,224,223,223,222,222,221,221,219,219,218,218,217,217,216,216,215,215,214,214,213,213,212,212,211,211,210,210,209,209,208,208,207,207,206,206,205,205,204,204,203,203,202,202,201,201,200,200,199,199,198,198,197,197,196,196,195,195,194,194,193,193,192,192,191,191,190,190,189,189,188,188,186,186,185,185,184,184,183,183,182,182,181,181,180,180,179,179,178,178,177,177,176,176,175,175,174,174,173,173,172,172,171,171,170,170,169,169,168,168,167,167,166,166,165,165,164,164,163,163,162,162,161,161,160,160,159,159,158,158,157,157,156,156,155,155,154,154,153,153,152,152,151,151,150,150,149,149,148,148,147,147,146,146,145,145,144,144,143,143,142,142,141,141,140,140,139,139,138,138,137,137,136,136,135,135,134,134,133,133,132,132,131,131,130,130,129,129,128,128,127,127,126,126,125,125,124,124,123,123,122,122,121,121,120,120,119,119,118,118,117,117,116,116,115,115,114,114,113,113,112,112,111,111,110,110,109,109,108,108,107,107,106,106,105,105,104,104,103,103,102,102,101,101,0.0]],"coord\_x":[[0.0,1e-10,0.48925566145944355,0.48925566155944356,0.9731170399887474,0.9731170400887474,1.440940577333281,1.440940577433281,1.8919722339436704,1.8919722340436704,2.327836095003183,2.327836095103183,2.7615538434990916,2.7615538435990916,3.1831779576838803,3.1831779577838804,3.6037290155868673,3.6037290156868673,4.015840630841088,4.015840630941088,4.3862770602318095,4.3862770603318095,4.7476940165511685,4.7476940166511685,5.098409411573637,5.098409411673637,5.4476167274973575,5.4476167275973575,5.786644509504524,5.786644509604524,6.122250112018375,6.122250112118375,6.442455906812312,6.442455906912312,6.757876450619835,6.757876450719835,7.066394632398469,7.066394632498469,7.370881602740064,7.370881602840064,7.671598375334787,7.671598375434787,7.95352216223741,7.95352216233741,8.235329943055513,8.235329943155513,8.512352472887203,8.512352472987203,8.786590856690433,8.786590856790433,9.058045094465205,9.058045094565205,9.324511070605654,9.324511070705654,9.589642976774131,9.589642976874131,9.851004685195738,9.851004685295738,10.106682095475907,10.106682095575907,10.356965222825938,10.356965222925938,10.606610316711112,10.606610316811112,10.856052399948377,10.856052400048377,11.104247417777062,11.104247417877062,11.350122313915366,11.350122314015366,11.594953155292995,11.594953155392995,11.837289865853464,11.837289865953464,12.076436409089657,12.076436409189657,12.314190879311619,12.314190879411619,12.550205258265796,12.550205258365796,12.782710453163268,12.782710453263268,13.013098537018267,13.013098537118267,13.242442566112592,13.242442566212592,13.47143857695336,13.47143857705336,13.700405586273,13.700405586373,13.926443441958531,13.926443442058531,14.151756259615818,14.151756259715818,14.37474895558272,14.37474895568272,14.596088564845227,14.596088564945227,14.817312168023212,14.817312168123213,15.034330550637382,15.034330550737382,15.250797904350083,15.250797904450083,15.465670174400648,15.465670174500648,15.679121369915853,15.679121370015853,15.889788419402597,15.889788419502597,16.09955642173432,16.09955642183432,16.308773395164575,16.308773395264573,16.516366283411564,16.516366283511562,16.723843165574035,16.723843165674033,16.929318942778547,16.929318942878545,17.127660345785138,17.127660345885136,17.325914744228335,17.325914744328333,17.52315408943199,17.52315408953199,17.72007441790322,17.720074418003218,17.916414715951852,17.91641471605185,18.112668009437094,18.112668009537092,18.30866028923217,18.308660289332167,18.503086486886236,18.503086486986234,18.69646862977963,18.69646862987963,18.88932874529269,18.88932874539269,19.082130857763488,19.082130857863486,19.274671956544122,19.27467195664412,19.46703904619797,19.46703904629797,19.65726002328822,19.65726002338822,19.84713298212491,19.84713298222491,20.035700872510763,20.03570087261076,20.412459633507776,20.412459633607774,20.60047649499216,20.60047649509216,20.787391298673615,20.787391298773613,20.973436056721177,20.973436056821175,21.15901679043066,21.159016790530657,21.342799429830094,21.342799429930093,21.52550901294773,21.525509013047728,21.707116538262433,21.70711653836243,21.887389993605165,21.887389993705163,22.066677397229483,22.06667739732948,22.244775738487483,22.24477573858748,22.422236046280627,22.422236046380625,22.599029319087787,22.599029319187785,22.774662531049753,22.77466253114975,22.950063730842682,22.95006373094268,23.123521828719916,23.123521828819914,23.29686392051263,23.29686392061263,23.46991599709405,23.469915997194047,23.642881069112075,23.642881069212073,23.988144178162145,23.988144178262143,24.159601171081423,24.15960117118142,24.330942157916184,24.33094215801618,24.50092007325784,24.50092007335784,24.670317958176906,24.670317958276904,24.838033754870445,24.838033754970443,25.005082516577996,25.005082516677994,25.171957269158767,25.171957269258765,25.338455001964853,25.33845500206485,25.50486573020755,25.504865730307547,25.669768379351495,25.669768379451494,25.834613025453184,25.834613025553182,25.999428670033744,25.999428670133742,26.163722287233966,26.163722287333965,26.32642082077205,26.32642082087205,26.488916343662225,26.488916343762224,26.650483817876246,26.650483817976244,26.811094241892988,26.811094241992986,26.97126964309278,26.971269643192777,27.131010021475625,27.131010021575623,27.290547389210563,27.29054738931056,27.44993974933985,27.44993974943985,27.609071095778972,27.60907109587897,27.767709416358887,27.767709416458885,27.926231730854283,27.92623173095428,28.083593984504486,28.083593984604484,28.240782229027914,28.240782229127912,28.39753545073439,28.39753545083439,28.553128611595678,28.553128611695676,28.707764722259682,28.70776472235968,28.86205281467013,28.862052814770127,29.01439780516488,29.014397805264878,29.166075760673646,29.166075760773644,29.317405697928855,29.317405698028853,29.468619629099546,29.468619629199544,29.61945654049555,29.619456540595547,29.7701484442859,29.7701484443859,29.920666338949477,29.920666339049475,30.071010224486276,30.071010224586274,30.22123810393855,30.221238104038548,30.370595937756935,30.370595937856933,30.51969275788515,30.519692757985148,30.816552328169614,30.816552328269612,30.964025063114562,30.96402506321456,31.110743758510132,31.11074375861013,31.25708543413102,31.25708543423102,31.403340105188516,31.403340105288514,31.549536773203755,31.549536773303753,31.695298418402047,31.695298418502045,31.840944057515816,31.840944057615815,31.985632646432304,31.985632646532302,32.13029223382767,32.13029223392767,32.273414740603144,32.273414740703146,32.415638200223604,32.415638200323606,32.55748464006937,32.55748464016937,32.69883805405593,32.698838054155935,32.838915401112786,32.83891540121279,32.97893474512738,32.978934745227384,33.11872207697294,33.11872207707294,33.258451405776235,33.25845140587624,33.39791972088936,33.397919720989364,33.5367210010165,33.536721001116504,33.675319270495734,33.675319270595736,33.81359852324255,33.81359852334255,33.951587760778054,33.951587760878056,34.22745022976455,34.22745022986455,34.36503344600424,34.36503344610424,34.50076056489163,34.50076056499163,34.635965656398675,34.635965656498676,34.77061971900426,34.77061971910426,34.904983766398544,34.904983766498546,35.038158751426515,35.03815875152652,35.171275733412216,35.17127573351222,35.304363713876796,35.3043637139768,35.43736468977798,35.43736468987798,35.56955362308753,35.56955362318753,35.70171355487595,35.70171355497595,35.83297443950935,35.83297443960935,35.964061315015975,35.96406131511598,36.094945179874685,36.09494517997469,36.22577104169114,36.22577104179114,36.35645189590194,36.35645189600194,36.48661072273241,36.48661072283241,36.61633452674593,36.61633452684593,36.74571031250589,36.745710312605894,36.87482508457568,36.874825084675685,37.00330182318063,37.00330182328063,37.131691557222176,37.13169155732218,37.25947225932,37.25947225942,37.38719495837557,37.38719495847557,37.51456963917757,37.514569639277575,37.641799312373934,37.641799312473935,37.768854976443514,37.768854976543516,37.895881638991966,37.89588163909197,38.02212526046991,38.022125260569915,38.148049865215434,38.148049865315436,38.273800460834174,38.273800460934176,38.39929004276274,38.399290042862745,38.524489609480014,38.524489609580016,38.64916714881696,38.64916714891696,38.77378668511163,38.77378668521163,38.89823221227953,38.89823221237953,39.02221371510936,39.02221371520936,39.14610821337579,39.14610821347579,39.26945168274076,39.26945168284076,39.39230212624652,39.39230212634652,39.513702493695796,39.5137024937958,39.75630021794643,39.756300218046434,39.877468573226665,39.87746857332667,39.99802789656317,39.998027896663174,40.1185002153363,40.1185002154363,40.2382764976023,40.2382764977023,40.357733763135876,40.35773376323588,40.477162027148324,40.477162027248326,40.71572853996192,40.715728540061924,40.83477978419968,40.834779784299684,41.072853271154074,41.072853271254075,41.191614500180535,41.19161450028054,41.31014371703796,41.31014371713796,41.42852792628973,41.428527926389734,41.54685413249925,41.54685413259925,41.66480331893408,41.66480331903408,41.78191146125614,41.781911461356145,41.89829456554996,41.89829456564996,42.01464866832266,42.01464866842266,42.13079976044744,42.13079976054744,42.24666083736092,42.24666083746092,42.36176787472503,42.361767874825034,42.475975864934114,42.475975865034115,42.589516820157215,42.58951682025722,42.70279676169015,42.702796761790154,42.81561267888501,42.81561267898501,42.928399594558734,42.928399594658735,43.26655733093201,43.26655733103201,43.37832919336619,43.37832919346619,43.49004305275812,43.49004305285812,43.601524899981,43.601524900081,43.712890741119374,43.712890741219375,43.82385056096192,43.823850561061924,43.93414334581849,43.93414334591849,44.04359508656229,44.04359508666229,44.153017825784964,44.153017825884966,44.3716602935824,44.3716602936824,44.48073501455151,44.480735014651515,44.589229705098035,44.589229705198036,44.69763739108116,44.69763739118116,44.805813064895254,44.805813064995256,44.913785728061434,44.913785728161436,45.02059833038243,45.02059833048243,45.12732392814003,45.12732392824003,45.23396252133424,45.23396252143424,45.339818073457955,45.33981807355796,45.4453256073281,45.4453256074281,45.550717135113736,45.55071713521374,45.65503560661757,45.65503560671757,45.75909306443123,45.75909306453123,45.86265749638569,45.86265749648569,45.96581590704432,45.965815907144325,46.06885831161844,46.068858311718444,46.1713496872911,46.1713496873911,46.273638052315846,46.27363805241585,46.37560740060816,46.37560740070816,46.47748974433709,46.477489744437094,46.578444039389865,46.57844403948987,46.78006261428412,46.78006261438412,46.88046588043543,46.88046588053543,46.98060813289657,46.98060813299657,47.07979333516043,47.07979333526043,47.17883352981864,47.17883352991864,47.27703268036409,47.27703268046409,47.3752028293884,47.3752028294884,47.47302496015916,47.47302496025916,47.57058607723975,47.570586077339755,47.667828177587914,47.667828177687916,47.86181935242504,47.86181935252504,47.957988396491395,47.9579883965914,48.0538094223042,48.0538094224042,48.14945643899023,48.14945643909023,48.24492944654947,48.24492944664947,48.3402864480242,48.3402864481242,48.4356144479778,48.4356144480778,48.53079744032574,48.530797440425744,48.62574842050466,48.62574842060466,48.72064139764131,48.72064139774131,48.815331364130046,48.81533136423005,48.90918028650603,48.90918028660603,49.00221716629037,49.00221716639037,49.095167041511324,49.095167041611326,49.18776889847872,49.187768898578724,49.37294361089239,49.372943610992394,49.46525545264849,49.465255452748494,49.557538292883464,49.557538292983466,49.649763130076174,49.649763130176176,49.741958965747756,49.74195896584776,49.83377778164465,49.83377778174465,49.925480591457024,49.925480591557026,50.01666137388906,50.01666137398906,50.10769714871545,50.107697148815454,50.19870392202071,50.198703922120714,50.28962369076258,50.289623690862584,50.38048545646219,50.38048545656219,50.469926147626445,50.46992614772645,50.559076823579396,50.5590768236794,50.64805349040557,50.648053490505575,50.736247116161245,50.73624711626125,50.82415072670562,50.82415072680562,50.91124229465836,50.91124229475836,50.99795684283641,50.99795684293641,51.08458438645107,51.08458438655107,51.17086391181218,51.17086391191218,51.256679412835204,51.256679412935206,51.3419728864779,51.3419728865779,51.4271793555572,51.4271793556572,51.512356823115375,51.51235682321538,51.5973892830679,51.5973892831679,51.68239274149929,51.682392741599294,51.76736719840956,51.76736719850956,51.85231265379869,51.85231265389869,51.93708410006105,51.93708410016105,52.021449525027585,52.021449525127586,52.105785948473,52.105785948573,52.19006436887615,52.19006436897615,52.27419778167365,52.27419778177365,52.44243560574752,52.44243560584752,52.52651101550276,52.526511015602765,52.610499420694616,52.61049942079462,52.69440082132308,52.69440082142308,52.77783819761346,52.777838197713464,52.86124657238272,52.86124657248272,52.9444809380252,52.9444809381252,53.02739628693525,53.02739628703525,53.11005062215513,53.11005062225513,53.19264695433275,53.19264695443275,53.27498227282021,53.27498227292021,53.357114580659754,53.357114580759756,53.43918888545704,53.439188885557044,53.52120518721207,53.52120518731207,53.603018478319186,53.60301847841919,53.6843387435671,53.684338743667105,53.76563000729388,53.76563000739388,53.846805264936144,53.846805265036146,53.92789351801502,53.92789351811502,54.00892376805164,54.00892376815164,54.08963500135582,54.089635001455825,54.17023022857549,54.17023022867549,54.25073845123177,54.25073845133177,54.3308116510711,54.3308116511711,54.41044982809348,54.410449828193485,54.489507974693275,54.48950797479328,54.568305107602896,54.5683051077029,54.64660921465331,54.64660921475331,54.72465230801355,54.724652308113555,54.80263739833154,54.80263739843154,54.88047748104388,54.880477481143885,54.95808555158718,54.958085551687184,55.0355196130037,55.0355196131037,55.112779665293445,55.11277966539345,55.26709675922502,55.26709675932502,55.3440087932612,55.344008793361205,55.420717816649486,55.42071781674949,55.57338182387666,55.57338182397666,55.649307806194436,55.64930780629444,55.725146783948816,55.72514678404882,55.80089875713981,55.800898757239814,55.876331713598375,55.87633171369838,55.95170666701468,55.951706667114685,56.02687860978308,56.026878609883084,56.101847541903574,56.101847542003576,56.1767584709818,56.176758471081804,56.251350383327605,56.25135038342761,56.32588429263115,56.32588429273115,56.4003311973713,56.4003311974713,56.47474910059032,56.474749100690325,56.5487319809924,56.5487319810924,56.62248284922544,56.62248284932544,56.69542167486685,56.69542167496685,56.76760646095888,56.76760646105888,56.83970424248752,56.83970424258752,56.91168601793164,56.911686018031645,56.9836097903335,56.983609790433505,57.05544655817198,57.05544655827198,57.12687730471463,57.126877304814634,57.19790202996147,57.19790203006147,57.268810749123794,57.268810749223796,57.4105991859273,57.410599186027305,57.55203960447726,57.55203960457726,57.62254230234376,57.62254230244376,57.693015998689134,57.693015998789136,57.83376038073197,57.83376038083197,57.903741051218134,57.903741051318136,57.97360571561978,57.973605715719785,58.04341237697917,58.04341237707917,58.11307403073291,58.11307403083291,58.182706682965524,58.182706683065526,58.2515272926065,58.2515272927065,58.31970986878262,58.31970986888262,58.38771843583196,58.38771843593196,58.45563999831791,58.455639998417915,58.52350355776161,58.52350355786161,58.591251111120776,58.59125111122078,58.658969662958825,58.658969663058826,58.726601210233476,58.72660121033348,58.79405874838135,58.794058748481355,58.86134227740245,58.86134227750245,58.92842279577564,58.92842279587564,58.99532930502205,58.99532930512205,59.26278133288091,59.262781332980914,59.32948483147941,59.329484831579414,59.395985319430004,59.395985319530006,59.46231179825382,59.462311798353824,59.528609275556505,59.52860927565651,59.5948197482958,59.5948197483958,59.660972217992835,59.66097221809284,59.72700868160535,59.72700868170535,59.79287113609109,59.79287113619109,59.8587045890557,59.8587045891557,59.924248026809,59.924248026909005,59.98958845391441,59.98958845401441,60.05478387341416,60.05478387351416,60.119747280744875,60.11974728084488,60.2494710847584,60.2494710848584,60.31411547535668,60.31411547545668,60.37855685530706,60.37855685540706,60.44296923373631,60.44296923383631,60.50729460760217,60.50729460770217,60.57138796929899,60.57138796939899,60.63516231426338,60.635162314363384,60.69841463184744,60.69841463194744,60.76131893117794,60.76131893127794,60.82419422898731,60.82419422908731,60.88675051006425,60.886750510164255,60.94919078505668,60.94919078515668,61.01157305700684,61.011573057106844,61.073694315266835,61.073694315366836,61.1357865720057,61.1357865721057,61.19782082570231,61.19782082580231,61.259797076356655,61.25979707645666,61.32159931788422,61.32159931798422,61.38328555332727,61.38328555342727,61.44462377051676,61.444623770616765,61.50575897705835,61.50575897715835,61.56680717903654,61.56680717913654,61.62776837645134,61.627768376551344,61.68852656321824,61.68852656331824,61.74867571804141,61.74867571814141,61.8086508637378,61.808650863837805,61.86848100182855,61.86848100192855,61.92828213839816,61.92828213849816,61.98805427344664,61.988054273546645,62.04771040241061,62.04771040251061,62.10725052529005,62.10725052539005,62.166616639042715,62.16661663914272,62.28473983460432,62.284739834704325,62.34346791489214,62.34346791499214,62.40213799213769,62.40213799223769,62.46077906786211,62.460779067962115,62.5193911420654,62.519391142165404,62.63658628895085,62.636586289050854,62.69499535250624,62.69499535260624,62.75323040693485,62.75323040703485,62.81117544615215,62.81117544625215,62.86909148384833,62.86909148394833,62.98489455771955,62.98489455781955,63.04254958172556,63.04254958182556,63.100146602689314,63.100146602789316,63.157627617568544,63.157627617668545,63.214992626363255,63.21499262646326,63.32960663786815,63.329606637968155,63.44352461286594,63.443524612965945,63.50016458363241,63.50016458373241,63.55671754983549,63.55671754993549,63.61324151451743,63.613241514617435,63.72608643323343,63.72608643333343,63.78171135076035,63.78171135086035,63.837220262202756,63.83722026230276,63.89270017212403,63.89270017222403,63.948093077481914,63.948093077581916,64.00342797979754,64.00342797989754,64.11406878290767,64.11406878300767,64.16934568218103,64.16934568228103,64.22421656015858,64.22421656025858,64.33390031307142,64.33390031317143,64.38865518496445,64.38865518506445,64.44326504925183,64.44326504935184,64.55242677478434,64.55242677488434,64.60689163146607,64.60689163156607,64.66132748662667,64.66132748672668,64.71538632201259,64.7153863221126,64.76930014979285,64.76930014989286,64.82312697300974,64.82312697310974,64.87680878862096,64.87680878872096,64.93040359966881,64.93040359976881,64.98379540006873,64.98379540016873,65.03707119438414,65.03707119448414,65.09014397805164,65.09014397815164,65.14310075563463,65.14310075573464,65.19556450735841,65.19556450745841,65.24794125451879,65.2479412546188,65.30005698798902,65.30005698808903,65.35176670016342,65.35176670026343,65.4034474108167,65.4034474109167,65.45498311386432,65.45498311396432,65.5063738093063,65.5063738094063,65.60898119106348,65.60898119116348,65.71109554696143,65.71109554706143,65.76193521350194,65.76193521360194,65.81260087091567,65.81260087101568,65.8631505222449,65.8631505223449,65.91358416748959,65.91358416758959,65.96395980969201,65.96395980979202,66.01424844733106,66.01424844743106,66.11473871804576,66.11473871814576,66.16485334655803,66.16485334665803,66.26505360206143,66.26505360216143,66.31511022753143,66.31511022763144,66.36507984843806,66.36507984853806,66.41496246478128,66.41496246488128,66.46475807656111,66.46475807666111,66.5144086807353,66.5144086808353,66.56403028338836,66.56403028348836,66.61359388299917,66.61359388309917,66.66312848108883,66.66312848118883,66.71263407765737,66.71263407775737,66.76211067270478,66.76211067280478,66.81155826623106,66.81155826633106,66.86068684302492,66.86068684312492,66.90943840004408,66.90943840014408,66.95816095554211,66.95816095564211,67.0555190619748,67.05551906207481,67.10409660986718,67.10409660996719,67.20116470108857,67.20116470118857,67.24959724137531,67.24959724147531,67.29794277709865,67.29794277719866,67.34617230673749,67.34617230683749,67.39428583029179,67.3942858303918,67.44234135080384,67.44234135090385,67.49028086523138,67.49028086533139,67.5381043735744,67.5381043736744,67.58586987887514,67.58586987897515,67.63349037657025,67.63349037667025,67.6810528712231,67.6810528713231,67.72855736283368,67.72855736293369,67.77603285292314,67.77603285302314,67.87089682853866,67.87089682863866,67.91816930798021,67.91816930808021,67.96532578133723,67.96532578143723,68.01245325317313,68.01245325327314,68.0594647189245,68.0594647190245,68.10641818163363,68.10641818173363,68.1532266367371,68.1532266368371,68.1998610827138,68.1998610828138,68.24637952260596,68.24637952270596,68.292868960977,68.292868961077,68.38578983467683,68.38578983477683,68.43190225327318,68.43190225337318,68.4779856703484,68.4779856704484,68.52372106917008,68.52372106927008,68.56936946342836,68.56936946352836,68.61481484703873,68.61481484713873,68.66014422456458,68.66014422466458,68.70538659752704,68.70538659762704,68.79581334040971,68.79581334050971,68.84082370120313,68.84082370130314,68.88574705743316,68.88574705753317,68.93064141214207,68.93064141224207,68.97515874707628,68.97515874717628,69.06399040629681,69.06399040639681,69.10781170472391,69.10781170482392,69.15148799554537,69.15148799564537,69.19504828028231,69.19504828038231,69.23855056197698,69.23855056207698,69.28193683758712,69.28193683768713,69.32529411167616,69.32529411177616,69.36853537968067,69.36853537978067,69.41174764616405,69.41174764626405,69.49811417608854,69.49811417618854,69.54121043648739,69.54121043658739,69.62731595272172,69.62731595282172,69.6701511994304,69.67015119953041,69.7555316776365,69.7555316777365,69.79804790761276,69.79804790771276,69.8402451208566,69.8402451209566,69.92461054582313,69.92461054592313,69.9667497560247,69.9667497561247,70.0087149570995,70.0087149571995,70.05065115665316,70.05065115675316,70.09247135012231,70.09247135022231,70.13420453902808,70.13420453912808,70.17582172184932,70.17582172194932,70.21717789098038,70.21717789108038,70.25847605706919,70.25847605716919,70.29962921555236,70.29962921565236,70.38184852795528,70.38184852805529,70.4228566788328,70.4228566789328,70.46369082058354,70.46369082068354,70.50446695929202,70.50446695939202,70.5451560934371,70.5451560935371,70.58581622606106,70.58581622616106,70.62615734195259,70.6261573420526,70.66635345023846,70.66635345033846,70.70634654787644,70.70634654797644,70.7461366348665,70.7461366349665,70.78589772033543,70.78589772043543,70.82557180124097,70.82557180134097,70.86507187301974,70.86507187311975,70.90454294327738,70.90454294337738,70.94395601049276,70.94395601059276,70.98322407010248,70.98322407020248,71.02237612362768,71.02237612372768,71.06147017411064,71.06147017421064,71.10044821850906,71.10044821860906,71.17837530578478,71.17837530588479,71.21723734409869,71.2172373441987,71.25598337632809,71.25598337642809,71.29467140551522,71.29467140561522,71.37184445324158,71.37184445334158,71.48727050733811,71.48727050743811,71.52558151675055,71.52558151685055,71.60214553253319,71.60214553263319,71.64013752521319,71.6401375253132,71.7158894984042,71.7158894985042,71.75362047739404,71.75362047749404,71.79129345334162,71.79129345344163,71.82887942472583,71.82887942482583,71.8664363945889,71.8664363946889,71.90390635988858,71.90390635998858,71.97881728896681,71.97881728906681,72.05361221196051,72.05361221206051,72.09090816813342,72.09090816823343,72.12803011517954,72.12803011527954,72.16503605614115,72.16503605624115,72.20192599101823,72.20192599111823,72.23872892133193,72.23872892143193,72.27544484708223,72.27544484718223,72.31192876066349,72.3119287607635,72.34829666816024,72.34829666826025,72.38451956805135,72.38451956815135,72.42065546337905,72.42065546347905,72.45664635110111,72.45664635120112,72.49240522665414,72.49240522675414,72.52813510068603,72.52813510078603,72.5637489686334,72.5637489687334,72.59924683049626,72.59924683059626,72.63471569083798,72.63471569093798,72.67009754661632,72.67009754671632,72.70545040087353,72.70545040097353,72.81147996212401,72.81147996222401,72.8467168102967,72.8467168103967,72.88172164630035,72.88172164640035,72.91666847926173,72.91666847936173,72.95155730918087,72.95155730928087,73.02107395532896,73.02107395542896,73.0556727700368,73.0556727701368,73.09001057105445,73.09001057115445,73.12431937055099,73.124319370651,73.1585991685264,73.1585991686264,73.22704275839268,73.22704275849269,73.2611485472413,73.2611485473413,73.29508032696315,73.29508032706315,73.32898310516387,73.32898310526387,73.39667265548078,73.39667265558079,73.43043042607584,73.43043042617585,73.46392718298075,73.46392718308076,73.49664089881514,73.49664089891515,73.52929661160728,73.52929661170728,73.5944630295859,73.5944630296859,73.626886730209,73.626886730309,73.65928142931097,73.65928142941097,73.69164712689181,73.69164712699181,73.72392581990925,73.72392582000926,73.78830919681738,73.78830919691738,73.85225755090855,73.85225755100855,73.8840432180668,73.8840432181668,73.91574188066164,73.91574188076164,73.94741154173536,73.94741154183536,74.01069286084055,74.01069286094055,74.04227551735089,74.04227551745089,74.07380017081896,74.07380017091896,74.10517981668139,74.10517981678139,74.13629844885364,74.13629844895364,74.16724307189912,74.16724307199912,74.19815869342347,74.19815869352347,74.22898731038443,74.22898731048443,74.2904995367007,74.2904995368007,74.32112514301375,74.32112514311375,74.38214434347081,74.38214434357081,74.44275752263206,74.44275752273207,74.4728901030859,74.4728901031859,74.53303925790908,74.53303925800908,74.59301440360547,74.59301440370547,74.62288597036914,74.62288597046914,74.65249652344265,74.65249652354265,74.6820490734739,74.6820490735739,74.71148561742064,74.71148561752064,74.74089315984624,74.74089315994624,74.7996792431763,74.7996792432763,74.82894177799625,74.82894177809625,74.85817531129507,74.85817531139507,74.88732184003051,74.88732184013051,74.9163233611603,74.9163233612603,74.94529588076895,74.94529588086895,74.97423939885647,74.97423939895647,75.00297990629609,75.00297990639609,75.03166241069344,75.03166241079344,75.06031591356967,75.06031591366967,75.08882440884024,75.08882440894024,75.14581239786027,75.14581239796027,75.17423388856746,75.17423388866746,75.20259737623239,75.20259737633239,75.23090286085505,75.23090286095506,75.2590923393932,75.25909233949321,75.28719481336796,75.28719481346796,75.31526828582159,75.31526828592159,75.34331275675409,75.34331275685409,75.37124122160208,75.37124122170208,75.39914068492892,75.39914068502893,75.4267791345656,75.42677913466561,75.45427257659664,75.45427257669664,75.48173701710655,75.48173701720656,75.53663689660523,75.53663689670523,75.59144977154051,75.59144977164051,75.61874020292365,75.61874020302365,75.64600163278564,75.64600163288564,75.70043748794625,75.70043748804625,75.72752490868146,75.72752490878146,75.75458332789555,75.75458332799555,75.7816127455885,75.7816127456885,75.8085841602392,75.80858416033921,75.83546857032651,75.83546857042651,75.86226597585043,75.86226597595044,75.88894737528983,75.88894737538983,75.91557077168697,75.91557077178697,75.94216516656299,75.94216516666299,75.9686725568756,75.9686725569756,76.0215423298952,76.0215423299952,76.04787571108105,76.04787571118105,76.07418009074576,76.07418009084576,76.10042646736821,76.10042646746821,76.12658583942728,76.12658583952728,76.1527162099652,76.15271621006521,76.17881757898202,76.17881757908202,76.20488994647769,76.2048899465777,76.23093331245224,76.23093331255224,76.25677366777887,76.25677366787887,76.28246901549986,76.28246901559986,76.30804835713633,76.30804835723633,76.33359869725167,76.33359869735168,76.3590330312825,76.3590330313825,76.38443836379219,76.38443836389219,76.40964068565397,76.40964068575397,76.43481400599461,76.43481400609461,76.4851316451548,76.4851316452548,76.53536227975157,76.53536227985157,76.56039059248657,76.56039059258657,76.58533190065819,76.58533190075819,76.61009919970303,76.61009919980303,76.63477949418447,76.63477949428447,76.65943078714479,76.65943078724479,76.70867537002316,76.70867537012316,76.78242623825619,76.7824262383562,76.80693252361087,76.80693252371087,76.831322802881,76.831322802981,76.85568408063003,76.85568408073003,76.88001635685792,76.88001635695792,76.90423262700129,76.9042326271013,76.92833289106014,76.92833289116014,76.95240415359785,76.95240415369786,76.97630140700879,76.97630140710879,77.00016965889861,77.00016965899862,77.02397990774617,77.02397990784617,77.04776115507258,77.04776115517258,77.07145539783562,77.07145539793562,77.09497563147187,77.09497563157187,77.11846686358699,77.11846686368699,77.141929094181,77.141929094281,77.16536232325386,77.16536232335386,77.21217077835732,77.21217077845732,77.2355170028668,77.2355170029668,77.2820644442801,77.2820644443801,77.32835087200324,77.32835087210324,77.35120407065351,77.35120407075351,77.37402826778265,77.37402826788265,77.39679446186952,77.39679446196952,77.41944464987189,77.4194446499719,77.48736621235784,77.48736621245784,77.5099293957968,77.5099293958968,77.532318570109,77.532318570209,77.55464974137894,77.55464974147894,77.57689390808548,77.57689390818548,77.59896406566524,77.59896406576524,77.62097622020275,77.62097622030275,77.64290137017687,77.64290137027687,77.6647395155876,77.6647395156876,77.6865486594772,77.6865486595772,77.7082707988034,77.7082707989034,77.72990593356622,77.72990593366622,77.75148306528678,77.75148306538678,77.77297319244394,77.77297319254394,77.7943473135166,77.7943473136166,77.81566343154698,77.81566343164698,77.83695054805625,77.83695054815625,77.85820866304438,77.85820866314438,77.90069589149951,77.90069589159951,77.92183800040313,77.92183800050313,77.96400621212582,77.96400621222583,77.98500331342379,77.98500331352379,78.0059424116795,78.0059424117795,78.02682350689294,78.02682350699294,78.04767560058525,78.04767560068525,78.06849869275644,78.06849869285644,78.08923478036424,78.08923478046424,78.10979685884524,78.10979685894524,78.13024293124174,78.13024293134174,78.15048599299033,78.15048599309033,78.17064205017553,78.17064205027553,78.1907691058396,78.19076910593961,78.21086715998254,78.21086716008254,78.23093621260435,78.23093621270435,78.25097626370503,78.25097626380503,78.2709003087212,78.2709003088212,78.31063239266899,78.310632392769,78.33038242855838,78.33038242865838,78.36979549577376,78.36979549587376,78.38916851188844,78.38916851198844,78.40851252648201,78.40851252658202,78.4276825319488,78.4276825320488,78.44682353589445,78.44682353599445,78.46590653679786,78.46590653689786,78.48490253313787,78.48490253323787,78.50386952795674,78.50386952805674,78.52280752125449,78.5228075213545,78.5416005069466,78.5416005070466,78.56033548959643,78.56033548969643,78.59774745185385,78.59774745195385,78.65374938915546,78.65374938925547,78.67233936419966,78.67233936429966,78.72799328324771,78.72799328334771,78.74640924916513,78.74640924926513,78.7647962135614,78.7647962136614,78.78312517491543,78.78312517501543,78.80139613322719,78.80139613332719,78.81963809001782,78.81963809011782,78.83785104528732,78.83785104538732,78.85580298686666,78.85580298696667,78.87360992084035,78.87360992094035,78.89135885177177,78.89135885187177,78.92682771211351,78.92682771221351,78.94451864000267,78.94451864010267,78.96212256332845,78.96212256342845,78.97966848361196,78.97966848371196,78.99709839781096,78.99709839791096,79.0319002231667,79.0319002232667,79.04912712671779,79.0491271268178,79.06623802418437,79.06623802428437,79.08323291556643,79.08323291566643,79.1171936968094,79.1171936969094,79.13410158362807,79.13410158372807,79.15086446284107,79.15086446294107,79.16759834053296,79.16759834063296,79.20097909135335,79.20097909145335,79.21759696296071,79.21759696306071,79.23412783000468,79.23412783010468,79.25062969552754,79.25062969562754,79.26698655344472,79.26698655354473,79.2833144098408,79.2833144099408,79.29961326471573,79.29961326481573,79.33212396990223,79.33212397000223,79.3482778171715,79.34827781727151,79.36434465987742,79.36434465997742,79.38038250106219,79.38038250116219,79.39639134072583,79.39639134082583,79.41237117886833,79.41237117896833,79.42814800636293,79.42814800646293,79.44389583233641,79.44389583243641,79.45955665374649,79.45955665384649,79.47513047059319,79.47513047069319,79.49058828135536,79.49058828145536,79.52141689831632,79.52141689841632,79.53675870299398,79.53675870309398,79.55189749702372,79.55189749712372,79.56686228192669,79.56686228202669,79.61169863359333,79.61169863369334,79.64148319579363,79.64148319589363,79.67118075343052,79.67118075353052,79.68597152920671,79.68597152930671,79.70073330346176,79.70073330356176,79.71543707467457,79.71543707477457,79.72990883371833,79.72990883381833,79.74435159124096,79.74435159134096,79.75873634572133,79.75873634582133,79.78744785163983,79.78744785173983,79.8017456015568,79.8017456016568,79.81598534843152,79.81598534853153,79.83019609378512,79.83019609388512,79.87279932832477,79.87279932842478,79.90110481294744,79.90110481304744,79.91519955221652,79.91519955231652,79.92920728692219,79.9292072870222,79.94315701858562,79.94315701868562,79.95704874720678,79.95704874730679,79.97091147430683,79.97091147440683,79.98462919380121,79.98462919390121,80.01200662974773,80.01200662984773,80.0256083431576,80.0256083432576,80.03918105504634,80.03918105514634,80.05255075628716,80.05255075638716,80.06583345296461,80.06583345306461,80.07905814659979,80.0790581466998,80.10547853234903,80.10547853244903,80.11858721989968,80.11858721999968,80.13157990136582,80.13157990146583,80.15753626277699,80.15753626287699,80.17047094120086,80.17047094130086,80.18323161049797,80.18323161059797,80.19596327827395,80.19596327837395,80.22139761230477,80.22139761240477,80.23401327399623,80.23401327409623,80.24657093264541,80.24657093274541,80.25909958977348,80.25909958987349,80.27157024385929,80.27157024395929,80.28401189642396,80.28401189652396,80.30880819698993,80.30880819708993,80.3210468389067,80.3210468390067,80.33319847626008,80.33319847636008,80.35738574488232,80.35738574498232,80.36936337310892,80.36936337320893,80.39328962804099,80.39328962814099,80.40520925322534,80.40520925332534,80.42896149903062,80.42896149913062,80.4762629799933,80.4762629800933,80.49975421210843,80.49975421220843,80.52307143509677,80.52307143519677,80.53467204354868,80.53467204364868,80.55781525741025,80.55781525751026,80.5808424651873,80.5808424652873,80.59229806603356,80.59229806613357,80.61518026620496,80.61518026630496,80.62657786400896,80.62657786410897,80.6492860550536,80.6492860551536,80.66056764677307,80.66056764687308,80.68310182869091,80.68310182879091,80.7055780075665,80.7055780076665,80.71674359320146,80.71674359330146,80.72779317275192,80.72779317285192,80.73881375078123,80.73881375088123,80.76082590531874,80.76082590541874,80.77178848030579,80.77178848040579,80.78272205377172,80.78272205387172,80.80450219614019,80.8045021962402,80.8153197635216,80.8153197636216,80.82607932786074,80.82607932796074,80.84748245045452,80.84748245055452,80.8580680056669,80.8580680057669,80.87918111304938,80.87918111314939,80.91073476803858,80.91073476813858,80.92120431716644,80.92120431726644,80.94211441390101,80.94211441400101,80.9525259599866,80.9525259600866,80.96282149998767,80.96282150008767,80.9730590369465,80.9730590370465,80.9830935632574,80.9830935633574,80.99309908804717,80.99309908814718,81.00301760827355,81.00301760837355,81.02273864264181,81.02273864274181,81.0323671476569,81.0323671477569,81.04190864810859,81.04190864820859,81.05130514095464,81.05130514105464,81.06067263227956,81.06067263237956,81.06998212056223,81.06998212066223,81.07926260732376,81.07926260742376,81.0884560895219,81.0884560896219,81.11600753459518,81.11600753469519,81.12511401222994,81.12511401232995,81.13416248682243,81.13416248692243,81.1431819598938,81.1431819599938,81.16119190451539,81.1611919046154,81.17012437302337,81.17012437312337,81.17899883848908,81.17899883858908,81.18784430243366,81.18784430253366,81.1965447587726,81.1965447588726,81.22258812474715,81.22258812484715,81.2312015765227,81.2312015766227,81.23978602677711,81.23978602687711,81.24834147551039,81.24834147561039,81.25683892120142,81.25683892130142,81.26530736537131,81.26530736547132,81.27368880497782,81.27368880507782,81.29880412227621,81.29880412237621,81.30712755884046,81.30712755894046,81.31536399084132,81.31536399094132,81.3318078533219,81.3318078534219,81.33995728075938,81.33995728085938,81.36437656155066,81.36437656165066,81.37246798594586,81.37246798604586,81.38053040881994,81.38053040891994,81.38853482865176,81.38853482875176,81.39651024696245,81.39651024706245,81.42838291868408,81.42838291878408,81.43630033395252,81.43630033405252,81.45201915840485,81.45201915850485,81.45976256454651,81.45976256464651,81.47522037530868,81.47522037540868,81.4906201830286,81.4906201831286,81.49824758308573,81.49824758318573,81.5058169801006,81.5058169802006,81.51335737559435,81.51335737569435,81.53594956055446,81.53594956065446,81.55094334697854,81.55094334707854,81.58084391526336,81.58084391536336,81.5956926940818,81.5956926941818,81.61045446833687,81.61045446843687,81.6177918531827,81.6177918532827,81.63971700315682,81.63971700325682,81.66143914248302,81.66143914258302,81.6758238969634,81.6758238970634,81.69012164688039,81.69012164698039,81.69722701955718,81.69722701965718,81.70430339071284,81.70430339081284,81.71842713150305,81.71842713160305,81.72544549961646,81.72544549971646,81.73942423280101,81.73942423290102,81.74635559635102,81.74635559645102,81.75325795837992,81.75325795847992,81.76703368091657,81.76703368101657,81.78075140041095,81.78075140051095,81.78753775635532,81.78753775645532,81.79429511077856,81.79429511087856,81.81453817252715,81.81453817262715,81.84142258261446,81.84142258271446,81.86149163523626,81.86149163533626,81.868132983575,81.868132983675,81.88802802707002,81.88802802717002,81.89458237084536,81.89458237094536,81.90766205687488,81.90766205697489,81.92068373986216,81.92068373996216,81.93364741980717,81.93364741990717,81.94655309670992,81.94655309680992,81.9529624328796,81.9529624329796,81.95931376600703,81.95931376610703,81.97198743074074,81.97198743084074,81.97828076082591,81.97828076092591,81.98454508938994,81.98454508948994,82.00330907356091,82.00330907366092,82.00951539908269,82.00951539918269,82.01569272308332,82.01569272318332,82.02801836956348,82.02801836966349,82.03413769052187,82.03413769062188,82.05246665187589,82.05246665197589,82.07070860866652,82.07070860876652,82.07674092506151,82.07674092516152,82.08877655633037,82.08877655643037,82.10075418455698,82.10075418465698,82.11267380974132,82.11267380984133,82.13048074371501,82.13048074381501,82.14228436281483,82.14228436291484,82.16580459645108,82.16580459655108,82.17163390219817,82.17163390229817,82.17743420642412,82.17743420652413,82.18900581335491,82.18900581345491,82.20051941724344,82.20051941734344,82.21771731927339,82.21771731937339,82.2462548160651,82.2462548161651,82.25759441082685,82.25759441092686,82.26887600254634,82.26887600264634,82.30260477162027,82.30260477172027,82.31377035725524,82.31377035735524,82.32487793984795,82.32487793994795,82.3359275193984,82.3359275194984,82.34691909590659,82.34691909600659,82.35237138187898,82.35237138197898,82.37412252272631,82.37412252282631,82.38488208706546,82.38488208716547,82.39558364836236,82.39558364846236,82.43290860605639,82.43290860615639,82.44349416126876,82.44349416136876,82.46985654397574,82.46985654407574,82.50657246972604,82.50657246982604,82.52220428961499,82.52220428971499,82.5273665603761,82.5273665604761,82.55314891266048,82.55314891276048,82.57365298809923,82.57365298819923,82.58893678973462,82.58893678983462,82.5939830544112,82.5939830545112,82.60909284691982,82.60909284701982,82.64418468748686,82.64418468758686,82.66410873250302,82.66410873260303,82.68887603154785,82.68887603164785,82.73814961594735,82.73814961604735,82.7626268997809,82.7626268998809,82.79670368710839,82.79670368720839,82.83057746378798,82.83057746388798,82.86424822981965,82.86424822991965,82.892930734217,82.892930734317,82.90717048109174,82.90717048119174,82.96386845490046,82.96386845500047,82.98733068549446,82.98733068559446,83.0153171533847,83.0153171534847,83.02920888200586,83.02920888210586,83.04762484792327,83.04762484802328,83.0888360094487,83.0888360095487,83.16621206782295,83.16621206792296,83.23857086304177,83.23857086314177,83.31496086969761,83.31496086979762,83.38639161624027,83.38639161634028,83.4928562003077,83.4928562004077,83.54572597332731,83.54572597342731,83.62890233592752,83.62890233602752,83.68977652877894,83.68977652887894,83.75024470033453,83.75024470043454,83.81030685059432,83.81030685069432,83.89980554480083,83.89980554490083,83.98446098497867,83.98446098507867,84.0475102919148,84.0475102920148,84.12265323316208,84.12265323326208,84.16409640685654,84.16409640695655,84.25054994134442,84.25054994144442,84.31594837149208,84.31594837159209,84.42148490688336,84.42148490698337,84.48595528835487,84.48595528845487,84.56597048515195,84.56597048525195,84.67719131868466,84.67719131878466,84.76393486838384,84.76393486848384,84.86178600067574,84.86178600077574,84.93170866811964,84.93170866821964,85.00110930818322,85.00110930828322,85.05850331849905,85.05850331859905,85.12306070453396,85.12306070463396,85.19843565795026,85.19843565805026,85.28819536584695,85.28819536594695,85.36983464782728,85.36983464792728,85.42137035087491,85.42137035097491,85.50173356592553,85.50173356602554,85.56695798694642,85.56695798704642,85.62446800334678,85.62446800344678,85.74215617609144,85.74215617619144,85.82350544286048,85.82350544296048,85.92524277898376,85.92524277908376,86.01569852338756,86.01569852348756,86.10194904722752,86.10194904732752,86.18405235354594,86.18405235364594,86.27224597930162,86.27224597940162,86.38659897711635,86.38659897721635,86.48662522349298,86.48662522359298,86.59900611787089,86.59900611797089,86.73006399185637,86.73006399195637,86.8534654642636,86.85346546436361,87.01761407385817,87.01761407395817,87.18666394052369,87.18666394062369,87.37946605299447,87.37946605309448,87.59868855121451,87.59868855131451,87.88725368645585,87.88725368655585,88.27149483990435,88.27149484000435,88.72519463645868,88.72519463655868,89.31934879984455,89.31934879994455,90.08403190747354,90.08403190757355,91.36192593301519,91.36192593311519]],"filenames":["genome.ctg.filtered"]}

{"coord\_y":[[16870,16870,16870,16684,16684,16131,16131,15552,15552,15029,15029,14955,14955,14538,14538,14501,14501,14210,14210,12773,12773,12462,12462,12093,12093,12041,12041,11690,11690,11572,11572,11041,11041,10876,10876,10638,10638,10499,10499,10369,10369,9721,9721,9717,9717,9552,9552,9456,9456,9360,9360,9188,9188,9142,9142,9012,9012,8816,8816,8630,8630,8608,8608,8601,8601,8558,8558,8478,8478,8442,8442,8356,8356,8246,8246,8198,8198,8138,8138,8017,8017,7944,7944,7908,7908,7896,7896,7895,7895,7794,7794,7769,7769,7689,7689,7632,7632,7628,7628,7483,7483,7464,7464,7409,7409,7360,7360,7264,7264,7233,7233,7214,7214,7158,7158,7154,7154,7085,7085,6839,6839,6836,6836,6801,6801,6790,6790,6770,6770,6767,6767,6758,6758,6704,6704,6668,6668,6650,6650,6648,6648,6639,6639,6633,6633,6559,6559,6547,6547,6502,6502,6489,6489,6483,6483,6445,6445,6415,6415,6399,6399,6337,6337,6300,6300,6262,6262,6216,6216,6182,6182,6141,6141,6119,6119,6096,6096,6056,6056,6048,6048,5981,5981,5977,5977,5967,5967,5964,5964,5941,5941,5912,5912,5908,5908,5861,5861,5841,5841,5783,5783,5760,5760,5754,5754,5741,5741,5738,5738,5686,5686,5684,5684,5683,5683,5665,5665,5610,5610,5603,5603,5571,5571,5538,5538,5523,5523,5508,5508,5501,5501,5496,5496,5487,5487,5470,5470,5466,5466,5426,5426,5420,5420,5405,5405,5365,5365,5332,5332,5320,5320,5253,5253,5230,5230,5218,5218,5214,5214,5201,5201,5196,5196,5190,5190,5184,5184,5180,5180,5150,5150,5141,5141,5095,5095,5085,5085,5059,5059,5046,5046,5043,5043,5041,5041,5026,5026,5022,5022,4989,4989,4988,4988,4935,4935,4904,4904,4891,4891,4874,4874,4830,4830,4828,4828,4820,4820,4818,4818,4809,4809,4786,4786,4779,4779,4768,4768,4758,4758,4754,4754,4744,4744,4680,4680,4662,4662,4643,4643,4633,4633,4592,4592,4590,4590,4589,4589,4586,4586,4558,4558,4557,4557,4526,4526,4520,4520,4513,4513,4511,4511,4506,4506,4488,4488,4473,4473,4461,4461,4452,4452,4430,4430,4427,4427,4406,4406,4404,4404,4392,4392,4387,4387,4381,4381,4380,4380,4353,4353,4342,4342,4336,4336,4327,4327,4317,4317,4299,4299,4297,4297,4291,4291,4275,4275,4272,4272,4253,4253,4236,4236,4186,4186,4179,4179,4178,4178,4157,4157,4154,4154,4130,4130,4119,4119,4118,4118,4108,4108,4105,4105,4104,4104,4095,4095,4087,4087,4082,4082,4080,4080,4067,4067,4038,4038,4013,4013,4012,4012,4005,4005,3995,3995,3969,3969,3938,3938,3915,3915,3906,3906,3890,3890,3889,3889,3882,3882,3854,3854,3852,3852,3844,3844,3840,3840,3826,3826,3803,3803,3774,3774,3773,3773,3766,3766,3761,3761,3741,3741,3738,3738,3730,3730,3723,3723,3683,3683,3680,3680,3677,3677,3650,3650,3638,3638,3634,3634,3597,3597,3588,3588,3571,3571,3557,3557,3553,3553,3534,3534,3527,3527,3516,3516,3513,3513,3481,3481,3471,3471,3462,3462,3453,3453,3420,3420,3415,3415,3386,3386,3385,3385,3373,3373,3364,3364,3353,3353,3336,3336,3316,3316,3304,3304,3298,3298,3292,3292,3288,3288,3287,3287,3282,3282,3274,3274,3272,3272,3265,3265,3236,3236,3208,3208,3205,3205,3193,3193,3192,3192,3183,3183,3182,3182,3180,3180,3179,3179,3166,3166,3162,3162,3144,3144,3139,3139,3138,3138,3135,3135,3133,3133,3084,3084,3074,3074,3068,3068,3041,3041,3031,3031,3003,3003,2990,2990,2987,2987,2975,2975,2959,2959,2941,2941,2938,2938,2937,2937,2932,2932,2931,2931,2930,2930,2929,2929,2923,2923,2909,2909,2908,2908,2906,2906,2901,2901,2900,2900,2899,2899,2896,2896,2893,2893,2877,2877,2876,2876,2870,2870,2859,2859,2850,2850,2848,2848,2839,2839,2832,2832,2830,2830,2828,2828,2821,2821,2804,2804,2803,2803,2799,2799,2796,2796,2794,2794,2783,2783,2779,2779,2776,2776,2761,2761,2746,2746,2726,2726,2717,2717,2700,2700,2691,2691,2689,2689,2684,2684,2676,2676,2670,2670,2664,2664,2657,2657,2652,2652,2645,2645,2619,2619,2618,2618,2615,2615,2612,2612,2601,2601,2599,2599,2592,2592,2585,2585,2583,2583,2572,2572,2570,2570,2567,2567,2566,2566,2551,2551,2543,2543,2515,2515,2489,2489,2486,2486,2482,2482,2480,2480,2477,2477,2463,2463,2449,2449,2445,2445,2444,2444,2433,2433,2431,2431,2430,2430,2423,2423,2413,2413,2409,2409,2407,2407,2402,2402,2401,2401,2373,2373,2351,2351,2345,2345,2342,2342,2340,2340,2336,2336,2335,2335,2332,2332,2326,2326,2320,2320,2313,2313,2307,2307,2301,2301,2300,2300,2293,2293,2287,2287,2286,2286,2283,2283,2281,2281,2277,2277,2271,2271,2270,2270,2260,2260,2253,2253,2248,2248,2240,2240,2233,2233,2229,2229,2222,2222,2221,2221,2218,2218,2210,2210,2199,2199,2181,2181,2169,2169,2168,2168,2157,2157,2153,2153,2151,2151,2142,2142,2141,2141,2139,2139,2137,2137,2131,2131,2127,2127,2115,2115,2108,2108,2105,2105,2102,2102,2095,2095,2074,2074,2068,2068,2063,2063,2062,2062,2061,2061,2057,2057,2053,2053,2047,2047,2026,2026,2025,2025,2023,2023,2022,2022,2021,2021,2020,2020,2014,2014,2008,2008,1998,1998,1997,1997,1996,1996,1988,1988,1986,1986,1982,1982,1978,1978,1974,1974,1954,1954,1953,1953,1950,1950,1949,1949,1942,1942,1918,1918,1914,1914,1913,1913,1910,1910,1908,1908,1907,1907,1906,1906,1892,1892,1890,1890,1888,1888,1883,1883,1881,1881,1878,1878,1877,1877,1864,1864,1859,1859,1856,1856,1851,1851,1848,1848,1841,1841,1837,1837,1830,1830,1826,1826,1809,1809,1806,1806,1797,1797,1783,1783,1782,1782,1777,1777,1772,1772,1766,1766,1755,1755,1753,1753,1747,1747,1743,1743,1739,1739,1737,1737,1734,1734,1731,1731,1728,1728,1727,1727,1726,1726,1723,1723,1720,1720,1717,1717,1712,1712,1711,1711,1709,1709,1708,1708,1707,1707,1706,1706,1705,1705,1694,1694,1681,1681,1680,1680,1677,1677,1675,1675,1672,1672,1670,1670,1667,1667,1663,1663,1659,1659,1657,1657,1653,1653,1649,1649,1647,1647,1642,1642,1640,1640,1638,1638,1637,1637,1634,1634,1630,1630,1626,1626,1625,1625,1621,1621,1619,1619,1614,1614,1608,1608,1604,1604,1603,1603,1601,1601,1590,1590,1589,1589,1577,1577,1574,1574,1567,1567,1563,1563,1560,1560,1558,1558,1552,1552,1549,1549,1548,1548,1535,1535,1528,1528,1511,1511,1506,1506,1502,1502,1500,1500,1496,1496,1495,1495,1491,1491,1490,1490,1488,1488,1486,1486,1483,1483,1477,1477,1467,1467,1466,1466,1455,1455,1454,1454,1453,1453,1447,1447,1446,1446,1442,1442,1439,1439,1435,1435,1426,1426,1424,1424,1419,1419,1416,1416,1414,1414,1408,1408,1406,1406,1403,1403,1402,1402,1391,1391,1386,1386,1379,1379,1372,1372,1371,1371,1368,1368,1362,1362,1361,1361,1359,1359,1354,1354,1350,1350,1348,1348,1344,1344,1343,1343,1340,1340,1336,1336,1334,1334,1327,1327,1326,1326,1321,1321,1319,1319,1310,1310,1302,1302,1301,1301,1299,1299,1296,1296,1295,1295,1292,1292,1291,1291,1288,1288,1286,1286,1280,1280,1276,1276,1272,1272,1269,1269,1266,1266,1258,1258,1254,1254,1249,1249,1246,1246,1241,1241,1233,1233,1232,1232,1228,1228,1224,1224,1223,1223,1220,1220,1219,1219,1218,1218,1215,1215,1207,1207,1205,1205,1203,1203,1194,1194,1193,1193,1184,1184,1183,1183,1182,1182,1178,1178,1176,1176,1170,1170,1169,1169,1165,1165,1164,1164,1155,1155,1128,1128,1126,1126,1121,1121,1118,1118,1117,1117,1116,1116,1113,1113,1107,1107,1098,1098,1096,1096,1093,1093,1092,1092,1090,1090,1089,1089,1087,1087,1082,1082,1073,1073,1067,1067,1066,1066,1063,1063,1058,1058,1056,1056,1048,1048,1042,1042,1039,1039,1035,1035,1033,1033,1030,1030,1021,1021,1019,1019,1015,1015,1014,1014,1013,1013,1009,1009,1008,1008,1005,1005,1000,1000,999,999,998,998,991,991,989,989,988,988,983,983,982,982,980,980,978,978,976,976,972,972,969,969,968,968,967,967,963,963,962,962,953,953,948,948,947,947,946,946,944,944,941,941,940,940,937,937,934,934,933,933,932,932,930,930,927,927,924,924,920,920,918,918,917,917,914,914,909,909,908,908,907,907,905,905,902,902,901,901,900,900,899,899,898,898,891,891,886,886,882,882,881,881,877,877,876,876,869,869,868,868,867,867,865,865,863,863,860,860,854,854,851,851,850,850,848,848,847,847,845,845,841,841,840,840,839,839,835,835,831,831,830,830,824,824,823,823,821,821,820,820,817,817,811,811,810,810,809,809,808,808,806,806,805,805,800,800,796,796,788,788,787,787,785,785,781,781,780,780,778,778,772,772,770,770,767,767,761,761,759,759,756,756,753,753,752,752,749,749,746,746,744,744,741,741,737,737,735,735,734,734,733,733,732,732,729,729,725,725,724,724,722,722,720,720,719,719,718,718,715,715,709,709,705,705,698,698,695,695,694,694,693,693,692,692,691,691,687,687,683,683,681,681,678,678,668,668,667,667,661,661,660,660,658,658,655,655,654,654,653,653,648,648,646,646,644,644,643,643,641,641,637,637,635,635,634,634,632,632,630,630,629,629,628,628,619,619,614,614,612,612,611,611,610,610,607,607,605,605,601,601,599,599,594,594,590,590,586,586,585,585,583,583,578,578,577,577,574,574,573,573,570,570,569,569,564,564,563,563,562,562,559,559,557,557,554,554,553,553,552,552,551,551,544,544,543,543,540,540,537,537,533,533,530,530,529,529,522,522,516,516,514,514,513,513,511,511,510,510,509,509,507,507,499,499,498,498,496,496,494,494,493,493,491,491,490,490,489,489,487,487,486,486,483,483,481,481,479,479,478,478,473,473,471,471,469,469,468,468,461,461,458,458,456,456,455,455,452,452,448,448,447,447,446,446,440,440,439,439,438,438,435,435,433,433,432,432,430,430,429,429,426,426,422,422,419,419,415,415,413,413,412,412,411,411,408,408,407,407,403,403,401,401,400,400,398,398,396,396,395,395,394,394,393,393,390,390,389,389,388,388,387,387,385,385,381,381,380,380,379,379,378,378,377,377,374,374,373,373,371,371,367,367,365,365,363,363,362,362,361,361,360,360,359,359,355,355,353,353,346,346,345,345,342,342,338,338,332,332,329,329,324,324,323,323,321,321,320,320,317,317,316,316,314,314,312,312,311,311,310,310,308,308,306,306,305,305,300,300,298,298,297,297,296,296,295,295,293,293,292,292,289,289,288,288,287,287,284,284,283,283,281,281,280,280,279,279,278,278,276,276,275,275,274,274,273,273,269,269,267,267,266,266,265,265,263,263,261,261,260,260,259,259,258,258,257,257,255,255,254,254,253,253,250,250,249,249,247,247,246,246,245,245,244,244,243,243,242,242,240,240,239,239,238,238,237,237,236,236,234,234,233,233,232,232,231,231,230,230,229,229,228,228,226,226,225,225,224,224,223,223,222,222,221,221,219,219,218,218,217,217,216,216,215,215,214,214,213,213,212,212,211,211,210,210,209,209,208,208,207,207,206,206,205,205,204,204,203,203,202,202,201,201,200,200,199,199,198,198,197,197,196,196,195,195,194,194,193,193,192,192,191,191,190,190,189,189,188,188,186,186,185,185,184,184,183,183,182,182,181,181,180,180,179,179,178,178,177,177,176,176,175,175,174,174,173,173,172,172,171,171,170,170,169,169,168,168,167,167,166,166,165,165,164,164,163,163,162,162,161,161,160,160,159,159,158,158,157,157,156,156,155,155,154,154,153,153,152,152,151,151,150,150,149,149,148,148,147,147,146,146,145,145,144,144,143,143,142,142,141,141,140,140,139,139,138,138,137,137,136,136,135,135,134,134,133,133,132,132,131,131,130,130,129,129,128,128,127,127,126,126,125,125,124,124,123,123,122,122,121,121,120,120,119,119,118,118,117,117,116,116,115,115,114,114,113,113,112,112,111,111,110,110,109,109,108,108,107,107,106,106,105,105,104,104,103,103,102,102,101,101,0.0]],"coord\_x":[[0.0,1e-10,0.6544088810306378,0.6544088811306378,1.3016025841198589,1.3016025842198589,1.9273447097811047,1.9273447098811047,2.5306266847537473,2.5306266848537473,3.1136208206760623,3.1136208207760623,3.6937444019335124,3.6937444020335124,4.257692019741658,4.257692019841658,4.820204360217371,4.820204360317371,5.371428438429899,5.371428438529899,5.866909448353097,5.866909448453097,6.350326370428012,6.350326370528012,6.819429310457857,6.819429310557857,7.2865151039664475,7.2865151040664475,7.739985158456557,7.739985158556557,8.188877841994586,8.188877842094586,8.617172356248247,8.617172356348247,9.039066309424845,9.039066309524845,9.451727938138772,9.451727938238772,9.858997579036261,9.858997579136261,10.261224353630611,10.261224353730611,10.638314379267769,10.638314379367769,11.015249239787904,11.015249239887904,11.385783539230978,11.385783539330978,11.752593875865578,11.752593875965578,12.115680249691707,12.115680249791707,12.472094523485985,12.472094523585985,12.82672439843454,12.82672439853454,13.176311407079952,13.176311407179952,13.518295324991398,13.518295325091398,13.853064064961428,13.853064065061428,14.18697939678785,14.18697939688785,14.520623189659487,14.520623189759487,14.852598957523162,14.852598957623162,15.181471423046442,15.181471423146442,15.508947402516545,15.508947402616545,15.833087331970725,15.833087332070725,16.152960220706863,16.15296022080686,16.470971128038762,16.47097112813876,16.786654558615368,16.786654558715366,17.097644244402126,17.097644244502124,17.405802166803277,17.405802166903275,17.71256360315125,17.712563603251247,18.01885954414816,18.01885954424816,18.32511669386582,18.325116693965818,18.62745592437873,18.627455924478728,18.928825372910264,18.928825373010262,19.227091519101407,19.227091519201405,19.523146562375018,19.523146562475016,19.81904644053161,19.81904644063161,20.109321583196238,20.109321583296236,20.39885969155502,20.39885969165502,20.686264279554784,20.686264279654782,20.971768094871056,20.971768094971054,21.253547947378856,21.253547947478854,21.534125270229747,21.534125270329746,21.8139655587748,21.8139655588748,22.091633535681577,22.091633535781575,22.369146347471332,22.36914634757133,22.643982560992498,22.643982561092496,22.90927611981695,22.90927611991695,23.17445330480364,23.17445330490364,23.438272795016406,23.438272795116404,23.701665581157368,23.701665581257366,23.96428254171323,23.96428254181323,24.226783128431332,24.22678312853133,24.488934593636134,24.488934593736133,24.748991329761175,24.748991329861173,25.007651579833034,25.007651579933032,25.265613586878306,25.265613586978304,25.52349801136507,25.52349801146507,25.781033314338536,25.781033314438535,26.038335869636477,26.038335869736475,26.29276787026955,26.29276787036955,26.546734375551562,26.54673437565156,26.798955273267108,26.798955273367106,27.30289278206788,27.302892782167877,27.55437664547758,27.554376645577577,27.80438644027559,27.804386440375588,28.05323249669595,28.05323249679595,28.301457892648237,28.301457892748235,28.547278229286718,28.547278229386716,28.791663288592765,28.791663288692764,29.034574279287124,29.034574279387122,29.275700871135758,29.275700871235756,29.515508559489724,29.515508559589723,29.753725805394236,29.753725805494234,29.991089643155142,29.99108964325514,30.227561281493184,30.227561281593182,30.46248126866103,30.462481268761028,30.697090925594836,30.697090925694834,30.92910156681856,30.929101566918558,31.160957042925265,31.160957043025263,31.392424606239423,31.39242460633942,31.623775795715815,31.623775795815813,32.085585975245735,32.08558597534574,32.314920018200866,32.31492001830087,32.54409889603898,32.54409889613898,32.77145458375212,32.77145458385212,32.99803444588015,32.99803444598015,33.222364413811405,33.22236441391141,33.44580218231979,33.44580218241979,33.669007203152646,33.66900720325265,33.891707937355186,33.89170793745519,34.11429229771996,34.114292297819965,34.33485951156349,34.33485951166349,34.5553491428485,34.5553491429485,34.77579998285425,34.775799982954254,34.99555257983342,34.995552579933424,35.21317165645357,35.21317165655357,35.43051919411893,35.43051919421893,35.64662541084814,35.64662541094814,35.86145151536193,35.86145151546193,36.0756957506869,36.0756957507869,36.28935811682304,36.28935811692304,36.5027489440044,36.502748944104404,36.715945814789485,36.71594581488949,36.92879356406127,36.928793564161275,37.140981861585736,37.14098186168574,37.35301499399317,37.35301499409317,37.56349647523041,37.56349647533041,37.77374520879212,37.77374520889212,37.983412073165006,37.98341207326501,38.191527286367695,38.1915272864677,38.39836238735497,38.39836238745497,38.60473199299119,38.60473199309119,38.80850258291733,38.80850258301733,39.0113809734206,39.0113809735206,39.21379386857282,39.21379386867282,39.416051598608014,39.416051598708016,39.6178050420129,39.6178050421129,39.8193645290215,39.8193645291215,40.020691268354575,40.02069126845458,40.221785260012126,40.22178526011213,40.422724086552655,40.42272408665266,40.622499174715536,40.62249917481554,40.82192514136512,40.82192514146512,41.21899267581856,41.218992675918564,41.41624633082987,41.41624633092987,41.61249141258055,41.612491412680555,41.808232207700925,41.80823220780093,42.00385662898353,42.00385662908353,42.19940346770762,42.19940346780762,42.39436843724289,42.39436843734289,42.58917824166114,42.58917824176114,42.782707933863975,42.78270793396398,42.97619883478755,42.976198834887555,43.167633797910625,43.16763379801063,43.35786623137679,43.35786623147679,43.547594378212644,43.547594378312645,43.736663073301166,43.73666307340117,43.92402495210247,43.92402495220247,44.11130924834526,44.11130924844526,44.298283214354015,44.29828321445402,44.48517959780426,44.48517959790426,44.671726859741206,44.67172685984121,44.85738192225529,44.857381922355295,45.042765445814595,45.0427654459146,45.22772226530209,45.22772226540209,45.41229117199704,45.41229117209704,45.78127382026992,45.78127382036992,45.9652996490553,45.9652996491553,46.14684283596836,46.14684283606836,46.327687779854834,46.327687779954836,46.50779568943547,46.50779568953547,46.68751568622355,46.68751568632355,46.86564524056218,46.86564524066218,47.0436972123423,47.043697212442304,47.221710392843164,47.221710392943166,47.399607199506264,47.399607199606265,47.576417850350225,47.57641785045023,47.753189709914935,47.75318971001494,47.92875903982274,47.92875903992274,48.10409562205501,48.104095622155015,48.279160665332505,48.27916066543251,48.454148126051486,48.45414812615149,48.62894163037419,48.628941630474195,48.80303689167031,48.80303689177031,48.976550283777605,48.97655028387761,49.149598180533836,49.14959818063384,49.32229695577678,49.32229695587678,49.49414232287611,49.49414232297611,49.665871316137675,49.66587131623768,49.83678569253489,49.83678569263489,50.00762248637359,50.00762248647359,50.177993784861236,50.17799378496124,50.34817112695261,50.34817112705261,50.518115721368446,50.51811572146845,50.68802152450503,50.688021524605034,50.85687996310173,50.856879963201735,51.025311697626634,51.025311697726636,51.193510684476,51.193510684576005,51.361360549812076,51.36136054991208,51.5288225023556,51.528822502455604,51.69558621187254,51.69558621197254,51.86227233883096,51.862272338930964,52.02872571811386,52.02872571821386,52.19455843692868,52.19455843702868,52.36027478190573,52.36027478200573,52.52525409257694,52.52525409267694,52.68957395150081,52.68957395160081,52.85195424646194,52.851954246561945,53.176443297429415,53.17644329752942,53.33851326215651,53.33851326225651,53.499768610019245,53.49976861011925,53.66090758404422,53.66090758414422,53.821115567367066,53.82111556746707,53.980896846618116,53.98089684671812,54.14063933458991,54.140639334689915,54.459736397740954,54.459736397840956,54.61897459908243,54.61897459918243,54.93741221048614,54.93741221058614,55.09626249903507,55.09626249913507,55.25480245734996,55.25480245744996,55.41314845926858,55.41314845936858,55.57141687862868,55.571416878728684,55.72918101135848,55.72918101145848,55.885820196989876,55.88582019708988,56.0414896006399,56.0414896007399,56.19712021301067,56.19712021311067,56.35247928642666,56.35247928652666,56.5074504470501,56.5074504471501,56.66141303441291,56.66141303451291,56.814173092118814,56.814173092218816,56.96604095040186,56.96604095050186,57.11755968717161,57.11755968727161,57.26845776347328,57.26845776357328,57.41931704849569,57.419317048595694,57.871623364608155,57.87162336470816,58.021124954856646,58.02112495495665,58.17054896254663,58.170548962646635,58.319662640002576,58.31966264010258,58.4686211523415,58.4686211524415,58.61703658677086,58.61703658687086,58.76455982177735,58.76455982187735,58.910958109685446,58.91095810978545,59.05731760631429,59.057317606414294,59.349765060617194,59.349765060717196,59.495659061894976,59.49565906199498,59.64077723758766,59.640777237687665,59.78577903944259,59.78577903954259,59.93047051106347,59.93047051116347,60.074890443729565,60.07489044382957,60.217758725225465,60.21775872532547,60.3605106328836,60.360510632983605,60.50314616670397,60.50314616680397,60.64473433598446,60.64473433608446,60.78585700991389,60.78585701001389,60.9268245187263,60.9268245188263,61.066356750206275,61.06635675030628,61.205539860172955,61.20553986027296,61.3440635183923,61.344063518492305,61.48204409870208,61.482044098802085,61.61986951389484,61.61986951399484,61.756957894781756,61.75695789488176,61.89377473671389,61.89377473681389,62.03016487457422,62.03016487467422,62.16643863859678,62.16643863869678,62.30147108168318,62.301471081783184,62.571148055063446,62.57114805516345,62.705443463844006,62.70544346394401,62.83938975111127,62.83938975121127,62.97205592616313,62.97205592626313,63.10452814481871,63.10452814491871,63.23587541637589,63.23587541647589,63.36718389665383,63.36718389675383,63.4980268815807,63.4980268816807,63.628520744994276,63.62852074509428,63.75858790433605,63.758587904436055,64.01806277127227,64.01806277137227,64.1466946532816,64.1466946533816,64.27486103993989,64.2748610400399,64.40279467892265,64.40279467902265,64.53049557022986,64.53049557032986,64.65804129642007,64.65804129652007,64.78554823133102,64.78554823143102,64.91286120984569,64.91286120994569,65.03986385812632,65.03986385822633,65.16678892384846,65.16678892394846,65.29344245061579,65.29344245071579,65.41897103028474,65.41897103038474,65.54341345413455,65.54341345423455,65.6677395041466,65.6677395042466,65.79160005880757,65.79160005890758,66.0392823768503,66.0392823769503,66.16275501871873,66.16275501881873,66.2861888693079,66.28618886940791,66.40954513733858,66.40954513743858,66.53286261409,66.53286261419001,66.6556758042111,66.6556758043111,66.77833382921519,66.77833382931519,66.90029361119268,66.90029361129268,67.0220594367739,67.0220594368739,67.14378647107587,67.14378647117587,67.26539713154007,67.26539713164007,67.38693020944575,67.38693020954575,67.50656251466795,67.50656251476795,67.6258069070976,67.6258069071976,67.74481855185172,67.74481855195172,67.86278283206596,67.86278283216596,67.98035919948765,67.98035919958765,68.09684941109019,68.09684941119019,68.21283533606243,68.21283533616243,68.3287048871969,68.32870488729691,68.44410894298031,68.44410894308031,68.55889233829565,68.55889233839565,68.67297749058439,68.67297749068439,68.78694626903537,68.78694626913537,68.90087625620708,68.90087625630709,69.01461228698254,69.01461228708254,69.12830952647873,69.12830952657873,69.24196797469567,69.24196797479567,69.35558763163336,69.35558763173336,69.46897454089552,69.46897454099552,69.5818183722481,69.5818183723481,69.69462341232143,69.69462341242144,69.80735086983626,69.80735086993626,69.91988437095479,69.91988437105479,70.14491258191264,70.14491258201264,70.25736850047267,70.25736850057267,70.36970804519494,70.36970804529494,70.48193121607945,70.48193121617945,70.59353372649588,70.59353372659588,70.70509744563306,70.70509744573306,70.8164284170947,70.8164284171947,70.92733268448454,70.92733268458454,71.03788783036109,71.03788783046109,71.14836539367911,71.14836539377912,71.25849383548386,71.25849383558386,71.36835073833382,71.36835073843382,71.47813005862525,71.47813005872526,71.5878317963582,71.5878317964582,71.69726199513634,71.69726199523635,71.80603274216716,71.80603274226716,71.91476469791873,71.91476469801873,72.02334148855327,72.02334148865327,72.13180190535006,72.13180190545006,72.24018473958833,72.24018473968833,72.34814086975479,72.34814086985479,72.45594183480424,72.45594183490424,72.56362642601592,72.56362642611592,72.67072914803877,72.67072914813878,72.7772500008728,72.7772500009728,72.88299502812174,72.88299502822174,72.98839093385737,72.98839093395738,73.09312738784568,73.09312738794569,73.1975147203207,73.1975147204207,73.3018244702372,73.3018244703372,73.40594026375743,73.40594026385743,73.50974572704361,73.50974572714361,73.61331844265428,73.61331844275428,73.7166584105894,73.7166584106894,73.92306680750487,73.92306680760487,74.02594128008894,74.02594128018895,74.12854421371823,74.12854421381823,74.33274150771616,74.33274150781617,74.43429707680556,74.43429707690557,74.5357362720572,74.53573627215721,74.63705909347108,74.63705909357108,74.73795521081314,74.73795521091314,74.83877374559671,74.83877374569671,74.93932074142548,74.93932074152548,75.03959619829946,75.03959619839947,75.13979407261495,75.13979407271495,75.23956524285862,75.23956524295862,75.33925883054378,75.33925883064379,75.4388360443912,75.4388360444912,75.53837446695934,75.53837446705934,75.63733102033865,75.63733102043865,75.73597724348394,75.73597724358395,75.83353731081009,75.83353731091009,75.9300888048756,75.9300888049756,76.02652392510335,76.02652392520335,76.12280388021408,76.12280388031408,76.2190062527663,76.21900625286631,76.31509225148076,76.31509225158077,76.41063517228565,76.41063517238565,76.50563501518097,76.50563501528097,76.60047969295927,76.60047969305927,76.79013025723661,76.79013025733661,76.97931532616289,76.97931532626289,77.07361692603162,77.07361692613162,77.1678797346211,77.1678797347211,77.35613381284527,77.35613381294527,77.44973716968741,77.44973716978741,77.54318536141253,77.54318536151253,77.63655597057914,77.63655597067914,77.72973262334948,77.72973262344948,77.82287048484056,77.82287048494057,77.91492219051251,77.91492219061251,78.00612048804085,78.00612048814085,78.09708603789365,78.09708603799365,78.18793521390869,78.18793521400869,78.27870680736523,78.27870680746523,78.36932323570474,78.36932323580474,78.45990087276499,78.45990087286499,78.55036213598748,78.55036213608749,78.64059065153445,78.64059065163445,78.73058641940588,78.73058641950588,78.82031064832253,78.82031064842253,78.90980212956364,78.90980212966365,79.2675353068526,79.2675353069526,79.35675524913893,79.35675524923893,79.44570365247048,79.44570365257049,79.5344193081265,79.5344193082265,79.62309617250327,79.62309617260327,79.71165666304226,79.71165666314226,79.80013957102275,79.80013957112276,79.88846731388622,79.88846731398623,79.97656230907417,79.97656230917417,80.06461851298286,80.06461851308286,80.15228680409899,80.152286804199,80.23968355626035,80.23968355636035,80.32688635202543,80.32688635212543,80.41377881755646,80.41377881765646,80.58729220966376,80.58729220976376,80.673757971123,80.673757971223,80.75995219362744,80.75995219372744,80.84610762485264,80.84610762495264,80.93214668224007,80.93214668234008,81.01787540939347,81.01787540949347,81.10317743247505,81.10317743257505,81.18778121253006,81.18778121263006,81.271919497234,81.271919497334,81.35601899065867,81.35601899075867,81.43969178001156,81.43969178011156,81.52320940424741,81.52320940434741,81.60664944592476,81.60664944602476,81.68974036608881,81.68974036618881,81.77279249497361,81.77279249507362,81.85576704129991,81.85576704139991,81.9386640050677,81.9386640051677,82.02132822115995,82.02132822125995,82.10383727213518,82.10383727223518,82.18588082775935,82.18588082785935,82.26765284442874,82.26765284452874,82.34930848726036,82.34930848736036,82.43084775625422,82.43084775635423,82.5121154862933,82.5121154863933,82.59256859946801,82.59256859956801,82.67278896496721,82.67278896506721,82.75281537407012,82.75281537417013,82.83280299189379,82.83280299199379,82.9127518184382,82.9127518185382,82.99254547986558,82.99254547996559,83.07218397617595,83.07218397627595,83.15158972481079,83.15158972491079,83.30958660521611,83.30958660531611,83.38813894570734,83.38813894580734,83.46661370364006,83.46661370374007,83.54504967029352,83.54504967039352,83.62344684566773,83.62344684576773,83.78020240513689,83.78020240523689,83.85832804155632,83.85832804165632,83.93622093030022,83.93622093040022,84.01372590625157,84.01372590635157,84.09119209092366,84.09119209102366,84.24608566898858,84.24608566908859,84.32320273214738,84.32320273224738,84.40024221274767,84.40024221284767,84.47712652823094,84.47712652833094,84.55385567859719,84.55385567869719,84.70715881421266,84.70715881431266,84.85953095912602,84.85953095922602,84.9352903275109,84.9352903276109,85.01093332205801,85.01093332215801,85.08653752532585,85.08653752542585,85.23747439290678,85.23747439300678,85.31187606651774,85.31187606661774,85.38612257501167,85.38612257511167,85.46033029222635,85.46033029232635,85.53442163560325,85.53442163570325,85.60843539642165,85.60843539652166,85.75642412677921,85.75642412687921,85.83036030503911,85.83036030513911,85.90375340538942,85.90375340548943,86.05046202353157,86.05046202363157,86.12369995876487,86.12369995886488,86.1967439376019,86.1967439377019,86.34275431271745,86.34275431281745,86.4156043351582,86.41560433525821,86.4884155663197,86.4884155664197,86.5607225108509,86.5607225109509,86.6328354989858,86.6328354990858,86.70483211328295,86.70483211338295,86.77663477118382,86.77663477128382,86.84832105524693,86.84832105534693,86.91973580035526,86.91973580045526,86.99099538034655,86.99099538044655,87.06198342138308,87.06198342148308,87.13281629730257,87.13281629740257,87.20298972147474,87.20298972157474,87.27304677180913,87.27304677190914,87.34275470063024,87.34275470073024,87.41191955154179,87.41191955164179,87.48104561117407,87.48104561127407,87.54997771441006,87.54997771451006,87.6187158612498,87.6187158613498,87.75595940725374,87.75595940735374,87.89254350151033,87.89254350161033,87.96054461404422,87.96054461414423,88.02831297890259,88.02831297900259,88.09592617864392,88.09592617874392,88.16338421326824,88.16338421336825,88.23076466533405,88.23076466543405,88.2980287435621,88.2980287436621,88.43244052618043,88.43244052628043,88.49947185673294,88.49947185683294,88.63349572655872,88.63349572665872,88.70044947455273,88.70044947465273,88.76728684870896,88.76728684880896,88.83400784902744,88.83400784912745,88.90061247550815,88.90061247560816,88.96702314559259,88.9670231456926,89.03339502439778,89.03339502449778,89.09968932064444,89.09968932074445,89.16594482561186,89.16594482571186,89.23216153930002,89.23216153940002,89.29833946170893,89.29833946180894,89.36447859283858,89.36447859293858,89.43019101989644,89.43019101999644,89.49539916032397,89.49539916042397,89.56056850947225,89.56056850957225,89.69079083393105,89.69079083403105,89.75576622668305,89.75576622678305,89.88560063834929,89.88560063844929,89.95038207470502,89.95038207480502,90.015047137223,90.015047137323,90.07955703462393,90.07955703472393,90.14391176690786,90.14391176700786,90.20818891663328,90.20818891673328,90.27231090124167,90.27231090134167,90.33627772073305,90.33627772083305,90.40016695766592,90.40016695776592,90.46386223820251,90.46386223830251,90.52747993618058,90.52747993628059,90.59102005160015,90.59102005170016,90.65452137574047,90.65452137584047,90.78140765018335,90.78140765028336,90.84463743536888,90.84463743546888,90.90771205543739,90.90771205553739,90.97074788422665,90.97074788432666,91.03362854789889,91.03362854799889,91.09643162901261,91.09643162911262,91.15904075373007,91.15904075383007,91.221417130772,91.221417130872,91.2836383426969,91.2836383427969,91.34582076334254,91.34582076344255,91.47010802207534,91.47010802217534,91.53178615609067,91.53178615619068,91.59342549882676,91.59342549892676,91.65459934621178,91.65459934631178,91.71565681975903,91.71565681985903,91.7764427543515,91.77644275445151,91.83707352382696,91.83707352392696,91.89758791946466,91.89758791956466,92.01853912818152,92.01853912828152,92.07874319358517,92.07874319368517,92.13883088515105,92.13883088525105,92.19887978543768,92.19887978553768,92.258424399094,92.258424399194,92.37724208745183,92.37724208755183,92.43585571040603,92.43585571050603,92.49427537696396,92.49427537706396,92.55253987840486,92.55253987850486,92.61072679728724,92.61072679738724,92.66875855105262,92.66875855115262,92.72675151353874,92.72675151363875,92.78458931090783,92.78458931100784,92.84238831699768,92.84238831709769,92.95790874661886,92.95790874671886,93.01555258759169,93.01555258769169,93.13072389569956,93.13072389579956,93.1880186151591,93.1880186152591,93.3022201412856,93.3022201413856,93.35908815667332,93.35908815677332,93.41552946798924,93.41552946808925,93.52837329934182,93.52837329944182,93.58473702809924,93.58473702819924,93.64086800918112,93.64086800928112,93.69696019898375,93.69696019908375,93.75289722366935,93.75289722376935,93.8087178745172,93.8087178746172,93.86438336024801,93.86438336034801,93.91969972446554,93.91969972456555,93.97493850612456,93.97493850622456,94.0299833313873,94.0299833314873,94.13995660807502,94.13995660817503,94.19480747694149,94.1948074770415,94.24942559813243,94.24942559823243,94.30396613676486,94.30396613686486,94.35839030155952,94.35839030165953,94.41277567507494,94.41277567517494,94.46673434451854,94.46673434461854,94.52049905756587,94.52049905766587,94.57399223165842,94.57399223175842,94.62721386679618,94.62721386689618,94.68039671065469,94.68039671075469,94.73346318067543,94.73346318077543,94.78629690302064,94.78629690312064,94.8390918340866,94.8390918341866,94.89180918259405,94.89180918269405,94.94433257470521,94.94433257480522,94.99670080169936,94.99670080179936,95.048991446135,95.04899144623501,95.10112692545364,95.10112692555364,95.20535909281163,95.20535909291164,95.25733940701323,95.25733940711324,95.30916455609781,95.30916455619781,95.36091212262389,95.36091212272389,95.46413571672126,95.46413571682126,95.61852500815587,95.61852500825587,95.66976828805163,95.66976828815163,95.77217726528464,95.77217726538464,95.8229938411086,95.8229938412086,95.92431666252247,95.92431666262247,95.97478411683312,95.97478411693312,96.02517398858528,96.02517398868528,96.07544748649967,96.07544748659967,96.1256821931348,96.1256821932348,96.17580052593216,96.17580052603216,96.27599840024764,96.27599840034765,96.3760411094461,96.37604110954611,96.42592669456793,96.42592669466794,96.47557953201425,96.47557953211425,96.52507720434353,96.52507720444353,96.5744197115558,96.5744197116558,96.62364584493031,96.62364584503031,96.67275560446704,96.67275560456704,96.72155503376975,96.72155503386975,96.77019929795543,96.77019929805543,96.81864960574484,96.81864960584484,96.86698353969648,96.86698353979648,96.91512351725184,96.91512351735184,96.96295316457316,96.96295316467317,97.01074402061523,97.01074402071524,97.05837971154028,97.05837971164028,97.10586023734832,97.10586023744833,97.1533019718771,97.1533019719771,97.2006273325681,97.2006273326681,97.24791390197987,97.24791390207987,97.38973481893589,97.3897348190359,97.43686622323062,97.43686622333063,97.48368729729133,97.48368729739133,97.53043078879351,97.53043078889351,97.57709669773719,97.57709669783719,97.67007939411126,97.67007939421126,97.71635739026237,97.71635739036238,97.76228626490021,97.76228626500021,97.8081763482588,97.8081763483588,97.85402764033812,97.85402764043812,97.94557505937975,97.94557505947975,97.99119360378354,97.99119360388354,98.03657940051181,98.03657940061181,98.08192640596083,98.08192640606083,98.17246525174183,98.17246525184183,98.21761830079456,98.21761830089456,98.262422228334,98.262422228434,98.30617879133356,98.30617879143357,98.34985777177461,98.34985777187461,98.43702177626044,98.43702177636044,98.48039042646745,98.48039042656745,98.5237202853952,98.5237202854952,98.5670113530437,98.5670113531437,98.61018604685444,98.61018604695444,98.69630268680038,98.69630268690038,98.78183745755749,98.78183745765749,98.82435269962089,98.82435269972089,98.86675156784653,98.86675156794654,98.90911164479292,98.90911164489292,98.99375421612716,98.99375421622716,99.03599791923578,99.03599791933578,99.07816403978589,99.07816403988589,99.12013620393972,99.12013620403972,99.16175924658026,99.16175924668026,99.20314954154527,99.20314954164527,99.24450104523102,99.24450104533102,99.28573617507901,99.28573617517901,99.36801247837872,99.36801247847872,99.40897606927192,99.40897606937192,99.49059292082428,99.49059292092429,99.57166669446708,99.57166669456709,99.61197083361296,99.61197083371296,99.69242394678767,99.69242394688767,99.77264431228687,99.77264431238687,99.81259932991944,99.81259933001944,99.85220522603872,99.85220522613872,99.8917335395995,99.8917335396995,99.93110668804324,99.93110668814325,99.97044104520775,99.97044104530775,100.04907096825748,100.04907096835748,100.0882113690257,100.0882113691257,100.12731297851468,100.12731297861468,100.16629821416588,100.16629821426588,100.2050894934208,100.2050894935208,100.24384198139647,100.24384198149647,100.28255567809289,100.28255567819289,100.32099783583453,100.32099783593453,100.35936241101766,100.35936241111766,100.39768819492153,100.39768819502153,100.43582002242911,100.43582002252911,100.51204488616506,100.51204488626506,100.55006033983489,100.55006033993489,100.5879982109462,100.5879982110462,100.62585849949902,100.62585849959902,100.6635636229348,100.6635636230348,100.70115237253283,100.70115237263283,100.7387023308516,100.7387023309516,100.7762134978911,100.77621349799111,100.81356949981361,100.81356949991361,100.85088671045685,100.85088671055685,100.88785479958679,100.88785479968679,100.92462893232047,100.92462893242048,100.96136427377489,100.96136427387489,101.03479616540446,101.03479616550446,101.10811168319627,101.10811168329627,101.14461427697516,101.14461427707516,101.1810780794748,101.1810780795748,101.2538893106363,101.2538893107363,101.2901203654604,101.2901203655604,101.32631262900524,101.32631262910525,101.36246610127084,101.36246610137084,101.39854199097792,101.39854199107792,101.43450150684724,101.43450150694724,101.4703446488788,101.4703446489788,101.50603262579332,101.50603262589333,101.54164302014935,101.54164302024935,101.57721462322613,101.57721462332613,101.61266985246513,101.61266985256513,101.68338635454685,101.68338635464686,101.71860883611033,101.71860883621034,101.75379252639456,101.75379252649456,101.78889863412027,101.78889863422027,101.82388836800821,101.82388836810821,101.8588393106169,101.8588393107169,101.89375146194634,101.89375146204634,101.92862482199652,101.92862482209652,101.96345939076744,101.96345939086744,101.99802242058358,101.99802242068358,102.03239149400345,102.03239149410345,102.0666054023063,102.0666054024063,102.10078051932989,102.10078051942989,102.13480047123646,102.13480047133646,102.16878163186378,102.16878163196378,102.20249125353631,102.20249125363631,102.23616208392959,102.23616208402959,102.30346495343689,102.30346495353689,102.37065144910642,102.37065144920642,102.40412832310342,102.40412832320342,102.43748882326267,102.43748882336267,102.47061657574638,102.47061657584638,102.50362795439231,102.50362795449232,102.536600541759,102.536600541859,102.60246813393387,102.60246813403387,102.70111435707916,102.70111435717916,102.73389298804958,102.73389298814958,102.76651645390297,102.76651645400297,102.7991011284771,102.7991011285771,102.83164701177199,102.831647011872,102.86403772994986,102.86403773004986,102.8962732830107,102.8962732831107,102.92847004479229,102.9284700448923,102.96043405889836,102.96043405899836,102.99235928172516,102.99235928182516,103.02420692199345,103.02420692209346,103.0560157709825,103.0560157710825,103.08770824613377,103.08770824623377,103.11916797360952,103.11916797370952,103.150588909806,103.150588909906,103.18197105472325,103.18197105482325,103.21331440836123,103.21331440846123,103.27592353307868,103.27592353317868,103.3071505128789,103.30715051297891,103.36941051608306,103.36941051618307,103.43132139777393,103.43132139787393,103.46188892582681,103.46188892592681,103.49241766260043,103.49241766270043,103.52286881681556,103.52286881691556,103.55316480591365,103.55316480601365,103.6440139819287,103.6440139820287,103.67419359718903,103.67419359728903,103.70414046477383,103.70414046487383,103.73400974980012,103.73400974990012,103.76376266098866,103.76376266108866,103.79328282450166,103.79328282460166,103.82272540545615,103.82272540555616,103.85205161257288,103.85205161267288,103.88126144585183,103.88126144595184,103.91043248785154,103.91043248795154,103.93948715601348,103.93948715611349,103.96842545033766,103.96842545043766,103.99728616210332,103.99728616220332,104.02603050003123,104.02603050013123,104.05461967284211,104.05461967294211,104.08313126309449,104.08313126319449,104.1116040620676,104.1116040621676,104.14003806976146,104.14003806986146,104.19686729386993,104.19686729396993,104.22514613644677,104.22514613654677,104.28154865648344,104.28154865658344,104.309633542664,104.309633542764,104.33764084628606,104.33764084638607,104.36557056734961,104.36557056744961,104.39346149713391,104.39346149723391,104.42131363563894,104.42131363573894,104.44904940030622,104.44904940040622,104.47655241729797,104.47655241739797,104.50390026917269,104.50390026927269,104.53097658209262,104.53097658219262,104.5579365211748,104.5579365212748,104.58485766897772,104.58485766907772,104.61174002550139,104.61174002560139,104.6385835907458,104.6385835908458,104.66538836471095,104.66538836481095,104.69203797355908,104.69203797365908,104.74518202613834,104.74518202623834,104.77159888731094,104.77159888741095,104.82431623581839,104.82431623591839,104.85022881036069,104.85022881046069,104.87610259362373,104.87610259372373,104.90174362921123,104.90174362931123,104.92734587351949,104.92734587361949,104.95287053526923,104.95287053536923,104.9782788231812,104.9782788232812,105.00364831981392,105.00364831991392,105.02897902516739,105.02897902526739,105.05411577412458,105.05411577422458,105.07917494052327,105.07917494062328,105.12921569076212,105.12921569086213,105.20412165100339,105.20412165110339,105.22898686100581,105.22898686110581,105.30342732589601,105.30342732599601,105.32805978822289,105.32805978832289,105.35265345927051,105.35265345937052,105.37716954775964,105.37716954785964,105.40160805369024,105.40160805379024,105.42600776834158,105.42600776844158,105.45036869171368,105.45036869181368,105.47438049357248,105.47438049367248,105.498198339035,105.498198339135,105.52193860193903,105.52193860203903,105.5693803364678,105.5693803365678,105.5930430168133,105.5930430169133,105.61658932332105,105.61658932342105,105.64005804727027,105.64005804737027,105.66337160610249,105.66337160620249,105.7099211412084,105.7099211413084,105.73296316108583,105.73296316118584,105.75585001584624,105.75585001594624,105.77858170548963,105.77858170558963,105.82400629349715,105.82400629359715,105.84662160930277,105.84662160940277,105.86904296871212,105.86904296881212,105.8914255368422,105.89142553694221,105.93607429926463,105.93607429936463,105.95830170227771,105.95830170237771,105.98041273145301,105.98041273155302,106.00248496934907,106.00248496944907,106.02436325084885,106.02436325094885,106.04620274106938,106.04620274116938,106.06800344001064,106.06800344011064,106.11148846405541,106.11148846415541,106.13309520660042,106.13309520670042,106.15458557530765,106.15458557540765,106.17603715273562,106.17603715283562,106.19744993888433,106.19744993898433,106.21882393375381,106.21882393385381,106.23992638966848,106.23992638976848,106.26099005430392,106.26099005440392,106.28193734510157,106.28193734520157,106.30276826206146,106.30276826216146,106.32344401390435,106.32344401400435,106.36467914375234,106.36467914385234,106.38519973047819,106.3851997305782,106.40544877824927,106.40544877834927,106.42546507834481,106.42546507844482,106.48543639607293,106.48543639617293,106.52527503986774,106.52527503996774,106.56499730982479,106.56499730992479,106.5847808622448,106.5847808623448,106.60452562338556,106.60452562348556,106.62419280196781,106.62419280206781,106.64354965031602,106.64354965041602,106.66286770738496,106.66286770748496,106.68210818189542,106.68210818199542,106.72051154835779,106.7205115484578,106.73963564903048,106.73963564913048,106.75868216714464,106.75868216724464,106.77768989397956,106.77768989407956,106.83467428320505,106.83467428330505,106.87253457175785,106.87253457185786,106.89138713347575,106.89138713357575,106.91012332135588,106.91012332145588,106.9287819266775,106.9287819267775,106.94736294944062,106.94736294954062,106.96590518092447,106.96590518102447,106.98425345601204,106.98425345611204,107.0208724236287,107.0208724237287,107.03906553359926,107.03906553369926,107.05721985229057,107.05721985239057,107.07510263202708,107.07510263212708,107.09286903792585,107.09286903802585,107.11055786126609,107.11055786136609,107.14589671666734,107.14589671676734,107.16343037489057,107.16343037499057,107.18080886799677,107.18080886809678,107.21552706292992,107.21552706302992,107.23282797347763,107.23282797357763,107.24989613634979,107.24989613644979,107.2669255079427,107.2669255080427,107.30094545984927,107.30094545994928,107.31781966632518,107.31781966642518,107.33461629024256,107.33461629034257,107.35137412288069,107.35137412298069,107.3680543729603,107.3680543730603,107.38469583176067,107.38469583186067,107.41786237552364,107.41786237562364,107.43423229536921,107.43423229546922,107.45048584137703,107.45048584147703,107.48283776827564,107.48283776837565,107.49885856660792,107.49885856670792,107.53086137199324,107.53086137209324,107.54680458776701,107.54680458786702,107.5785746454768,107.57857464557681,107.64184322194158,107.64184322204159,107.67326415813808,107.67326415823808,107.70445234665904,107.70445234675904,107.71996885836101,107.71996885846102,107.75092429920645,107.75092429930645,107.78172457493486,107.78172457503486,107.79704713024056,107.79704713034056,107.8276534495727,107.8276534496727,107.84289842231988,107.84289842241988,107.87327199397649,107.87327199407649,107.88836180160666,107.88836180170667,107.91850262558773,107.91850262568774,107.9485658670103,107.9485658671103,107.96350050952346,107.96350050962346,107.97827998691957,107.97827998701958,107.99302067303645,107.99302067313646,108.02246325399095,108.02246325409095,108.03712635754931,108.03712635764931,108.05175066982841,108.05175066992841,108.08088292054886,108.08088292064886,108.09535206771095,108.09535206781095,108.10974363231453,108.10974363241453,108.13837159640467,108.13837159650467,108.15253041333271,108.15253041343271,108.1807704646303,108.1807704647303,108.22297537645967,108.22297537655967,108.2369790282707,108.2369790283707,108.2649475406135,108.26494754071351,108.27887360986603,108.27887360996603,108.29264451400152,108.29264451410153,108.3063378355785,108.3063378356785,108.3197596182007,108.31975961830071,108.33314260954366,108.33314260964366,108.34640922704885,108.34640922714885,108.3727872969422,108.37278729704221,108.38566600165484,108.38566600175484,108.3984283325297,108.3984283326297,108.41099670700831,108.41099670710831,108.42352629020765,108.42352629030765,108.43597829084848,108.43597829094848,108.44839150021005,108.44839150031005,108.46068833573386,108.46068833583386,108.49754005102605,108.49754005112605,108.5097205127121,108.5097205128121,108.52182339183963,108.52182339193963,108.53388747968792,108.53388747978792,108.55797686410523,108.55797686420523,108.56992457811575,108.56992457821575,108.58179470956776,108.58179470966776,108.59362604974051,108.59362604984051,108.60526343351698,108.60526343361698,108.64009800228791,108.64009800238792,108.65161901222662,108.65161901232662,108.66310123088608,108.66310123098609,108.67454465826629,108.67454465836629,108.68591050308798,108.68591050318798,108.69723755663041,108.69723755673041,108.70844823633509,108.70844823643509,108.74204148416986,108.74204148426986,108.75317458131603,108.75317458141603,108.76419130462442,108.76419130472442,108.78618595996197,108.78618596006197,108.7970863094326,108.7970863095326,108.82974856656526,108.82974856666526,108.84057133347738,108.84057133357739,108.85135530911025,108.85135530921025,108.8620617021846,108.8620617022846,108.87272930397971,108.87272930407971,108.91536091988088,108.91536091998088,108.92595093911747,108.92595093921747,108.94697581247365,108.94697581257365,108.95733308403472,108.95733308413472,108.97800883587759,108.97800883597759,108.99860700516196,108.99860700526196,109.008809111606,109.008809111706,109.01893363549154,109.01893363559154,109.02901936809782,109.02901936819782,109.05923777463741,109.05923777473741,109.07929286601221,109.07929286611221,109.11928667492404,109.11928667502404,109.13914780990255,109.13914781000256,109.15889257104332,109.15889257114333,109.16870676469482,109.16870676479482,109.19803297181154,109.19803297191154,109.22708763997348,109.22708764007348,109.24632811448393,109.24632811458393,109.2654522151566,109.2654522152566,109.27495607857406,109.27495607867407,109.28442115071226,109.28442115081226,109.30331250370942,109.30331250380942,109.31269999328912,109.31269999338912,109.33139738988999,109.33139738998999,109.34066850563191,109.34066850573191,109.34990083009458,109.34990083019458,109.36832668774068,109.36832668784068,109.38667496282825,109.38667496292825,109.3957521221739,109.3957521222739,109.4047904902403,109.4047904903403,109.43186680316025,109.43186680326025,109.46782631902957,109.46782631912957,109.49466988427397,109.49466988437398,109.50355308722335,109.50355308732335,109.53016390479223,109.53016390489223,109.53893073390385,109.53893073400386,109.55642560084782,109.55642560094782,109.57384288523329,109.57384288533329,109.59118258706023,109.59118258716023,109.60844470632868,109.60844470642868,109.61701757904402,109.61701757914402,109.62551286920085,109.62551286930085,109.64246465823526,109.64246465833526,109.65088236583357,109.65088236593357,109.65926128215264,109.65926128225264,109.68435923983057,109.68435923993057,109.69266057359113,109.69266057369113,109.70092311607243,109.70092311617243,109.71740940975577,109.71740940985578,109.72559436967856,109.72559436977856,109.75011045816768,109.75011045826768,109.77451017281903,109.77451017291904,109.78257875890405,109.78257875900405,109.79867713979485,109.79867713989485,109.81469793812714,109.81469793822714,109.83064115390091,109.83064115400092,109.85445899936343,109.85445899946343,109.8702470500202,109.8702470501202,109.90170677749593,109.90170677759593,109.90950382462619,109.90950382472619,109.91726208047716,109.91726208057716,109.93273980089988,109.93273980099988,109.94813993876409,109.94813993886409,109.97114316736226,109.97114316746226,110.00931378614911,110.00931378624911,110.02448117633779,110.02448117643779,110.03957098396795,110.03957098406795,110.08468524174144,110.08468524184144,110.09961988425458,110.09961988435458,110.11447694420922,110.11447694430922,110.12925642160535,110.12925642170535,110.14395831644296,110.14395831654296,110.15125107694288,110.15125107704289,110.18034453638408,110.18034453648409,110.19473610098767,110.19473610108767,110.20905008303274,110.20905008313274,110.25897445943383,110.25897445953383,110.27313327636188,110.27313327646188,110.3083945492046,110.30839454930461,110.35750430874134,110.35750430884134,110.37841280825975,110.37841280835976,110.38531765596713,110.38531765606713,110.41980310322475,110.41980310332475,110.44722853765799,110.447228537758,110.46767154182534,110.46767154192534,110.47442122441569,110.4744212245157,110.49463148090751,110.49463148100752,110.54156892880597,110.54156892890597,110.56821853765412,110.56821853775412,110.60134629013783,110.60134629023783,110.66725267359195,110.66725267369195,110.6999925132831,110.69999251338311,110.74557226640765,110.74557226650765,110.7908804805774,110.7908804806774,110.83591715579237,110.83591715589237,110.8742817309755,110.8742817310755,110.89332824908966,110.89332824918966,110.96916520003305,110.96916520013305,111.00054734495029,111.0005473450503,111.0379809294313,111.0379809295313,111.0565619521944,111.0565619522944,111.08119441452128,111.08119441462128,111.13631682234254,111.13631682244254,111.23981195539469,111.23981195549469,111.33659619713573,111.33659619723574,111.4387724266932,111.43877242679321,111.5343153474981,111.5343153475981,111.67671813364294,111.67671813374294,111.74743463572467,111.74743463582467,111.85868802462781,111.85868802472781,111.9401109197839,111.9401109198839,112.02099073703043,112.02099073713043,112.10132747636739,112.10132747646739,112.22103736414809,112.22103736424809,112.33426910829323,112.33426910839323,112.41860134939344,112.41860134949344,112.51910955394295,112.51910955404296,112.57454229199826,112.57454229209826,112.6901790954572,112.6901790955572,112.77765343017705,112.77765343027706,112.91881489538574,112.91881489548574,113.00504790916945,113.00504790926945,113.11207304863379,113.11207304873379,113.26083760457644,113.26083760467644,113.37686232082793,113.37686232092793,113.50774409703406,113.50774409713406,113.60126987131768,113.60126987141768,113.69409740257473,113.69409740267473,113.77086534422024,113.77086534432024,113.85721473184171,113.85721473194171,113.95803326662526,113.95803326672527,114.07809227591926,114.07809227601926,114.18728972702189,114.1872897271219,114.2562218302579,114.2562218303579,114.3637124650733,114.36371246517331,114.45095405211764,114.45095405221764,114.52787715888016,114.52787715898016,114.68529217009666,114.68529217019666,114.79410170840673,114.79410170850673,114.93018151603302,114.93018151613302,115.05117151602914,115.05117151612914,115.16653678053329,115.1665367806333,115.276354892104,115.276354892204,115.39431917231823,115.39431917241824,115.54727318642041,115.54727318652041,115.68106430857067,115.68106430867067,115.8313805156835,115.83138051578351,116.00667830663653,116.00667830673653,116.17173519986625,116.17173519996625,116.39129384044914,116.39129384054914,116.61740820722612,116.61740820732612,116.87529263171288,116.87529263181288,117.16851591160088,117.16851591170088,117.55448914018741,117.55448914028742,118.06843479903596,118.06843479913596,118.67528557170006,118.67528557180006,119.47000250979576,119.47000250989576,120.49281216991047,120.49281217001047,122.20207230772036,122.20207230782036]],"filenames":["genome.ctg.filtered"]}

{"coord\_y":[[2981,2981,2981,1622,1622,197,197,194,194,180,180,170,170,164,164,158,158,157,157,156,156,155,155,153,153,152,152,145,145,142,142,140,140,139,139,138,138,137,137,135,135,134,134,131,131,130,130,129,129,128,128,127,127,126,126,125,125,122,122,121,121,119,119,118,118,117,117,116,116,114,114,113,113,112,112,111,111,110,110,109,109,108,108,107,107,106,106,105,105,104,104,103,103,102,102,101,101,100,100,99,99,98,98,97,97,96,96,93,93,92,92,88,88,87,87,86,86,85,85,84,84,83,83,82,82,81,81,79,79,78,78,72,72,71,71,67,67,65,65,0.0]],"coord\_x":[[0.0,1e-10,0.08645353448788388,0.08645353458788388,0.13349400176039233,0.13349400186039234,0.13920730142295965,0.13920730152295965,0.1448335965221376,0.1448335966221376,0.15005387032549858,0.1500538704254986,0.15498412891756172,0.15498412901756173,0.1597403783828462,0.1597403784828462,0.16432261872135193,0.16432261882135193,0.1688758575387279,0.16887585763872792,0.17795333365235005,0.17795333375235006,0.18244856942746648,0.1824485695274665,0.1913810379354397,0.1913810380354397,0.19578926914716677,0.19578926924716678,0.19999448971098535,0.19999448981098536,0.20411270571141457,0.20411270581141458,0.2081729186695842,0.20817291876958421,0.2122041301066241,0.2122041302066241,0.22023755145957405,0.22023755155957406,0.22821296977026445,0.22821296987026446,0.2321281751227852,0.2321281752227852,0.23601437895417615,0.23601437905417616,0.23981357822217775,0.23981357832217776,0.2473829752370512,0.2473829753370512,0.2511241714627932,0.2511241715627932,0.25483636616740546,0.25483636626740547,0.26594394876011246,0.26594394886011247,0.26959814042246516,0.26959814052246517,0.2732233305636881,0.2732233306636881,0.2876370865651903,0.28763708666519033,0.29114627062189413,0.29114627072189414,0.2945974516363383,0.29459745173633833,0.30147081214409693,0.30147081224409694,0.31170834910291045,0.31170834920291046,0.32864523744270385,0.32864523754270386,0.35550064600888315,0.35550064610888316,0.36539016471413927,0.3653901648141393,0.375192678856006,0.375192678956006,0.38166001806794764,0.38166001816794765,0.3848501853922238,0.38485018549222383,0.39120151851964635,0.39120151861964636,0.4038171802111021,0.4038171803111021,0.41318467153602206,0.41318467163602207,0.43177464658021314,0.43177464668021315,0.4624872574566536,0.4624872575566536,0.48072921424728726,0.48072921434728727,0.5048294783061371,0.5048294784061371,0.5227234168432134,0.5227234169432134,0.5552341220297005,0.5552341221297005,0.6460088831659221,0.6460088832659221,0.6633807943226622,0.6633807944226622,0.6748363951689266,0.6748363952689266,0.6804916917892343,0.6804916918892343,0.6860889853672825,0.6860889854672825,0.6915702728608115,0.6915702729608115,0.6969355542698215,0.6969355543698215,0.6994876881292423,0.6994876882292423,0.7045629543269545,0.7045629544269545,0.709580217482407,0.709580217582407,0.7120453467784386,0.7120453468784386,0.7144814745533403,0.7144814746533403,0.7193247285820141,0.7193247286820141,0.7217028533146563,0.7217028534146563,0.726430101258811,0.726430101358811,0.728721221428064,0.728721221528064,0.7332744602454399,0.7332744603454399,0.7376246884149074,0.7376246885149074,0.7396837964151219,0.739683796515122,0.7416268983308174,0.7416268984308174,0.7454550991199489,0.7454550992199489]],"filenames":["genome.ctg.filtered"]}

{"coord\_y":[[2981,2981,2981,1622,1622,197,197,194,194,180,180,170,170,164,164,158,158,157,157,156,156,155,155,153,153,152,152,145,145,142,142,140,140,139,139,138,138,137,137,135,135,134,134,131,131,130,130,129,129,128,128,127,127,126,126,125,125,122,122,121,121,119,119,118,118,117,117,116,116,114,114,113,113,112,112,111,111,110,110,109,109,108,108,107,107,106,106,105,105,104,104,103,103,102,102,101,101,100,100,99,99,98,98,97,97,96,96,93,93,92,92,88,88,87,87,86,86,85,85,84,84,83,83,82,82,81,81,79,79,78,78,72,72,71,71,67,67,65,65,0.0]],"coord\_x":[[0.0,1e-10,0.11563680345894078,0.11563680355894078,0.17855625841043424,0.17855625851043425,0.18619814042365507,0.18619814052365508,0.19372364859911115,0.19372364869911116,0.2007060788649982,0.2007060789649982,0.20730059633833597,0.20730059643833598,0.2136623661361442,0.2136623662361442,0.21979138825842284,0.21979138835842285,0.22588161910144658,0.2258816192014466,0.23802328950823906,0.23802328960823907,0.24403593779275293,0.24403593789275294,0.2559836518032708,0.2559836519032708,0.2618799262500199,0.2618799263500199,0.26750466174198445,0.26750466184198446,0.2730130233961843,0.2730130234961843,0.27844380249187417,0.2784438025918742,0.2838357903083092,0.2838357904083092,0.2945809746619243,0.2945809747619243,0.3052485764570295,0.3052485765570295,0.31048539915644485,0.31048539925644486,0.3156834305766052,0.3156834306766052,0.3207650881590008,0.3207650882590008,0.33088961204453704,0.33088961214453705,0.33589368706842276,0.33589368716842277,0.34085897081305355,0.34085897091305356,0.35571603076769104,0.35571603086769105,0.360603731953812,0.360603732053812,0.365452641860678,0.365452641960678,0.3847319076503773,0.3847319077503773,0.3894256524402236,0.3894256525402236,0.3940418146715601,0.3940418147715601,0.403235347854978,0.403235347954978,0.41692866943196766,0.41692866953196767,0.4395827765168457,0.4395827766168457,0.47550350110690914,0.47550350120690915,0.48873132733283964,0.48873132743283965,0.5018427797210053,0.5018427798210053,0.5104932349948543,0.5104932350948543,0.5147602757128964,0.5147602758128964,0.5232555658697257,0.5232555659697257,0.5401297723456194,0.5401297724456194,0.5526593555449613,0.5526593556449613,0.5775245655473702,0.5775245656473702,0.6186045302783391,0.6186045303783391,0.6430042449296889,0.6430042450296889,0.6752397979905341,0.6752397980905341,0.6991740172908248,0.6991740173908249,0.7426590413355992,0.7426590414355992,0.8640757454035244,0.8640757455035244,0.8873117216772263,0.8873117217772263,0.9026342769829229,0.9026342770829229,0.9101985764376339,0.9101985765376339,0.9176852933338351,0.9176852934338351,0.9250168451130164,0.9250168452130164,0.9321932317751782,0.9321932318751782,0.9356068643496118,0.9356068644496118,0.9423953382192243,0.9423953383192243,0.9491062295303269,0.9491062296303269,0.9524034882669957,0.9524034883669957,0.9556619557244097,0.9556619558244097,0.9621400993599827,0.9621400994599827,0.9653209842588868,0.9653209843588868,0.97164396277744,0.97164396287744,0.9747084738385794,0.9747084739385794,0.9807987046816031,0.9807987047816031,0.9866173965698424,0.9866173966698424,0.9893715773969423,0.9893715774969423,0.9919705931070224,0.9919705932070224,0.9970910419686729,0.9970910420686729]],"filenames":["genome.ctg.filtered"]}

{"coord\_y":[[0.9970910419686729,0.9970910419686729]],"coord\_x":[[0,1]],"filenames":["genome.ctg.filtered"]}

{{ genesInContigs }}

{{ operonsInContigs }}

[{{ num\_contigs }},
{{ Largest\_alignment }},
{{ Total\_aligned\_length }},
{{ num\_misassemblies }},
{{ Misassembled\_contigs\_length }},
{{ num\_mismatches\_per\_100\_kbp }},
{{ num\_indels\_per\_100\_kbp }},
{{ num\_N's\_per\_100\_kbp }},
{{ Genome\_fraction }},
{{ Duplication\_ratio }},
{{ NGA50 }}]

{{ allMisassemblies }}

{{ krona }}

{"list\_of\_GC\_distributions":[[[0.0,1.0,2.0,3.0,4.0,5.0,6.0,7.0,8.0,9.0,10.0,11.0,12.0,13.0,14.0,15.0,16.0,17.0,18.0,19.0,20.0,21.0,22.0,23.0,24.0,25.0,26.0,27.0,28.0,29.0,30.0,31.0,32.0,33.0,34.0,35.0,36.0,37.0,38.0,39.0,40.0,41.0,42.0,43.0,44.0,45.0,46.0,47.0,48.0,49.0,50.0,51.0,52.0,53.0,54.0,55.0,56.0,57.0,58.0,59.0,60.0,61.0,62.0,63.0,64.0,65.0,66.0,67.0,68.0,69.0,70.0,71.0,72.0,73.0,74.0,75.0,76.0,77.0,78.0,79.0,80.0,81.0,82.0,83.0,84.0,85.0,86.0,87.0,88.0,89.0,90.0,91.0,92.0,93.0,94.0,95.0,96.0,97.0,98.0,99.0,100.0],[1,0,0,0,0,0,0,0,0,1,1,0,8,22,32,51,43,63,119,169,291,395,522,707,897,1177,1399,1614,1944,2067,2388,2393,2227,2223,2085,2027,1823,1634,1410,1098,896,745,499,361,300,182,124,90,66,44,45,24,4,8,12,4,3,1,3,2,1,1,0,0,0,0,0,0,0,0,0,0,0,0,0,0,0,0,0,0,0,0,0,0,0,0,0,0,0,0,0,0,0,0,0,1,0,0,0,1,0]],[[0.0,1.0,2.0,3.0,4.0,5.0,6.0,7.0,8.0,9.0,10.0,11.0,12.0,13.0,14.0,15.0,16.0,17.0,18.0,19.0,20.0,21.0,22.0,23.0,24.0,25.0,26.0,27.0,28.0,29.0,30.0,31.0,32.0,33.0,34.0,35.0,36.0,37.0,38.0,39.0,40.0,41.0,42.0,43.0,44.0,45.0,46.0,47.0,48.0,49.0,50.0,51.0,52.0,53.0,54.0,55.0,56.0,57.0,58.0,59.0,60.0,61.0,62.0,63.0,64.0,65.0,66.0,67.0,68.0,69.0,70.0,71.0,72.0,73.0,74.0,75.0,76.0,77.0,78.0,79.0,80.0,81.0,82.0,83.0,84.0,85.0,86.0,87.0,88.0,89.0,90.0,91.0,92.0,93.0,94.0,95.0,96.0,97.0,98.0,99.0,100.0],[0,0,0,0,0,0,0,0,0,0,1,1,2,6,11,20,28,61,91,132,189,260,324,408,542,690,816,1002,1117,1298,1487,1625,1703,1648,1704,1652,1425,1409,1271,1074,875,720,557,428,325,232,146,101,80,67,72,44,40,23,22,16,11,6,6,3,7,2,0,0,0,0,0,0,0,0,0,0,0,0,0,0,0,0,0,0,0,0,0,0,0,0,0,0,0,0,0,0,0,0,0,0,0,0,0,0,0]]],"reference\_index":1,"list\_of\_GC\_contigs\_distributions":[[[0,5,10,15,20,25,30,35,40,45,50,55,60,65,70,75,80,85,90,95,100],[1,0,53,116,740,2560,2409,717,165,85,5,2,0,0,0,0,0,0,0,1,0]]],"lists\_of\_gc\_info":null,"filenames":["genome.ctg.filtered"]}

{"links\_names":["View in Icarus contig browser"],"links":["icarus\_viewers/alignment\_viewer.html"]}

{
"# contigs" : "is the total number of contigs in the assembly.",
"Largest contig" : "is the length of the longest contig in the assembly.",
"Total length" : "is the total number of bases in the assembly.",
"Reference length" : "is the total number of bases in the reference.",
"# contigs (>= 0 bp)" : "is the total number of contigs in the assembly that have size greater than or equal to 0 bp.",
"Total length (>= 0 bp)" : "is the total number of bases in the contigs having size greater than or equal to 0 bp.",
"N50" : "is the contig length such that using longer or equal length contigs produces half (50%) of the bases of the assembly. Usually there is no value that produces exactly 50%, so the technical definition is the maximum length x such that using contigs of length at least x accounts for at least 50% of the total assembly length.",
"NG50" : "is the contig length such that using longer or equal length contigs produces half (50%) of the bases of the reference genome. This metric is computed only if a reference genome is provided.",
"N75" : "is the contig length such that using longer or equal length contigs produces 75% of the bases of the assembly. Usually there is no value that produces exactly 75%, so the technical definition is the maximum length x such that using contigs of length at least x accounts for at least 75% of the total assembly length.",
"NG75" : "is the contig length such that using longer or equal length contigs produces 75% of the bases of the reference genome. This metric is computed only if a reference genome is provided.",
"L50" : "is the minimum number of contigs that produce half (50%) of the bases of the assembly. In other words, it's the number of contigs of length at least N50.",
"LG50" : "is the minimum number of contigs that produce half (50%) of the bases of the reference genome. In other words, it's the number of contigs of length at least NG50. This metric is computed only if a reference genome is provided.",
"L75" : "is the minimum number of contigs that produce 75% of the bases of the assembly. In other words, it's the number of contigs of length at least N75.",
"LG75" : "is the minimum number of contigs that produce 75% of the bases of the reference genome. In other words, it's the number of contigs of length at least NG75. This metric is computed only if a reference genome is provided.",
"NA50" : "is N50 where the lengths of aligned blocks are counted instead of contig lengths. I.e., if a contig has a misassembly with respect to the reference, the contig is broken into smaller pieces. This metric is computed only if a reference genome is provided.",
"NGA50" : "is NG50 where the lengths of aligned blocks are counted instead of contig lengths. I.e., if a contig has a misassembly with respect to the reference, the contig is broken into smaller pieces. This metric is computed only if a reference genome is provided.",
"NA75" : "is N75 where the lengths of aligned blocks are counted instead of contig lengths. I.e., if a contig has a misassembly with respect to the reference, the contig is broken into smaller pieces. This metric is computed only if a reference genome is provided.",
"NGA75" : "is NG75 where the lengths of aligned blocks are counted instead of contig lengths. I.e., if a contig has a misassembly with respect to the reference, the contig is broken into smaller pieces. This metric is computed only if a reference genome is provided.",
"LA50" : "is L50 where aligned blocks are counted instead of contigs. I.e., if a contig has a misassembly with respect to the reference, the contig is broken into smaller pieces.",
"LGA50" : "is LG50 where aligned blocks are counted instead of contigs. I.e., if a contig has a misassembly with respect to the reference, the contig is broken into smaller pieces.",
"LA75" : "is L75 where aligned blocks are counted instead of contigs. I.e., if a contig has a misassembly with respect to the reference, the contig is broken into smaller pieces.",
"LGA75" : "is LG75 where aligned blocks are counted instead of contigs. I.e., if a contig has a misassembly with respect to the reference, the contig is broken into smaller pieces.",
"Average %IDY" : "is the average of alignment identity percent (Nucmer measure of alignment accuracy) among all contigs.",
"# misassemblies" : "is the number of positions in the assembled contigs where the left flanking sequence aligns over 1 kbp away from the right flanking sequence on the reference (*relocation*) or they overlap on more than 1 kbp (*relocation*) or flanking sequences align on different strands (*inversion*) or different chromosomes (*translocation*).",
"# misassembled contigs" : "is the number of contigs that contain misassembly events.",
"Misassembled contigs length" : "is the number of total bases contained in all contigs that have one or more misassemblies.",
"# relocations" : "is the number of relocation events among all misassembly events. Relocation is a misassembly where the left flanking sequence aligns over 1 kbp away from the right flanking sequence on the reference, or they overlap by more than 1 kbp and both flanking sequences align on the same chromosome.",
"# translocations" : "is the number of translocation events among all misassembly events. Translocation is a misassembly where the flanking sequences align on different chromosomes.",
"# interspecies translocations" : "is the number of interspecies translocation events among all misassembly events. Interspecies translocation is a misassembly where the flanking sequences align on different references (based on alignments to the combined reference).",
"# inversions" : "is the number of inversion events among all misassembly events. Inversion is a misassembly where it is not a *relocation* and the flanking sequences align on opposite strands of the same chromosome.",
"# local misassemblies" : "is the number of local misassemblies. We define a local misassembly breakpoint as a breakpoint that satisfies these conditions:

1. Two or more distinct alignments cover the breakpoint.
2. The gap between left and right flanking sequences is less than 1 kbp.
3. The left and right flanking sequences both are on the same strand of the same chromosome of the reference genome.
",
"# scaffold gap size misassemblies" : "is the number of scaffold gap size misassemblies. We define scaffold gap size misassembly as a breakpoint where the flanking sequences combined in scaffold on the wrong distance. These misassemblies are not included in the total number of misassemblies. ",
"# possibly misassembled contigs": "is the number of contigs that contain large unaligned fragment (default min length is 500 bp) and thus could possibly contain interspecies translocation with unknown reference.",
"# possible misassemblies" : "is the number of putative interspecies translocations in possibly misassembled contigs if each large unaligned fragment is supposed to be a fragment of unknown reference.",
"# intergenomic misassemblies" : "is the number of all found and putative (possible) interspecies translocations.",
"# structural variations" : "is the number of misassemblies matched with structural variations.",
"# unaligned mis. contigs" : "is the number of contigs that have the number of unaligned bases more than 50% of contig length and a misassembly event in their aligned fragment. Note that such misassemblies are not counted in *# misassemblies* and other *misassemblies* statistics.",
"# fully unaligned contigs" : "is the number of contigs that have no alignment to the reference sequence.",
"Fully unaligned length" : "is the total number of bases contained in all fully unaligned contigs.",
"# partially unaligned contigs" : "is the number of contigs that have at least one alignment to the reference sequence but also have at least one unaligned fragment of length ≥ *unaligned-part-size threshold*.",
"Partially unaligned length" : "is the total number of unaligned bases in all partially unaligned contigs.",
"# ambiguous contigs" : "is the number of contigs that have reference alignments of equal quality in multiple locations on the reference.",
"Ambiguous contigs length" : "is the total number of bases contained in all ambiguous contigs.",
"Genome fraction (%)" : "is the total number of aligned bases in the reference, divided by the genome size. A base in the reference genome is counted as aligned if there is at least one contig with at least one alignment to this base. Contigs from repeat regions may map to multiple places, and thus may be counted multiple times in this quantity.",
"GC (%)" : "is the total number of G and C nucleotides in the assembly, divided by the total length of the assembly.",
"Reference GC (%)" : "is the total number of G and C nucleotides in the reference, divided by the total length of the reference.",
"# mismatches per 100 kbp" : "is the average number of mismatches per 100000 aligned bases.",
"# mismatches" : "is the number of mismatches in all aligned bases.",
"# indels per 100 kbp" : "is the average number of indels per 100000 aligned bases.",
"# indels" : "is the number of indels in all aligned bases",
"# indels (<= 5 bp)" : "is the number of indels of length less than or equal to 5 bp",
"# indels (> 5 bp)" : "is the number of indels of length greater than 5 bp",
"Indels length" : "is the number of total bases contained in all indels",
"# genes" : "is the number of genes in the assembly (complete and partial), based on a user-provided annotated list of gene positions in the reference genome. A gene counts as 'partially covered' if the assembly contains at least 100 bp of this gene but not the whole gene.",
"# operons" : "is the number of operons in the assembly (complete and partial), based on a user-provided annotated list of operon positions in the reference genome. An operon counts as 'partially covered' if the assembly contains at least 100 bp of this operon but not the whole operon.",
"# predicted genes (unique)" : "is the number of unique genes in the assembly found by a gene prediction tool.",
"# predicted genes (>= 0 bp)" : "is the number of found genes having length greater than or equal to 0 bp.",
"Cumulative length" : "plot shows the growth of assembly contig lengths. On the x-axis, contigs are ordered from largest (contig #1) to smallest. The y-axis gives the size of the x largest contigs in the assembly.",
"Nx" : "plot shows the Nx metric value as x varies from 0 to 100. Nx is the minimum contig length **y** such that using contigs of length at least **y** accounts for at least x% of the total assembly length.",
"NGx" : "plot shows the NGx metric value as x varies from 0 to 100. NGx is the minimum contig length **y** such that using contigs of length at least **y** accounts for at least x% of the bases of the reference genome. This metric is computed only if a reference genome is provided.",
"NAx" : "plot shows the NAx metric value as x varies from 0 to 100. NAx is computed similarly to Nx, but based on lengths of aligned blocks instead of contig lengths. Contigs are broken into aligned blocks at misassembly breakpoints. NAx is the minimum block length **y** such that using blocks of length at least **y** accounts for at least x% of the bases of the assembly. This metric is computed only if a reference genome is provided.",
"NGAx" : "plot shows the NGAx metric value as x varies from 0 to 100.NGAx is computed similarly to NGx, but based on lengths of aligned blocks instead of contig lengths. Contigs are broken at misassembly breakpoints. NGAx is the minimum block length **y** such that using blocks of length at least **y** accounts for at least x% of the bases of the reference genome. This metric is computed only if a reference genome is provided.",
"GC content" : "plot shows the distribution of GC percentage among the contigs, i.e., the total number of bases in contigs with such GC content. Typically, the distribution is approximately Gaussian. However, for some genomes it is not Gaussian. For assembly projects with contaminants, the GC distribution of the contaminants often differs from the reference genome and may give a superposition of multiple curves with different peaks.",
"Duplication ratio" : "is the total number of aligned bases in the assembly (i.e. *Total length* - *Fully unaligned length* - *Partially unaligned length*), divided by the total number of aligned bases in the reference (see the **Genome fraction (%)** metric). If the assembly contains many contigs that cover the same regions of the reference, its *Duplication ratio* may be much larger than 1. This may occur due to overestimating repeat multiplicities and due to small overlaps between contigs, among other reasons.",
"Largest alignment" : "is the length of the largest continuous alignment in the assembly. This metric is always equal to the *Largest contig* metric but it can be smaller if the largest contig of the assembly contains a misassembly event.",
"Total aligned length" : "is the total number of aligned bases in the assembly.",
"Avg contig read support" : "is the average coverage of contigs that have large unique alignments to the reference. Read coverage is extracted from contig names (SPAdes/Velvet naming scheme only).",
"# N's" : "is the total number of uncalled bases (N's) in the assembly.",
"# N's per 100 kbp" : "is the average number of uncalled bases (N's) per 100000 assembly bases.",
"# similar correct contigs" : "is the number of correct contigs similar among > 50% assemblies (see Icarus for visualization).",
"# similar misassembled blocks" : "is the number of misassembled blocks similar among > 50% assemblies (see Icarus for visualization)."
}
