# Supplementary material for: HiMMe: using genetic patterns as a proxy for genome assembly reliability assessment
Source: BMC Genomics. 2017 Sep 5;18:694. doi: 10.1186/s12864-017-3965-2 (PMC5584555; doi:10.1186/s12864-017-3965-2)
Supplement: Supplementary file 3 — ’Supplementary_file3.zip’, Title: ’Data from GAGE’. Description: contains all real data as well as the pertinent results. (ZIP 18,698 kb) [file 12864_2017_3965_MOESM3_ESM.zip › Real data/QUAST/lineage/SOAPdenovo/full_output/icarus.html]

|  |
| --- |
| Icarus **QUAST Contig Browser** by CAB |

**Assemblies:** genome.ctg.filtered| Contig size viewer |
| QUAST report |

  

Contig alignment viewer

Aligned to sequences from staphylococcus\_saprophyticus.fa

Fragments: 3, length: 2 577 899 bp, mean genome fraction: 0.254%,
misassembled blocks: 0
