# Supplementary material for: HiMMe: using genetic patterns as a proxy for genome assembly reliability assessment
Source: BMC Genomics. 2017 Sep 5;18:694. doi: 10.1186/s12864-017-3965-2 (PMC5584555; doi:10.1186/s12864-017-3965-2)
Supplement: Supplementary file 3 — ’Supplementary_file3.zip’, Title: ’Data from GAGE’. Description: contains all real data as well as the pertinent results. (ZIP 18,698 kb) [file 12864_2017_3965_MOESM3_ESM.zip › Real data/QUAST/exact/Bambus2/full_output/icarus_viewers/alignment_viewer.html]

|  |  |  |
| --- | --- | --- |
| Main menu  Icarus  **QUAST Contig Browser** | Move << < > >> zoom +5x +2x –2x –5x \| start end Show misassemblies: relocations (32) translocations (0) inversions (0) local (74) | Search contig or gene: |

**Contig alignment viewer**. Contigs aligned to NZ CP009828.1 Staphylococcus aureus strain MS4 complete genome

Hide
Show annotation
Hide
Show read coverage
Show physical
Show physical

+
-
Reset

+
-
Reset
