# Supplementary material for: HiMMe: using genetic patterns as a proxy for genome assembly reliability assessment
Source: BMC Genomics. 2017 Sep 5;18:694. doi: 10.1186/s12864-017-3965-2 (PMC5584555; doi:10.1186/s12864-017-3965-2)
Supplement: Supplementary file 3 — ’Supplementary_file3.zip’, Title: ’Data from GAGE’. Description: contains all real data as well as the pertinent results. (ZIP 18,698 kb) [file 12864_2017_3965_MOESM3_ESM.zip › Real data/QUAST/exact/SGA/report.html]

# QUAST

**Quality Assessment Tool for Genome Assemblies**
by CAB

Loading...

Aligned to
""

Combined reference
Estimated reference size:
 bp
|
 bp
|
 references
|
 fragments
|
 % G+C
|
 chromosomes
  
 reads
|
 mapped
|
 properly paired
  
 genes
|
 operons

Unfortunately, JavaScript in your
browser is disabled or is not supported.
We need JavaScript to build report and plots.

Worst
Median
Best

Show heatmap

|  |  |  |
| --- | --- | --- |
|  |  |  |
|  | |  |
| Contigs are ordered from largest (contig #1) to smallest.  FRCurve: Y is the total number of aligned bases divided by the reference length, in the contigs having the total number of  at most X.  Contigs are broken into nonoverlapping 100 bp windows. Plot shows number of windows for each GC percentage.  Plot shows number of contigs with GC percentage in a certain range. | | |

{"subreports":[],"report":[["Genome statistics",[{"values":["84.368"],"quality":"More is better","isMain":true,"metricName":"Genome fraction (%)"},{"values":["1.035"],"quality":"Less is better","isMain":true,"metricName":"Duplication ratio"},{"values":[16862],"quality":"More is better","isMain":true,"metricName":"Largest alignment"},{"values":[2354073],"quality":"More is better","isMain":true,"metricName":"Total aligned length"},{"values":[4236],"quality":"More is better","isMain":false,"metricName":"NG50"},{"values":[2307],"quality":"More is better","isMain":false,"metricName":"NG75"},{"values":[1988],"quality":"More is better","isMain":false,"metricName":"NA50"},{"values":[3099],"quality":"More is better","isMain":true,"metricName":"NGA50"},{"values":[1184],"quality":"More is better","isMain":false,"metricName":"NGA75"},{"values":[204],"quality":"Less is better","isMain":false,"metricName":"LG50"},{"values":[419],"quality":"Less is better","isMain":false,"metricName":"LG75"},{"values":[400],"quality":"Less is better","isMain":false,"metricName":"LA50"},{"values":[250],"quality":"Less is better","isMain":true,"metricName":"LGA50"},{"values":[596],"quality":"Less is better","isMain":false,"metricName":"LGA75"}]],["Misassemblies",[{"values":[30],"quality":"Less is better","isMain":true,"metricName":"# misassemblies"},{"values":[30],"quality":"Less is better","isMain":false,"metricName":" # relocations"},{"values":[0],"quality":"Less is better","isMain":false,"metricName":" # translocations"},{"values":[0],"quality":"Less is better","isMain":false,"metricName":" # inversions"},{"values":[30],"quality":"Less is better","isMain":false,"metricName":"# misassembled contigs"},{"values":[109966],"quality":"Less is better","isMain":true,"metricName":"Misassembled contigs length"},{"values":[48],"quality":"Less is better","isMain":false,"metricName":"# local misassemblies"},{"values":[8],"quality":"Less is better","isMain":false,"metricName":"# unaligned mis. contigs"}]],["Unaligned",[{"values":[5247],"quality":"Less is better","isMain":false,"metricName":"# fully unaligned contigs"},{"values":[928735],"quality":"Less is better","isMain":false,"metricName":"Fully unaligned length"},{"values":[64],"quality":"Less is better","isMain":false,"metricName":"# partially unaligned contigs"},{"values":[152955],"quality":"Less is better","isMain":false,"metricName":"Partially unaligned length"}]],["Mismatches",[{"values":[32640],"quality":"Less is better","isMain":false,"metricName":"# mismatches"},{"values":[1035],"quality":"Less is better","isMain":false,"metricName":"# indels"},{"values":[2286],"quality":"Less is better","isMain":false,"metricName":"Indels length"},{"values":["1427.69"],"quality":"Less is better","isMain":true,"metricName":"# mismatches per 100 kbp"},{"values":["45.27"],"quality":"Less is better","isMain":true,"metricName":"# indels per 100 kbp"},{"values":[963],"quality":"Less is better","isMain":false,"metricName":" # indels (<= 5 bp)"},{"values":[72],"quality":"Less is better","isMain":false,"metricName":" # indels (> 5 bp)"},{"values":[0],"quality":"Less is better","isMain":false,"metricName":"# N's"},{"values":["0.00"],"quality":"Less is better","isMain":true,"metricName":"# N's per 100 kbp"}]],["Statistics without reference",[{"values":[6854],"quality":"Equal","isMain":true,"metricName":"# contigs"},{"values":[6854],"quality":"Equal","isMain":false,"metricName":"# contigs (>= 0 bp)"},{"values":[764],"quality":"Equal","isMain":false,"metricName":"# contigs (>= 1000 bp)"},{"values":[147],"quality":"Equal","isMain":false,"metricName":"# contigs (>= 5000 bp)"},{"values":[20],"quality":"Equal","isMain":false,"metricName":"# contigs (>= 10000 bp)"},{"values":[0],"quality":"Equal","isMain":false,"metricName":"# contigs (>= 25000 bp)"},{"values":[0],"quality":"Equal","isMain":false,"metricName":"# contigs (>= 50000 bp)"},{"values":[16870],"quality":"More is better","isMain":true,"metricName":"Largest contig"},{"values":[3448095],"quality":"More is better","isMain":true,"metricName":"Total length"},{"values":[3448095],"quality":"More is better","isMain":false,"metricName":"Total length (>= 0 bp)"},{"values":[2583186],"quality":"More is better","isMain":true,"metricName":"Total length (>= 1000 bp)"},{"values":[1097906],"quality":"More is better","isMain":false,"metricName":"Total length (>= 5000 bp)"},{"values":[264524],"quality":"More is better","isMain":true,"metricName":"Total length (>= 10000 bp)"},{"values":[0],"quality":"More is better","isMain":false,"metricName":"Total length (>= 25000 bp)"},{"values":[0],"quality":"More is better","isMain":true,"metricName":"Total length (>= 50000 bp)"},{"values":[3144],"quality":"More is better","isMain":false,"metricName":"N50"},{"values":[991],"quality":"More is better","isMain":false,"metricName":"N75"},{"values":[304],"quality":"Less is better","isMain":false,"metricName":"L50"},{"values":[767],"quality":"Less is better","isMain":false,"metricName":"L75"},{"values":["31.99"],"quality":"Equal","isMain":false,"metricName":"GC (%)"}]],["Predicted genes",[]],["Similarity statistics",[{"values":[0],"quality":"Equal","isMain":false,"metricName":"# similar correct contigs"},{"values":[0],"quality":"Equal","isMain":false,"metricName":"# similar misassembled blocks"}]],["Reference statistics",[{"values":[2709797],"quality":"Equal","isMain":false,"metricName":"Reference length"},{"values":[1],"quality":"Equal","isMain":false,"metricName":"Reference fragments"},{"values":["32.89"],"quality":"Equal","isMain":false,"metricName":"Reference GC (%)"}]]],"assembliesWithNs":null,"referenceName":"staphylococcus\_aureus","date":"15 June 2017, Thursday, 02:13:24","subreferences":[],"minContig":0,"order":[0],"assembliesNames":["genome.ctg.filtered"]}

{{ qualities }}

{{ mainMetrics }}

{"lists\_of\_lengths":[[65237,59023,51538,46344,42382,38446,36702,34655,33834,32599,31643,30884,29984,29071,28236,27197,26897,26570,26110,25832,25298,24658,24181,23872,23622,23138,22849,22561,22140,21954,21717,21270,20863,20750,20527,20233,20078,19718,19352,19192,19038,18729,18404,18227,18050,17874,17667,17540,17358,17204,17036,16729,16560,16449,16391,16267,16025,15728,15557,15432,15243,15073,14932,14690,14457,14215,14037,13867,13606,13443,13254,13149,13047,12799,12737,12651,12545,12267,12011,11813,11730,11669,11608,11565,11455,11349,11256,11172,11062,10834,10719,10637,10527,10427,10332,10254,10033,9902,9783,9738,9675,9583,9378,9329,9247,9215,9149,9099,9001,8924,8848,8675,8587,8534,8430,8300,8233,8145,8087,8040,7978,7924,7855,7790,7655,7629,7562,7525,7456,7377,7271,7139,7076,6998,6944,6914,6886,6840,6812,6718,6692,6646,6591,6552,6515,6462,6411,6330,6250,6207,6109,6004,5966,5943,5887,5817,5774,5704,5657,5602,5508,5450,5396,5363,5322,5295,5241,5191,5166,5130,5065,4990,4917,4881,4859,4805,4742,4706,4667,4535,4472,4440,4379,4358,4288,4240,4194,4148,4103,4056,4022,3977,3936,3906,3867,3810,3783,3758,3729,3689,3654,3622,3598,3540,3490,3467,3428,3396,3381,3345,3298,3258,3231,3210,3156,3123,3087,3029,2991,2947,2927,2900,2872,2807,2770,2738,2694,2634,2601,2578,2566,2538,2506,2449,2423,2384,2340,2306,2276,2246,2216,2178,2133,2083,2057,2043,2000,1974,1959,1945,1911,1881,1831,1803,1772,1745,1720,1686,1653,1638,1630,1607,1592,1577,1561,1548,1520,1506,1482,1456,1447,1427,1375,1323,1281,1264,1244,1219,1195,1176,1155,1138,1123,1108,1099,1082,1064,1044,1036,1031,1021,1009,998,985,973,959,948,933,928,923,915,902,894,881,867,858,847,841,832,824,818,812,803,794,789,786,778,776,770,762,753,745,737,736,730,727,724,722,715,711,707,701,694,692,689,685,682,680,680,676,673,672,668,667,664,662,660,655,652,652,651,648,644,642,637,633,632,630,628,628,628,627,624,624,624,623,620,620,620,620,616,616,616,616,612,612,612,612,612,612,608,608,608,606,604,604,604,604,603,600,600,600,597,596,596,595,592,592,592,589,588,588,588,588,586,584,584,584,584,582,580,580,580,577,576,576,576,575,572,572,569,568,568,568,568,566,564,564,564,562,560,560,560,560,560,560,556,556,556,556,552,552,552,552,552,548,548,548,548,548,548,548,544,544,544,544,544,542,540,540,540,540,540,539,536,536,536,536,533,532,532,532,531,528,528,528,526,524,524,524,523,520,520,520,520,519,516,516,516,516,516,515,512,512,512,512,512,509,508,508,507,504,504,504,504,504,501,500,500,500,499,496,496,496,495,492,492,492,492,492,492,492,492,488,488,488,488,488,487,484,484,484,484,484,484,484,480,480,480,480,480,480,478,476,476,476,476,476,475,472,472,472,472,472,471,468,468,468,468,468,468,465,464,464,464,464,464,464,464,463,460,460,460,460,460,460,460,457,456,456,456,456,456,456,456,455,452,452,452,452,452,452,452,452,452,451,448,448,448,448,448,448,448,448,448,445,444,444,444,444,444,444,444,444,444,444,444,444,440,440,440,440,440,440,440,440,440,440,440,440,440,437,436,436,436,436,436,436,436,436,436,436,436,436,436,436,434,432,432,432,432,432,432,432,432,432,432,432,432,432,432,432,432,432,428,428,428,428,428,428,428,428,428,428,428,428,428,428,428,428,428,428,428,428,428,428,428,425,424,424,424,424,424,424,424,424,424,424,424,424,424,424,424,424,424,424,424,424,424,424,424,424,424,424,424,424,424,424,422,420,420,420,420,420,420,420,420,420,420,420,420,420,420,420,420,420,420,420,420,420,420,420,420,420,420,420,420,420,420,420,420,420,420,420,420,419,416,416,416,416,416,416,416,416,416,416,416,416,416,416,416,416,416,416,416,416,416,416,416,416,416,416,416,416,416,416,416,416,416,416,416,416,416,416,416,416,416,416,416,416,416,416,416,416,416,412,412,412,412,412,412,412,412,412,412,412,412,412,412,412,412,412,412,412,412,412,412,412,412,412,412,412,412,412,412,412,412,412,412,412,412,412,412,412,412,412,412,412,412,412,412,412,412,412,412,412,412,412,412,412,412,412,412,412,412,412,412,412,412,408,408,408,408,408,408,408,408,408,408,408,408,408,408,408,408,408,408,408,408,408,408,408,408,408,408,408,408,408,408,408,408,408,408,408,408,408,408,408,408,408,408,408,408,408,408,408,408,408,408,408,408,408,408,408,408,408,408,408,408,408,408,408,408,408,408,408,408,408,408,408,408,408,408,408,408,408,408,408,408,408,408,408,408,408,408,408,408,408,408,408,408,408,408,408,408,408,408,408,408,408,408,408,408,408,408,408,408,404,404,404,404,404,404,404,404,404,404,404,404,404,404,404,404,404,404,404,404,404,404,404,404,404,404,404,404,404,404,404,404,404,404,404,404,404,404,404,404,404,404,404,404,404,404,404,404,404,404,404,404,404,404,404,404,404,404,404,404,404,404,404,404,404,404,404,404,404,404,404,404,404,404,404,404,404,404,404,404,404,404,404,404,404,404,404,404,404,404,404,404,404,404,404,404,404,404,404,404,404,404,404,404,404,404,404,404,404,404,404,404,404,404,404,404,404,404,404,404,404,404,404,404,404,404,404,404,404,404,404,404,404,404,404,404,404,404,404,404,404,404,404,404,404,404,404,404,404,404,404,404,404,404,404,404,404,404,404,404,404,404,404,404,404,404,404,404,404,404,404,404,404,404,404,404,404,404,404,404,404,404,404,404,404,404,404,404,404,404,404,404,404,404,404,404,404,404,404,404,404,404,404,404,404,404,404,404,404,404,404,404,404,404,404,404,404,404,404,404,404,404,404,404,404,404,404,404,404,404,404,404,404,404,404,404,404,404,404,404,404,404,404,404,404,404,404,404,404,404,404,404,404,404,404,404,404,404,404,404,404,404,404,404,404,404,404,404,404,404,404,404,404,404,404,404,404,404,404,404,404,404,404,404,404,404,404,404,404,404,404,404,404,404,404,404,404,404,404,404,404,404,404,404,404,404,404,404,404,404,404,404,404,404,404,404,404,404,404,404,404,404,404,404,404,404,404,404,404,404,404,404,404,404,404,404,404,404,404,404,404,404,404,404,404,404,404,404,404,404,404,404,404,404,404,404,404,404,404,404,404,404,404,404,404,404,404,404,404,404,404,404,404,404,404,404,404,404,404,404,404,404,404,404,404,404,404,404,404,404,404,404,404,404,404,404,404,404,404,404,404,404,404,404,404,404,404,404,404,404,404,404,404,404,404,404,404,404,404,404,404,404,404,404,404,404,404,404,404,404,404,404,404,404,404,404,404,404,404,404,404,404,404,404,404,404,404,404,404,404,404,404,404,404,404,404,404,404,404,404,404,404,404,404,404,404,404,404,404,404,404,404,404,404,404,404,404,404,404,404,404,404,404,404,404,404,404,404,404,404,404,404,404,404,404,404,404,404,404,404,404,404,404,404,404,404,404,404,404,404,404,404,404,404,404,404,404,404,404,404,404,404,404,404,404,404,404,404,404,404,404,404,404,404,404,404,404,404,404,404,404,404,404,404,404,404,404,404,404,404,404,404,404,404,404,404,404,404,404,404,404,404,404,404,404,404,404,404,404,404,404,404,404,404,404,404,404,404,404,404,404,404,404,404,404,404,404,404,404,404,404,404,404,404,404,404,404,404,404,404,404,404,404,404,404,404,404,404,404,404,404,404,404,404,404,404,404,404,404,404,404,404,404,404,404,404,404,404,404,404,404,404,404,404,404,404,404,404,404,404,404,404,404,404,404,404,404,404,404,404,404,404,404,404,404,404,404,404,404,404,404,404,404,404,404,404,404,404,404,404,404,404,404,404,404,404,404,404,404,404,404,404,404,404,404,404,404,404,404,404,404,404,404,404,404,404,404,404,404,404,404,404,404,404,404,404,404,404,404,404,404,404,404,404,404,404,404,404,404,404,404,404,404,404,404,404,404,404,404,404,404,404,404,404,404,404]],"filenames":["genome.ctg.filtered"]}

{"assemblies\_lengths":[3448095],"filenames":["genome.ctg.filtered"]}

{"reflen":[2709797]}

{"tickX":4}

{"coord\_y":[[16870,16870,16870,16684,16684,16131,16131,15552,15552,15029,15029,14955,14955,14538,14538,14501,14501,14210,14210,12773,12773,12462,12462,12093,12093,12041,12041,11690,11690,11572,11572,11041,11041,10876,10876,10638,10638,10499,10499,10369,10369,9721,9721,9717,9717,9552,9552,9456,9456,9360,9360,9188,9188,9142,9142,9012,9012,8816,8816,8630,8630,8608,8608,8601,8601,8558,8558,8478,8478,8442,8442,8356,8356,8246,8246,8198,8198,8138,8138,8017,8017,7944,7944,7908,7908,7896,7896,7895,7895,7794,7794,7769,7769,7689,7689,7632,7632,7628,7628,7483,7483,7464,7464,7409,7409,7360,7360,7264,7264,7233,7233,7214,7214,7158,7158,7154,7154,7085,7085,6839,6839,6836,6836,6801,6801,6790,6790,6770,6770,6767,6767,6758,6758,6704,6704,6668,6668,6650,6650,6648,6648,6639,6639,6633,6633,6559,6559,6547,6547,6502,6502,6489,6489,6483,6483,6445,6445,6415,6415,6399,6399,6337,6337,6300,6300,6262,6262,6216,6216,6182,6182,6141,6141,6119,6119,6096,6096,6056,6056,6048,6048,5981,5981,5977,5977,5967,5967,5964,5964,5941,5941,5912,5912,5908,5908,5861,5861,5841,5841,5783,5783,5760,5760,5754,5754,5741,5741,5738,5738,5686,5686,5684,5684,5683,5683,5665,5665,5610,5610,5603,5603,5571,5571,5538,5538,5523,5523,5508,5508,5501,5501,5496,5496,5487,5487,5470,5470,5466,5466,5426,5426,5420,5420,5405,5405,5365,5365,5332,5332,5320,5320,5253,5253,5230,5230,5218,5218,5214,5214,5201,5201,5196,5196,5190,5190,5184,5184,5180,5180,5150,5150,5141,5141,5095,5095,5085,5085,5059,5059,5046,5046,5043,5043,5041,5041,5026,5026,5022,5022,4989,4989,4988,4988,4935,4935,4904,4904,4891,4891,4874,4874,4830,4830,4828,4828,4820,4820,4818,4818,4809,4809,4786,4786,4779,4779,4768,4768,4758,4758,4754,4754,4744,4744,4680,4680,4662,4662,4643,4643,4633,4633,4592,4592,4590,4590,4589,4589,4586,4586,4558,4558,4557,4557,4526,4526,4520,4520,4513,4513,4511,4511,4506,4506,4488,4488,4473,4473,4461,4461,4452,4452,4430,4430,4427,4427,4406,4406,4404,4404,4392,4392,4387,4387,4381,4381,4380,4380,4353,4353,4342,4342,4336,4336,4327,4327,4317,4317,4299,4299,4297,4297,4291,4291,4275,4275,4272,4272,4253,4253,4236,4236,4186,4186,4179,4179,4178,4178,4157,4157,4154,4154,4130,4130,4119,4119,4118,4118,4108,4108,4105,4105,4104,4104,4095,4095,4087,4087,4082,4082,4080,4080,4067,4067,4038,4038,4013,4013,4012,4012,4005,4005,3995,3995,3969,3969,3938,3938,3915,3915,3906,3906,3890,3890,3889,3889,3882,3882,3854,3854,3852,3852,3844,3844,3840,3840,3826,3826,3803,3803,3774,3774,3773,3773,3766,3766,3761,3761,3741,3741,3738,3738,3730,3730,3723,3723,3683,3683,3680,3680,3677,3677,3650,3650,3638,3638,3634,3634,3597,3597,3588,3588,3571,3571,3557,3557,3553,3553,3534,3534,3527,3527,3516,3516,3513,3513,3481,3481,3471,3471,3462,3462,3453,3453,3420,3420,3415,3415,3386,3386,3385,3385,3373,3373,3364,3364,3353,3353,3336,3336,3316,3316,3304,3304,3298,3298,3292,3292,3288,3288,3287,3287,3282,3282,3274,3274,3272,3272,3265,3265,3236,3236,3208,3208,3205,3205,3193,3193,3192,3192,3183,3183,3182,3182,3180,3180,3179,3179,3166,3166,3162,3162,3144,3144,3139,3139,3138,3138,3135,3135,3133,3133,3084,3084,3074,3074,3068,3068,3041,3041,3031,3031,3003,3003,2990,2990,2987,2987,2975,2975,2959,2959,2941,2941,2938,2938,2937,2937,2932,2932,2931,2931,2930,2930,2929,2929,2923,2923,2909,2909,2908,2908,2906,2906,2901,2901,2900,2900,2899,2899,2896,2896,2893,2893,2877,2877,2876,2876,2870,2870,2859,2859,2850,2850,2848,2848,2839,2839,2832,2832,2830,2830,2828,2828,2821,2821,2804,2804,2803,2803,2799,2799,2796,2796,2794,2794,2783,2783,2779,2779,2776,2776,2761,2761,2746,2746,2726,2726,2717,2717,2700,2700,2691,2691,2689,2689,2684,2684,2676,2676,2670,2670,2664,2664,2657,2657,2652,2652,2645,2645,2619,2619,2618,2618,2615,2615,2612,2612,2601,2601,2599,2599,2592,2592,2585,2585,2583,2583,2572,2572,2570,2570,2567,2567,2566,2566,2551,2551,2543,2543,2515,2515,2489,2489,2486,2486,2482,2482,2480,2480,2477,2477,2463,2463,2449,2449,2445,2445,2444,2444,2433,2433,2431,2431,2430,2430,2423,2423,2413,2413,2409,2409,2407,2407,2402,2402,2401,2401,2373,2373,2351,2351,2345,2345,2342,2342,2340,2340,2336,2336,2335,2335,2332,2332,2326,2326,2320,2320,2313,2313,2307,2307,2301,2301,2300,2300,2293,2293,2287,2287,2286,2286,2283,2283,2281,2281,2277,2277,2271,2271,2270,2270,2260,2260,2253,2253,2248,2248,2240,2240,2233,2233,2229,2229,2222,2222,2221,2221,2218,2218,2210,2210,2199,2199,2181,2181,2169,2169,2168,2168,2157,2157,2153,2153,2151,2151,2142,2142,2141,2141,2139,2139,2137,2137,2131,2131,2127,2127,2115,2115,2108,2108,2105,2105,2102,2102,2095,2095,2074,2074,2068,2068,2063,2063,2062,2062,2061,2061,2057,2057,2053,2053,2047,2047,2026,2026,2025,2025,2023,2023,2022,2022,2021,2021,2020,2020,2014,2014,2008,2008,1998,1998,1997,1997,1996,1996,1988,1988,1986,1986,1982,1982,1978,1978,1974,1974,1954,1954,1953,1953,1950,1950,1949,1949,1942,1942,1918,1918,1914,1914,1913,1913,1910,1910,1908,1908,1907,1907,1906,1906,1892,1892,1890,1890,1888,1888,1883,1883,1881,1881,1878,1878,1877,1877,1864,1864,1859,1859,1856,1856,1851,1851,1848,1848,1841,1841,1837,1837,1830,1830,1826,1826,1809,1809,1806,1806,1797,1797,1783,1783,1782,1782,1777,1777,1772,1772,1766,1766,1755,1755,1753,1753,1747,1747,1743,1743,1739,1739,1737,1737,1734,1734,1731,1731,1728,1728,1727,1727,1726,1726,1723,1723,1720,1720,1717,1717,1712,1712,1711,1711,1709,1709,1708,1708,1707,1707,1706,1706,1705,1705,1694,1694,1681,1681,1680,1680,1677,1677,1675,1675,1672,1672,1670,1670,1667,1667,1663,1663,1659,1659,1657,1657,1653,1653,1649,1649,1647,1647,1642,1642,1640,1640,1638,1638,1637,1637,1634,1634,1630,1630,1626,1626,1625,1625,1621,1621,1619,1619,1614,1614,1608,1608,1604,1604,1603,1603,1601,1601,1590,1590,1589,1589,1577,1577,1574,1574,1567,1567,1563,1563,1560,1560,1558,1558,1552,1552,1549,1549,1548,1548,1535,1535,1528,1528,1511,1511,1506,1506,1502,1502,1500,1500,1496,1496,1495,1495,1491,1491,1490,1490,1488,1488,1486,1486,1483,1483,1477,1477,1467,1467,1466,1466,1455,1455,1454,1454,1453,1453,1447,1447,1446,1446,1442,1442,1439,1439,1435,1435,1426,1426,1424,1424,1419,1419,1416,1416,1414,1414,1408,1408,1406,1406,1403,1403,1402,1402,1391,1391,1386,1386,1379,1379,1372,1372,1371,1371,1368,1368,1362,1362,1361,1361,1359,1359,1354,1354,1350,1350,1348,1348,1344,1344,1343,1343,1340,1340,1336,1336,1334,1334,1327,1327,1326,1326,1321,1321,1319,1319,1310,1310,1302,1302,1301,1301,1299,1299,1296,1296,1295,1295,1292,1292,1291,1291,1288,1288,1286,1286,1280,1280,1276,1276,1272,1272,1269,1269,1266,1266,1258,1258,1254,1254,1249,1249,1246,1246,1241,1241,1233,1233,1232,1232,1228,1228,1224,1224,1223,1223,1220,1220,1219,1219,1218,1218,1215,1215,1207,1207,1205,1205,1203,1203,1194,1194,1193,1193,1184,1184,1183,1183,1182,1182,1178,1178,1176,1176,1170,1170,1169,1169,1165,1165,1164,1164,1155,1155,1128,1128,1126,1126,1121,1121,1118,1118,1117,1117,1116,1116,1113,1113,1107,1107,1098,1098,1096,1096,1093,1093,1092,1092,1090,1090,1089,1089,1087,1087,1082,1082,1073,1073,1067,1067,1066,1066,1063,1063,1058,1058,1056,1056,1048,1048,1042,1042,1039,1039,1035,1035,1033,1033,1030,1030,1021,1021,1019,1019,1015,1015,1014,1014,1013,1013,1009,1009,1008,1008,1005,1005,1000,1000,999,999,998,998,991,991,989,989,988,988,983,983,982,982,980,980,978,978,976,976,972,972,969,969,968,968,967,967,963,963,962,962,953,953,948,948,947,947,946,946,944,944,941,941,940,940,937,937,934,934,933,933,932,932,930,930,927,927,924,924,920,920,918,918,917,917,914,914,909,909,908,908,907,907,905,905,902,902,901,901,900,900,899,899,898,898,891,891,886,886,882,882,881,881,877,877,876,876,869,869,868,868,867,867,865,865,863,863,860,860,854,854,851,851,850,850,848,848,847,847,845,845,841,841,840,840,839,839,835,835,831,831,830,830,824,824,823,823,821,821,820,820,817,817,811,811,810,810,809,809,808,808,806,806,805,805,800,800,796,796,788,788,787,787,785,785,781,781,780,780,778,778,772,772,770,770,767,767,761,761,759,759,756,756,753,753,752,752,749,749,746,746,744,744,741,741,737,737,735,735,734,734,733,733,732,732,729,729,725,725,724,724,722,722,720,720,719,719,718,718,715,715,709,709,705,705,698,698,695,695,694,694,693,693,692,692,691,691,687,687,683,683,681,681,678,678,668,668,667,667,661,661,660,660,658,658,655,655,654,654,653,653,648,648,646,646,644,644,643,643,641,641,637,637,635,635,634,634,632,632,630,630,629,629,628,628,619,619,614,614,612,612,611,611,610,610,607,607,605,605,601,601,599,599,594,594,590,590,586,586,585,585,583,583,578,578,577,577,574,574,573,573,570,570,569,569,564,564,563,563,562,562,559,559,557,557,554,554,553,553,552,552,551,551,544,544,543,543,540,540,537,537,533,533,530,530,529,529,522,522,516,516,514,514,513,513,511,511,510,510,509,509,507,507,499,499,498,498,496,496,494,494,493,493,491,491,490,490,489,489,487,487,486,486,483,483,481,481,479,479,478,478,473,473,471,471,469,469,468,468,461,461,458,458,456,456,455,455,452,452,448,448,447,447,446,446,440,440,439,439,438,438,435,435,433,433,432,432,430,430,429,429,426,426,422,422,419,419,415,415,413,413,412,412,411,411,408,408,407,407,403,403,401,401,400,400,398,398,396,396,395,395,394,394,393,393,390,390,389,389,388,388,387,387,385,385,381,381,380,380,379,379,378,378,377,377,374,374,373,373,371,371,367,367,365,365,363,363,362,362,361,361,360,360,359,359,355,355,353,353,346,346,345,345,342,342,338,338,332,332,329,329,324,324,323,323,321,321,320,320,317,317,316,316,314,314,312,312,311,311,310,310,308,308,306,306,305,305,300,300,298,298,297,297,296,296,295,295,293,293,292,292,289,289,288,288,287,287,284,284,283,283,281,281,280,280,279,279,278,278,276,276,275,275,274,274,273,273,269,269,267,267,266,266,265,265,263,263,261,261,260,260,259,259,258,258,257,257,255,255,254,254,253,253,250,250,249,249,247,247,246,246,245,245,244,244,243,243,242,242,240,240,239,239,238,238,237,237,236,236,234,234,233,233,232,232,231,231,230,230,229,229,228,228,226,226,225,225,224,224,223,223,222,222,221,221,219,219,218,218,217,217,216,216,215,215,214,214,213,213,212,212,211,211,210,210,209,209,208,208,207,207,206,206,205,205,204,204,203,203,202,202,201,201,200,200,199,199,198,198,197,197,196,196,195,195,194,194,193,193,192,192,191,191,190,190,189,189,188,188,186,186,185,185,184,184,183,183,182,182,181,181,180,180,179,179,178,178,177,177,176,176,175,175,174,174,173,173,172,172,171,171,170,170,169,169,168,168,167,167,166,166,165,165,164,164,163,163,162,162,161,161,160,160,159,159,158,158,157,157,156,156,155,155,154,154,153,153,152,152,151,151,150,150,149,149,148,148,147,147,146,146,145,145,144,144,143,143,142,142,141,141,140,140,139,139,138,138,137,137,136,136,135,135,134,134,133,133,132,132,131,131,130,130,129,129,128,128,127,127,126,126,125,125,124,124,123,123,122,122,121,121,120,120,119,119,118,118,117,117,116,116,115,115,114,114,113,113,112,112,111,111,110,110,109,109,108,108,107,107,106,106,105,105,104,104,103,103,102,102,101,101,0.0]],"coord\_x":[[0.0,1e-10,0.48925566145944355,0.48925566155944356,0.9731170399887474,0.9731170400887474,1.440940577333281,1.440940577433281,1.8919722339436704,1.8919722340436704,2.327836095003183,2.327836095103183,2.7615538434990916,2.7615538435990916,3.1831779576838803,3.1831779577838804,3.6037290155868673,3.6037290156868673,4.015840630841088,4.015840630941088,4.3862770602318095,4.3862770603318095,4.7476940165511685,4.7476940166511685,5.098409411573637,5.098409411673637,5.4476167274973575,5.4476167275973575,5.786644509504524,5.786644509604524,6.122250112018375,6.122250112118375,6.442455906812312,6.442455906912312,6.757876450619835,6.757876450719835,7.066394632398469,7.066394632498469,7.370881602740064,7.370881602840064,7.671598375334787,7.671598375434787,7.95352216223741,7.95352216233741,8.235329943055513,8.235329943155513,8.512352472887203,8.512352472987203,8.786590856690433,8.786590856790433,9.058045094465205,9.058045094565205,9.324511070605654,9.324511070705654,9.589642976774131,9.589642976874131,9.851004685195738,9.851004685295738,10.106682095475907,10.106682095575907,10.356965222825938,10.356965222925938,10.606610316711112,10.606610316811112,10.856052399948377,10.856052400048377,11.104247417777062,11.104247417877062,11.350122313915366,11.350122314015366,11.594953155292995,11.594953155392995,11.837289865853464,11.837289865953464,12.076436409089657,12.076436409189657,12.314190879311619,12.314190879411619,12.550205258265796,12.550205258365796,12.782710453163268,12.782710453263268,13.013098537018267,13.013098537118267,13.242442566112592,13.242442566212592,13.47143857695336,13.47143857705336,13.700405586273,13.700405586373,13.926443441958531,13.926443442058531,14.151756259615818,14.151756259715818,14.37474895558272,14.37474895568272,14.596088564845227,14.596088564945227,14.817312168023212,14.817312168123213,15.034330550637382,15.034330550737382,15.250797904350083,15.250797904450083,15.465670174400648,15.465670174500648,15.679121369915853,15.679121370015853,15.889788419402597,15.889788419502597,16.09955642173432,16.09955642183432,16.308773395164575,16.308773395264573,16.516366283411564,16.516366283511562,16.723843165574035,16.723843165674033,16.929318942778547,16.929318942878545,17.127660345785138,17.127660345885136,17.325914744228335,17.325914744328333,17.52315408943199,17.52315408953199,17.72007441790322,17.720074418003218,17.916414715951852,17.91641471605185,18.112668009437094,18.112668009537092,18.30866028923217,18.308660289332167,18.503086486886236,18.503086486986234,18.69646862977963,18.69646862987963,18.88932874529269,18.88932874539269,19.082130857763488,19.082130857863486,19.274671956544122,19.27467195664412,19.46703904619797,19.46703904629797,19.65726002328822,19.65726002338822,19.84713298212491,19.84713298222491,20.035700872510763,20.03570087261076,20.412459633507776,20.412459633607774,20.60047649499216,20.60047649509216,20.787391298673615,20.787391298773613,20.973436056721177,20.973436056821175,21.15901679043066,21.159016790530657,21.342799429830094,21.342799429930093,21.52550901294773,21.525509013047728,21.707116538262433,21.70711653836243,21.887389993605165,21.887389993705163,22.066677397229483,22.06667739732948,22.244775738487483,22.24477573858748,22.422236046280627,22.422236046380625,22.599029319087787,22.599029319187785,22.774662531049753,22.77466253114975,22.950063730842682,22.95006373094268,23.123521828719916,23.123521828819914,23.29686392051263,23.29686392061263,23.46991599709405,23.469915997194047,23.642881069112075,23.642881069212073,23.988144178162145,23.988144178262143,24.159601171081423,24.15960117118142,24.330942157916184,24.33094215801618,24.50092007325784,24.50092007335784,24.670317958176906,24.670317958276904,24.838033754870445,24.838033754970443,25.005082516577996,25.005082516677994,25.171957269158767,25.171957269258765,25.338455001964853,25.33845500206485,25.50486573020755,25.504865730307547,25.669768379351495,25.669768379451494,25.834613025453184,25.834613025553182,25.999428670033744,25.999428670133742,26.163722287233966,26.163722287333965,26.32642082077205,26.32642082087205,26.488916343662225,26.488916343762224,26.650483817876246,26.650483817976244,26.811094241892988,26.811094241992986,26.97126964309278,26.971269643192777,27.131010021475625,27.131010021575623,27.290547389210563,27.29054738931056,27.44993974933985,27.44993974943985,27.609071095778972,27.60907109587897,27.767709416358887,27.767709416458885,27.926231730854283,27.92623173095428,28.083593984504486,28.083593984604484,28.240782229027914,28.240782229127912,28.39753545073439,28.39753545083439,28.553128611595678,28.553128611695676,28.707764722259682,28.70776472235968,28.86205281467013,28.862052814770127,29.01439780516488,29.014397805264878,29.166075760673646,29.166075760773644,29.317405697928855,29.317405698028853,29.468619629099546,29.468619629199544,29.61945654049555,29.619456540595547,29.7701484442859,29.7701484443859,29.920666338949477,29.920666339049475,30.071010224486276,30.071010224586274,30.22123810393855,30.221238104038548,30.370595937756935,30.370595937856933,30.51969275788515,30.519692757985148,30.816552328169614,30.816552328269612,30.964025063114562,30.96402506321456,31.110743758510132,31.11074375861013,31.25708543413102,31.25708543423102,31.403340105188516,31.403340105288514,31.549536773203755,31.549536773303753,31.695298418402047,31.695298418502045,31.840944057515816,31.840944057615815,31.985632646432304,31.985632646532302,32.13029223382767,32.13029223392767,32.273414740603144,32.273414740703146,32.415638200223604,32.415638200323606,32.55748464006937,32.55748464016937,32.69883805405593,32.698838054155935,32.838915401112786,32.83891540121279,32.97893474512738,32.978934745227384,33.11872207697294,33.11872207707294,33.258451405776235,33.25845140587624,33.39791972088936,33.397919720989364,33.5367210010165,33.536721001116504,33.675319270495734,33.675319270595736,33.81359852324255,33.81359852334255,33.951587760778054,33.951587760878056,34.22745022976455,34.22745022986455,34.36503344600424,34.36503344610424,34.50076056489163,34.50076056499163,34.635965656398675,34.635965656498676,34.77061971900426,34.77061971910426,34.904983766398544,34.904983766498546,35.038158751426515,35.03815875152652,35.171275733412216,35.17127573351222,35.304363713876796,35.3043637139768,35.43736468977798,35.43736468987798,35.56955362308753,35.56955362318753,35.70171355487595,35.70171355497595,35.83297443950935,35.83297443960935,35.964061315015975,35.96406131511598,36.094945179874685,36.09494517997469,36.22577104169114,36.22577104179114,36.35645189590194,36.35645189600194,36.48661072273241,36.48661072283241,36.61633452674593,36.61633452684593,36.74571031250589,36.745710312605894,36.87482508457568,36.874825084675685,37.00330182318063,37.00330182328063,37.131691557222176,37.13169155732218,37.25947225932,37.25947225942,37.38719495837557,37.38719495847557,37.51456963917757,37.514569639277575,37.641799312373934,37.641799312473935,37.768854976443514,37.768854976543516,37.895881638991966,37.89588163909197,38.02212526046991,38.022125260569915,38.148049865215434,38.148049865315436,38.273800460834174,38.273800460934176,38.39929004276274,38.399290042862745,38.524489609480014,38.524489609580016,38.64916714881696,38.64916714891696,38.77378668511163,38.77378668521163,38.89823221227953,38.89823221237953,39.02221371510936,39.02221371520936,39.14610821337579,39.14610821347579,39.26945168274076,39.26945168284076,39.39230212624652,39.39230212634652,39.513702493695796,39.5137024937958,39.75630021794643,39.756300218046434,39.877468573226665,39.87746857332667,39.99802789656317,39.998027896663174,40.1185002153363,40.1185002154363,40.2382764976023,40.2382764977023,40.357733763135876,40.35773376323588,40.477162027148324,40.477162027248326,40.71572853996192,40.715728540061924,40.83477978419968,40.834779784299684,41.072853271154074,41.072853271254075,41.191614500180535,41.19161450028054,41.31014371703796,41.31014371713796,41.42852792628973,41.428527926389734,41.54685413249925,41.54685413259925,41.66480331893408,41.66480331903408,41.78191146125614,41.781911461356145,41.89829456554996,41.89829456564996,42.01464866832266,42.01464866842266,42.13079976044744,42.13079976054744,42.24666083736092,42.24666083746092,42.36176787472503,42.361767874825034,42.475975864934114,42.475975865034115,42.589516820157215,42.58951682025722,42.70279676169015,42.702796761790154,42.81561267888501,42.81561267898501,42.928399594558734,42.928399594658735,43.26655733093201,43.26655733103201,43.37832919336619,43.37832919346619,43.49004305275812,43.49004305285812,43.601524899981,43.601524900081,43.712890741119374,43.712890741219375,43.82385056096192,43.823850561061924,43.93414334581849,43.93414334591849,44.04359508656229,44.04359508666229,44.153017825784964,44.153017825884966,44.3716602935824,44.3716602936824,44.48073501455151,44.480735014651515,44.589229705098035,44.589229705198036,44.69763739108116,44.69763739118116,44.805813064895254,44.805813064995256,44.913785728061434,44.913785728161436,45.02059833038243,45.02059833048243,45.12732392814003,45.12732392824003,45.23396252133424,45.23396252143424,45.339818073457955,45.33981807355796,45.4453256073281,45.4453256074281,45.550717135113736,45.55071713521374,45.65503560661757,45.65503560671757,45.75909306443123,45.75909306453123,45.86265749638569,45.86265749648569,45.96581590704432,45.965815907144325,46.06885831161844,46.068858311718444,46.1713496872911,46.1713496873911,46.273638052315846,46.27363805241585,46.37560740060816,46.37560740070816,46.47748974433709,46.477489744437094,46.578444039389865,46.57844403948987,46.78006261428412,46.78006261438412,46.88046588043543,46.88046588053543,46.98060813289657,46.98060813299657,47.07979333516043,47.07979333526043,47.17883352981864,47.17883352991864,47.27703268036409,47.27703268046409,47.3752028293884,47.3752028294884,47.47302496015916,47.47302496025916,47.57058607723975,47.570586077339755,47.667828177587914,47.667828177687916,47.86181935242504,47.86181935252504,47.957988396491395,47.9579883965914,48.0538094223042,48.0538094224042,48.14945643899023,48.14945643909023,48.24492944654947,48.24492944664947,48.3402864480242,48.3402864481242,48.4356144479778,48.4356144480778,48.53079744032574,48.530797440425744,48.62574842050466,48.62574842060466,48.72064139764131,48.72064139774131,48.815331364130046,48.81533136423005,48.90918028650603,48.90918028660603,49.00221716629037,49.00221716639037,49.095167041511324,49.095167041611326,49.18776889847872,49.187768898578724,49.37294361089239,49.372943610992394,49.46525545264849,49.465255452748494,49.557538292883464,49.557538292983466,49.649763130076174,49.649763130176176,49.741958965747756,49.74195896584776,49.83377778164465,49.83377778174465,49.925480591457024,49.925480591557026,50.01666137388906,50.01666137398906,50.10769714871545,50.107697148815454,50.19870392202071,50.198703922120714,50.28962369076258,50.289623690862584,50.38048545646219,50.38048545656219,50.469926147626445,50.46992614772645,50.559076823579396,50.5590768236794,50.64805349040557,50.648053490505575,50.736247116161245,50.73624711626125,50.82415072670562,50.82415072680562,50.91124229465836,50.91124229475836,50.99795684283641,50.99795684293641,51.08458438645107,51.08458438655107,51.17086391181218,51.17086391191218,51.256679412835204,51.256679412935206,51.3419728864779,51.3419728865779,51.4271793555572,51.4271793556572,51.512356823115375,51.51235682321538,51.5973892830679,51.5973892831679,51.68239274149929,51.682392741599294,51.76736719840956,51.76736719850956,51.85231265379869,51.85231265389869,51.93708410006105,51.93708410016105,52.021449525027585,52.021449525127586,52.105785948473,52.105785948573,52.19006436887615,52.19006436897615,52.27419778167365,52.27419778177365,52.44243560574752,52.44243560584752,52.52651101550276,52.526511015602765,52.610499420694616,52.61049942079462,52.69440082132308,52.69440082142308,52.77783819761346,52.777838197713464,52.86124657238272,52.86124657248272,52.9444809380252,52.9444809381252,53.02739628693525,53.02739628703525,53.11005062215513,53.11005062225513,53.19264695433275,53.19264695443275,53.27498227282021,53.27498227292021,53.357114580659754,53.357114580759756,53.43918888545704,53.439188885557044,53.52120518721207,53.52120518731207,53.603018478319186,53.60301847841919,53.6843387435671,53.684338743667105,53.76563000729388,53.76563000739388,53.846805264936144,53.846805265036146,53.92789351801502,53.92789351811502,54.00892376805164,54.00892376815164,54.08963500135582,54.089635001455825,54.17023022857549,54.17023022867549,54.25073845123177,54.25073845133177,54.3308116510711,54.3308116511711,54.41044982809348,54.410449828193485,54.489507974693275,54.48950797479328,54.568305107602896,54.5683051077029,54.64660921465331,54.64660921475331,54.72465230801355,54.724652308113555,54.80263739833154,54.80263739843154,54.88047748104388,54.880477481143885,54.95808555158718,54.958085551687184,55.0355196130037,55.0355196131037,55.112779665293445,55.11277966539345,55.26709675922502,55.26709675932502,55.3440087932612,55.344008793361205,55.420717816649486,55.42071781674949,55.57338182387666,55.57338182397666,55.649307806194436,55.64930780629444,55.725146783948816,55.72514678404882,55.80089875713981,55.800898757239814,55.876331713598375,55.87633171369838,55.95170666701468,55.951706667114685,56.02687860978308,56.026878609883084,56.101847541903574,56.101847542003576,56.1767584709818,56.176758471081804,56.251350383327605,56.25135038342761,56.32588429263115,56.32588429273115,56.4003311973713,56.4003311974713,56.47474910059032,56.474749100690325,56.5487319809924,56.5487319810924,56.62248284922544,56.62248284932544,56.69542167486685,56.69542167496685,56.76760646095888,56.76760646105888,56.83970424248752,56.83970424258752,56.91168601793164,56.911686018031645,56.9836097903335,56.983609790433505,57.05544655817198,57.05544655827198,57.12687730471463,57.126877304814634,57.19790202996147,57.19790203006147,57.268810749123794,57.268810749223796,57.4105991859273,57.410599186027305,57.55203960447726,57.55203960457726,57.62254230234376,57.62254230244376,57.693015998689134,57.693015998789136,57.83376038073197,57.83376038083197,57.903741051218134,57.903741051318136,57.97360571561978,57.973605715719785,58.04341237697917,58.04341237707917,58.11307403073291,58.11307403083291,58.182706682965524,58.182706683065526,58.2515272926065,58.2515272927065,58.31970986878262,58.31970986888262,58.38771843583196,58.38771843593196,58.45563999831791,58.455639998417915,58.52350355776161,58.52350355786161,58.591251111120776,58.59125111122078,58.658969662958825,58.658969663058826,58.726601210233476,58.72660121033348,58.79405874838135,58.794058748481355,58.86134227740245,58.86134227750245,58.92842279577564,58.92842279587564,58.99532930502205,58.99532930512205,59.26278133288091,59.262781332980914,59.32948483147941,59.329484831579414,59.395985319430004,59.395985319530006,59.46231179825382,59.462311798353824,59.528609275556505,59.52860927565651,59.5948197482958,59.5948197483958,59.660972217992835,59.66097221809284,59.72700868160535,59.72700868170535,59.79287113609109,59.79287113619109,59.8587045890557,59.8587045891557,59.924248026809,59.924248026909005,59.98958845391441,59.98958845401441,60.05478387341416,60.05478387351416,60.119747280744875,60.11974728084488,60.2494710847584,60.2494710848584,60.31411547535668,60.31411547545668,60.37855685530706,60.37855685540706,60.44296923373631,60.44296923383631,60.50729460760217,60.50729460770217,60.57138796929899,60.57138796939899,60.63516231426338,60.635162314363384,60.69841463184744,60.69841463194744,60.76131893117794,60.76131893127794,60.82419422898731,60.82419422908731,60.88675051006425,60.886750510164255,60.94919078505668,60.94919078515668,61.01157305700684,61.011573057106844,61.073694315266835,61.073694315366836,61.1357865720057,61.1357865721057,61.19782082570231,61.19782082580231,61.259797076356655,61.25979707645666,61.32159931788422,61.32159931798422,61.38328555332727,61.38328555342727,61.44462377051676,61.444623770616765,61.50575897705835,61.50575897715835,61.56680717903654,61.56680717913654,61.62776837645134,61.627768376551344,61.68852656321824,61.68852656331824,61.74867571804141,61.74867571814141,61.8086508637378,61.808650863837805,61.86848100182855,61.86848100192855,61.92828213839816,61.92828213849816,61.98805427344664,61.988054273546645,62.04771040241061,62.04771040251061,62.10725052529005,62.10725052539005,62.166616639042715,62.16661663914272,62.28473983460432,62.284739834704325,62.34346791489214,62.34346791499214,62.40213799213769,62.40213799223769,62.46077906786211,62.460779067962115,62.5193911420654,62.519391142165404,62.63658628895085,62.636586289050854,62.69499535250624,62.69499535260624,62.75323040693485,62.75323040703485,62.81117544615215,62.81117544625215,62.86909148384833,62.86909148394833,62.98489455771955,62.98489455781955,63.04254958172556,63.04254958182556,63.100146602689314,63.100146602789316,63.157627617568544,63.157627617668545,63.214992626363255,63.21499262646326,63.32960663786815,63.329606637968155,63.44352461286594,63.443524612965945,63.50016458363241,63.50016458373241,63.55671754983549,63.55671754993549,63.61324151451743,63.613241514617435,63.72608643323343,63.72608643333343,63.78171135076035,63.78171135086035,63.837220262202756,63.83722026230276,63.89270017212403,63.89270017222403,63.948093077481914,63.948093077581916,64.00342797979754,64.00342797989754,64.11406878290767,64.11406878300767,64.16934568218103,64.16934568228103,64.22421656015858,64.22421656025858,64.33390031307142,64.33390031317143,64.38865518496445,64.38865518506445,64.44326504925183,64.44326504935184,64.55242677478434,64.55242677488434,64.60689163146607,64.60689163156607,64.66132748662667,64.66132748672668,64.71538632201259,64.7153863221126,64.76930014979285,64.76930014989286,64.82312697300974,64.82312697310974,64.87680878862096,64.87680878872096,64.93040359966881,64.93040359976881,64.98379540006873,64.98379540016873,65.03707119438414,65.03707119448414,65.09014397805164,65.09014397815164,65.14310075563463,65.14310075573464,65.19556450735841,65.19556450745841,65.24794125451879,65.2479412546188,65.30005698798902,65.30005698808903,65.35176670016342,65.35176670026343,65.4034474108167,65.4034474109167,65.45498311386432,65.45498311396432,65.5063738093063,65.5063738094063,65.60898119106348,65.60898119116348,65.71109554696143,65.71109554706143,65.76193521350194,65.76193521360194,65.81260087091567,65.81260087101568,65.8631505222449,65.8631505223449,65.91358416748959,65.91358416758959,65.96395980969201,65.96395980979202,66.01424844733106,66.01424844743106,66.11473871804576,66.11473871814576,66.16485334655803,66.16485334665803,66.26505360206143,66.26505360216143,66.31511022753143,66.31511022763144,66.36507984843806,66.36507984853806,66.41496246478128,66.41496246488128,66.46475807656111,66.46475807666111,66.5144086807353,66.5144086808353,66.56403028338836,66.56403028348836,66.61359388299917,66.61359388309917,66.66312848108883,66.66312848118883,66.71263407765737,66.71263407775737,66.76211067270478,66.76211067280478,66.81155826623106,66.81155826633106,66.86068684302492,66.86068684312492,66.90943840004408,66.90943840014408,66.95816095554211,66.95816095564211,67.0555190619748,67.05551906207481,67.10409660986718,67.10409660996719,67.20116470108857,67.20116470118857,67.24959724137531,67.24959724147531,67.29794277709865,67.29794277719866,67.34617230673749,67.34617230683749,67.39428583029179,67.3942858303918,67.44234135080384,67.44234135090385,67.49028086523138,67.49028086533139,67.5381043735744,67.5381043736744,67.58586987887514,67.58586987897515,67.63349037657025,67.63349037667025,67.6810528712231,67.6810528713231,67.72855736283368,67.72855736293369,67.77603285292314,67.77603285302314,67.87089682853866,67.87089682863866,67.91816930798021,67.91816930808021,67.96532578133723,67.96532578143723,68.01245325317313,68.01245325327314,68.0594647189245,68.0594647190245,68.10641818163363,68.10641818173363,68.1532266367371,68.1532266368371,68.1998610827138,68.1998610828138,68.24637952260596,68.24637952270596,68.292868960977,68.292868961077,68.38578983467683,68.38578983477683,68.43190225327318,68.43190225337318,68.4779856703484,68.4779856704484,68.52372106917008,68.52372106927008,68.56936946342836,68.56936946352836,68.61481484703873,68.61481484713873,68.66014422456458,68.66014422466458,68.70538659752704,68.70538659762704,68.79581334040971,68.79581334050971,68.84082370120313,68.84082370130314,68.88574705743316,68.88574705753317,68.93064141214207,68.93064141224207,68.97515874707628,68.97515874717628,69.06399040629681,69.06399040639681,69.10781170472391,69.10781170482392,69.15148799554537,69.15148799564537,69.19504828028231,69.19504828038231,69.23855056197698,69.23855056207698,69.28193683758712,69.28193683768713,69.32529411167616,69.32529411177616,69.36853537968067,69.36853537978067,69.41174764616405,69.41174764626405,69.49811417608854,69.49811417618854,69.54121043648739,69.54121043658739,69.62731595272172,69.62731595282172,69.6701511994304,69.67015119953041,69.7555316776365,69.7555316777365,69.79804790761276,69.79804790771276,69.8402451208566,69.8402451209566,69.92461054582313,69.92461054592313,69.9667497560247,69.9667497561247,70.0087149570995,70.0087149571995,70.05065115665316,70.05065115675316,70.09247135012231,70.09247135022231,70.13420453902808,70.13420453912808,70.17582172184932,70.17582172194932,70.21717789098038,70.21717789108038,70.25847605706919,70.25847605716919,70.29962921555236,70.29962921565236,70.38184852795528,70.38184852805529,70.4228566788328,70.4228566789328,70.46369082058354,70.46369082068354,70.50446695929202,70.50446695939202,70.5451560934371,70.5451560935371,70.58581622606106,70.58581622616106,70.62615734195259,70.6261573420526,70.66635345023846,70.66635345033846,70.70634654787644,70.70634654797644,70.7461366348665,70.7461366349665,70.78589772033543,70.78589772043543,70.82557180124097,70.82557180134097,70.86507187301974,70.86507187311975,70.90454294327738,70.90454294337738,70.94395601049276,70.94395601059276,70.98322407010248,70.98322407020248,71.02237612362768,71.02237612372768,71.06147017411064,71.06147017421064,71.10044821850906,71.10044821860906,71.17837530578478,71.17837530588479,71.21723734409869,71.2172373441987,71.25598337632809,71.25598337642809,71.29467140551522,71.29467140561522,71.37184445324158,71.37184445334158,71.48727050733811,71.48727050743811,71.52558151675055,71.52558151685055,71.60214553253319,71.60214553263319,71.64013752521319,71.6401375253132,71.7158894984042,71.7158894985042,71.75362047739404,71.75362047749404,71.79129345334162,71.79129345344163,71.82887942472583,71.82887942482583,71.8664363945889,71.8664363946889,71.90390635988858,71.90390635998858,71.97881728896681,71.97881728906681,72.05361221196051,72.05361221206051,72.09090816813342,72.09090816823343,72.12803011517954,72.12803011527954,72.16503605614115,72.16503605624115,72.20192599101823,72.20192599111823,72.23872892133193,72.23872892143193,72.27544484708223,72.27544484718223,72.31192876066349,72.3119287607635,72.34829666816024,72.34829666826025,72.38451956805135,72.38451956815135,72.42065546337905,72.42065546347905,72.45664635110111,72.45664635120112,72.49240522665414,72.49240522675414,72.52813510068603,72.52813510078603,72.5637489686334,72.5637489687334,72.59924683049626,72.59924683059626,72.63471569083798,72.63471569093798,72.67009754661632,72.67009754671632,72.70545040087353,72.70545040097353,72.81147996212401,72.81147996222401,72.8467168102967,72.8467168103967,72.88172164630035,72.88172164640035,72.91666847926173,72.91666847936173,72.95155730918087,72.95155730928087,73.02107395532896,73.02107395542896,73.0556727700368,73.0556727701368,73.09001057105445,73.09001057115445,73.12431937055099,73.124319370651,73.1585991685264,73.1585991686264,73.22704275839268,73.22704275849269,73.2611485472413,73.2611485473413,73.29508032696315,73.29508032706315,73.32898310516387,73.32898310526387,73.39667265548078,73.39667265558079,73.43043042607584,73.43043042617585,73.46392718298075,73.46392718308076,73.49664089881514,73.49664089891515,73.52929661160728,73.52929661170728,73.5944630295859,73.5944630296859,73.626886730209,73.626886730309,73.65928142931097,73.65928142941097,73.69164712689181,73.69164712699181,73.72392581990925,73.72392582000926,73.78830919681738,73.78830919691738,73.85225755090855,73.85225755100855,73.8840432180668,73.8840432181668,73.91574188066164,73.91574188076164,73.94741154173536,73.94741154183536,74.01069286084055,74.01069286094055,74.04227551735089,74.04227551745089,74.07380017081896,74.07380017091896,74.10517981668139,74.10517981678139,74.13629844885364,74.13629844895364,74.16724307189912,74.16724307199912,74.19815869342347,74.19815869352347,74.22898731038443,74.22898731048443,74.2904995367007,74.2904995368007,74.32112514301375,74.32112514311375,74.38214434347081,74.38214434357081,74.44275752263206,74.44275752273207,74.4728901030859,74.4728901031859,74.53303925790908,74.53303925800908,74.59301440360547,74.59301440370547,74.62288597036914,74.62288597046914,74.65249652344265,74.65249652354265,74.6820490734739,74.6820490735739,74.71148561742064,74.71148561752064,74.74089315984624,74.74089315994624,74.7996792431763,74.7996792432763,74.82894177799625,74.82894177809625,74.85817531129507,74.85817531139507,74.88732184003051,74.88732184013051,74.9163233611603,74.9163233612603,74.94529588076895,74.94529588086895,74.97423939885647,74.97423939895647,75.00297990629609,75.00297990639609,75.03166241069344,75.03166241079344,75.06031591356967,75.06031591366967,75.08882440884024,75.08882440894024,75.14581239786027,75.14581239796027,75.17423388856746,75.17423388866746,75.20259737623239,75.20259737633239,75.23090286085505,75.23090286095506,75.2590923393932,75.25909233949321,75.28719481336796,75.28719481346796,75.31526828582159,75.31526828592159,75.34331275675409,75.34331275685409,75.37124122160208,75.37124122170208,75.39914068492892,75.39914068502893,75.4267791345656,75.42677913466561,75.45427257659664,75.45427257669664,75.48173701710655,75.48173701720656,75.53663689660523,75.53663689670523,75.59144977154051,75.59144977164051,75.61874020292365,75.61874020302365,75.64600163278564,75.64600163288564,75.70043748794625,75.70043748804625,75.72752490868146,75.72752490878146,75.75458332789555,75.75458332799555,75.7816127455885,75.7816127456885,75.8085841602392,75.80858416033921,75.83546857032651,75.83546857042651,75.86226597585043,75.86226597595044,75.88894737528983,75.88894737538983,75.91557077168697,75.91557077178697,75.94216516656299,75.94216516666299,75.9686725568756,75.9686725569756,76.0215423298952,76.0215423299952,76.04787571108105,76.04787571118105,76.07418009074576,76.07418009084576,76.10042646736821,76.10042646746821,76.12658583942728,76.12658583952728,76.1527162099652,76.15271621006521,76.17881757898202,76.17881757908202,76.20488994647769,76.2048899465777,76.23093331245224,76.23093331255224,76.25677366777887,76.25677366787887,76.28246901549986,76.28246901559986,76.30804835713633,76.30804835723633,76.33359869725167,76.33359869735168,76.3590330312825,76.3590330313825,76.38443836379219,76.38443836389219,76.40964068565397,76.40964068575397,76.43481400599461,76.43481400609461,76.4851316451548,76.4851316452548,76.53536227975157,76.53536227985157,76.56039059248657,76.56039059258657,76.58533190065819,76.58533190075819,76.61009919970303,76.61009919980303,76.63477949418447,76.63477949428447,76.65943078714479,76.65943078724479,76.70867537002316,76.70867537012316,76.78242623825619,76.7824262383562,76.80693252361087,76.80693252371087,76.831322802881,76.831322802981,76.85568408063003,76.85568408073003,76.88001635685792,76.88001635695792,76.90423262700129,76.9042326271013,76.92833289106014,76.92833289116014,76.95240415359785,76.95240415369786,76.97630140700879,76.97630140710879,77.00016965889861,77.00016965899862,77.02397990774617,77.02397990784617,77.04776115507258,77.04776115517258,77.07145539783562,77.07145539793562,77.09497563147187,77.09497563157187,77.11846686358699,77.11846686368699,77.141929094181,77.141929094281,77.16536232325386,77.16536232335386,77.21217077835732,77.21217077845732,77.2355170028668,77.2355170029668,77.2820644442801,77.2820644443801,77.32835087200324,77.32835087210324,77.35120407065351,77.35120407075351,77.37402826778265,77.37402826788265,77.39679446186952,77.39679446196952,77.41944464987189,77.4194446499719,77.48736621235784,77.48736621245784,77.5099293957968,77.5099293958968,77.532318570109,77.532318570209,77.55464974137894,77.55464974147894,77.57689390808548,77.57689390818548,77.59896406566524,77.59896406576524,77.62097622020275,77.62097622030275,77.64290137017687,77.64290137027687,77.6647395155876,77.6647395156876,77.6865486594772,77.6865486595772,77.7082707988034,77.7082707989034,77.72990593356622,77.72990593366622,77.75148306528678,77.75148306538678,77.77297319244394,77.77297319254394,77.7943473135166,77.7943473136166,77.81566343154698,77.81566343164698,77.83695054805625,77.83695054815625,77.85820866304438,77.85820866314438,77.90069589149951,77.90069589159951,77.92183800040313,77.92183800050313,77.96400621212582,77.96400621222583,77.98500331342379,77.98500331352379,78.0059424116795,78.0059424117795,78.02682350689294,78.02682350699294,78.04767560058525,78.04767560068525,78.06849869275644,78.06849869285644,78.08923478036424,78.08923478046424,78.10979685884524,78.10979685894524,78.13024293124174,78.13024293134174,78.15048599299033,78.15048599309033,78.17064205017553,78.17064205027553,78.1907691058396,78.19076910593961,78.21086715998254,78.21086716008254,78.23093621260435,78.23093621270435,78.25097626370503,78.25097626380503,78.2709003087212,78.2709003088212,78.31063239266899,78.310632392769,78.33038242855838,78.33038242865838,78.36979549577376,78.36979549587376,78.38916851188844,78.38916851198844,78.40851252648201,78.40851252658202,78.4276825319488,78.4276825320488,78.44682353589445,78.44682353599445,78.46590653679786,78.46590653689786,78.48490253313787,78.48490253323787,78.50386952795674,78.50386952805674,78.52280752125449,78.5228075213545,78.5416005069466,78.5416005070466,78.56033548959643,78.56033548969643,78.59774745185385,78.59774745195385,78.65374938915546,78.65374938925547,78.67233936419966,78.67233936429966,78.72799328324771,78.72799328334771,78.74640924916513,78.74640924926513,78.7647962135614,78.7647962136614,78.78312517491543,78.78312517501543,78.80139613322719,78.80139613332719,78.81963809001782,78.81963809011782,78.83785104528732,78.83785104538732,78.85580298686666,78.85580298696667,78.87360992084035,78.87360992094035,78.89135885177177,78.89135885187177,78.92682771211351,78.92682771221351,78.94451864000267,78.94451864010267,78.96212256332845,78.96212256342845,78.97966848361196,78.97966848371196,78.99709839781096,78.99709839791096,79.0319002231667,79.0319002232667,79.04912712671779,79.0491271268178,79.06623802418437,79.06623802428437,79.08323291556643,79.08323291566643,79.1171936968094,79.1171936969094,79.13410158362807,79.13410158372807,79.15086446284107,79.15086446294107,79.16759834053296,79.16759834063296,79.20097909135335,79.20097909145335,79.21759696296071,79.21759696306071,79.23412783000468,79.23412783010468,79.25062969552754,79.25062969562754,79.26698655344472,79.26698655354473,79.2833144098408,79.2833144099408,79.29961326471573,79.29961326481573,79.33212396990223,79.33212397000223,79.3482778171715,79.34827781727151,79.36434465987742,79.36434465997742,79.38038250106219,79.38038250116219,79.39639134072583,79.39639134082583,79.41237117886833,79.41237117896833,79.42814800636293,79.42814800646293,79.44389583233641,79.44389583243641,79.45955665374649,79.45955665384649,79.47513047059319,79.47513047069319,79.49058828135536,79.49058828145536,79.52141689831632,79.52141689841632,79.53675870299398,79.53675870309398,79.55189749702372,79.55189749712372,79.56686228192669,79.56686228202669,79.61169863359333,79.61169863369334,79.64148319579363,79.64148319589363,79.67118075343052,79.67118075353052,79.68597152920671,79.68597152930671,79.70073330346176,79.70073330356176,79.71543707467457,79.71543707477457,79.72990883371833,79.72990883381833,79.74435159124096,79.74435159134096,79.75873634572133,79.75873634582133,79.78744785163983,79.78744785173983,79.8017456015568,79.8017456016568,79.81598534843152,79.81598534853153,79.83019609378512,79.83019609388512,79.87279932832477,79.87279932842478,79.90110481294744,79.90110481304744,79.91519955221652,79.91519955231652,79.92920728692219,79.9292072870222,79.94315701858562,79.94315701868562,79.95704874720678,79.95704874730679,79.97091147430683,79.97091147440683,79.98462919380121,79.98462919390121,80.01200662974773,80.01200662984773,80.0256083431576,80.0256083432576,80.03918105504634,80.03918105514634,80.05255075628716,80.05255075638716,80.06583345296461,80.06583345306461,80.07905814659979,80.0790581466998,80.10547853234903,80.10547853244903,80.11858721989968,80.11858721999968,80.13157990136582,80.13157990146583,80.15753626277699,80.15753626287699,80.17047094120086,80.17047094130086,80.18323161049797,80.18323161059797,80.19596327827395,80.19596327837395,80.22139761230477,80.22139761240477,80.23401327399623,80.23401327409623,80.24657093264541,80.24657093274541,80.25909958977348,80.25909958987349,80.27157024385929,80.27157024395929,80.28401189642396,80.28401189652396,80.30880819698993,80.30880819708993,80.3210468389067,80.3210468390067,80.33319847626008,80.33319847636008,80.35738574488232,80.35738574498232,80.36936337310892,80.36936337320893,80.39328962804099,80.39328962814099,80.40520925322534,80.40520925332534,80.42896149903062,80.42896149913062,80.4762629799933,80.4762629800933,80.49975421210843,80.49975421220843,80.52307143509677,80.52307143519677,80.53467204354868,80.53467204364868,80.55781525741025,80.55781525751026,80.5808424651873,80.5808424652873,80.59229806603356,80.59229806613357,80.61518026620496,80.61518026630496,80.62657786400896,80.62657786410897,80.6492860550536,80.6492860551536,80.66056764677307,80.66056764687308,80.68310182869091,80.68310182879091,80.7055780075665,80.7055780076665,80.71674359320146,80.71674359330146,80.72779317275192,80.72779317285192,80.73881375078123,80.73881375088123,80.76082590531874,80.76082590541874,80.77178848030579,80.77178848040579,80.78272205377172,80.78272205387172,80.80450219614019,80.8045021962402,80.8153197635216,80.8153197636216,80.82607932786074,80.82607932796074,80.84748245045452,80.84748245055452,80.8580680056669,80.8580680057669,80.87918111304938,80.87918111314939,80.91073476803858,80.91073476813858,80.92120431716644,80.92120431726644,80.94211441390101,80.94211441400101,80.9525259599866,80.9525259600866,80.96282149998767,80.96282150008767,80.9730590369465,80.9730590370465,80.9830935632574,80.9830935633574,80.99309908804717,80.99309908814718,81.00301760827355,81.00301760837355,81.02273864264181,81.02273864274181,81.0323671476569,81.0323671477569,81.04190864810859,81.04190864820859,81.05130514095464,81.05130514105464,81.06067263227956,81.06067263237956,81.06998212056223,81.06998212066223,81.07926260732376,81.07926260742376,81.0884560895219,81.0884560896219,81.11600753459518,81.11600753469519,81.12511401222994,81.12511401232995,81.13416248682243,81.13416248692243,81.1431819598938,81.1431819599938,81.16119190451539,81.1611919046154,81.17012437302337,81.17012437312337,81.17899883848908,81.17899883858908,81.18784430243366,81.18784430253366,81.1965447587726,81.1965447588726,81.22258812474715,81.22258812484715,81.2312015765227,81.2312015766227,81.23978602677711,81.23978602687711,81.24834147551039,81.24834147561039,81.25683892120142,81.25683892130142,81.26530736537131,81.26530736547132,81.27368880497782,81.27368880507782,81.29880412227621,81.29880412237621,81.30712755884046,81.30712755894046,81.31536399084132,81.31536399094132,81.3318078533219,81.3318078534219,81.33995728075938,81.33995728085938,81.36437656155066,81.36437656165066,81.37246798594586,81.37246798604586,81.38053040881994,81.38053040891994,81.38853482865176,81.38853482875176,81.39651024696245,81.39651024706245,81.42838291868408,81.42838291878408,81.43630033395252,81.43630033405252,81.45201915840485,81.45201915850485,81.45976256454651,81.45976256464651,81.47522037530868,81.47522037540868,81.4906201830286,81.4906201831286,81.49824758308573,81.49824758318573,81.5058169801006,81.5058169802006,81.51335737559435,81.51335737569435,81.53594956055446,81.53594956065446,81.55094334697854,81.55094334707854,81.58084391526336,81.58084391536336,81.5956926940818,81.5956926941818,81.61045446833687,81.61045446843687,81.6177918531827,81.6177918532827,81.63971700315682,81.63971700325682,81.66143914248302,81.66143914258302,81.6758238969634,81.6758238970634,81.69012164688039,81.69012164698039,81.69722701955718,81.69722701965718,81.70430339071284,81.70430339081284,81.71842713150305,81.71842713160305,81.72544549961646,81.72544549971646,81.73942423280101,81.73942423290102,81.74635559635102,81.74635559645102,81.75325795837992,81.75325795847992,81.76703368091657,81.76703368101657,81.78075140041095,81.78075140051095,81.78753775635532,81.78753775645532,81.79429511077856,81.79429511087856,81.81453817252715,81.81453817262715,81.84142258261446,81.84142258271446,81.86149163523626,81.86149163533626,81.868132983575,81.868132983675,81.88802802707002,81.88802802717002,81.89458237084536,81.89458237094536,81.90766205687488,81.90766205697489,81.92068373986216,81.92068373996216,81.93364741980717,81.93364741990717,81.94655309670992,81.94655309680992,81.9529624328796,81.9529624329796,81.95931376600703,81.95931376610703,81.97198743074074,81.97198743084074,81.97828076082591,81.97828076092591,81.98454508938994,81.98454508948994,82.00330907356091,82.00330907366092,82.00951539908269,82.00951539918269,82.01569272308332,82.01569272318332,82.02801836956348,82.02801836966349,82.03413769052187,82.03413769062188,82.05246665187589,82.05246665197589,82.07070860866652,82.07070860876652,82.07674092506151,82.07674092516152,82.08877655633037,82.08877655643037,82.10075418455698,82.10075418465698,82.11267380974132,82.11267380984133,82.13048074371501,82.13048074381501,82.14228436281483,82.14228436291484,82.16580459645108,82.16580459655108,82.17163390219817,82.17163390229817,82.17743420642412,82.17743420652413,82.18900581335491,82.18900581345491,82.20051941724344,82.20051941734344,82.21771731927339,82.21771731937339,82.2462548160651,82.2462548161651,82.25759441082685,82.25759441092686,82.26887600254634,82.26887600264634,82.30260477162027,82.30260477172027,82.31377035725524,82.31377035735524,82.32487793984795,82.32487793994795,82.3359275193984,82.3359275194984,82.34691909590659,82.34691909600659,82.35237138187898,82.35237138197898,82.37412252272631,82.37412252282631,82.38488208706546,82.38488208716547,82.39558364836236,82.39558364846236,82.43290860605639,82.43290860615639,82.44349416126876,82.44349416136876,82.46985654397574,82.46985654407574,82.50657246972604,82.50657246982604,82.52220428961499,82.52220428971499,82.5273665603761,82.5273665604761,82.55314891266048,82.55314891276048,82.57365298809923,82.57365298819923,82.58893678973462,82.58893678983462,82.5939830544112,82.5939830545112,82.60909284691982,82.60909284701982,82.64418468748686,82.64418468758686,82.66410873250302,82.66410873260303,82.68887603154785,82.68887603164785,82.73814961594735,82.73814961604735,82.7626268997809,82.7626268998809,82.79670368710839,82.79670368720839,82.83057746378798,82.83057746388798,82.86424822981965,82.86424822991965,82.892930734217,82.892930734317,82.90717048109174,82.90717048119174,82.96386845490046,82.96386845500047,82.98733068549446,82.98733068559446,83.0153171533847,83.0153171534847,83.02920888200586,83.02920888210586,83.04762484792327,83.04762484802328,83.0888360094487,83.0888360095487,83.16621206782295,83.16621206792296,83.23857086304177,83.23857086314177,83.31496086969761,83.31496086979762,83.38639161624027,83.38639161634028,83.4928562003077,83.4928562004077,83.54572597332731,83.54572597342731,83.62890233592752,83.62890233602752,83.68977652877894,83.68977652887894,83.75024470033453,83.75024470043454,83.81030685059432,83.81030685069432,83.89980554480083,83.89980554490083,83.98446098497867,83.98446098507867,84.0475102919148,84.0475102920148,84.12265323316208,84.12265323326208,84.16409640685654,84.16409640695655,84.25054994134442,84.25054994144442,84.31594837149208,84.31594837159209,84.42148490688336,84.42148490698337,84.48595528835487,84.48595528845487,84.56597048515195,84.56597048525195,84.67719131868466,84.67719131878466,84.76393486838384,84.76393486848384,84.86178600067574,84.86178600077574,84.93170866811964,84.93170866821964,85.00110930818322,85.00110930828322,85.05850331849905,85.05850331859905,85.12306070453396,85.12306070463396,85.19843565795026,85.19843565805026,85.28819536584695,85.28819536594695,85.36983464782728,85.36983464792728,85.42137035087491,85.42137035097491,85.50173356592553,85.50173356602554,85.56695798694642,85.56695798704642,85.62446800334678,85.62446800344678,85.74215617609144,85.74215617619144,85.82350544286048,85.82350544296048,85.92524277898376,85.92524277908376,86.01569852338756,86.01569852348756,86.10194904722752,86.10194904732752,86.18405235354594,86.18405235364594,86.27224597930162,86.27224597940162,86.38659897711635,86.38659897721635,86.48662522349298,86.48662522359298,86.59900611787089,86.59900611797089,86.73006399185637,86.73006399195637,86.8534654642636,86.85346546436361,87.01761407385817,87.01761407395817,87.18666394052369,87.18666394062369,87.37946605299447,87.37946605309448,87.59868855121451,87.59868855131451,87.88725368645585,87.88725368655585,88.27149483990435,88.27149484000435,88.72519463645868,88.72519463655868,89.31934879984455,89.31934879994455,90.08403190747354,90.08403190757355,91.36192593301519,91.36192593311519]],"filenames":["genome.ctg.filtered"]}

{"coord\_y":[[16870,16870,16870,16684,16684,16131,16131,15552,15552,15029,15029,14955,14955,14538,14538,14501,14501,14210,14210,12773,12773,12462,12462,12093,12093,12041,12041,11690,11690,11572,11572,11041,11041,10876,10876,10638,10638,10499,10499,10369,10369,9721,9721,9717,9717,9552,9552,9456,9456,9360,9360,9188,9188,9142,9142,9012,9012,8816,8816,8630,8630,8608,8608,8601,8601,8558,8558,8478,8478,8442,8442,8356,8356,8246,8246,8198,8198,8138,8138,8017,8017,7944,7944,7908,7908,7896,7896,7895,7895,7794,7794,7769,7769,7689,7689,7632,7632,7628,7628,7483,7483,7464,7464,7409,7409,7360,7360,7264,7264,7233,7233,7214,7214,7158,7158,7154,7154,7085,7085,6839,6839,6836,6836,6801,6801,6790,6790,6770,6770,6767,6767,6758,6758,6704,6704,6668,6668,6650,6650,6648,6648,6639,6639,6633,6633,6559,6559,6547,6547,6502,6502,6489,6489,6483,6483,6445,6445,6415,6415,6399,6399,6337,6337,6300,6300,6262,6262,6216,6216,6182,6182,6141,6141,6119,6119,6096,6096,6056,6056,6048,6048,5981,5981,5977,5977,5967,5967,5964,5964,5941,5941,5912,5912,5908,5908,5861,5861,5841,5841,5783,5783,5760,5760,5754,5754,5741,5741,5738,5738,5686,5686,5684,5684,5683,5683,5665,5665,5610,5610,5603,5603,5571,5571,5538,5538,5523,5523,5508,5508,5501,5501,5496,5496,5487,5487,5470,5470,5466,5466,5426,5426,5420,5420,5405,5405,5365,5365,5332,5332,5320,5320,5253,5253,5230,5230,5218,5218,5214,5214,5201,5201,5196,5196,5190,5190,5184,5184,5180,5180,5150,5150,5141,5141,5095,5095,5085,5085,5059,5059,5046,5046,5043,5043,5041,5041,5026,5026,5022,5022,4989,4989,4988,4988,4935,4935,4904,4904,4891,4891,4874,4874,4830,4830,4828,4828,4820,4820,4818,4818,4809,4809,4786,4786,4779,4779,4768,4768,4758,4758,4754,4754,4744,4744,4680,4680,4662,4662,4643,4643,4633,4633,4592,4592,4590,4590,4589,4589,4586,4586,4558,4558,4557,4557,4526,4526,4520,4520,4513,4513,4511,4511,4506,4506,4488,4488,4473,4473,4461,4461,4452,4452,4430,4430,4427,4427,4406,4406,4404,4404,4392,4392,4387,4387,4381,4381,4380,4380,4353,4353,4342,4342,4336,4336,4327,4327,4317,4317,4299,4299,4297,4297,4291,4291,4275,4275,4272,4272,4253,4253,4236,4236,4186,4186,4179,4179,4178,4178,4157,4157,4154,4154,4130,4130,4119,4119,4118,4118,4108,4108,4105,4105,4104,4104,4095,4095,4087,4087,4082,4082,4080,4080,4067,4067,4038,4038,4013,4013,4012,4012,4005,4005,3995,3995,3969,3969,3938,3938,3915,3915,3906,3906,3890,3890,3889,3889,3882,3882,3854,3854,3852,3852,3844,3844,3840,3840,3826,3826,3803,3803,3774,3774,3773,3773,3766,3766,3761,3761,3741,3741,3738,3738,3730,3730,3723,3723,3683,3683,3680,3680,3677,3677,3650,3650,3638,3638,3634,3634,3597,3597,3588,3588,3571,3571,3557,3557,3553,3553,3534,3534,3527,3527,3516,3516,3513,3513,3481,3481,3471,3471,3462,3462,3453,3453,3420,3420,3415,3415,3386,3386,3385,3385,3373,3373,3364,3364,3353,3353,3336,3336,3316,3316,3304,3304,3298,3298,3292,3292,3288,3288,3287,3287,3282,3282,3274,3274,3272,3272,3265,3265,3236,3236,3208,3208,3205,3205,3193,3193,3192,3192,3183,3183,3182,3182,3180,3180,3179,3179,3166,3166,3162,3162,3144,3144,3139,3139,3138,3138,3135,3135,3133,3133,3084,3084,3074,3074,3068,3068,3041,3041,3031,3031,3003,3003,2990,2990,2987,2987,2975,2975,2959,2959,2941,2941,2938,2938,2937,2937,2932,2932,2931,2931,2930,2930,2929,2929,2923,2923,2909,2909,2908,2908,2906,2906,2901,2901,2900,2900,2899,2899,2896,2896,2893,2893,2877,2877,2876,2876,2870,2870,2859,2859,2850,2850,2848,2848,2839,2839,2832,2832,2830,2830,2828,2828,2821,2821,2804,2804,2803,2803,2799,2799,2796,2796,2794,2794,2783,2783,2779,2779,2776,2776,2761,2761,2746,2746,2726,2726,2717,2717,2700,2700,2691,2691,2689,2689,2684,2684,2676,2676,2670,2670,2664,2664,2657,2657,2652,2652,2645,2645,2619,2619,2618,2618,2615,2615,2612,2612,2601,2601,2599,2599,2592,2592,2585,2585,2583,2583,2572,2572,2570,2570,2567,2567,2566,2566,2551,2551,2543,2543,2515,2515,2489,2489,2486,2486,2482,2482,2480,2480,2477,2477,2463,2463,2449,2449,2445,2445,2444,2444,2433,2433,2431,2431,2430,2430,2423,2423,2413,2413,2409,2409,2407,2407,2402,2402,2401,2401,2373,2373,2351,2351,2345,2345,2342,2342,2340,2340,2336,2336,2335,2335,2332,2332,2326,2326,2320,2320,2313,2313,2307,2307,2301,2301,2300,2300,2293,2293,2287,2287,2286,2286,2283,2283,2281,2281,2277,2277,2271,2271,2270,2270,2260,2260,2253,2253,2248,2248,2240,2240,2233,2233,2229,2229,2222,2222,2221,2221,2218,2218,2210,2210,2199,2199,2181,2181,2169,2169,2168,2168,2157,2157,2153,2153,2151,2151,2142,2142,2141,2141,2139,2139,2137,2137,2131,2131,2127,2127,2115,2115,2108,2108,2105,2105,2102,2102,2095,2095,2074,2074,2068,2068,2063,2063,2062,2062,2061,2061,2057,2057,2053,2053,2047,2047,2026,2026,2025,2025,2023,2023,2022,2022,2021,2021,2020,2020,2014,2014,2008,2008,1998,1998,1997,1997,1996,1996,1988,1988,1986,1986,1982,1982,1978,1978,1974,1974,1954,1954,1953,1953,1950,1950,1949,1949,1942,1942,1918,1918,1914,1914,1913,1913,1910,1910,1908,1908,1907,1907,1906,1906,1892,1892,1890,1890,1888,1888,1883,1883,1881,1881,1878,1878,1877,1877,1864,1864,1859,1859,1856,1856,1851,1851,1848,1848,1841,1841,1837,1837,1830,1830,1826,1826,1809,1809,1806,1806,1797,1797,1783,1783,1782,1782,1777,1777,1772,1772,1766,1766,1755,1755,1753,1753,1747,1747,1743,1743,1739,1739,1737,1737,1734,1734,1731,1731,1728,1728,1727,1727,1726,1726,1723,1723,1720,1720,1717,1717,1712,1712,1711,1711,1709,1709,1708,1708,1707,1707,1706,1706,1705,1705,1694,1694,1681,1681,1680,1680,1677,1677,1675,1675,1672,1672,1670,1670,1667,1667,1663,1663,1659,1659,1657,1657,1653,1653,1649,1649,1647,1647,1642,1642,1640,1640,1638,1638,1637,1637,1634,1634,1630,1630,1626,1626,1625,1625,1621,1621,1619,1619,1614,1614,1608,1608,1604,1604,1603,1603,1601,1601,1590,1590,1589,1589,1577,1577,1574,1574,1567,1567,1563,1563,1560,1560,1558,1558,1552,1552,1549,1549,1548,1548,1535,1535,1528,1528,1511,1511,1506,1506,1502,1502,1500,1500,1496,1496,1495,1495,1491,1491,1490,1490,1488,1488,1486,1486,1483,1483,1477,1477,1467,1467,1466,1466,1455,1455,1454,1454,1453,1453,1447,1447,1446,1446,1442,1442,1439,1439,1435,1435,1426,1426,1424,1424,1419,1419,1416,1416,1414,1414,1408,1408,1406,1406,1403,1403,1402,1402,1391,1391,1386,1386,1379,1379,1372,1372,1371,1371,1368,1368,1362,1362,1361,1361,1359,1359,1354,1354,1350,1350,1348,1348,1344,1344,1343,1343,1340,1340,1336,1336,1334,1334,1327,1327,1326,1326,1321,1321,1319,1319,1310,1310,1302,1302,1301,1301,1299,1299,1296,1296,1295,1295,1292,1292,1291,1291,1288,1288,1286,1286,1280,1280,1276,1276,1272,1272,1269,1269,1266,1266,1258,1258,1254,1254,1249,1249,1246,1246,1241,1241,1233,1233,1232,1232,1228,1228,1224,1224,1223,1223,1220,1220,1219,1219,1218,1218,1215,1215,1207,1207,1205,1205,1203,1203,1194,1194,1193,1193,1184,1184,1183,1183,1182,1182,1178,1178,1176,1176,1170,1170,1169,1169,1165,1165,1164,1164,1155,1155,1128,1128,1126,1126,1121,1121,1118,1118,1117,1117,1116,1116,1113,1113,1107,1107,1098,1098,1096,1096,1093,1093,1092,1092,1090,1090,1089,1089,1087,1087,1082,1082,1073,1073,1067,1067,1066,1066,1063,1063,1058,1058,1056,1056,1048,1048,1042,1042,1039,1039,1035,1035,1033,1033,1030,1030,1021,1021,1019,1019,1015,1015,1014,1014,1013,1013,1009,1009,1008,1008,1005,1005,1000,1000,999,999,998,998,991,991,989,989,988,988,983,983,982,982,980,980,978,978,976,976,972,972,969,969,968,968,967,967,963,963,962,962,953,953,948,948,947,947,946,946,944,944,941,941,940,940,937,937,934,934,933,933,932,932,930,930,927,927,924,924,920,920,918,918,917,917,914,914,909,909,908,908,907,907,905,905,902,902,901,901,900,900,899,899,898,898,891,891,886,886,882,882,881,881,877,877,876,876,869,869,868,868,867,867,865,865,863,863,860,860,854,854,851,851,850,850,848,848,847,847,845,845,841,841,840,840,839,839,835,835,831,831,830,830,824,824,823,823,821,821,820,820,817,817,811,811,810,810,809,809,808,808,806,806,805,805,800,800,796,796,788,788,787,787,785,785,781,781,780,780,778,778,772,772,770,770,767,767,761,761,759,759,756,756,753,753,752,752,749,749,746,746,744,744,741,741,737,737,735,735,734,734,733,733,732,732,729,729,725,725,724,724,722,722,720,720,719,719,718,718,715,715,709,709,705,705,698,698,695,695,694,694,693,693,692,692,691,691,687,687,683,683,681,681,678,678,668,668,667,667,661,661,660,660,658,658,655,655,654,654,653,653,648,648,646,646,644,644,643,643,641,641,637,637,635,635,634,634,632,632,630,630,629,629,628,628,619,619,614,614,612,612,611,611,610,610,607,607,605,605,601,601,599,599,594,594,590,590,586,586,585,585,583,583,578,578,577,577,574,574,573,573,570,570,569,569,564,564,563,563,562,562,559,559,557,557,554,554,553,553,552,552,551,551,544,544,543,543,540,540,537,537,533,533,530,530,529,529,522,522,516,516,514,514,513,513,511,511,510,510,509,509,507,507,499,499,498,498,496,496,494,494,493,493,491,491,490,490,489,489,487,487,486,486,483,483,481,481,479,479,478,478,473,473,471,471,469,469,468,468,461,461,458,458,456,456,455,455,452,452,448,448,447,447,446,446,440,440,439,439,438,438,435,435,433,433,432,432,430,430,429,429,426,426,422,422,419,419,415,415,413,413,412,412,411,411,408,408,407,407,403,403,401,401,400,400,398,398,396,396,395,395,394,394,393,393,390,390,389,389,388,388,387,387,385,385,381,381,380,380,379,379,378,378,377,377,374,374,373,373,371,371,367,367,365,365,363,363,362,362,361,361,360,360,359,359,355,355,353,353,346,346,345,345,342,342,338,338,332,332,329,329,324,324,323,323,321,321,320,320,317,317,316,316,314,314,312,312,311,311,310,310,308,308,306,306,305,305,300,300,298,298,297,297,296,296,295,295,293,293,292,292,289,289,288,288,287,287,284,284,283,283,281,281,280,280,279,279,278,278,276,276,275,275,274,274,273,273,269,269,267,267,266,266,265,265,263,263,261,261,260,260,259,259,258,258,257,257,255,255,254,254,253,253,250,250,249,249,247,247,246,246,245,245,244,244,243,243,242,242,240,240,239,239,238,238,237,237,236,236,234,234,233,233,232,232,231,231,230,230,229,229,228,228,226,226,225,225,224,224,223,223,222,222,221,221,219,219,218,218,217,217,216,216,215,215,214,214,213,213,212,212,211,211,210,210,209,209,208,208,207,207,206,206,205,205,204,204,203,203,202,202,201,201,200,200,199,199,198,198,197,197,196,196,195,195,194,194,193,193,192,192,191,191,190,190,189,189,188,188,186,186,185,185,184,184,183,183,182,182,181,181,180,180,179,179,178,178,177,177,176,176,175,175,174,174,173,173,172,172,171,171,170,170,169,169,168,168,167,167,166,166,165,165,164,164,163,163,162,162,161,161,160,160,159,159,158,158,157,157,156,156,155,155,154,154,153,153,152,152,151,151,150,150,149,149,148,148,147,147,146,146,145,145,144,144,143,143,142,142,141,141,140,140,139,139,138,138,137,137,136,136,135,135,134,134,133,133,132,132,131,131,130,130,129,129,128,128,127,127,126,126,125,125,124,124,123,123,122,122,121,121,120,120,119,119,118,118,117,117,116,116,115,115,114,114,113,113,112,112,111,111,110,110,109,109,108,108,107,107,106,106,105,105,104,104,103,103,102,102,101,101,0.0]],"coord\_x":[[0.0,1e-10,0.6225558593503498,0.6225558594503499,1.2382477359005122,1.2382477360005122,1.8335321797167832,1.8335321798167832,2.4074497093324703,2.4074497094324703,2.9620669002142965,2.9620669003142965,3.5139532592293814,3.5139532593293814,4.0504510116440455,4.0504510117440455,4.585583348125339,4.585583348225339,5.10997687280634,5.10997687290634,5.58134059488589,5.58134059498589,6.04122744249846,6.04122744259846,6.487497033910659,6.487497034010659,6.931847662389471,6.931847662489471,7.363245291067929,7.363245291167929,7.7902883500129345,7.7902883501129345,8.197735845157405,8.197735845257405,8.599094323301708,8.599094323401708,8.99166985571244,8.99166985581244,9.379115852589695,9.379115852689695,9.761764442133488,9.761764442233488,10.120499801276628,10.120499801376628,10.479087547886428,10.479087547986428,10.831586277496063,10.831586277596063,11.180542306305602,11.180542306405602,11.525955634315043,11.525955634415043,11.865021623390977,11.865021623490977,12.202390068333532,12.202390068433532,12.534961105942623,12.534961106042623,12.860299129418182,12.860299129518182,13.178773170093553,13.178773170193553,13.49643534183557,13.49643534193557,13.813839191644245,13.813839191744245,14.129656206719543,14.129656206819543,14.442520971128095,14.442520971228095,14.754057222736611,14.754057222836611,15.062419804878372,15.062419804978372,15.366723042353357,15.366723042453357,15.669254929428293,15.669254929528293,15.969572628503169,15.969572628603169,16.265425048444587,16.265425048544586,16.558583539652602,16.5585835397526,16.850413518060577,16.850413518160575,17.141800658868544,17.141800658968542,17.43315089654317,17.43315089664317,17.720773917751035,17.720773917851034,18.007474360625537,18.007474360725535,18.291222552833293,18.29122255293329,18.57286726644099,18.57286726654099,18.854364367515352,18.85436436761535,19.130510514256233,19.13051051435623,19.40595550146376,19.40595550156376,19.679370816337904,19.679370816437903,19.95097787767866,19.95097787777866,20.21904223821932,20.219042238319318,20.485962601626618,20.485962601726616,20.75218180550056,20.752181805600557,21.01633443390778,21.016334434007778,21.280339449781664,21.280339449881662,21.541798149455477,21.541798149555476,21.79417867832904,21.794178678429038,22.046448497802604,22.046448497902603,22.297426707609464,22.297426707709462,22.547998982949647,22.547998983049645,22.79783319562314,22.79783319572314,23.047556698896635,23.047556698996633,23.296948073970118,23.296948074070116,23.544346679843546,23.544346679943544,23.790416772916938,23.790416773016936,24.035822609590312,24.03582260969031,24.28115463999702,24.281154640097018,24.526154542203717,24.526154542303715,24.770933025610404,24.770933025710402,25.012980677150356,25.012980677250354,25.254585491090292,25.25458549119029,25.494529664030186,25.494529664130184,25.97393826917662,25.97393826927662,26.21318128258316,26.21318128268316,26.451021976922995,26.451021977022993,26.6877555772628,26.687755577362797,26.923898727469254,26.923898727569252,27.15775388340898,27.157753883508978,27.390243623415333,27.39024362351533,27.62133104435498,27.62133104445498,27.850720921161255,27.850720921261253,28.078856091434155,28.078856091534153,28.30547823324035,28.30547823334035,28.53128850611319,28.531288506213187,28.756250006919338,28.756250007019336,28.979735382392114,28.979735382492112,29.202925532798215,29.202925532898213,29.423643173270914,29.423643173370913,29.644213201210274,29.644213201310272,29.864414197816295,29.864414197916293,30.08450448502231,30.08450448512231,30.52383628736765,30.52383628746765,30.742007611640283,30.74200761174028,30.960031323379575,30.960031323479573,31.176320587852153,31.17632058795215,31.391871789658044,31.391871789758042,31.605282609730544,31.605282609830542,31.817844657736355,31.817844657836353,32.03018528694216,32.03018528704216,32.242046175414615,32.242046175514616,32.45379635448707,32.45379635458707,32.66362757062614,32.66362757072614,32.87338498049854,32.873384980598544,33.0831054872376,33.083105487337605,33.292161737576656,33.29216173767666,33.49918831558231,33.49918831568231,33.70595657165463,33.70595657175463,33.91154392746025,33.91154392756025,34.11591347986583,34.11591347996583,34.31972948527141,34.31972948537141,34.52299194367696,34.522991943776965,34.72599608014918,34.72599608024918,34.92881570095472,34.928815701054724,35.13130319356026,35.13130319366026,35.333163332899105,35.333163332999106,35.53487585970462,35.53487585980462,35.73511226117676,35.73511226127676,35.935127243848896,35.9351272439489,36.13458867952101,36.13458867962101,36.33257398985976,36.33257398995976,36.529341496798466,36.52934149689847,36.72566616613717,36.72566616623717,36.91951832554247,36.91951832564247,37.112521712881076,37.11252171298108,37.30508226261967,37.30508226271967,37.49749519982493,37.497495199924934,37.68942839629685,37.68942839639685,37.88117707710209,37.88117707720209,38.07270433910732,38.07270433920732,38.264010182312546,38.26401018241255,38.45516841298444,38.455168413084444,38.645219549656304,38.645219549756305,38.83493855812816,38.83493855822816,39.21267903093848,39.21267903103848,39.40033146394361,39.40033146404361,39.58702441548205,39.58702441558205,39.773237626287134,39.773237626387136,39.95934012769222,39.959340127792224,40.14536882283064,40.14536882293064,40.33084397096904,40.330843971069044,40.51617150657411,40.51617150667411,40.70028123877914,40.70028123887914,40.884354067850836,40.88435406795084,41.066471030855816,41.06647103095582,41.247443996727434,41.247443996827435,41.4279372218657,41.4279372219657,41.60780309373728,41.60780309383728,41.78604522774215,41.78604522784215,41.964213555480356,41.96421355558036,42.14208665815188,42.142086658251884,42.319885954556746,42.31988595465675,42.497353122761595,42.4973531228616,42.673971518899755,42.67397151899976,42.85033159310458,42.85033159320458,43.026285732842716,43.02628573294272,43.20187084124752,43.20187084134752,43.55289344552378,43.55289344562378,43.7279619100619,43.7279619101619,43.90066857406662,43.90066857416662,44.07271098167132,44.07271098177132,44.24405222974267,44.24405222984267,44.41502444648068,44.41502444658068,44.584483634751976,44.58448363485198,44.7538690167566,44.753869016856605,44.9232174956279,44.923217495727904,45.0924552650992,45.0924552651992,45.26065974683713,45.26065974693713,45.42882732544172,45.428827325541725,45.59585090691295,45.59585090701295,45.76265306958418,45.762653069684184,45.92919691032206,45.929196910422064,46.09566694479328,46.09566694489328,46.26195246359783,46.26195246369783,46.42757372600235,46.42757372610235,46.59264144140687,46.59264144150687,46.75726631921137,46.75726631931137,46.92155906881585,46.921559068915855,47.08503994948699,47.08503994958699,47.24841012075812,47.248410120858125,47.41100532622924,47.41100532632924,47.57352672543367,47.573526725533675,47.735605287038105,47.73560528713811,47.89749933297586,47.897499333075864,48.059171960113616,48.05917196021362,48.220807684118036,48.22080768421804,48.381447023522426,48.38144702362243,48.54168042846014,48.54168042856014,48.701692414597844,48.701692414697845,48.86137227253555,48.86137227263555,49.0206830991399,49.0206830992399,49.179329669344234,49.179329669444236,49.337902433281904,49.337902433381906,49.496253778419565,49.496253778519566,49.65401467342387,49.654014673523875,49.811664859028184,49.811664859128186,49.968613885099145,49.96861388519915,50.12493555790341,50.124935558003415,50.27941207404097,50.27941207414097,50.58810678438274,50.58810678448274,50.74228807545362,50.742288075553624,50.89569440072448,50.89569440082448,51.04899001659534,51.04899001669534,51.20139995726617,51.20139995736617,51.35340396347033,51.35340396357033,51.505371066541144,51.505371066641146,51.80893624134944,51.808936241449445,51.96042360368692,51.96042360378692,52.26336142522853,52.26336142532853,52.414479756232666,52.41447975633267,52.56530286217012,52.56530286227012,52.7159414524409,52.7159414525409,52.86650623644502,52.86650623654502,53.01659127971579,53.01659127981579,53.16560613211986,53.165606132219864,53.313698406190575,53.31369840629058,53.46175377712795,53.461753777227955,53.60955082613199,53.609550826231995,53.75697884380269,53.756978843902694,53.903447380006696,53.9034473801067,54.04877191907733,54.048771919177334,54.193247686081286,54.19324768618129,54.337391324885225,54.33739132498523,54.48094451355581,54.480944513655814,54.62446079909307,54.62446079919307,55.0547513337715,55.0547513338715,55.19697600964205,55.19697600974205,55.33912687924594,55.33912687934594,55.48098252378315,55.48098252388315,55.62269055578702,55.62269055588702,55.76388194392421,55.76388194402421,55.90422455999472,55.90422456009472,56.04349698519852,56.043496985298525,56.182732507268994,56.182732507368996,56.4609452294766,56.4609452295766,56.59973791394706,56.59973791404706,56.737792535750835,56.737792535850836,56.875736448154605,56.87573644825461,57.0133851354917,57.013385135591705,57.150775500895456,57.15077550099546,57.28668974096583,57.286689741065835,57.42249327163621,57.422493271736215,57.55818609290659,57.55818609300659,57.69288252957694,57.69288252967694,57.827136128647275,57.82713612874728,57.96124211518428,57.96124211528428,58.0939826857879,58.0939826858879,58.22639112819152,58.226391128291525,58.35817221732846,58.35817221742846,58.48943666259871,58.489436662698715,58.62055349533563,58.62055349543563,58.75096916853919,58.750969168639195,58.88112651980942,58.88112651990942,59.010877936612964,59.010877936712966,59.14051864401651,59.14051864411651,59.26897845115335,59.26897845125335,59.5255290340937,59.5255290341937,59.653287681697186,59.65328768179719,59.78071420110067,59.780714201200674,59.906922917104126,59.90692291720413,60.0329471174409,60.0329471175409,60.157901126910986,60.15790112701099,60.28281823324773,60.28281823334773,60.407292501984465,60.407292502084466,60.53143464252119,60.53143464262119,60.65517084859124,60.65517084869124,60.90201590746466,60.90201590756466,61.02438669760134,61.02438669770134,61.146314650138,61.146314650238004,61.26802118387466,61.26802118397466,61.38950629881131,61.389506298911314,61.51084380121463,61.51084380131463,61.63214440048461,61.632144400584615,61.753260484087924,61.753260484187926,61.87408134262456,61.87408134272456,61.994828394894526,61.99482839499453,62.11531712523115,62.11531712533115,62.234735664701084,62.234735664801086,62.35312091643765,62.353120916537655,62.47139545877422,62.47139545887422,62.58922716351077,62.58922716361077,62.82485366985055,62.82485366995055,62.94231634325376,62.94231634335376,63.05974211352363,63.059742113623635,63.17709407752684,63.17709407762684,63.294409138396716,63.29440913849672,63.41124445853324,63.41124445863324,63.52793216613643,63.52793216623643,63.6439556173396,63.6439556174396,63.7597945528761,63.7597945529761,63.87559658527927,63.87559658537927,63.99128790828243,63.99128790838243,64.10690542501892,64.10690542511892,64.22071468822203,64.22071468832203,64.3341549200918,64.3341549201918,64.44737373316156,64.44737373326156,64.55959616163129,64.55959616173129,64.67144955876769,64.67144955886769,64.78226966817071,64.78226966827071,64.8926100368404,64.8926100369404,65.00283969611007,65.00283969621007,65.11262651777974,65.11262651787975,65.22182288931606,65.22182288941606,65.33035500445236,65.33035500455236,65.43877641018867,65.43877641028867,65.54716091279163,65.54716091289163,65.65536089972791,65.65536089982791,65.76352398353087,65.76352398363088,65.87165016420049,65.87165016430049,65.97973944173678,65.97973944183678,66.08760730047307,66.08760730057307,66.19495851534266,66.19495851544266,66.30227282707892,66.30227282717892,66.40951333254853,66.40951333264853,66.51656932235146,66.51656932245146,66.73064439882397,66.73064439892397,66.83762658236023,66.83762658246023,66.94449805649649,66.94449805659649,67.05125882123274,67.05125882133274,67.15742913583564,67.15742913593564,67.2635625473052,67.2635625474052,67.36947453997476,67.36947454007476,67.47498059817765,67.47498059827765,67.58015452818053,67.58015452828053,67.68525465191674,67.68525465201674,67.79002264745293,67.79002264755293,67.89453232105578,67.89453232115578,67.99896818839197,67.99896818849197,68.1033302494615,68.1033302495615,68.20743398859767,68.20743398869767,68.31091037446717,68.31091037456717,68.41434985720332,68.41434985730332,68.51764172740614,68.51764172750615,68.62082288820896,68.62082288830896,68.72393024274513,68.72393024284513,68.82663166281459,68.8266316629146,68.92918547035073,68.92918547045073,69.03162856848687,69.03162856858687,69.13351811962298,69.13351811972298,69.23485412375909,69.2348541238591,69.33545206522851,69.33545206532851,69.43571787849791,69.43571787859791,69.53535633850063,69.53535633860064,69.63466267030334,69.63466267040334,69.7338951958394,69.7338951959394,69.83294320570877,69.83294320580877,69.93169599051147,69.93169599061147,70.03022735651416,70.03022735661416,70.12853730371684,70.12853730381684,70.32489887618888,70.32489887628888,70.42276598579156,70.42276598589156,70.52037477346089,70.52037477356089,70.71463286733287,70.71463286743287,70.81124527040217,70.81124527050217,70.90774696407148,70.90774696417148,71.00413794834078,71.00413794844079,71.1001229981434,71.1001229982434,71.19603424167936,71.19603424177936,71.29168716328198,71.29168716338198,71.38708176295124,71.38708176305124,71.48240255635385,71.48240255645385,71.57731741528978,71.57731741538979,71.67215846795904,71.67215846805904,71.7668888112283,71.7668888113283,71.86158225136421,71.86158225146421,71.95572214450013,71.95572214460013,72.04956681256935,72.04956681266935,72.14237819290523,72.14237819300523,72.2342300917744,72.23423009187441,72.32597128124358,72.32597128134358,72.41756485817942,72.41756485827942,72.50908462884858,72.50908462894859,72.60049369011774,72.60049369021775,72.69138610752023,72.69138610762023,72.78176188105604,72.78176188115604,72.8719900420585,72.8719900421585,73.0524094609301,73.0524094610301,73.23238604220168,73.23238604230168,73.32209755933746,73.32209755943747,73.41177217333993,73.41177217343993,73.59086307941148,73.59086307951148,73.67991034014725,73.67991034024725,73.76880998834969,73.76880998844969,73.85763583028545,73.85763583038545,73.94627715655453,73.94627715665453,74.03488157969029,74.0348815797903,74.12245271509268,74.12245271519268,74.20921198156172,74.20921198166172,74.29574982923074,74.29574982933075,74.38217696749977,74.38217696759978,74.46853029950213,74.46853029960214,74.55473601897117,74.55473601907117,74.64090483530686,74.64090483540686,74.72696294224254,74.72696294234254,74.81279963037822,74.81279963047822,74.89841489971388,74.89841489981389,74.98377184711622,74.98377184721622,75.06890737571855,75.06890737581855,75.40922807132785,75.40922807142785,75.49410527799684,75.49410527809684,75.57872416273248,75.57872416283249,75.66312162866812,75.66312162876812,75.74748219147043,75.74748219157043,75.83173204487274,75.83173204497274,75.91590809200837,75.91590809210837,75.99993652661067,75.99993652671067,76.08374354241296,76.08374354251296,76.16751365508192,76.16751365518192,76.25091473641753,76.25091473651753,76.33405749581979,76.3340574959198,76.4170157395554,76.4170157396554,76.49967875822432,76.49967875832432,76.66474647362884,76.66474647372884,76.74700355783108,76.74700355793108,76.82900232009999,76.82900232019999,76.91096417923556,76.91096417933556,76.99281532897113,76.99281532907114,77.07437125364004,77.07437125374004,77.15552124384224,77.15552124394225,77.23600697764445,77.23600697774445,77.31604987384664,77.31604987394664,77.39605586691549,77.39605586701549,77.47565592551767,77.47565592561767,77.55510837158651,77.55510837168652,77.63448701138867,77.63448701148867,77.71353352299084,77.71353352309085,77.79254313145967,77.79254313155967,77.87147893366182,77.87147893376182,77.9503409295973,77.9503409296973,78.02898150673279,78.02898150683279,78.10747447133494,78.10747447143494,78.18552459833707,78.18552459843707,78.26331640340587,78.26331640350587,78.34099749907466,78.34099749917466,78.41856788534344,78.41856788544344,78.4958799496789,78.4958799497789,78.57241704821432,78.57241704831432,78.64873272794974,78.64873272804974,78.72486389201848,78.72486389211848,78.8009581529539,78.8009581530539,78.87701551075597,78.87701551085597,78.95292525602471,78.95292525612471,79.02868738876012,79.02868738886012,79.10422810269552,79.10422810279552,79.25453456476629,79.25453456486629,79.32926340976833,79.32926340986833,79.4039184485037,79.4039184486037,79.47853658410574,79.47853658420574,79.55311781657446,79.55311781667446,79.70224337837853,79.70224337847853,79.7765662889139,79.7765662890139,79.85066778064925,79.85066778074925,79.92440024105127,79.92440024115128,79.99809579831995,79.99809579841995,80.14545000972397,80.14545000982397,80.21881343879265,80.21881343889265,80.29210306159464,80.29210306169465,80.36524507186331,80.36524507196332,80.43823946959864,80.43823946969864,80.58408065253596,80.58408065263596,80.72903616027327,80.72903616037327,80.80110797967524,80.80110797977524,80.8730690896772,80.8730690897772,80.94499329654583,80.94499329664583,81.08858338834976,81.08858338844976,81.15936359808502,81.15936359818502,81.22999619528696,81.22999619538696,81.30059188935554,81.30059188945555,81.37107687402414,81.37107687412414,81.44148805242607,81.44148805252607,81.58227350609658,81.58227350619659,81.65261087823184,81.65261087833184,81.72243160650041,81.72243160660041,81.8619992567709,81.8619992568709,81.93167237250613,81.93167237260613,82.0011609725747,82.0011609726747,82.14006436644516,82.14006436654516,82.20936845084705,82.20936845094705,82.27863563211561,82.27863563221561,82.34742307265083,82.34742307275083,82.41602599751937,82.41602599761937,82.48451821298791,82.48451821308791,82.55282591278977,82.55282591288977,82.62102290319164,82.62102290329165,82.68896157166016,82.68896157176016,82.75675262759535,82.75675262769535,82.82428536159719,82.82428536169719,82.89167048306571,82.89167048316571,82.95842825126753,82.95842825136754,83.02507531006935,83.02507531016936,83.09139024067116,83.09139024077116,83.15718852740629,83.15718852750629,83.2229499110081,83.2229499111081,83.28852677894322,83.28852677904322,83.35391913121167,83.35391913131167,83.48448241694858,83.48448241704858,83.6144183494188,83.6144183495188,83.6791095421539,83.6791095422539,83.743579316089,83.743579316189,83.80790147749074,83.80790147759075,83.87207602635917,83.87207602645917,83.93617676896092,83.93617676906092,84.00016680216267,84.00016680226267,84.12803615916617,84.12803615926617,84.19180477356791,84.19180477366791,84.31930509923806,84.31930509933807,84.38299990737313,84.38299990747313,84.44658400610821,84.44658400620821,84.51005739544327,84.51005739554327,84.57342007537834,84.57342007547834,84.63659823964673,84.63659823974673,84.69973950078179,84.69973950088179,84.76280695565018,84.76280695575018,84.82583750738524,84.82583750748525,84.88883115598696,84.88883115608697,84.95178790145535,84.95178790155535,85.0147077437904,85.0147077438904,85.07722165165877,85.07722165175878,85.1392558187938,85.1392558188938,85.2012530827955,85.2012530828955,85.32513690139889,85.3251369014989,85.38694964973391,85.38694964983391,85.51046443700395,85.51046443710395,85.5720926696723,85.5720926697723,85.63361019294065,85.63361019304065,85.69498010367566,85.69498010377566,85.75620240187733,85.75620240197733,85.81735089381235,85.81735089391235,85.878351773214,85.87835177331401,85.93920504008234,85.93920504018234,85.999984500684,85.999984500784,86.06057944561898,86.06057944571899,86.1211005842873,86.1211005843873,86.18154791668896,86.18154791678896,86.24195834595729,86.24195834605729,86.36266849509391,86.36266849519392,86.42282060242889,86.42282060252889,86.48282509723053,86.48282509733053,86.54279268889884,86.54279268899884,86.6026126680338,86.6026126681338,86.6623588409021,86.6623588410021,86.72192049810373,86.72192049820373,86.78126073650536,86.78126073660536,86.84045336237364,86.84045336247364,86.8996090851086,86.8996090852086,87.01784672431182,87.01784672441183,87.07652270631343,87.07652270641343,87.1351617851817,87.1351617852817,87.19335802644996,87.19335802654996,87.2514435583182,87.2514435584182,87.30927076825311,87.30927076835312,87.3669503656547,87.3669503657547,87.42451925365627,87.42451925375627,87.53958322339275,87.53958322349276,87.59685688632764,87.59685688642764,87.65401983986254,87.65401983996254,87.7111458902641,87.71114589036411,87.76779219993232,87.76779220003232,87.8808264973354,87.88082649743541,87.9365871318036,87.93658713190361,87.99216325060512,87.99216325070512,88.0475917568733,88.0475917569733,88.10294645687482,88.10294645697482,88.158153544343,88.158153544443,88.21332372867784,88.21332372877784,88.26834630047934,88.26834630057934,88.3233319691475,88.3233319692475,88.43322950021718,88.43322950031718,88.48806755635201,88.48806755645201,88.59763295922167,88.59763295932167,88.6521388871565,88.6521388872565,88.7607817116928,88.7607817117928,88.81488170516094,88.81488170526094,88.8685757641624,88.8685757642624,88.97592697903201,88.97592697913201,89.02954723176681,89.02954723186681,89.0829460657016,89.08294606580161,89.13630799650306,89.13630799660307,89.18952231477118,89.18952231487118,89.2426259236393,89.2426259237393,89.29558191997408,89.29558192007408,89.34820578810886,89.34820578820886,89.40075584997696,89.40075585007696,89.45312139617839,89.45312139627839,89.55774177918124,89.55774177928124,89.60992280971601,89.60992280981601,89.66188242145076,89.66188242155076,89.71376822691884,89.71376822701885,89.76554332298693,89.76554332308693,89.81728151592168,89.81728151602168,89.86861377438974,89.86861377448975,89.91976151719113,89.91976151729114,89.9706509380592,89.9706509381592,90.02128203699391,90.02128203709391,90.0718762327953,90.0718762328953,90.12235971919668,90.12235971929668,90.17262178679805,90.17262178689805,90.22284695126609,90.2228469513661,90.27299830946747,90.27299830956747,90.32296515200215,90.32296515210216,90.37278438200352,90.37278438210352,90.42252980573822,90.42252980583822,90.47212761693957,90.47212761703958,90.57128633620894,90.57128633630894,90.62073653487697,90.62073653497697,90.67003912101164,90.67003912111164,90.71926790087966,90.71926790097966,90.81746713868235,90.81746713878235,90.96434160935303,90.96434160945303,91.01309064848769,91.01309064858769,91.11051492049036,91.11051492059036,91.15885802515834,91.15885802525834,91.25524900942764,91.25524900952765,91.30325998589562,91.30325998599562,91.35119715609693,91.35119715619693,91.39902361689823,91.39902361699824,91.44681317456622,91.44681317466622,91.49449202283418,91.49449202293418,91.58981281623679,91.58981281633679,91.68498599710605,91.68498599720606,91.73244342657402,91.73244342667402,91.77967943724198,91.77967943734198,91.8267678353766,91.8267678354766,91.87370862097788,91.87370862107788,91.92053869717917,91.92053869727917,91.96725806398044,91.96725806408044,92.01368220571504,92.01368220581504,92.05995873491631,92.05995873501631,92.1060507484509,92.1060507485509,92.15203205258548,92.15203205268548,92.19782884105341,92.19782884115341,92.24333040445465,92.24333040455465,92.28879506472256,92.28879506482257,92.33411211245713,92.33411211255714,92.37928154765837,92.37928154775837,92.42441407972626,92.42441407982626,92.46943590239417,92.46943590249417,92.51442082192872,92.51442082202873,92.64933867739909,92.64933867749909,92.6941759844003,92.6941759845003,92.73871806633485,92.73871806643486,92.78318634200274,92.78318634210274,92.82758081140395,92.82758081150395,92.91603762200637,92.91603762210637,92.96006306007425,92.96006306017425,93.0037563699421,93.0037563700421,93.04741277667662,93.04741277677662,93.09103228027782,93.09103228037782,93.17812367494687,93.17812367504688,93.22152175974806,93.22152175984806,93.26469842574923,93.26469842584923,93.30783818861708,93.30783818871708,93.39397010181943,93.39397010191944,93.43692534902061,93.43692534912061,93.47954846802178,93.47954846812178,93.52117520242291,93.52117520252291,93.56272813055737,93.56272813065738,93.64564947115964,93.64564947125965,93.68690717422744,93.68690717432744,93.72812797416191,93.72812797426191,93.76931187096302,93.76931187106302,93.81038505836415,93.81038505846415,93.89231001436639,93.89231001446639,93.97368142336862,93.97368142346862,94.01412725750305,94.01412725760305,94.05446238223749,94.05446238233749,94.0947606038386,94.0947606039386,94.17528324077412,94.17528324087412,94.21547075297522,94.21547075307522,94.25558445890965,94.25558445900965,94.29551364917741,94.29551364927741,94.33511071124516,94.33511071134517,94.3744863545129,94.3744863546129,94.41382509464731,94.41382509474731,94.45305312538171,94.45305312548172,94.53132467118385,94.53132467128385,94.57029437998492,94.57029438008492,94.64793857252037,94.64793857262038,94.72506612118916,94.72506612128916,94.76340847672353,94.76340847682353,94.83994557525895,94.83994557535895,94.91626125499438,94.91626125509438,94.95427148232875,94.95427148242875,94.99194958146312,94.99194958156312,95.0295538743308,95.0295538744308,95.06701055466516,95.06701055476516,95.10443033186618,95.10443033196619,95.1792329831349,95.1792329832349,95.21646824466924,95.21646824476925,95.25366660307027,95.25366660317027,95.29075425207128,95.29075425217128,95.32765738540563,95.32765738550563,95.36452361560663,95.36452361570663,95.40135294267431,95.40135294277431,95.43792394780864,95.43792394790864,95.4744211466763,95.47442114677631,95.51088144241064,95.51088144251064,95.54715722247829,95.54715722257829,95.61967187948026,95.61967187958027,95.65583695014793,95.65583695024793,95.69192821454891,95.69192821464891,95.72794567268323,95.72794567278324,95.7638155182842,95.7638155183842,95.79957465448518,95.79957465458519,95.83529688755283,95.83529688765283,95.87098221748714,95.87098221758714,95.90651993488811,95.90651993498811,95.94202074915574,95.94202074925575,95.97718943522338,95.97718943532338,96.01217360562433,96.01217360572433,96.04712087289195,96.04712087299195,96.11697850429387,96.11697850439387,96.18672542629577,96.18672542639577,96.22145127476338,96.22145127486338,96.25614022009766,96.25614022019766,96.32540740136623,96.32540740146624,96.3598749279005,96.35987492800051,96.39430555130144,96.39430555140144,96.42869927156904,96.42869927166905,96.46301918556999,96.46301918566999,96.49722839017092,96.49722839027092,96.53132688537185,96.53132688547186,96.56527776803945,96.56527776813945,96.59915484444038,96.59915484454038,96.63299501770797,96.63299501780797,96.66672448157556,96.66672448167556,96.73399889364406,96.73399889374406,96.76750693871165,96.76750693881165,96.80097808064589,96.80097808074589,96.83437541631348,96.83437541641348,96.86766204258105,96.86766204268105,96.90091176571529,96.9009117658153,96.9341245857162,96.9341245858162,96.96730050258377,96.96730050268377,97.00043951631801,97.00043951641801,97.03332020811891,97.03332020821891,97.06601638425313,97.06601638435313,97.09856494785403,97.09856494795403,97.13107660832158,97.13107660842158,97.1634406562558,97.1634406563558,97.19576780105669,97.19576780115669,97.22783662392423,97.22783662402423,97.25986854365844,97.25986854375844,97.32389547999352,97.32389548009353,97.3878117069286,97.3878117070286,97.41965911099614,97.41965911109614,97.45139580566367,97.45139580576367,97.4829110815312,97.4829110816312,97.51431564799873,97.51431564809873,97.54568331133291,97.54568331143291,97.60834483173463,97.60834483183463,97.70218949980386,97.70218949990387,97.73337264747138,97.73337264757139,97.76440818260556,97.76440818270557,97.79540681460641,97.79540681470641,97.82636854347392,97.82636854357392,97.8571826598081,97.8571826599081,97.88784916360893,97.88784916370894,97.91847876427644,97.91847876437645,97.94888694614393,97.94888694624393,97.9792582248781,97.9792582249781,98.00955569734559,98.00955569744559,98.03981626667975,98.03981626677975,98.0699661266139,98.06996612671391,98.09989456774807,98.09989456784807,98.12978610574888,98.12978610584888,98.15964074061637,98.15964074071637,98.18945847235051,98.18945847245051,98.24902012955214,98.24902012965214,98.27872715188629,98.27872715198629,98.3379566808879,98.3379566809879,98.39685408168951,98.39685408178951,98.42593375075698,98.42593375085698,98.4549765166911,98.4549765167911,98.48394547635856,98.48394547645856,98.51276682349268,98.51276682359268,98.59919396176171,98.59919396186172,98.62790459949584,98.62790459959584,98.65639381842995,98.65639381852995,98.68480923109739,98.68480923119739,98.71311393436483,98.71311393446483,98.74119721883226,98.74119721893226,98.76920669703303,98.76920669713303,98.79710546583378,98.79710546593378,98.82489352523454,98.82489352533455,98.85264468150197,98.85264468160197,98.8802851283694,98.8802851284694,98.90781486583681,98.90781486593681,98.93527079703756,98.93527079713756,98.96261601883832,98.96261601893832,98.98981362810572,98.98981362820572,99.01693743110647,99.01693743120647,99.04402433097387,99.04402433107387,99.07107432770795,99.07107432780795,99.12513741804275,99.12513741814276,99.1520398022435,99.1520398023435,99.20569695811162,99.20569695821162,99.23241482664568,99.23241482674568,99.25905888891307,99.25905888901308,99.28562914491381,99.28562914501381,99.3121624977812,99.3121624978812,99.33865894751526,99.33865894761526,99.3650446878493,99.36504468794931,99.39120900938336,99.39120900948336,99.41722571838407,99.41722571848408,99.44298410545144,99.44298410555145,99.46863178311881,99.46863178321881,99.49424255765284,99.49424255775284,99.51981642905355,99.51981642915355,99.54535339732091,99.54535339742091,99.57085346245493,99.57085346255494,99.59620591505563,99.59620591515564,99.64676320772368,99.64676320782368,99.67189424152437,99.67189424162437,99.72204559972573,99.72204559982573,99.74669689279308,99.74669689289308,99.77131128272708,99.77131128282709,99.79570425386109,99.79570425396109,99.82006032186175,99.82006032196175,99.84434258359575,99.84434258369575,99.86851413592974,99.86851413602975,99.8926487851304,99.8926487852304,99.91674653119773,99.91674653129773,99.94065976159838,99.94065976169838,99.96449918573236,99.96449918583237,100.01210422773366,100.01210422783366,100.08336417820227,100.08336417830228,100.1070190866696,100.1070190867696,100.17783619953819,100.1778361996382,100.2012696892055,100.2012696893055,100.22466627573948,100.22466627583948,100.24798905600677,100.24798905610677,100.27123803000741,100.27123803010741,100.29445010087471,100.29445010097471,100.31762526860868,100.31762526870868,100.34046830814263,100.34046830824263,100.36312683200993,100.36312683210993,100.38571154961055,100.38571154971055,100.43084408167844,100.43084408177845,100.4533549930124,100.4533549931124,100.47575519494633,100.47575519504633,100.49808159061361,100.49808159071361,100.52026037374755,100.52026037384755,100.56454413374877,100.56454413384877,100.58646459494936,100.58646459504936,100.60823744361663,100.60823744371663,100.62986267975054,100.62986267985055,100.67307624888507,100.67307624898507,100.69459077561899,100.69459077571899,100.71592078668624,100.71592078678624,100.73721389462015,100.73721389472016,100.77968940108798,100.77968940118798,100.80083489648855,100.80083489658855,100.82186968248914,100.82186968258914,100.84286756535637,100.84286756545637,100.86368093255695,100.86368093265695,100.88445739662417,100.88445739672417,100.90519695755808,100.90519695765808,100.94656537002588,100.94656537012588,100.9671204152931,100.9671204153931,100.98756475116032,100.98756475126032,101.00797218389422,101.00797218399423,101.02834271349478,101.02834271359478,101.048676339962,101.048676340062,101.06875164449588,101.06875164459588,101.08879004589643,101.08879004599643,101.10871773789697,101.10871773799697,101.12853472049751,101.12853472059751,101.14820409056472,101.14820409066472,101.18743212129912,101.18743212139913,101.20695387883299,101.20695387893299,101.22621731443351,101.22621731453351,101.24525933123404,101.24525933133404,101.30231157536893,101.30231157546893,101.3402110933033,101.3402110934033,101.37799990183767,101.37799990193767,101.39682049983819,101.39682049993819,101.41560419470537,101.41560419480537,101.43431408330588,101.43431408340588,101.45272874683971,101.45272874693971,101.47110650724021,101.47110650734021,101.48941046137405,101.48941046147405,101.52594456337505,101.52594456347505,101.54413780810887,101.54413780820887,101.56225724657604,101.56225724667604,101.58033978190986,101.58033978200986,101.63455048477802,101.63455048487802,101.67056794291233,101.67056794301233,101.68850286571282,101.68850286581282,101.70632707911331,101.70632707921331,101.72407748624713,101.72407748634713,101.74175408711427,101.74175408721428,101.75939378484809,101.75939378494809,101.77684896691524,101.77684896701524,101.81168552478286,101.81168552488286,101.82899309431666,101.82899309441666,101.84626376071714,101.84626376081714,101.86327610518427,101.86327610528427,101.8801777402514,101.8801777403514,101.89700556905186,101.89700556915186,101.93062432351944,101.93062432361944,101.94730453978656,101.94730453988656,101.96383714352035,101.96383714362035,101.99686544785457,101.99686544795458,102.0133242453217,102.0133242454217,102.02956162398881,102.02956162408881,102.04576209952259,102.04576209962259,102.0781261474568,102.0781261475568,102.09417901045724,102.09417901055724,102.11015806719101,102.11015806729101,102.12610022079144,102.12610022089144,102.14196856812521,102.14196856822521,102.15780001232565,102.15780001242565,102.1893521913265,102.18935219142651,102.2049253135936,102.20492531369361,102.22038772646069,102.22038772656069,102.25116493966154,102.25116493976154,102.26640593372862,102.26640593382862,102.29685101872944,102.29685101882944,102.31201820652986,102.31201820662986,102.34224187273068,102.34224187283068,102.402430883199,102.402430883299,102.43232242119981,102.43232242129982,102.46199254040063,102.46199254050063,102.47675379373436,102.47675379383436,102.50620249413517,102.50620249423517,102.53550358200263,102.53550358210263,102.5500803196697,102.5500803197697,102.5791968918705,102.5791968919705,102.5936998232709,102.5936998233709,102.62259497667168,102.62259497677168,102.63695029553874,102.63695029563874,102.66562403013953,102.66562403023953,102.69422395847364,102.69422395857364,102.70843166480736,102.70843166490737,102.72249175860775,102.72249175870775,102.73651494927479,102.7365149493748,102.76452442747556,102.76452442757557,102.77847381187594,102.77847381197594,102.792386293143,102.792386293243,102.82010054627709,102.82010054637709,102.83386541501079,102.8338654151108,102.84755647747784,102.84755647757784,102.87479098987858,102.87479098997858,102.88826063354561,102.88826063364561,102.91512611461302,102.91512611471302,102.95527672368078,102.95527672378078,102.96859875481448,102.96859875491448,102.99520591394854,102.99520591404854,103.00845413881557,103.00845413891557,103.02155475114925,103.02155475124925,103.03458155721628,103.03458155731629,103.04735004134996,103.04735004144996,103.06008162235031,103.06008162245031,103.07270249395066,103.07270249405066,103.09779662461801,103.09779662471801,103.11004846488501,103.11004846498501,103.122189595752,103.122189595852,103.13414621095234,103.13414621105234,103.14606592301932,103.14606592311932,103.15791182881965,103.15791182891965,103.16972083148664,103.16972083158664,103.18141912475363,103.18141912485363,103.21647710142125,103.21647710152125,103.22806468528823,103.22806468538823,103.23957846288855,103.23957846298855,103.25105533735552,103.25105533745553,103.27397218315616,103.27397218325616,103.28533834822314,103.28533834832314,103.29663070702344,103.29663070712344,103.30788616269041,103.30788616279041,103.31895710269072,103.31895710279072,103.35209611642496,103.35209611652496,103.36305634702526,103.36305634712527,103.37397967449222,103.37397967459222,103.38486609882585,103.38486609892585,103.39567871689282,103.39567871699282,103.40645443182645,103.40645443192645,103.41711943736007,103.41711943746007,103.44907755082761,103.44907755092761,103.45966875009456,103.45966875019457,103.47014923996151,103.47014924006152,103.4910733165621,103.4910733166621,103.50144309702904,103.50144309712904,103.53251553529655,103.53251553539656,103.54281150949684,103.54281150959685,103.55307058056378,103.55307058066379,103.56325584536407,103.56325584546407,103.573404207031,103.573404207131,103.61396075056545,103.61396075066546,103.62403530596572,103.62403530606572,103.64403680423294,103.64403680433294,103.65388994083321,103.65388994093321,103.67355931090042,103.67355931100042,103.69315487470095,103.69315487480095,103.70286039876788,103.70286039886788,103.71249211656814,103.71249211666814,103.72208693123507,103.72208693133507,103.75083447210253,103.75083447220253,103.76991339203637,103.76991339213637,103.80796052250409,103.80796052260409,103.82685492677126,103.82685492687126,103.84563862163844,103.84563862173844,103.85497511437204,103.85497511447204,103.88287388317279,103.8828738832728,103.91051433004021,103.91051433014022,103.92881828417406,103.92881828427406,103.94701152890788,103.94701152900788,103.9560527965748,103.9560527966748,103.96505716110838,103.96505716120838,103.9830289870422,103.9830289871422,103.99195954530911,103.99195954540912,104.00974685557627,104.00974685567627,104.01856670444317,104.01856670454318,104.02734965017675,104.02734965027675,104.04487863851055,104.04487863861056,104.0623338205777,104.06233382067771,104.07096915377794,104.07096915387794,104.07956758384483,104.07956758394484,104.10532597091222,104.10532597101222,104.13953517551315,104.13953517561315,104.1650721437805,104.16507214388051,104.17352296131408,104.17352296141408,104.19883851078143,104.19883851088143,104.207178618915,104.207178619015,104.22382193204878,104.22382193214878,104.2403914389159,104.24039143901591,104.25688713951635,104.25688713961635,104.27330903385014,104.27330903395014,104.28146462631703,104.28146462641703,104.28954641251725,104.28954641261726,104.30567308178435,104.30567308188435,104.3136810617179,104.3136810618179,104.32165213851812,104.32165213861812,104.34552846578545,104.34552846588545,104.353425736319,104.353425736419,104.36128610371921,104.36128610381921,104.3769699353863,104.3769699354863,104.38475649651986,104.38475649661986,104.40807927678715,104.40807927688715,104.43129134765445,104.43129134775445,104.438967199388,104.438967199488,104.45428199972174,104.45428199982175,104.46952299378883,104.46952299388883,104.48469018158924,104.48469018168925,104.50734870545654,104.50734870555654,104.52236828072361,104.52236828082361,104.55229672185776,104.55229672195776,104.55971425165797,104.55971425175797,104.56709487832484,104.56709487842484,104.58181922852523,104.58181922862524,104.59646977245897,104.59646977255898,104.61835333052623,104.61835333062623,104.65466601372722,104.65466601382722,104.66909513886095,104.66909513896096,104.68345045772801,104.68345045782802,104.72636880179586,104.72636880189586,104.74057650812958,104.74057650822958,104.75471040819663,104.75471040829663,104.76877050199701,104.76877050209701,104.78275678953072,104.78275678963072,104.78969457859759,104.78969457869759,104.81737192859835,104.81737192869835,104.83106299106538,104.83106299116538,104.84468024726576,104.84468024736576,104.89217457986705,104.89217457996705,104.90564422353408,104.90564422363408,104.93918917173501,104.93918917183501,104.98590853853628,104.98590853863628,105.0057993274035,105.0057993275035,105.012368085137,105.012368085237,105.04517497067124,105.04517497077124,105.07126548593861,105.07126548603861,105.0907134372058,105.09071343730581,105.09713458240599,105.09713458250599,105.11636111487319,105.11636111497319,105.16101390620773,105.16101390630773,105.18636635880843,105.18636635890843,105.21788163467596,105.21788163477596,105.280580058211,105.280580058311,105.31172630274519,105.31172630284519,105.35508748441303,105.35508748451304,105.39819034414755,105.39819034424755,105.44103488194872,105.44103488204873,105.47753208081639,105.47753208091639,105.49565151928354,105.49565151938354,105.56779714495218,105.56779714505218,105.59765177981967,105.59765177991967,105.63326330348731,105.63326330358731,105.65093990435446,105.65093990445446,105.67437339402177,105.67437339412177,105.72681274648987,105.72681274658987,105.82527030622589,105.82527030632589,105.91734362389508,105.91734362399508,106.01454647709772,106.01454647719773,106.10543889450021,106.10543889460021,106.24091029697058,106.24091029707058,106.3081847090391,106.3081847091391,106.41402289544199,106.41402289554199,106.49148257231077,106.49148257241077,106.56842560531287,106.56842560541287,106.64485199444829,106.64485199454829,106.75873506391807,106.75873506401807,106.86645531012101,106.86645531022101,106.94668272198987,106.94668272208988,107.04229874045916,107.04229874055916,107.09503331799394,107.09503331809394,107.2050415584636,107.20504155856361,107.28825812413254,107.28825812423254,107.42254862633622,107.42254862643622,107.50458429173845,107.50458429183846,107.60640003660791,107.60640003670791,107.74792355294511,107.74792355304511,107.85830082474813,107.85830082484813,107.9828119966182,107.9828119967182,108.0717854510873,108.0717854511873,108.16009464915638,108.16009464925638,108.23312595002504,108.23312595012504,108.31527232482729,108.31527232492729,108.41118356836324,108.41118356846324,108.52539876603302,108.52539876613302,108.6292810863692,108.6292810864692,108.69485795430433,108.69485795440433,108.79711653677379,108.79711653687379,108.88011168364272,108.88011168374273,108.95329059704473,108.95329059714473,109.10304351211548,109.10304351221548,109.20655680111831,109.20655680121831,109.33601299285519,109.33601299295519,109.45111386572499,109.451113865825,109.56086378426133,109.56086378436133,109.66533655473086,109.66533655483086,109.77755898320059,109.7775589833006,109.9230680379379,109.9230680380379,110.05034694480804,110.05034694490804,110.19334658647861,110.19334658657861,110.36011184601651,110.36011184611651,110.51713467835414,110.51713467845414,110.72600641302651,110.72600641312651,110.94111477723239,110.94111477733239,111.1864468076391,111.1864468077391,111.46539759251338,111.46539759261339,111.8325837691901,111.8325837692901,112.32151338273678,112.32151338283678,112.89882600061924,112.89882600071924,113.6548604932399,113.6548604933399,114.6278854098665,114.6278854099665,116.2539481739776,116.2539481740776]],"filenames":["genome.ctg.filtered"]}

{"coord\_y":[[16862,16862,16862,15551,15551,15264,15264,14955,14955,14538,14538,14034,14034,12773,12773,12093,12093,12041,12041,11520,11520,11041,11041,10638,10638,10369,10369,9768,9768,9704,9704,9360,9360,9327,9327,9142,9142,9113,9113,9012,9012,8938,8938,8630,8630,8608,8608,8558,8558,8478,8478,8356,8356,8246,8246,8063,8063,7944,7944,7908,7908,7896,7896,7871,7871,7689,7689,7632,7632,7628,7628,7464,7464,7458,7458,7409,7409,7400,7400,7338,7338,7261,7261,7233,7233,7214,7214,7158,7158,6790,6790,6758,6758,6662,6662,6650,6650,6639,6639,6633,6633,6592,6592,6547,6547,6502,6502,6489,6489,6445,6445,6347,6347,6337,6337,6300,6300,6262,6262,6229,6229,6182,6182,6056,6056,6048,6048,5991,5991,5981,5981,5967,5967,5964,5964,5941,5941,5912,5912,5841,5841,5831,5831,5783,5783,5760,5760,5754,5754,5741,5741,5738,5738,5734,5734,5684,5684,5610,5610,5603,5603,5523,5523,5508,5508,5487,5487,5466,5466,5457,5457,5448,5448,5440,5440,5423,5423,5391,5391,5365,5365,5332,5332,5320,5320,5290,5290,5278,5278,5253,5253,5188,5188,5184,5184,5141,5141,5132,5132,5117,5117,5116,5116,5110,5110,5095,5095,5085,5085,5050,5050,5046,5046,5043,5043,5041,5041,5026,5026,5022,5022,4990,4990,4989,4989,4974,4974,4904,4904,4891,4891,4830,4830,4828,4828,4823,4823,4820,4820,4810,4810,4809,4809,4786,4786,4768,4768,4758,4758,4754,4754,4744,4744,4711,4711,4678,4678,4662,4662,4630,4630,4589,4589,4586,4586,4553,4553,4526,4526,4520,4520,4513,4513,4511,4511,4506,4506,4488,4488,4487,4487,4473,4473,4461,4461,4452,4452,4430,4430,4406,4406,4404,4404,4392,4392,4387,4387,4353,4353,4342,4342,4336,4336,4327,4327,4317,4317,4270,4270,4236,4236,4189,4189,4181,4181,4179,4179,4157,4157,4154,4154,4119,4119,4108,4108,4104,4104,4094,4094,4080,4080,4079,4079,4074,4074,4067,4067,4043,4043,4005,4005,3995,3995,3953,3953,3920,3920,3915,3915,3906,3906,3890,3890,3889,3889,3882,3882,3844,3844,3840,3840,3826,3826,3803,3803,3774,3774,3773,3773,3766,3766,3763,3763,3761,3761,3749,3749,3741,3741,3737,3737,3730,3730,3723,3723,3683,3683,3680,3680,3640,3640,3634,3634,3606,3606,3597,3597,3581,3581,3572,3572,3571,3571,3553,3553,3546,3546,3534,3534,3527,3527,3516,3516,3481,3481,3471,3471,3462,3462,3453,3453,3420,3420,3413,3413,3394,3394,3386,3386,3355,3355,3353,3353,3351,3351,3338,3338,3336,3336,3316,3316,3306,3306,3304,3304,3298,3298,3291,3291,3287,3287,3274,3274,3272,3272,3251,3251,3236,3236,3208,3208,3206,3206,3205,3205,3198,3198,3193,3193,3183,3183,3182,3182,3180,3180,3179,3179,3173,3173,3166,3166,3162,3162,3144,3144,3139,3139,3138,3138,3135,3135,3133,3133,3099,3099,3084,3084,3068,3068,3031,3031,3003,3003,2990,2990,2987,2987,2975,2975,2958,2958,2941,2941,2938,2938,2931,2931,2928,2928,2908,2908,2901,2901,2900,2900,2899,2899,2896,2896,2893,2893,2874,2874,2872,2872,2859,2859,2850,2850,2839,2839,2832,2832,2830,2830,2828,2828,2821,2821,2805,2805,2804,2804,2803,2803,2799,2799,2796,2796,2784,2784,2776,2776,2761,2761,2754,2754,2735,2735,2726,2726,2700,2700,2689,2689,2684,2684,2671,2671,2664,2664,2652,2652,2645,2645,2643,2643,2625,2625,2619,2619,2615,2615,2612,2612,2591,2591,2587,2587,2585,2585,2572,2572,2570,2570,2566,2566,2560,2560,2543,2543,2526,2526,2523,2523,2515,2515,2489,2489,2486,2486,2482,2482,2477,2477,2470,2470,2460,2460,2449,2449,2445,2445,2444,2444,2442,2442,2433,2433,2430,2430,2424,2424,2423,2423,2413,2413,2407,2407,2401,2401,2383,2383,2373,2373,2351,2351,2336,2336,2332,2332,2326,2326,2320,2320,2307,2307,2300,2300,2291,2291,2287,2287,2286,2286,2283,2283,2277,2277,2275,2275,2271,2271,2253,2253,2248,2248,2240,2240,2238,2238,2233,2233,2229,2229,2222,2222,2221,2221,2218,2218,2210,2210,2199,2199,2169,2169,2157,2157,2153,2153,2151,2151,2141,2141,2139,2139,2137,2137,2131,2131,2127,2127,2115,2115,2108,2108,2105,2105,2095,2095,2074,2074,2068,2068,2066,2066,2063,2063,2057,2057,2053,2053,2047,2047,2026,2026,2023,2023,2022,2022,2021,2021,2020,2020,2014,2014,2008,2008,1997,1997,1996,1996,1991,1991,1988,1988,1986,1986,1981,1981,1978,1978,1974,1974,1954,1954,1952,1952,1949,1949,1942,1942,1925,1925,1916,1916,1913,1913,1911,1911,1908,1908,1907,1907,1906,1906,1891,1891,1890,1890,1889,1889,1888,1888,1883,1883,1881,1881,1877,1877,1864,1864,1859,1859,1854,1854,1851,1851,1848,1848,1844,1844,1842,1842,1841,1841,1834,1834,1830,1830,1826,1826,1809,1809,1806,1806,1797,1797,1783,1783,1782,1782,1777,1777,1772,1772,1766,1766,1753,1753,1747,1747,1743,1743,1739,1739,1737,1737,1734,1734,1731,1731,1728,1728,1727,1727,1721,1721,1720,1720,1717,1717,1711,1711,1709,1709,1708,1708,1707,1707,1706,1706,1705,1705,1694,1694,1681,1681,1680,1680,1677,1677,1676,1676,1672,1672,1666,1666,1662,1662,1657,1657,1656,1656,1653,1653,1649,1649,1647,1647,1642,1642,1640,1640,1638,1638,1637,1637,1634,1634,1630,1630,1626,1626,1625,1625,1621,1621,1616,1616,1608,1608,1604,1604,1603,1603,1601,1601,1589,1589,1586,1586,1576,1576,1574,1574,1566,1566,1563,1563,1560,1560,1552,1552,1548,1548,1547,1547,1533,1533,1530,1530,1504,1504,1500,1500,1496,1496,1495,1495,1490,1490,1489,1489,1486,1486,1483,1483,1477,1477,1466,1466,1465,1465,1455,1455,1447,1447,1446,1446,1440,1440,1439,1439,1435,1435,1424,1424,1419,1419,1415,1415,1413,1413,1408,1408,1406,1406,1403,1403,1402,1402,1388,1388,1386,1386,1379,1379,1372,1372,1370,1370,1362,1362,1361,1361,1359,1359,1353,1353,1348,1348,1344,1344,1343,1343,1336,1336,1334,1334,1327,1327,1326,1326,1321,1321,1319,1319,1310,1310,1301,1301,1299,1299,1296,1296,1292,1292,1291,1291,1289,1289,1288,1288,1286,1286,1276,1276,1272,1272,1266,1266,1265,1265,1263,1263,1256,1256,1246,1246,1241,1241,1240,1240,1233,1233,1232,1232,1228,1228,1224,1224,1223,1223,1219,1219,1218,1218,1217,1217,1207,1207,1203,1203,1194,1194,1193,1193,1187,1187,1184,1184,1182,1182,1178,1178,1169,1169,1168,1168,1165,1165,1164,1164,1158,1158,1155,1155,1153,1153,1125,1125,1121,1121,1118,1118,1117,1117,1116,1116,1113,1113,1107,1107,1097,1097,1096,1096,1094,1094,1093,1093,1092,1092,1089,1089,1087,1087,1082,1082,1073,1073,1058,1058,1056,1056,1048,1048,1042,1042,1036,1036,1035,1035,1033,1033,1028,1028,1020,1020,1019,1019,1014,1014,1009,1009,1005,1005,1001,1001,999,999,998,998,989,989,988,988,983,983,982,982,980,980,978,978,972,972,971,971,969,969,967,967,963,963,962,962,946,946,944,944,941,941,940,940,937,937,934,934,933,933,927,927,917,917,916,916,907,907,905,905,903,903,900,900,899,899,898,898,891,891,886,886,882,882,877,877,869,869,868,868,867,867,865,865,860,860,850,850,848,848,847,847,846,846,841,841,840,840,839,839,831,831,830,830,824,824,822,822,821,821,820,820,817,817,809,809,805,805,800,800,797,797,796,796,789,789,788,788,787,787,785,785,781,781,780,780,778,778,773,773,772,772,770,770,765,765,761,761,759,759,756,756,753,753,752,752,746,746,744,744,741,741,737,737,735,735,734,734,733,733,727,727,725,725,722,722,720,720,719,719,716,716,709,709,705,705,699,699,698,698,695,695,694,694,693,693,687,687,683,683,681,681,678,678,666,666,664,664,662,662,661,661,658,658,655,655,653,653,646,646,644,644,643,643,641,641,640,640,632,632,631,631,630,630,629,629,626,626,619,619,614,614,612,612,611,611,610,610,607,607,605,605,601,601,599,599,594,594,590,590,585,585,578,578,577,577,574,574,573,573,570,570,569,569,563,563,562,562,559,559,557,557,552,552,547,547,545,545,543,543,541,541,538,538,537,537,533,533,529,529,522,522,516,516,514,514,513,513,511,511,509,509,505,505,499,499,498,498,496,496,494,494,490,490,489,489,487,487,483,483,481,481,480,480,476,476,473,473,468,468,461,461,456,456,452,452,450,450,449,449,448,448,439,439,436,436,435,435,433,433,432,432,429,429,427,427,426,426,425,425,422,422,419,419,417,417,415,415,414,414,413,413,408,408,407,407,406,406,405,405,404,404,402,402,401,401,400,400,395,395,390,390,389,389,388,388,387,387,385,385,381,381,380,380,377,377,371,371,367,367,366,366,365,365,363,363,362,362,361,361,355,355,352,352,348,348,346,346,343,343,342,342,340,340,338,338,329,329,326,326,322,322,317,317,316,316,313,313,312,312,311,311,310,310,308,308,306,306,304,304,301,301,300,300,295,295,293,293,289,289,284,284,283,283,281,281,278,278,277,277,276,276,275,275,274,274,273,273,270,270,269,269,267,267,266,266,263,263,260,260,259,259,258,258,257,257,255,255,253,253,250,250,249,249,245,245,240,240,239,239,238,238,237,237,236,236,233,233,232,232,231,231,230,230,229,229,226,226,224,224,222,222,221,221,219,219,218,218,215,215,214,214,213,213,212,212,211,211,210,210,207,207,205,205,204,204,202,202,201,201,200,200,199,199,198,198,197,197,196,196,195,195,194,194,193,193,192,192,191,191,188,188,187,187,185,185,184,184,183,183,182,182,181,181,180,180,179,179,178,178,174,174,173,173,172,172,171,171,170,170,168,168,167,167,166,166,165,165,164,164,163,163,162,162,161,161,160,160,159,159,158,158,157,157,156,156,155,155,154,154,153,153,152,152,151,151,150,150,149,149,148,148,147,147,146,146,145,145,143,143,142,142,141,141,140,140,139,139,138,138,137,137,136,136,135,135,134,134,133,133,132,132,131,131,130,130,129,129,128,128,127,127,126,126,125,125,124,124,123,123,122,122,121,121,120,120,119,119,118,118,117,117,116,116,115,115,114,114,113,113,112,112,111,111,110,110,109,109,108,108,107,107,106,106,105,105,104,104,103,103,102,102,101,101,100,100,99,99,98,98,97,97,96,96,94,94,93,93,92,92,90,90,89,89,87,87,86,86,85,85,83,83,82,82,81,81,80,80,78,78,77,77,75,75,72,72,69,69,68,68,0.0]],"coord\_x":[[0.0,1e-10,0.48902364929040526,0.48902364939040527,0.9400263043796647,0.9400263044796647,1.3827055229046763,1.3827055230046763,1.816423271400585,1.816423271500585,2.2380473855853737,2.2380473856853738,2.645054733120752,2.645054733220752,3.0154911625114735,3.0154911626114735,3.3662065575339426,3.3662065576339426,3.715413873457663,3.715413873557663,4.049511396872766,4.049511396972766,4.369717191666703,4.369717191766703,4.678235373445337,4.678235373545337,4.97895214604006,4.97895214614006,5.2622390044357825,5.2622390045357825,5.5436697654791995,5.5436697655791995,5.81512400325397,5.81512400335397,6.085621190831459,6.085621190931459,6.350753096999938,6.350753097099938,6.615043959055653,6.615043959155653,6.876405667477259,6.876405667577259,7.135621263335262,7.135621263435262,7.385904390685291,7.385904390785291,7.635549484570466,7.635549484670466,7.883744502399151,7.883744502499151,8.129619398537454,8.129619398637454,8.371956109097923,8.371956109197923,8.611102652334115,8.611102652434115,8.844941917203558,8.844941917303558,9.075330001058555,9.075330001158555,9.304674030152881,9.304674030252881,9.53367004099365,9.53367004109365,9.761941013806174,9.761941013906174,9.984933709773077,9.984933709873077,10.206273319035583,10.206273319135583,10.427496922213571,10.427496922313571,10.643964275926272,10.643964276026272,10.860257620512195,10.860257620612195,11.07512989056276,11.07512989066276,11.289741146923156,11.289741147023156,11.502554308973506,11.502554309073506,11.713134353896862,11.713134353996862,11.922902356228585,11.922902356328585,12.13211932965884,12.13211932975884,12.339712217905829,12.339712218005829,12.536632546377058,12.536632546477058,12.732624826172133,12.732624826272133,12.925832959938749,12.925832960038749,13.118693075451807,13.118693075551807,13.311234174232439,13.311234174332439,13.50360126388629,13.50360126398629,13.694779291173822,13.694779291273822,13.884652250010513,13.884652250110513,14.073220140396364,14.073220140496364,14.261411011007528,14.261411011107528,14.44832581468898,14.44832581478898,14.632398469299714,14.632398469399714,14.816181108699151,14.816181108799151,14.998890691816786,14.998890691916786,15.180498217131488,15.180498217231488,15.54275621756361,15.54275621766361,15.72204362118793,15.72204362128793,15.897676833149898,15.897676833249898,16.073078032942828,16.073078033042826,16.24682614603136,16.246826146131358,16.420284243908593,16.42028424400859,16.59333632049001,16.59333632059001,16.766301392508037,16.766301392608035,16.93859942954008,16.938599429640078,17.11005642245936,17.110056422559357,17.279454307378423,17.27945430747842,17.44856217708619,17.448562177186187,17.616277973779724,17.616277973879722,17.783326735487275,17.783326735587274,17.95020148806805,17.950201488168048,18.116699220874136,18.116699220974134,18.28310994911683,18.28310994921683,18.44940467127501,18.449404671375007,18.614249317376697,18.614249317476695,18.77694785091478,18.77694785101478,18.939443373804956,18.939443373904954,19.09961877500475,19.099618775104748,19.259359153387596,19.259359153487594,19.418490499826717,19.418490499926715,19.57701281432211,19.57701281442211,19.73527411512734,19.735274115227337,19.893274402242398,19.893274402342396,20.05104267718842,20.051042677288418,20.20831792627523,20.20831792637523,20.364665126685896,20.364665126785894,20.520258287547183,20.52025828764718,20.674894398211187,20.674894398311185,20.829182490621633,20.82918249072163,20.982600537398188,20.982600537498186,21.13567056592118,21.13567056602118,21.288015556415935,21.288015556515933,21.43847544803725,21.438475448137247,21.588819333574047,21.588819333674046,21.737916153702262,21.73791615380226,21.88675196014031,21.886751960240307,22.03515274376141,22.03515274386141,22.183524525861383,22.18352452596138,22.331722298834574,22.331722298934572,22.47948504899082,22.47948504909082,22.62695778393577,22.626957784035767,22.773415465641172,22.77341546574117,22.91975714126206,22.91975714136206,23.066011812319555,23.066011812419553,23.212208480334795,23.212208480434793,23.357970125533082,23.35797012563308,23.503615764646856,23.503615764746854,23.648333355084475,23.648333355184473,23.793021944000962,23.79302194410096,23.937275510100505,23.937275510200504,24.079498969720962,24.07949896982096,24.22134540956673,24.22134540966673,24.361422756623586,24.361422756723584,24.50144210063818,24.501442100738178,24.641316437047124,24.641316437147122,24.781103768892677,24.781103768992676,24.920601085526936,24.920601085626934,25.060069400640064,25.060069400740062,25.198870680767207,25.198870680867206,25.337149933514013,25.33714993361401,25.47513917104952,25.475139171149518,25.613012402500512,25.61301240260051,25.750595618740203,25.7505956188402,25.88722178478261,25.88722178488261,26.022890900627736,26.022890900727734,26.158095992134786,26.158095992234784,26.292373034965685,26.292373035065683,26.42546101543026,26.425461015530257,26.558461991331445,26.558461991431443,26.69050591703535,26.690505917135347,26.821766801668748,26.821766801768746,26.95285367717537,26.952853677275368,27.08373754203408,27.083737542134077,27.214563403850534,27.21456340395053,27.345244258061335,27.345244258161333,27.475403084891802,27.4754030849918,27.60553291020114,27.60553291030114,27.73525671421466,27.73525671431466,27.864632499974622,27.86463250007462,27.99374727204442,27.993747272144418,28.122224010649358,28.122224010749356,28.250004712747184,28.250004712847183,28.37772741180275,28.377727411902747,28.50510209260476,28.505102092704757,28.632331765801116,28.632331765901114,28.758575387279063,28.75857538737906,28.884499992024583,28.88449999212458,29.010250587643323,29.01025058774332,29.135740169571893,29.13574016967189,29.260939736289167,29.260939736389165,29.384776231513342,29.38477623161334,29.507626675019104,29.507626675119102,29.629114047031766,29.629114047131765,29.75036940687539,29.750369406975388,29.871566763676753,29.87156676377675,29.992126087013265,29.992126087113263,30.112598405786382,30.11259840588638,30.232055671319962,30.23205567141996,30.351193920121112,30.35119392022111,30.47021616283774,30.47021616293774,30.588948390343074,30.588948390443072,30.70727459655259,30.70727459665259,30.825571801240976,30.825571801340974,30.943723998323712,30.94372399842371,31.06167318475854,31.061673184858538,31.178926334686253,31.17892633478625,31.295077426811037,31.295077426911035,31.41093850372452,31.410938503824518,31.64144259366404,31.641442593764037,31.75512855649279,31.755128556592787,31.86866951171589,31.868669511815888,31.981949453248824,31.981949453348822,32.09476537044368,32.09476537054368,32.207552286117405,32.20755228621741,32.32013619114323,32.32013619124323,32.43161803836611,32.43161803846611,32.54298387950448,32.542983879604485,32.65394369934703,32.653943699447034,32.76423648420359,32.764236484303595,32.8736882249474,32.8736882250474,32.98311096417007,32.983110964270075,33.201753431967504,33.201753432067505,33.31088615597888,33.31088615607888,33.419960876948,33.419960877048,33.528687579663554,33.528687579763556,33.637182270210076,33.63718227031008,33.74556095467207,33.745560954772074,33.85373662848617,33.85373662858617,33.96170929165235,33.96170929175235,34.06852189397334,34.06852189407334,34.17524749173094,34.175247491830945,34.28081302864335,34.28081302874335,34.386204556428986,34.38620455652899,34.49078404162298,34.49078404172298,34.59510251312681,34.59510251322681,34.69895696029257,34.69895696039257,34.80255039376815,34.80255039386815,34.90611482572261,34.90611482582261,35.00915723029673,35.00915723039673,35.11199662422294,35.11199662432294,35.214487999895596,35.2144879999956,35.31677636492034,35.31677636502034,35.41874571321266,35.41874571331266,35.51970000826543,35.51970000836543,35.62036428810691,35.620364288206915,35.720767554258224,35.720767554358225,35.820909806719364,35.820909806819365,35.920095008983225,35.92009500908323,36.01907720059917,36.01907720069917,36.117508363313654,36.117508363413656,36.2157075138591,36.2157075139591,36.313007617249525,36.31300761734953,36.41024971759769,36.41024971769769,36.50743381490359,36.507433815003594,36.60424089243481,36.60424089253481,36.700989966923764,36.700989967023766,36.79715901099013,36.79715901109013,36.89303803984519,36.89303803994519,36.98885906565799,36.98885906575799,37.08450608234402,37.08450608244402,37.179950088382135,37.17995008848214,37.27527808833573,37.27527808843573,37.37022906851465,37.37022906861465,37.46512204565129,37.46512204575129,37.55940599084422,37.55940599094422,37.653254913220195,37.6532549133202,37.746291793004545,37.74629179310455,37.839270669746625,37.83927066984663,37.932220544967585,37.93222054506759,38.02496740954063,38.02496740964063,38.11756926650803,38.11756926660803,38.20988110826413,38.20988110836413,38.3021639484991,38.3021639485991,38.39438878569181,38.39438878579181,38.48658462136339,38.48658462146339,38.57860644790819,38.578606448008195,38.670425263805086,38.67042526390509,38.76212807361746,38.76212807371746,38.8533088560495,38.8533088561495,38.94434463087589,38.94434463097589,39.03535140418115,39.03535140428115,39.12627117292302,39.12627117302302,39.21713293862263,39.217132938722635,39.30700865260383,39.30700865270383,39.39644934376808,39.396449343868085,39.48542601059425,39.485426010694255,39.573329621138626,39.57332962123863,39.660421189091366,39.66042118919137,39.74713573726942,39.74713573736942,39.833763280884085,39.83376328098409,39.920042806245185,39.92004280634519,40.005829305747085,40.00582930584709,40.09112277938978,40.09112277948978,40.17632924846908,40.17632924856908,40.261332706900475,40.26133270700048,40.431252619199874,40.431252619299876,40.51558904264529,40.51558904274529,40.59972245544279,40.59972245554279,40.76796027951666,40.76796027961666,40.8520356892719,40.8520356893719,40.936024094463754,40.936024094563756,41.01992549509222,41.01992549519222,41.10327586681922,41.10327586691922,41.186568235503955,41.18656823560396,41.269483584414004,41.269483584514006,41.35213791963388,41.352137919733885,41.43447323812134,41.434473238221344,41.516605545960886,41.51660554606089,41.68081215859772,41.68081215869772,41.76282846035274,41.762828460452745,41.84464175145986,41.84464175155986,41.925991018228906,41.92599101832891,42.00731128347682,42.00731128357682,42.0886025472036,42.0886025473036,42.169777804845864,42.169777804945866,42.25086605792474,42.25086605802474,42.33160629275005,42.331606292850054,42.41211451540633,42.41211451550633,42.49218771524566,42.492187715345665,42.57205790443709,42.57205790453709,42.65137706472704,42.651377064827045,42.730435211326835,42.73043521142684,42.80873931837725,42.80873931847725,42.88672440869524,42.88672440879524,42.96456449140757,42.96456449150757,43.042027554345225,43.04202755444523,43.11928760663497,43.11928760673497,43.2734596929609,43.2734596930609,43.350168716349174,43.350168716449176,43.50352876008347,43.50352876018347,43.57965775304915,43.57965775314915,43.65561273688805,43.655612736988054,43.73145171464243,43.731451714742434,43.80720368783343,43.80720368793343,43.88234662908069,43.882346629180695,43.95737356424345,43.95737356434345,44.03234249636393,44.03234249646393,44.10693440870973,44.106934408809735,44.18146831801328,44.18146831811328,44.2558862212323,44.255886221332304,44.33013011532455,44.33013011542455,44.403880983557585,44.40388098365759,44.47713882593142,44.47713882603142,44.550309663741864,44.550309663841865,44.623248489383265,44.62324848948327,44.695433275475295,44.6954332755753,44.76753105700394,44.767531057103945,44.839512832448065,44.83951283254807,44.911349600286535,44.91134960038654,44.9829833574771,44.982983357577105,45.054327099456366,45.05432709955637,45.12535182470321,45.12535182480321,45.19626054386553,45.19626054396553,45.26714026150672,45.26714026160672,45.337961976105646,45.33796197620565,45.40852267701441,45.40852267711441,45.478996373359784,45.478996373459786,45.54929606057838,45.54929606067838,45.619566746275844,45.619566746375845,45.68954741676201,45.68954741686201,45.7593540781214,45.7593540782214,45.82898673035401,45.82898673045401,45.89809735520628,45.898097355306284,45.966917964847255,45.96691796494726,46.035100541023375,46.03510054112338,46.10284809438255,46.10284809448255,46.1704796416572,46.170479641757204,46.23793717980508,46.23793717990508,46.30522070882618,46.30522070892618,46.372127218072585,46.37212721817259,46.639550244410316,46.63955024451032,46.70599272931865,46.705992729418654,46.77231920814247,46.77231920824247,46.83861668544515,46.838616685545155,46.904827158184446,46.90482715828445,46.970863621796966,46.97086362189697,47.03684208236722,47.036842082467224,47.10270453685296,47.10270453695296,47.16804496395836,47.16804496405836,47.23324038345811,47.23324038355811,47.298203790788826,47.29820379088883,47.36310919507728,47.36310919517728,47.427869591760086,47.42786959186009,47.492513982358375,47.49251398245838,47.556955362308756,47.55695536240876,47.621367740738,47.621367740838004,47.68569311460386,47.68569311470386,47.749786476300685,47.749786476400686,47.81356082126508,47.81356082136508,47.876465120595576,47.87646512069558,47.93902140167252,47.93902140177252,48.00146167666494,48.00146167676494,48.0638439486151,48.0638439487151,48.12593620535397,48.125936205453975,48.187970459050575,48.18797045915058,48.24994670970492,48.249946709804924,48.37372520188684,48.373725201986844,48.43541143732989,48.43541143742989,48.49674965451938,48.49674965461938,48.55788486106096,48.557884861160964,48.68006826958074,48.68006826968074,48.740826456347634,48.740826456447635,48.800975611170806,48.80097561127081,48.8609507568672,48.8609507569672,48.92086789952133,48.920867899621335,48.98069803761207,48.980698037712074,49.04035416657604,49.04035416667604,49.099894289455484,49.099894289555486,49.15926040320815,49.15926040330815,49.218017485017086,49.21801748511709,49.27668756226264,49.27668756236264,49.33532863798706,49.33532863808706,49.452581787914774,49.452581788014776,49.56977693480023,49.56977693490023,49.62818599835561,49.62818599845561,49.68642105278422,49.68642105288422,49.802572144909,49.802572145009,49.91837521878023,49.91837521888023,49.97611724734962,49.97611724744962,50.03377227135563,50.03377227145563,50.091369292319385,50.09136929241939,50.14882130567748,50.14882130577748,50.20618631447219,50.20618631457219,50.320800325977096,50.3208003260771,50.37746929826469,50.377469298364694,50.43408026751003,50.434080267610035,50.49060423219198,50.49060423229198,50.60344915090796,50.603449151007965,50.6592770790828,50.6592770791828,50.71484399356746,50.714843993667465,50.77032390348874,50.77032390358874,50.825745810367756,50.82574581046776,50.88108071268338,50.88108071278338,50.93638661347788,50.93638661357788,51.04696941354574,51.04696941364574,51.10181129000216,51.10181129010216,51.15662416493745,51.15662416503745,51.211408038351614,51.211408038451616,51.266162910244645,51.26616291034465,51.320772774532024,51.320772774632026,51.37532463577715,51.37532463587715,51.42976049093775,51.429760491037754,51.59269103664487,51.592691036744874,51.646604864425136,51.64660486452514,51.70037368459976,51.70037368469976,51.75405550021099,51.75405550031099,51.86133212687005,51.861332126970055,51.91481093183337,51.914810931933374,51.968231733754436,51.96823173385444,52.021623534154365,52.02162353425437,52.074812323906386,52.07481232400639,52.127885107573896,52.1278851076739,52.18084188515687,52.180841885256875,52.23330563688065,52.23330563698065,52.285682384041046,52.28568238414105,52.33779811751126,52.337798117611264,52.38950782968567,52.38950782978567,52.44118854033894,52.441188540438944,52.49272424338657,52.49272424348657,52.54411493882854,52.544114938928544,52.64672232058572,52.64672232068572,52.74877867344142,52.74877867354142,52.79944433085515,52.799444330955154,52.849993982184365,52.84999398228437,52.90042762742906,52.90042762752906,52.95080326963149,52.950803269731495,53.001091907270535,53.00109190737054,53.051293540346194,53.051293540446196,53.101408168858455,53.10140816895846,53.15149379584959,53.15149379594959,53.20140541371395,53.20140541381395,53.251288030057175,53.25128803015718,53.30108364183702,53.30108364193702,53.35070524449007,53.350705244590074,53.40026884410087,53.40026884420087,53.44980344219054,53.449803442290545,53.49930903875908,53.49930903885908,53.548785633806496,53.5487856339065,53.59823322733278,53.59823322743278,53.647361804126625,53.64736180422663,53.69611336114579,53.69611336124579,53.74483591664383,53.74483591674383,53.84219402307651,53.84219402317651,53.89080057249003,53.89080057259003,53.939291115819024,53.939291115919026,53.987607650021246,53.98760765012125,54.03580817813894,54.035808178238945,54.083863698650994,54.083863698750996,54.13189021764192,54.13189021774192,54.17982973206945,54.17982973216945,54.22765324041246,54.22765324051246,54.27541874571321,54.27541874581321,54.32303924340832,54.32303924350832,54.37060173806116,54.37060173816116,54.41810622967174,54.418106229771745,54.465581719761204,54.465581719861206,54.56044569537672,54.56044569547672,54.60771817481827,54.60771817491827,54.654874648175294,54.654874648275296,54.702002120011194,54.702002120111196,54.749013585762576,54.74901358586258,54.795880043908305,54.79588004400831,54.842514489885,54.842514489985,54.889032929777166,54.88903292987717,54.98204080804038,54.98204080814038,55.074961681740206,55.07496168184021,55.121045098815436,55.12104509891544,55.16704151132727,55.16704151142727,55.212747908627804,55.212747908727806,55.258396302886084,55.258396302986085,55.30381268497533,55.30381268507533,55.34914206250118,55.34914206260118,55.39438443546364,55.394384435563644,55.484637169219525,55.48463716931953,55.52953152392843,55.529531524028435,55.57439687711621,55.57439687721621,55.618856209008165,55.618856209108166,55.66322853633673,55.66322853643673,55.70684682411593,55.70684682421593,55.7503491058106,55.7503491059106,55.79373538142075,55.793735381520754,55.83709265550978,55.83709265560978,55.88030492199316,55.88030492209316,55.9234881869554,55.923488187055405,55.966584447354265,55.96658444745427,56.00959370318973,56.00959370328973,56.05242894989842,56.05242894999842,56.094945179874685,56.09494517997469,56.137432408329815,56.13743240842982,56.17962962157365,56.179629621673655,56.26379203589228,56.26379203599228,56.30572823544595,56.30572823554595,56.347490425872834,56.347490425972836,56.389223614778594,56.389223614878595,56.430840797599835,56.43084079769984,56.47213896368864,56.472138963788645,56.55459028826062,56.55459028836062,56.595627440659264,56.595627440759266,56.636606590015646,56.63660659011565,56.67744073176638,56.67744073186638,56.718216870474855,56.71821687057486,56.758906004619945,56.75890600471995,56.84025527138898,56.840255271488985,56.88050938271712,56.880509382817124,56.920705491003005,56.92070549110301,56.96069858864097,56.960698588740975,57.00048867563104,57.00048867573104,57.04022075957884,57.04022075967884,57.079720831357605,57.07972083145761,57.11919190161524,57.11919190171524,57.158604968830616,57.15860496893062,57.19784402691921,57.197844027019215,57.23693807740216,57.23693807750216,57.27591612180059,57.27591612190059,57.31486516467789,57.31486516477789,57.35361119690728,57.35361119700728,57.39229922609441,57.39229922619441,57.43078424463363,57.43078424473363,57.546210298730166,57.54621029883017,57.58452130814261,57.584521308242614,57.622774314512796,57.6227743146128,57.66076630719281,57.66076630729281,57.69849728618266,57.69849728628266,57.73617026213025,57.73617026223025,57.77375623351445,57.77375623361445,57.811226198814126,57.81122619891413,57.848667162592676,57.84866716269268,57.92349108710752,57.92349108720752,57.96084504632268,57.96084504642268,57.99814100249558,57.99814100259558,58.035146943457185,58.03514694355719,58.072036878334266,58.07203687843427,58.108752804084574,58.108752804184576,58.14543972831375,58.14543972841375,58.182068649500664,58.182068649600666,58.21849456003967,58.218494560139675,58.25463045536738,58.254630455467385,58.29062134308945,58.29062134318945,58.32658322929038,58.32658322939038,58.3623421048434,58.3623421049434,58.398071978875294,58.398071978975295,58.43368584682266,58.433685846922664,58.46918370868552,58.46918370878552,58.50465256902724,58.504652569127245,58.54000542328445,58.54000542338445,58.646034984534936,58.64603498463494,58.681329835749885,58.68132983584989,58.716334671753536,58.71633467185354,58.75122350167266,58.751223501772664,58.85562897773988,58.855628977839885,58.89022779244771,58.89022779254771,58.924652598028764,58.924652598128766,58.95899039904643,58.95899039914643,58.993270197021836,58.99327019712184,59.06171378688812,59.06171378698812,59.09561656508884,59.09561656518884,59.16339311996914,59.163393120069145,59.19717989208534,59.19717989218534,59.23093766268041,59.23093766278041,59.2645214241487,59.2645214242487,59.2980181810536,59.2980181811536,59.33145693491624,59.33145693501624,59.36408364618724,59.36408364628724,59.39659435137373,59.39659435147373,59.42901805199683,59.42901805209683,59.4614127510988,59.4614127511988,59.493778448679635,59.493778448779636,59.52605714169708,59.52605714179708,59.55816182558775,59.558161825687755,59.62208117815779,59.622081178257794,59.65386684531604,59.65386684541604,59.68559450943202,59.685594509532024,59.71729317202687,59.71729317212687,59.7489628331006,59.7489628332006,59.78054548961093,59.78054548971093,59.812070143079005,59.81207014317901,59.84344978894143,59.84344978904143,59.874568421113686,59.87456842121369,59.905252030469,59.905252030569,59.93587763678205,59.935877636882054,59.96627123092606,59.96627123102606,60.02688441008731,60.026884410187314,60.05692998597777,60.05692998607777,60.08694656034709,60.08694656044709,60.14692170604348,60.14692170614348,60.1767352697649,60.1767352698649,60.23613038503869,60.236130385138694,60.26568293506995,60.26568293516995,60.29509047749554,60.295090477595544,60.3243530123155,60.3243530124155,60.35349954105093,60.35349954115093,60.38253006370184,60.382530063801845,60.411502583310494,60.411502583410496,60.44044610139802,60.44044610149802,60.469128605795376,60.46912860589538,60.4977821086716,60.4977821087716,60.52629060394218,60.52629060404218,60.55477009769162,60.554770097791625,60.58319158839881,60.58319158849881,60.61155507606374,60.61155507616374,60.63974455460189,60.63974455470189,60.66790503161891,60.66790503171891,60.69600750559367,60.69600750569367,60.72405197652617,60.72405197662617,60.75198044137415,60.75198044147415,60.779879904701005,60.77987990480101,60.80731534368978,60.80731534378978,60.88956365761384,60.88956365771384,60.94423152494348,60.944231525043485,60.97149295480548,60.97149295490548,61.02592880996608,61.025928810066084,61.0530162307013,61.053016230801305,61.08007464991539,61.08007465001539,61.106959060002694,61.106959060102696,61.13355345487871,61.13355345497871,61.1867132431096,61.1867132432096,61.213017622774316,61.21301762287432,61.265568379061484,61.265568379161486,61.291756752641675,61.29175675274168,61.31785812165848,61.31785812175848,61.343930489154154,61.343930489254156,61.3699738551287,61.3699738552287,61.39581421045534,61.39581421055534,61.421509558176325,61.42150955827633,61.4470888998128,61.4470888999128,61.47252323384362,61.47252323394362,61.497725555705394,61.497725555805395,61.52289887604605,61.52289887614605,61.57321651520622,61.57321651530622,61.598302830983485,61.59830283108349,61.6232441391551,61.6232441392551,61.64789543211541,61.647895432215414,61.697140014993785,61.69714001509379,61.721704303390716,61.72170430349072,61.74623959026651,61.746239590366514,61.770629869536656,61.77062986963666,61.79499114728568,61.79499114738568,61.84368470126258,61.843684701362584,61.867784965321434,61.867784965421436,61.89185622785915,61.89185622795915,61.93982474380781,61.93982474390781,61.987561247587436,61.98756124768744,62.01137149643499,62.01137149653499,62.03515274376141,62.03515274386141,62.05884698652444,62.058846986624445,62.082309217118436,62.08230921721844,62.10565544162791,62.10565544172791,62.12885665853174,62.12885665863174,62.15197087087218,62.15197087097218,62.17505608169149,62.17505608179149,62.19793828186288,62.197938281962884,62.22079148051315,62.22079148061315,62.28932207494283,62.28932207504283,62.31208826902971,62.31208826912971,62.334738457032074,62.334738457132076,62.380009831515665,62.380009831615666,62.40257301495463,62.402573015054635,62.424991190787956,62.42499119088796,62.44738036510015,62.447380365200154,62.469711536370085,62.469711536470086,62.491897700034365,62.491897700134366,62.51396785761413,62.513967857714135,62.53598001215164,62.53598001225164,62.557905162125756,62.55790516222576,62.6016684575106,62.6016684576106,62.6234776014002,62.6234776015002,62.645112736163014,62.645112736263016,62.66668986788357,62.66668986798357,62.68817999504074,62.68817999514074,62.70955411611339,62.70955411621339,62.73087023414378,62.73087023424378,62.75215735065304,62.752157350753045,62.77341546564117,62.773415465741174,62.79449957150253,62.79449957160253,62.81552567432162,62.81552567442162,62.83646477257732,62.836464772677324,62.85734586779076,62.857345867890764,62.87819796148308,62.87819796158308,62.898963050612004,62.898963050712005,62.919525129093024,62.919525129193026,62.93997120148952,62.93997120158952,62.960243264759235,62.96024326485924,62.98048632650782,62.980486326607824,63.00064238369303,63.00064238379303,63.04092549654229,63.040925496642295,63.061023550685235,63.06102355078524,63.080947595701396,63.0809475958014,63.1206796796492,63.1206796797492,63.14042971553858,63.14042971563858,63.19959281864334,63.19959281874334,63.218907831715775,63.21890783181578,63.23816484174595,63.23816484184595,63.25736384873387,63.25736384883387,63.27653385420065,63.27653385430065,63.29561685510405,63.29561685520405,63.31461285144406,63.31461285154406,63.33355084474181,63.33355084484181,63.35228582739165,63.35228582749165,63.37096280699923,63.37096280709923,63.42696474430084,63.42696474440084,63.44555471934503,63.445554719445035,63.482705667912285,63.48270566801229,63.50103462926631,63.50103462936631,63.519334589099195,63.5193345891992,63.53760554741096,63.537605547510964,63.555847504201594,63.555847504301596,63.574002456428836,63.57400245652884,63.59195439800818,63.59195439810818,63.609761331981865,63.60976133208187,63.62751026291329,63.62751026301329,63.64523019232359,63.64523019242359,63.66292112021276,63.66292112031276,63.68052504353853,63.68052504363853,63.698070963822055,63.698070963922056,63.715500878021054,63.715500878121055,63.73287278917779,63.73287278927779,63.75009969272888,63.75009969282888,63.767210590195454,63.767210590295456,63.78417648005638,63.78417648015638,63.80093935926939,63.800939359369394,63.81767323696128,63.81767323706128,63.85105398778166,63.85105398788166,63.86767185938903,63.86767185948903,63.884202726433,63.884202726533005,63.90070459195585,63.90070459205585,63.91703244835192,63.91703244845192,63.93333130322686,63.93333130332686,63.9495431535384,63.9495431536384,63.99812070143079,63.998120701530794,64.01412954109443,64.01412954119444,64.02999337315242,64.02999337325242,64.04579920216815,64.04579920226816,64.06154702814163,64.06154702824163,64.07723685107284,64.07723685117284,64.09283966944066,64.09283966954067,64.10841348628736,64.10841348638736,64.12387129704953,64.12387129714953,64.15467091248937,64.15467091258937,64.18515151119676,64.18515151129677,64.20011629609974,64.20011629619974,64.22998786286341,64.22998786296341,64.24486564320298,64.24486564330299,64.2596854205003,64.2596854206003,64.27444719475537,64.27444719485537,64.2890929629259,64.28909296302591,64.30356472196966,64.30356472206967,64.3180074794923,64.3180074795923,64.33239223397267,64.33239223407267,64.34671898541079,64.34671898551079,64.37525648220249,64.37525648230249,64.40364897138855,64.40364897148855,64.41777271217876,64.41777271227876,64.43178044688445,64.43178044698445,64.44573017854786,64.44573017864786,64.45965090869016,64.45965090879017,64.47345563274794,64.47345563284794,64.48717335224232,64.48717335234232,64.50074606413106,64.50074606423107,64.5141157653719,64.5141157654719,64.52734045900708,64.52734045910708,64.5668985338281,64.5668985339281,64.5799492183365,64.5799492184365,64.59297090132378,64.59297090142378,64.60596358278991,64.60596358288991,64.61869525056589,64.6186952506659,64.64407158155446,64.64407158165446,64.65668724324591,64.65668724334591,64.6692449018951,64.6692449019951,64.70688887632156,64.70688887642156,64.71933052888625,64.71933052898625,64.73171417840865,64.73171417850865,64.74406882640994,64.74406882650995,64.75639447289011,64.75639447299011,64.76863311480687,64.76863311490688,64.78078475216024,64.78078475226025,64.79287838647137,64.79287838657137,64.80491401774023,64.80491401784023,64.81692064748796,64.81692064758796,64.82889827571456,64.82889827581457,64.84073089633551,64.84073089643552,64.86436713605629,64.86436713615629,64.87614175363498,64.87614175373498,64.88788736969254,64.88788736979254,64.89960398422897,64.89960398432898,64.91126259572314,64.91126259582315,64.92289220569619,64.92289220579619,64.9344928141481,64.9344928142481,64.94594841499436,64.94594841509436,64.96871460908125,64.96871460918125,64.97999620080073,64.97999620090073,64.9912487909991,64.9912487910991,65.00247237967632,65.00247237977632,65.0248615539885,65.0248615540885,65.03591113353896,65.03591113363896,65.04693171156828,65.04693171166828,65.06888586306351,65.06888586316352,65.0905790008686,65.0905790009686,65.11198212346237,65.11198212356237,65.12259668019588,65.12259668029589,65.13318223540824,65.13318223550824,65.14370978757836,65.14370978767836,65.15420833822733,65.15420833832734,65.1646778873552,65.1646778874552,65.17497342735626,65.17497342745627,65.18518196279395,65.18518196289395,65.1952744921471,65.19527449224711,65.20530901845802,65.20530901855803,65.21525654020553,65.21525654030553,65.22517506043192,65.22517506053192,65.25487261806882,65.25487261816882,65.26467513221068,65.26467513231069,65.27421663266239,65.27421663276239,65.2932126290024,65.2932126291024,65.30255111880618,65.30255111890618,65.31174460100432,65.31174460110432,65.33929604607762,65.33929604617762,65.34837352219124,65.34837352229124,65.35742199678373,65.35742199688373,65.36644146985509,65.36644146995509,65.37543194140532,65.37543194150533,65.39335488146354,65.39335488156354,65.40222934692925,65.40222934702925,65.4110458093527,65.4110458094527,65.41977526721277,65.41977526731277,65.4284757235517,65.4284757236517,65.43703117228499,65.43703117238499,65.44552861797601,65.44552861807601,65.46240750327355,65.46240750337356,65.48740681448741,65.48740681458742,65.50385067696801,65.50385067706802,65.51200010440547,65.51200010450547,65.52821195471702,65.52821195481702,65.53624537606997,65.53624537616997,65.54424979590179,65.5442497960018,65.5602296340443,65.5602296341443,65.59210230576593,65.59210230586594,65.60001972103437,65.60001972113437,65.60785013173941,65.60785013183941,65.61565154092332,65.61565154102333,65.62339494706498,65.62339494716498,65.6311093516855,65.6311093517855,65.63873675174263,65.63873675184263,65.64627714723638,65.64627714733638,65.65378854120898,65.65378854130898,65.66127093366048,65.66127093376048,65.6836891094938,65.6836891095938,65.6910844973819,65.6910844974819,65.70581727011582,65.70581727021582,65.7204050352441,65.7204050353441,65.73487679428786,65.73487679438786,65.74920354572598,65.74920354582598,65.75616391079713,65.75616391089713,65.76309527434714,65.76309527444714,65.76999763637603,65.76999763647603,65.77687099688379,65.77687099698379,65.79058871637817,65.79058871647817,65.79734607080141,65.79734607090141,65.81083177812677,65.81083177822677,65.81753112950774,65.81753112960774,65.83760018212955,65.83760018222955,65.85091188032813,65.85091188042813,65.85746622410345,65.85746622420345,65.86396256483653,65.86396256493653,65.87040090252734,65.87040090262734,65.87681023869702,65.87681023879702,65.88316157182444,65.88316157192445,65.88948390343073,65.88948390353073,65.89571923047365,65.89571923057365,65.90192555599542,65.90192555609542,65.90810287999605,65.90810288009605,65.92042852647621,65.92042852657622,65.92654784743459,65.9265478475346,65.93263816687185,65.93263816697186,65.95082212062023,65.95082212072023,65.95676743245183,65.95676743255183,65.96862905459392,65.96862905469392,65.9804036721726,65.9804036722726,65.9862329779197,65.9862329780197,65.99203328214566,65.99203328224566,65.99780458485047,65.99780458495047,66.009318188739,66.009318188839,66.02651609076896,66.02651609086897,66.03791368857297,66.03791368867297,66.0492532833347,66.04925328343471,66.0605348750542,66.0605348751542,66.07175846373143,66.07175846383143,66.08852134294443,66.08852134304443,66.09962892553715,66.09962892563715,66.10508121150954,66.10508121160954,66.12140906790562,66.12140906800562,66.12677434931462,66.12677434941462,66.1321106292025,66.1321106293025,66.1534267472329,66.1534267473329,66.15870502407851,66.15870502417852,66.18506740678549,66.1850674068855,66.19553695591334,66.19553695601334,66.20072822819557,66.20072822829557,66.20589049895668,66.20589049905668,66.22642357591656,66.22642357601656,66.23648710374859,66.23648710384859,66.24147536538291,66.24147536548291,66.27137593366773,66.27137593376773,66.27630619225978,66.27630619235978,66.30089948217784,66.30089948227784,66.31061499175632,66.31061499185633,66.32995900634988,66.32995900644988,66.34918701485893,66.34918701495893,66.36351376629705,66.36351376639705,66.37299726370648,66.37299726380648,66.39660450190613,66.39660450200613,66.41067023965407,66.41067023975407,66.42931821774052,66.42931821784052,66.43856970298093,66.43856970308093,66.45237442703869,66.4523744271387,66.46150990619458,66.46150990629458,66.48424709876033,66.48424709886034,66.4932665718317,66.4932665719317,66.49773280608568,66.49773280618568,66.50217003881853,66.50217003891854,66.5243272009617,66.5243272010617,66.52870643065229,66.5287064307523,66.54619434789355,66.54619434799355,66.55921603088082,66.55921603098082,66.58079316260138,66.58079316270138,66.58505638620746,66.58505638630746,66.60208027911064,66.60208027921064,66.62322238801426,66.62322238811426,66.6273696055358,66.6273696056358,66.63978225657935,66.63978225667935,66.65210790305952,66.65210790315952,66.66434654497628,66.66434654507628,66.67649818232967,66.67649818242967,66.68856281511965,66.68856281521965,66.70054044334626,66.70054044344626,66.72832390058859,66.7283239006886,66.74407172656205,66.74407172666206,66.76361875180353,66.76361875190354,66.77136215794519,66.77136215804519,66.79061916797536,66.79061916807537,66.7982465680325,66.7982465681325,66.82101276211937,66.82101276221937,66.83606455158574,66.83606455168574,66.86970631609628,66.86970631619629,66.89195048280283,66.89195048290283,66.91402064038259,66.91402064048259,66.94687936382263,66.94687936392263,66.96860150314885,66.96860150324885,66.99734201058845,66.99734201068846,67.02585050585903,67.02585050595903,67.05412698896058,67.05412698906058,67.08217145989308,67.08217145999308,67.10302355358539,67.10302355368539,67.1375063622087,67.1375063623087,67.16143261714078,67.16143261724078,67.1919422173693,67.1919422174693,67.23228333326082,67.23228333336083,67.26894125596887,67.26894125606887,67.31189250876209,67.31189250886209,67.35118956989294,67.35118956999294,67.39338678313678,67.39338678323678,67.42233030122429,67.4223303013243,67.46696364224303,67.46696364234303,67.50486863035967,67.50486863045967,67.52989694309467,67.52989694319467,67.56710589470418,67.56710589480419,67.61318931177941,67.61318931187941,67.63752158800729,67.63752158810729,67.67971880125113,67.67971880135113,67.72449714987552,67.72449714997552,67.76588232052771,67.76588232062771,68.0646269896856,68.06462698978561,68.11100042197214,68.11100042207214,68.13681177577764,68.13681177587765,68.15667781775154,68.15667781785154,68.17071455397836,68.17071455407836,68.18179313504993,68.18179313514993,68.19266870547361,68.19266870557361,68.1980339868826,68.19803398698261,68.2086485436161,68.20864854371611,68.21644995280002,68.21644995290002,68.22155422051887,68.22155422061887,68.22404835133602,68.22404835143602,68.22900761144922,68.22900761154922,68.23141473770299,68.231414737803,68.23860711494318,68.23860711504318,68.24809061235261,68.24809061245261,68.250410734043,68.250410734143,68.25267285269112,68.25267285279112,68.25490596981811,68.25490596991811,68.26154731815684,68.26154731825685,68.26363542767818,68.26363542777818,68.26772464215748,68.26772464225748,68.27169785055226,68.27169785065226]],"filenames":["genome.ctg.filtered"]}

{"coord\_y":[[16862,16862,16862,15551,15551,15264,15264,14955,14955,14538,14538,14034,14034,12773,12773,12093,12093,12041,12041,11520,11520,11041,11041,10638,10638,10369,10369,9768,9768,9704,9704,9360,9360,9327,9327,9142,9142,9113,9113,9012,9012,8938,8938,8630,8630,8608,8608,8558,8558,8478,8478,8356,8356,8246,8246,8063,8063,7944,7944,7908,7908,7896,7896,7871,7871,7689,7689,7632,7632,7628,7628,7464,7464,7458,7458,7409,7409,7400,7400,7338,7338,7261,7261,7233,7233,7214,7214,7158,7158,6790,6790,6758,6758,6662,6662,6650,6650,6639,6639,6633,6633,6592,6592,6547,6547,6502,6502,6489,6489,6445,6445,6347,6347,6337,6337,6300,6300,6262,6262,6229,6229,6182,6182,6056,6056,6048,6048,5991,5991,5981,5981,5967,5967,5964,5964,5941,5941,5912,5912,5841,5841,5831,5831,5783,5783,5760,5760,5754,5754,5741,5741,5738,5738,5734,5734,5684,5684,5610,5610,5603,5603,5523,5523,5508,5508,5487,5487,5466,5466,5457,5457,5448,5448,5440,5440,5423,5423,5391,5391,5365,5365,5332,5332,5320,5320,5290,5290,5278,5278,5253,5253,5188,5188,5184,5184,5141,5141,5132,5132,5117,5117,5116,5116,5110,5110,5095,5095,5085,5085,5050,5050,5046,5046,5043,5043,5041,5041,5026,5026,5022,5022,4990,4990,4989,4989,4974,4974,4904,4904,4891,4891,4830,4830,4828,4828,4823,4823,4820,4820,4810,4810,4809,4809,4786,4786,4768,4768,4758,4758,4754,4754,4744,4744,4711,4711,4678,4678,4662,4662,4630,4630,4589,4589,4586,4586,4553,4553,4526,4526,4520,4520,4513,4513,4511,4511,4506,4506,4488,4488,4487,4487,4473,4473,4461,4461,4452,4452,4430,4430,4406,4406,4404,4404,4392,4392,4387,4387,4353,4353,4342,4342,4336,4336,4327,4327,4317,4317,4270,4270,4236,4236,4189,4189,4181,4181,4179,4179,4157,4157,4154,4154,4119,4119,4108,4108,4104,4104,4094,4094,4080,4080,4079,4079,4074,4074,4067,4067,4043,4043,4005,4005,3995,3995,3953,3953,3920,3920,3915,3915,3906,3906,3890,3890,3889,3889,3882,3882,3844,3844,3840,3840,3826,3826,3803,3803,3774,3774,3773,3773,3766,3766,3763,3763,3761,3761,3749,3749,3741,3741,3737,3737,3730,3730,3723,3723,3683,3683,3680,3680,3640,3640,3634,3634,3606,3606,3597,3597,3581,3581,3572,3572,3571,3571,3553,3553,3546,3546,3534,3534,3527,3527,3516,3516,3481,3481,3471,3471,3462,3462,3453,3453,3420,3420,3413,3413,3394,3394,3386,3386,3355,3355,3353,3353,3351,3351,3338,3338,3336,3336,3316,3316,3306,3306,3304,3304,3298,3298,3291,3291,3287,3287,3274,3274,3272,3272,3251,3251,3236,3236,3208,3208,3206,3206,3205,3205,3198,3198,3193,3193,3183,3183,3182,3182,3180,3180,3179,3179,3173,3173,3166,3166,3162,3162,3144,3144,3139,3139,3138,3138,3135,3135,3133,3133,3099,3099,3084,3084,3068,3068,3031,3031,3003,3003,2990,2990,2987,2987,2975,2975,2958,2958,2941,2941,2938,2938,2931,2931,2928,2928,2908,2908,2901,2901,2900,2900,2899,2899,2896,2896,2893,2893,2874,2874,2872,2872,2859,2859,2850,2850,2839,2839,2832,2832,2830,2830,2828,2828,2821,2821,2805,2805,2804,2804,2803,2803,2799,2799,2796,2796,2784,2784,2776,2776,2761,2761,2754,2754,2735,2735,2726,2726,2700,2700,2689,2689,2684,2684,2671,2671,2664,2664,2652,2652,2645,2645,2643,2643,2625,2625,2619,2619,2615,2615,2612,2612,2591,2591,2587,2587,2585,2585,2572,2572,2570,2570,2566,2566,2560,2560,2543,2543,2526,2526,2523,2523,2515,2515,2489,2489,2486,2486,2482,2482,2477,2477,2470,2470,2460,2460,2449,2449,2445,2445,2444,2444,2442,2442,2433,2433,2430,2430,2424,2424,2423,2423,2413,2413,2407,2407,2401,2401,2383,2383,2373,2373,2351,2351,2336,2336,2332,2332,2326,2326,2320,2320,2307,2307,2300,2300,2291,2291,2287,2287,2286,2286,2283,2283,2277,2277,2275,2275,2271,2271,2253,2253,2248,2248,2240,2240,2238,2238,2233,2233,2229,2229,2222,2222,2221,2221,2218,2218,2210,2210,2199,2199,2169,2169,2157,2157,2153,2153,2151,2151,2141,2141,2139,2139,2137,2137,2131,2131,2127,2127,2115,2115,2108,2108,2105,2105,2095,2095,2074,2074,2068,2068,2066,2066,2063,2063,2057,2057,2053,2053,2047,2047,2026,2026,2023,2023,2022,2022,2021,2021,2020,2020,2014,2014,2008,2008,1997,1997,1996,1996,1991,1991,1988,1988,1986,1986,1981,1981,1978,1978,1974,1974,1954,1954,1952,1952,1949,1949,1942,1942,1925,1925,1916,1916,1913,1913,1911,1911,1908,1908,1907,1907,1906,1906,1891,1891,1890,1890,1889,1889,1888,1888,1883,1883,1881,1881,1877,1877,1864,1864,1859,1859,1854,1854,1851,1851,1848,1848,1844,1844,1842,1842,1841,1841,1834,1834,1830,1830,1826,1826,1809,1809,1806,1806,1797,1797,1783,1783,1782,1782,1777,1777,1772,1772,1766,1766,1753,1753,1747,1747,1743,1743,1739,1739,1737,1737,1734,1734,1731,1731,1728,1728,1727,1727,1721,1721,1720,1720,1717,1717,1711,1711,1709,1709,1708,1708,1707,1707,1706,1706,1705,1705,1694,1694,1681,1681,1680,1680,1677,1677,1676,1676,1672,1672,1666,1666,1662,1662,1657,1657,1656,1656,1653,1653,1649,1649,1647,1647,1642,1642,1640,1640,1638,1638,1637,1637,1634,1634,1630,1630,1626,1626,1625,1625,1621,1621,1616,1616,1608,1608,1604,1604,1603,1603,1601,1601,1589,1589,1586,1586,1576,1576,1574,1574,1566,1566,1563,1563,1560,1560,1552,1552,1548,1548,1547,1547,1533,1533,1530,1530,1504,1504,1500,1500,1496,1496,1495,1495,1490,1490,1489,1489,1486,1486,1483,1483,1477,1477,1466,1466,1465,1465,1455,1455,1447,1447,1446,1446,1440,1440,1439,1439,1435,1435,1424,1424,1419,1419,1415,1415,1413,1413,1408,1408,1406,1406,1403,1403,1402,1402,1388,1388,1386,1386,1379,1379,1372,1372,1370,1370,1362,1362,1361,1361,1359,1359,1353,1353,1348,1348,1344,1344,1343,1343,1336,1336,1334,1334,1327,1327,1326,1326,1321,1321,1319,1319,1310,1310,1301,1301,1299,1299,1296,1296,1292,1292,1291,1291,1289,1289,1288,1288,1286,1286,1276,1276,1272,1272,1266,1266,1265,1265,1263,1263,1256,1256,1246,1246,1241,1241,1240,1240,1233,1233,1232,1232,1228,1228,1224,1224,1223,1223,1219,1219,1218,1218,1217,1217,1207,1207,1203,1203,1194,1194,1193,1193,1187,1187,1184,1184,1182,1182,1178,1178,1169,1169,1168,1168,1165,1165,1164,1164,1158,1158,1155,1155,1153,1153,1125,1125,1121,1121,1118,1118,1117,1117,1116,1116,1113,1113,1107,1107,1097,1097,1096,1096,1094,1094,1093,1093,1092,1092,1089,1089,1087,1087,1082,1082,1073,1073,1058,1058,1056,1056,1048,1048,1042,1042,1036,1036,1035,1035,1033,1033,1028,1028,1020,1020,1019,1019,1014,1014,1009,1009,1005,1005,1001,1001,999,999,998,998,989,989,988,988,983,983,982,982,980,980,978,978,972,972,971,971,969,969,967,967,963,963,962,962,946,946,944,944,941,941,940,940,937,937,934,934,933,933,927,927,917,917,916,916,907,907,905,905,903,903,900,900,899,899,898,898,891,891,886,886,882,882,877,877,869,869,868,868,867,867,865,865,860,860,850,850,848,848,847,847,846,846,841,841,840,840,839,839,831,831,830,830,824,824,822,822,821,821,820,820,817,817,809,809,805,805,800,800,797,797,796,796,789,789,788,788,787,787,785,785,781,781,780,780,778,778,773,773,772,772,770,770,765,765,761,761,759,759,756,756,753,753,752,752,746,746,744,744,741,741,737,737,735,735,734,734,733,733,727,727,725,725,722,722,720,720,719,719,716,716,709,709,705,705,699,699,698,698,695,695,694,694,693,693,687,687,683,683,681,681,678,678,666,666,664,664,662,662,661,661,658,658,655,655,653,653,646,646,644,644,643,643,641,641,640,640,632,632,631,631,630,630,629,629,626,626,619,619,614,614,612,612,611,611,610,610,607,607,605,605,601,601,599,599,594,594,590,590,585,585,578,578,577,577,574,574,573,573,570,570,569,569,563,563,562,562,559,559,557,557,552,552,547,547,545,545,543,543,541,541,538,538,537,537,533,533,529,529,522,522,516,516,514,514,513,513,511,511,509,509,505,505,499,499,498,498,496,496,494,494,490,490,489,489,487,487,483,483,481,481,480,480,476,476,473,473,468,468,461,461,456,456,452,452,450,450,449,449,448,448,439,439,436,436,435,435,433,433,432,432,429,429,427,427,426,426,425,425,422,422,419,419,417,417,415,415,414,414,413,413,408,408,407,407,406,406,405,405,404,404,402,402,401,401,400,400,395,395,390,390,389,389,388,388,387,387,385,385,381,381,380,380,377,377,371,371,367,367,366,366,365,365,363,363,362,362,361,361,355,355,352,352,348,348,346,346,343,343,342,342,340,340,338,338,329,329,326,326,322,322,317,317,316,316,313,313,312,312,311,311,310,310,308,308,306,306,304,304,301,301,300,300,295,295,293,293,289,289,284,284,283,283,281,281,278,278,277,277,276,276,275,275,274,274,273,273,270,270,269,269,267,267,266,266,263,263,260,260,259,259,258,258,257,257,255,255,253,253,250,250,249,249,245,245,240,240,239,239,238,238,237,237,236,236,233,233,232,232,231,231,230,230,229,229,226,226,224,224,222,222,221,221,219,219,218,218,215,215,214,214,213,213,212,212,211,211,210,210,207,207,205,205,204,204,202,202,201,201,200,200,199,199,198,198,197,197,196,196,195,195,194,194,193,193,192,192,191,191,188,188,187,187,185,185,184,184,183,183,182,182,181,181,180,180,179,179,178,178,174,174,173,173,172,172,171,171,170,170,168,168,167,167,166,166,165,165,164,164,163,163,162,162,161,161,160,160,159,159,158,158,157,157,156,156,155,155,154,154,153,153,152,152,151,151,150,150,149,149,148,148,147,147,146,146,145,145,143,143,142,142,141,141,140,140,139,139,138,138,137,137,136,136,135,135,134,134,133,133,132,132,131,131,130,130,129,129,128,128,127,127,126,126,125,125,124,124,123,123,122,122,121,121,120,120,119,119,118,118,117,117,116,116,115,115,114,114,113,113,112,112,111,111,110,110,109,109,108,108,107,107,106,106,105,105,104,104,103,103,102,102,101,101,100,100,99,99,98,98,97,97,96,96,94,94,93,93,92,92,90,90,89,89,87,87,86,86,85,85,83,83,82,82,81,81,80,80,78,78,77,77,75,75,72,72,69,69,68,68,0.0]],"coord\_x":[[0.0,1e-10,0.6222606342836752,0.6222606343836752,1.1961412607660278,1.1961412608660278,1.7594306879814245,1.7594306880814246,2.311317046996509,2.311317047096509,2.8478147994111738,2.8478147995111738,3.3657133726253297,3.3657133727253297,3.8370770947048802,3.8370770948048802,4.283346686117079,4.283346686217079,4.727697314595891,4.727697314695891,5.15282141060751,5.15282141070751,5.560268905751981,5.560268905851981,5.952844438162711,5.952844438262711,6.335493027706503,6.335493027806503,6.695962834116356,6.695962834216356,7.054070839992812,7.054070840092812,7.399484168002252,7.399484168102252,7.743679692611661,7.743679692711661,8.081048137554216,8.081048137654216,8.417346391630074,8.417346391730074,8.749917429239165,8.749917429339165,9.079757634981513,9.079757635081513,9.398231675656884,9.398231675756884,9.7158938473989,9.7158938474989,10.0317108624742,10.0317108625742,10.344575626882751,10.344575626982751,10.652938209024514,10.652938209124514,10.957241446499498,10.957241446599499,11.254791410574297,11.254791410674297,11.54794990178231,11.54794990188231,11.839779880190287,11.839779880290287,12.131167020998252,12.131167021098252,12.421631583472857,12.421631583572857,12.705379775680614,12.705379775780614,12.987024489288313,12.987024489388313,13.268521590362672,13.268521590462672,13.543966577570203,13.543966577670203,13.819190145977725,13.819190146077725,14.092605460851864,14.092605460951864,14.365688647525996,14.365688647625996,14.636483839933398,14.636483840033398,14.904437491074054,14.904437491174054,15.17135785448135,15.17135785458135,15.437577058355293,15.437577058455293,15.701729686762514,15.701729686862514,15.952301962102696,15.952301962202696,16.20169333717618,16.20169333727618,16.447542011449567,16.447542011549565,16.69294784812294,16.69294784822294,16.937947750329638,16.937947750429636,17.18272623373633,17.182726233836327,17.425991688676312,17.42599168877631,17.66759650261625,17.667596502716247,17.907540675556138,17.907540675656136,18.147005107762684,18.147005107862682,18.384845802102518,18.384845802202516,18.61906998937559,18.619069989475587,18.852925145315314,18.852925145415313,19.08541488532167,19.085414885421667,19.316502306261317,19.316502306361315,19.777459344740585,19.777459344840583,20.005594515013485,20.005594515113483,20.22907989048626,20.22907989058626,20.452270040892362,20.45227004099236,20.673356712698403,20.6733567127984,20.894074353171106,20.894074353271105,21.114275349777124,21.114275349877122,21.33436563698314,21.33436563708314,21.553607152122467,21.553607152222465,21.771778476395095,21.771778476495093,21.987329678200986,21.987329678300984,22.202511848673534,22.202511848773533,22.415922668746035,22.415922668846033,22.628484716751846,22.628484716851844,22.84082534595765,22.84082534605765,23.052686234430105,23.052686234530103,23.264436413502562,23.26443641360256,23.47603898004168,23.476038980141677,23.685796389914078,23.685796390014076,23.892822967919738,23.892822968019736,24.099591223992057,24.099591224092055,24.303407229397628,24.303407229497626,24.506669687803182,24.50666968790318,24.709157180408717,24.709157180508715,24.910869707214232,24.91086970731423,25.112250105819736,25.112250105919735,25.31329837622523,25.31329837632523,25.51405142156405,25.51405142166405,25.714177113636186,25.714177113736184,25.913121905441624,25.913121905541622,26.111107215780372,26.11110721588037,26.30787472271908,26.30787472281908,26.50419939205778,26.50419939215778,26.699416967396452,26.69941696749645,26.894191705135107,26.894191705235105,27.088043864540406,27.088043864640404,27.279497320278974,27.279497320378972,27.470803163484202,27.4708031635842,27.660522171956053,27.66052217205605,27.849909052227897,27.849909052327895,28.038742385499727,28.038742385599726,28.22753881563822,28.227538815738217,28.41611382697671,28.416113827076707,28.60413529131518,28.60413529141518,28.791787724320308,28.791787724420306,28.978148547658737,28.978148547758735,29.164361758463826,29.164361758563825,29.35046425986891,29.35046425996891,29.53649295500733,29.536492955107327,29.721968103145734,29.721968103245732,29.9072956387508,29.907295638850798,30.091442274089164,30.091442274189163,30.2755520062942,30.275552006394197,30.459108191499215,30.459108191599213,30.64008115737083,30.640081157470828,30.820574382509097,30.820574382609095,30.99881651651397,30.998816516613967,31.17698484425217,31.17698484435217,31.354968656323702,31.3549686564237,31.532841758995232,31.53284175909523,31.710345830333416,31.710345830433415,31.887812998538266,31.887812998638264,32.064431394676426,32.06443139477643,32.24038553441457,32.24038553451457,32.41597064281937,32.41597064291937,32.59140813869083,32.59140813879083,32.76647660322895,32.766476603328954,32.940327264367035,32.94032726446704,33.112960122105086,33.11296012220509,33.28500252970979,33.285002529809795,33.455864037047796,33.4558640371478,33.62521251591909,33.62521251601909,33.79445028539038,33.794450285490385,33.96247025146164,33.96247025156164,34.12949383293287,34.129493833032875,34.2962959956041,34.2962959957041,34.462839836341985,34.46283983644199,34.6293098708132,34.629309870913204,34.795595389617745,34.79559538971775,34.96121665202227,34.961216652122275,35.126801011293466,35.12680101139347,35.29186872669798,35.29186872679798,35.45649360450248,35.45649360460248,35.62078635410697,35.62078635420697,35.784267234778106,35.78426723487811,35.94686244024921,35.946862440349214,36.109383839453656,36.10938383955366,36.27146240105809,36.27146240115809,36.433356446995845,36.433356447095846,36.593995786400235,36.59399578650024,36.75422919133795,36.75422919143795,36.91424117747565,36.914241177575654,37.073921035413356,37.07392103551336,37.23323186201771,37.23323186211771,37.39080824135535,37.39080824145535,37.54712991415962,37.54712991425962,37.70171713969718,37.70171713979718,37.85600914016806,37.856009140268064,38.01022733437228,38.01022733447228,38.16363365964314,38.16363365974314,38.316929275514,38.316929275614,38.46893328171815,38.46893328181815,38.620531353455625,38.62053135355563,38.77198181265977,38.77198181275977,38.92306324053057,38.92306324063057,39.07362802453468,39.07362802463468,39.22415590540546,39.22415590550546,39.37449927060957,39.37449927070957,39.52458431388034,39.52458431398034,39.673783681951086,39.67378368205109,39.82158073095513,39.82158073105513,39.96900874862582,39.96900874872582,40.26231485236717,40.26231485246717,40.40697513503779,40.40697513513779,40.551450902041736,40.55145090214174,40.69559454084568,40.695594540945685,40.83914772951627,40.83914772961627,40.98266401505352,40.982664015153524,41.125921978657445,41.12592197875745,41.267777623194654,41.267777623294656,41.40948565519852,41.409485655298525,41.55067704333572,41.55067704343572,41.69101965940622,41.691019659506225,41.83029208461003,41.83029208471003,41.9695276066805,41.9695276067805,42.247740328888106,42.24774032898811,42.386606819625236,42.38660681972524,42.525399504095695,42.5253995041957,42.66374935096614,42.663749351066144,42.80180397276992,42.80180397286992,42.93971098204035,42.939710982140355,43.07735966937745,43.07735966947745,43.214750034781204,43.214750034881206,43.35066427485158,43.35066427495158,43.48646780552196,43.48646780562196,43.620795210858965,43.62079521095897,43.75490119739597,43.75490119749597,43.887973896199604,43.887973896299606,44.020714466803234,44.020714466903236,44.15286458727351,44.152864587373514,44.284682579543784,44.284682579643786,44.41646366868072,44.41646366878072,44.547580501417634,44.547580501517636,44.67843901222121,44.67843901232121,44.80885468542478,44.80885468552478,44.939012036695,44.939012036795,45.06876345349855,45.06876345359855,45.19722326063539,45.19722326073539,45.32531403643889,45.32531403653889,45.45307268404238,45.453072684142384,45.58049920344587,45.58049920354587,45.706707919449315,45.706707919549316,45.832658313519424,45.832658313619426,45.957907548056184,45.957907548156186,46.082861557526265,46.082861557626266,46.20667156986298,46.206671569962985,46.33040777593303,46.330407776033034,46.45407017573641,46.454070175836414,46.577252834806444,46.577252834906446,46.700361687609806,46.70036168770981,46.82273247774649,46.82273247784649,46.94473423654982,46.94473423664982,47.066662189086486,47.06666218918649,47.18836872282315,47.18836872292315,47.30981693462647,47.30981693472647,47.43111753389645,47.43111753399645,47.55193839243309,47.55193839253309,47.672685444703056,47.67268544480306,47.792657531173,47.792657531273,47.91207607064293,47.912076070742934,48.0304613223795,48.0304613224795,48.1487727678494,48.1487727679494,48.267047310185966,48.26704731028597,48.38506353058919,48.385063530689195,48.502895235325745,48.50289523542575,48.620357908728955,48.62035790882896,48.73778367899883,48.73778367909883,48.85513564300204,48.855135643102045,48.972450703871914,48.972450703971916,49.08954434594178,49.089544346041784,49.206379666078305,49.20637966617831,49.323067373681496,49.3230673737815,49.439090824884666,49.43909082498467,49.55492976042117,49.55492976052117,49.670731792824334,49.670731792924336,49.786423115827496,49.7864231159275,49.90204063256399,49.90204063266399,50.016403442767114,50.016403442867116,50.13021270597022,50.130212706070225,50.24343151903999,50.24343151913999,50.35528491617638,50.35528491627638,50.46610502557941,50.46610502567941,50.57644539424909,50.57644539434909,50.68667505351877,50.68667505361877,50.796461875188434,50.796461875288436,50.90562134359142,50.90562134369142,51.01415345872772,51.01415345882772,51.12257486446402,51.12257486456402,51.23073794826697,51.23073794836697,51.446953406472886,51.44695340657289,51.55426771820915,51.55426771830915,51.66132370801208,51.66132370811208,51.875398784484595,51.8753987845846,51.98238096802085,51.98238096812085,52.089252442157104,52.089252442257106,52.196013206893355,52.19601320699336,52.302072812096256,52.30207281219626,52.40805861103249,52.40805861113249,52.51356466923537,52.51356466933537,52.61873859923825,52.61873859933825,52.72350659477444,52.72350659487444,52.8280162683773,52.8280162684773,53.036961809316345,53.03696180941635,53.14132387038586,53.14132387048586,53.24542760952204,53.24542760962204,53.34894089852487,53.348940898624875,53.452417284394365,53.452417284494366,53.55585676713053,53.55585676723053,53.65914863733335,53.659148637433354,53.76232979813617,53.76232979823617,53.86506812133898,53.86506812143898,53.967511219475114,53.967511219575115,54.06940077061123,54.06940077071123,54.171031999814005,54.17103199991401,54.271962069483436,54.27196206958344,54.37256001095285,54.372560011052855,54.472198470955576,54.47219847105558,54.57143099649162,54.57143099659162,54.67047900636099,54.670479006460994,54.76904727549702,54.769047275597025,54.86735722269971,54.86735722279971,55.06353427950507,55.06353427960507,55.16114306717441,55.16114306727441,55.356286836246404,55.356286836346406,55.45315756124905,55.45315756134905,55.54980686745169,55.549806867551695,55.646308561120996,55.646308561221,55.7426995453903,55.742699545490304,55.83831556385958,55.83831556395958,55.93378396979552,55.93378396989552,56.0291785694648,56.0291785695648,56.12409342840073,56.12409342850073,56.21893448106999,56.21893448116999,56.31362792120591,56.31362792130591,56.408099942541824,56.408099942641826,56.501944610611055,56.50194461071106,56.5951619254136,56.5951619255136,56.688268530816146,56.68826853091615,56.78107991115202,56.78107991125202,56.87293181002119,56.872931810121194,56.96467299949037,56.96467299959037,57.056266576426204,57.056266576526205,57.14767563769537,57.14767563779537,57.238826377031195,57.2388263771312,57.329608085033676,57.32960808513368,57.41998385856948,57.41998385866948,57.510212019571945,57.51021201967195,57.600403277441075,57.600403277541076,57.69052072904354,57.69052072914354,57.780306052445994,57.780306052545995,57.86998066644845,57.86998066654845,57.95943386165089,57.959433861750895,58.048850153720004,58.048850153820005,58.13789741445577,58.13789741455577,58.22672325639153,58.22672325649153,58.315327679527286,58.31532767962729,58.40326784626302,58.40326784636302,58.49083898166542,58.49083898176542,58.57759824813446,58.57759824823446,58.66380396760348,58.66380396770348,58.749862074539166,58.74986207463917,58.835698762674845,58.83569876277485,58.921314032010514,58.921314032110516,59.00644956061284,59.006449560712845,59.34673335308881,59.34673335318881,59.431278431557786,59.43127843165779,59.51567589749343,59.51567589759343,59.60003646029573,59.600036460395735,59.68428631369804,59.68428631379804,59.768314748300334,59.768314748400336,59.85226937663596,59.85226937673596,59.93607639243825,59.936076392538254,60.019219151840524,60.019219151940526,60.10217739557613,60.10217739567613,60.18484041424505,60.184840414345054,60.267429626647306,60.26742962674731,60.34983432338289,60.349834323482895,60.43209140758514,60.43209140768514,60.51409016985405,60.51409016995405,60.596052028989625,60.59605202908963,60.677903178725195,60.6779031788252,60.75945910339409,60.759459103494095,60.84060909359631,60.840609093696315,60.9206519897985,60.9206519898985,61.000252048400675,61.00025204850068,61.07970449446951,61.07970449456951,61.15908313427168,61.159083134371684,61.238092742740506,61.23809274284051,61.317028544942666,61.31702854504267,61.395890540878156,61.39589054097816,61.55339311394913,61.55339311404913,61.63188607855127,61.63188607865127,61.709936205553404,61.709936205653406,61.787728010622196,61.7877280107222,61.94320091135978,61.94320091145978,62.020512975695226,62.02051297579523,62.09705007423065,62.09705007433065,62.173365753966074,62.173365754066076,62.24960762743482,62.24960762753482,62.32573879150357,62.32573879160357,62.40164853677231,62.401648536872315,62.47741066950772,62.47741066960772,62.552951383443116,62.55295138354312,62.62771713157849,62.627717131678494,62.702372170313865,62.70237217041387,62.7769903059159,62.776990306015904,62.92618967398665,62.92618967408665,63.075315235790725,63.07531523589073,63.14963814632609,63.14963814642609,63.223739638061446,63.22373963816145,63.371536687065486,63.37153668716549,63.518890898469515,63.518890898569516,63.59236503693819,63.59236503703819,63.66572846600686,63.66572846610686,63.73901808880887,63.73901808890887,63.812123195944196,63.8121231960442,63.885117593679524,63.885117593779526,64.03095877661684,64.03095877671684,64.10306749915215,64.10306749925215,64.17510241542078,64.17510241552078,64.24702662228943,64.24702662238943,64.39061671409334,64.39061671419334,64.46165524576195,64.46165524586195,64.53236164923055,64.53236164933055,64.60295734329915,64.60295734339915,64.67347923110107,64.67347923120107,64.743890409503,64.743890409603,64.81426468477159,64.81426468487159,64.95497633217543,64.95497633227544,65.02476015731068,65.02476015741068,65.09450707931258,65.09450707941258,65.16421709818115,65.16421709828116,65.2338902139164,65.2338902140164,65.30337881398496,65.30337881408497,65.37279360778686,65.37279360788686,65.44206078905542,65.44206078915542,65.64938259212775,65.64938259222775,65.7179855169963,65.7179855170963,65.78640392619816,65.78640392629816,65.85471162600003,65.85471162610003,65.99121631620376,65.99121631630376,66.05926569407228,66.05926569417228,66.12724126567414,66.12724126577415,66.19517993414267,66.19517993424267,66.26286028067786,66.26286028077786,66.3303930146797,66.3303930147797,66.3977781361482,66.3977781362482,66.46453590435003,66.46453590445003,66.53118296315185,66.53118296325185,66.59749789375367,66.59749789385367,66.6632961804888,66.6632961805888,66.7290575640906,66.7290575641906,66.79463443202572,66.79463443212572,66.86002678429418,66.86002678439418,66.99059007003108,66.99059007013108,67.12045219623462,67.12045219633463,67.18492197016973,67.18492197026973,67.24924413157147,67.24924413167147,67.3134186804399,67.3134186805399,67.37751942304165,67.37751942314165,67.4415094562434,67.4415094563434,67.50538878004515,67.50538878014515,67.56915739444689,67.56915739454689,67.6328891057153,67.6328891058153,67.6963993981837,67.6963993982837,67.75987278751877,67.75987278761878,67.82323546745384,67.82323546755384,67.8863767285889,67.8863767286889,67.94944418345729,67.94944418355729,68.01247473519234,68.01247473529234,68.07546838379406,68.07546838389406,68.13842512926244,68.13842512936245,68.2013449715975,68.20134497169751,68.26385887946589,68.26385887956589,68.32589304660091,68.32589304670091,68.3878903106026,68.3878903107026,68.51177412920599,68.51177412930599,68.57362378067434,68.57362378077434,68.63532581960936,68.63532581970937,68.69680643974438,68.69680643984438,68.75813944734605,68.75813944744606,68.81928793928105,68.81928793938106,68.88039952808273,68.88039952818274,68.9414004074844,68.9414004075844,69.00225367435273,69.00225367445273,69.0630331349544,69.0630331350544,69.12362807988939,69.12362807998939,69.1841492185577,69.18414921865771,69.24459655095936,69.24459655105936,69.30500698022767,69.30500698032768,69.4257171293643,69.4257171294643,69.48586923669929,69.48586923679929,69.54587373150092,69.54587373160092,69.60584132316923,69.60584132326923,69.6656613023042,69.6656613024042,69.7252967657725,69.7252967658725,69.78463700417412,69.78463700427412,69.84382963004239,69.8438296301424,69.96217797864563,69.96217797874563,70.08041561784887,70.08041561794887,70.13905469671714,70.13905469681714,70.1975830661854,70.1975830662854,70.25574240432033,70.25574240442033,70.31382793618857,70.31382793628858,70.37161824299015,70.37161824309015,70.42929784039174,70.42929784049174,70.48686672839331,70.48686672849331,70.60170927932978,70.60170927942978,70.65883532973135,70.65883532983135,70.71592447699958,70.71592447709958,70.77249698040112,70.77249698050112,70.82895877440266,70.82895877450267,70.8844610869375,70.88446108703751,70.93981578693902,70.93981578703902,70.9950228744072,70.9950228745072,71.05019305874204,71.05019305884204,71.1051787274102,71.10517872751021,71.16012749294504,71.16012749304504,71.21496554907988,71.21496554917988,71.2696928958147,71.2696928959147,71.32419882374953,71.32419882384953,71.37829881721767,71.37829881731767,71.43236190755249,71.43236190765249,71.48605596655395,71.48605596665395,71.59314885949021,71.59314885959022,71.64651079029167,71.64651079039167,71.69965130229312,71.69965130239312,71.75275491116125,71.75275491126125,71.80571090749602,71.80571090759602,71.85826096936412,71.85826096946413,71.96317657743366,71.96317657753366,72.01539451110176,72.01539451120176,72.06753863850318,72.06753863860318,72.11949825023794,72.11949825033794,72.17138405570601,72.17138405580602,72.2231591517741,72.2231591518741,72.32667244077693,72.32667244087693,72.37789398984499,72.377893989945,72.4290417326464,72.4290417327464,72.47993115351446,72.47993115361446,72.53056225244917,72.53056225254917,72.58111954511722,72.58111954521722,72.63138161271858,72.63138161281859,72.68160677718663,72.68160677728663,72.731758135388,72.73175813548801,72.78168807478937,72.78168807488937,72.83143349852406,72.83143349862407,72.88103130972542,72.88103130982542,72.93059221779343,72.93059221789343,72.97989480392812,72.97989480402812,73.02912358379614,73.02912358389614,73.07809404173081,73.07809404183081,73.22496851240149,73.22496851250149,73.27371755153615,73.27371755163615,73.32239278440414,73.32239278450415,73.37073588907214,73.37073588917214,73.41874686554011,73.41874686564012,73.46668403574142,73.46668403584142,73.51451049654273,73.51451049664273,73.5621893448107,73.5621893449107,73.60983128994533,73.60983129004534,73.70504137394794,73.70504137404794,73.75257260968257,73.75257260978258,73.80003003915053,73.80003003925053,73.84711843728516,73.84711843738516,73.89405922288644,73.89405922298644,73.94077858968771,73.94077858978771,73.98746105335566,73.98746105345566,74.03406971075694,74.03406971085694,74.08042004622486,74.08042004632486,74.12640135035946,74.12640135045946,74.17219813882737,74.17219813892737,74.21795802416196,74.21795802426196,74.2634595875632,74.2634595876632,74.30892424783111,74.30892424793112,74.35424129556569,74.35424129566569,74.39941073076692,74.39941073086692,74.44454326283481,74.44454326293481,74.48952818236938,74.48952818246939,74.62444603783973,74.62444603793973,74.66935715110763,74.66935715120763,74.71389923304218,74.71389923314219,74.7582937024434,74.7582937025434,74.89114498244703,74.89114498254703,74.9351704205149,74.9351704206149,74.97897443978276,74.97897443988276,75.02266774965062,75.02266774975062,75.06628725325182,75.06628725335182,75.15337864792086,75.15337864802086,75.1965184107887,75.1965184108887,75.28276103339107,75.28276103349107,75.32575318372557,75.32575318382557,75.36870843092674,75.36870843102675,75.41144225932791,75.41144225942791,75.45406537832908,75.45406537842908,75.49661469106357,75.49661469116357,75.53813071606471,75.53813071616472,75.5794991285325,75.5794991286325,75.6207568316003,75.6207568317003,75.66197763153475,75.66197763163476,75.70316152833588,75.70316152843588,75.74423471573701,75.74423471583701,75.78508648433812,75.78508648443812,75.86642099020702,75.86642099030702,75.90686682434145,75.90686682444145,75.94723885220922,75.94723885230923,75.98757397694366,75.98757397704367,76.02787219854476,76.02787219864476,76.06805971074586,76.06805971084586,76.10817341668029,76.10817341678029,76.14810260694804,76.14810260704805,76.1876996690158,76.1876996691158,76.22674318408353,76.22674318418353,76.2657128928846,76.2657128929846,76.30438737661899,76.30438737671899,76.38151492528776,76.38151492538776,76.41974657142214,76.41974657152214,76.45794131442318,76.45794131452318,76.5342569941586,76.5342569942586,76.57219341522631,76.57219341532631,76.64777103229504,76.64777103239504,76.68537532516274,76.68537532526274,76.72279510236376,76.72279510246376,76.76003036389811,76.76003036399811,76.79711801289912,76.79711801299912,76.8340580493668,76.8340580494668,76.8709242795678,76.8709242796678,76.90775360663548,76.90775360673548,76.94425080550315,76.94425080560315,76.98071110123747,76.98071110133748,77.01698688130513,77.01698688140513,77.05322575823945,77.05322575833945,77.08939082890711,77.08939082900712,77.1254820933081,77.1254820934081,77.16135193890908,77.16135193900908,77.19718488137673,77.19718488147673,77.2329440175777,77.2329440176777,77.268629347512,77.268629347612,77.30416706491297,77.30416706501298,77.33966787918062,77.33966787928063,77.3745782433149,77.3745782434149,77.4792355294511,77.4792355295511,77.54879793578634,77.54879793588634,77.58348688112062,77.58348688122062,77.65275406238918,77.65275406248918,77.68722158892345,77.68722158902345,77.72165221232439,77.72165221242439,77.75586141692533,77.75586141702533,77.78970159019292,77.78970159029292,77.85734503359477,77.85734503369477,77.89081617552901,77.89081617562901,77.95768465313084,77.95768465323084,77.99100818253176,77.99100818263176,78.02422100253266,78.02422100263266,78.05739691940023,78.05739691950023,78.09053593313448,78.09053593323448,78.12341662493537,78.12341662503538,78.1561128010696,78.1561128011696,78.1886613646705,78.1886613647705,78.2210254126047,78.2210254127047,78.25309423547225,78.25309423557225,78.28512615520646,78.28512615530646,78.34915309154154,78.34915309164154,78.38107430187576,78.38107430197576,78.41281099654329,78.41281099664329,78.44417865987748,78.44417865997748,78.50684018027918,78.50684018037919,78.53809713421337,78.53809713431338,78.56931718501423,78.56931718511423,78.60035272014841,78.60035272024841,78.63135135214925,78.63135135224925,78.69331171301762,78.69331171311762,78.72397821681845,78.72397821691845,78.75460781748596,78.75460781758596,78.81564560002096,78.81564560012096,78.87638815748929,78.87638815758929,78.90668562995678,78.90668563005678,78.93694619929094,78.93694619939095,78.9670960592251,78.9670960593251,78.99695069409259,78.99695069419259,79.02665771642673,79.02665771652673,79.0561802230942,79.0561802231942,79.08559202036167,79.08559202046168,79.11496691449581,79.11496691459581,79.1440834866966,79.1440834867966,79.17316315576407,79.17316315586407,79.26036525983312,79.26036525993312,79.28933421950057,79.28933421960058,79.3181555666347,79.3181555667347,79.3757613577696,79.3757613578696,79.40447199550373,79.40447199560373,79.43299811757117,79.43299811767118,79.46148733650529,79.46148733660529,79.48990274917273,79.48990274927273,79.5181336461735,79.5181336462735,79.54621693064092,79.54621693074093,79.5742264088417,79.5742264089417,79.60212517764246,79.60212517774247,79.65781200584398,79.65781200594398,79.68556316211141,79.68556316221141,79.71309289957883,79.71309289967883,79.74054883077957,79.74054883087958,79.76789405258032,79.76789405268032,79.79509166184774,79.79509166194774,79.82221546484847,79.82221546494847,79.84930236471588,79.84930236481588,79.87635236144996,79.87635236154996,79.90318093938401,79.90318093948402,79.92993571105141,79.92993571115142,79.95657977331881,79.95657977341881,79.98315002931955,79.98315002941955,80.00968338218694,80.00968338228694,80.03610602565432,80.03610602575432,80.06227034718837,80.06227034728838,80.08828705618907,80.08828705628908,80.11408234638978,80.11408234648978,80.13984073345715,80.13984073355715,80.16548841112453,80.16548841122453,80.21674686332592,80.21674686342593,80.24232073472662,80.24232073482662,80.26767318732732,80.26767318742732,80.31823047999536,80.31823048009537,80.34336151379605,80.34336151389606,80.4186439057981,80.41864390589811,80.44322139259879,80.44322139269879,80.46772507313278,80.46772507323278,80.49215494740012,80.49215494750013,80.51654791853412,80.51654791863412,80.54083018026812,80.54083018036812,80.56500173260211,80.56500173270211,80.58909947866944,80.58909947876944,80.61293890280342,80.61293890290342,80.63670452067073,80.63670452077073,80.70796447113935,80.70796447123935,80.73161937960667,80.73161937970667,80.77889229340795,80.77889229350795,80.80221507367526,80.80221507377526,80.82550095080923,80.82550095090923,80.84874992480987,80.84874992490987,80.87196199567717,80.87196199577717,80.89506335714447,80.89506335724447,80.91790639667842,80.91790639677842,80.94056492054571,80.94056492064571,80.96314963814632,80.96314963824632,80.9856974526136,80.98569745271361,81.00820836394756,81.00820836404756,81.03060856588151,81.03060856598151,81.05293496154879,81.05293496164879,81.07511374468272,81.07511374478273,81.09721872154999,81.09721872165,81.11913918275059,81.11913918285059,81.14091203141786,81.14091203151786,81.16250036441843,81.16250036451844,81.1838303754857,81.1838303755857,81.20512348341961,81.20512348351961,81.24759898988744,81.24759898998744,81.26874448528801,81.26874448538801,81.28977927128858,81.28977927138858,81.31077715415583,81.31077715425583,81.33155361822307,81.33155361832307,81.35229317915696,81.35229317925696,81.37292203069086,81.37292203079086,81.43473477902589,81.43473477912589,81.45510530862644,81.45510530872644,81.47529132256032,81.47529132266033,81.49540353022753,81.49540353032754,81.51544193162809,81.51544193172809,81.53540652676197,81.53540652686198,81.55526041249584,81.55526041259584,81.57507739509639,81.57507739519639,81.59474676516359,81.5947467652636,81.63393789276466,81.63393789286467,81.67272308589905,81.67272308599905,81.69176510269958,81.69176510279958,81.72977533003395,81.72977533013395,81.74870663743447,81.74870663753447,81.76756413856832,81.76756413866832,81.7863478334355,81.7863478335355,81.80498391576934,81.80498391586934,81.82339857930317,81.82339857940318,81.84177633970367,81.84177633980367,81.86008029383751,81.86008029393751,81.87831044170467,81.87831044180467,81.91462312490566,81.91462312500566,81.95075129243999,81.95075129253999,81.96872311837382,81.96872311847382,81.9865473317743,81.9865473318743,82.00429773890812,82.00429773900812,82.0220112429086,82.0220112430086,82.03957713437575,82.03957713447575,82.05703231644289,82.05703231654289,82.07430298284336,82.07430298294337,82.0913153273105,82.0913153274105,82.10814315611096,82.10814315621096,82.15847902997899,82.158479030079,82.17508543997945,82.17508544007946,82.19165494684657,82.19165494694657,82.20818755058036,82.20818755068036,82.22438802611413,82.22438802621413,82.25667826778168,82.25667826788168,82.27273113078212,82.27273113088212,82.28871018751589,82.28871018761589,82.33661045458386,82.33661045468386,82.3524418987843,82.35244189888431,82.36819953671807,82.36819953681807,82.38392027151849,82.38392027161849,82.3996041031856,82.3996041032856,82.41517722545268,82.41517722555268,82.43063963831978,82.43063963841978,82.44602824492019,82.44602824502019,82.46134304525394,82.46134304535394,82.47662094245436,82.47662094255436,82.49186193652145,82.49186193662145,82.50691841492186,82.50691841502186,82.53699446858934,82.53699446868934,82.55197714072308,82.55197714082308,82.5669229097235,82.5669229098235,82.58183177559057,82.58183177569057,82.59666683519097,82.59666683529097,82.61146499165805,82.61146499175806,82.62622624499178,82.62622624509179,82.64080298265885,82.64080298275886,82.6697719423263,82.66977194242631,82.68412726119337,82.68412726129337,82.6984456769271,82.6984456770271,82.71272718952748,82.71272718962749,82.7412164084616,82.7412164085616,82.75527650226198,82.75527650236198,82.76929969292902,82.76929969302903,82.79723536486313,82.79723536496313,82.82483890859721,82.82483890869722,82.85207342099795,82.85207342109796,82.86557996779833,82.86557996789833,82.87904961146536,82.87904961156536,82.89244544886573,82.89244544896573,82.90580438313276,82.90580438323276,82.91912641426646,82.91912641436646,82.93222702660015,82.93222702670015,82.94521692953384,82.94521692963384,82.95805921993419,82.95805922003419,82.97082770406787,82.97082770416787,82.98348547880155,82.98348547890156,82.99610635040189,82.99610635050189,83.03389515893626,83.03389515903626,83.04636841800327,83.04636841810327,83.05850954887026,83.05850954897026,83.08268110120426,83.08268110130426,83.09456391013792,83.09456391023792,83.10626220340491,83.10626220350491,83.14132018007253,83.14132018017253,83.15287086080617,83.15287086090618,83.16438463840649,83.16438463850649,83.17586151287347,83.17586151297347,83.18730148420713,83.18730148430713,83.21010762060774,83.21010762070775,83.22139997940805,83.22139997950805,83.2326185319417,83.2326185320417,83.24372637507533,83.24372637517533,83.25479731507563,83.25479731517564,83.26568373940927,83.26568373950927,83.27649635747622,83.27649635757622,83.29797398107681,83.29797398117681,83.32978448201101,83.32978448211101,83.35070855861159,83.35070855871159,83.36107833907853,83.36107833917853,83.38170719061243,83.38170719071243,83.39192935854605,83.39192935864605,83.40211462334632,83.40211462344632,83.42244824981354,83.42244824991354,83.46300479334799,83.463004793448,83.47307934874826,83.47307934884826,83.48304319474853,83.48304319484853,83.49297013761547,83.49297013771547,83.50282327421574,83.50282327431574,83.51263950768268,83.51263950778268,83.52234503174961,83.52234503184961,83.53193984641653,83.53193984651654,83.54149775795014,83.54149775805014,83.5510187663504,83.5510187664504,83.57954488841784,83.57954488851784,83.5889551874181,83.5889551875181,83.60770197915194,83.60770197925194,83.62626425521911,83.62626425531911,83.64467891875296,83.64467891885296,83.66290906662012,83.66290906672012,83.67176581862036,83.67176581872036,83.68058566748726,83.68058566758727,83.68936861322084,83.68936861332084,83.69811465582109,83.69811465592109,83.71556983788822,83.71556983798823,83.72416826795512,83.72416826805512,83.7413282249556,83.7413282250556,83.74985284875584,83.74985284885584,83.7753898170232,83.7753898171232,83.79232835522366,83.79232835532366,83.80066846335721,83.80066846345721,83.80893476522411,83.80893476532411,83.81712726082434,83.81712726092434,83.82528285329123,83.82528285339123,83.83336463949145,83.83336463959145,83.84140952255834,83.84140952265834,83.84934369622522,83.84934369632522,83.85724096675877,83.85724096685877,83.86510133415898,83.86510133425898,83.88078516582607,83.88078516592607,83.88857172695963,83.88857172705963,83.89632138495983,83.89632138505984,83.91945964956047,83.91945964966047,83.927024791894,83.927024791994,83.94211817342776,83.94211817352776,83.9571008455615,83.9571008456615,83.96451837536169,83.9645183754617,83.97189900202856,83.97189900212857,83.9792427255621,83.9792427256621,83.99389326949583,83.99389326959583,84.0157768275631,84.0157768276631,84.03027975896349,84.03027975906349,84.04470888409722,84.04470888419722,84.05906420296428,84.05906420306428,84.07334571556467,84.07334571566467,84.09467572663192,84.09467572673192,84.10880962669897,84.10880962679897,84.11574741576582,84.11574741586583,84.13652387983306,84.13652387993307,84.14335095949991,84.14335095959991,84.15014113603344,84.15014113613344,84.17726493903417,84.17726493913418,84.18398130930103,84.18398130940103,84.21752625750194,84.21752625760195,84.23084828863564,84.23084828873564,84.2374539495025,84.2374539496025,84.244022707236,84.244022707336,84.27015012563672,84.27015012573672,84.28295551290374,84.28295551300374,84.28930285183723,84.28930285193724,84.32734998230495,84.32734998240495,84.33362351497179,84.33362351507179,84.3649173720393,84.3649173721393,84.37727992170632,84.37727992180632,84.40189431164032,84.40189431174032,84.42636108904098,84.42636108914098,84.44459123690815,84.44459123700815,84.45665856150848,84.45665856160848,84.48669771204264,84.48669771214264,84.5045957317098,84.5045957318098,84.52832444644378,84.52832444654378,84.54009654597743,84.54009654607744,84.55766243744458,84.55766243754458,84.5692869244449,84.5692869245449,84.59821898097901,84.59821898107901,84.60969585544599,84.609695855546,84.61537893797949,84.61537893807949,84.62102511737965,84.62102511747965,84.64921911124708,84.64921911134708,84.65479148438057,84.65479148448057,84.67704407378118,84.67704407388118,84.69361358064829,84.69361358074829,84.72106951184904,84.72106951194904,84.72649427244919,84.72649427254919,84.74815641171645,84.74815641181645,84.77505879591719,84.77505879601719,84.780335943984,84.780335944084,84.7961304850511,84.7961304851511,84.81181431671818,84.81181431681819,84.82738743898528,84.82738743908529,84.84284985185238,84.84284985195238,84.85820155531945,84.85820155541946,84.87344254938654,84.87344254948654,84.90879575112083,84.90879575122084,84.92883415252139,84.92883415262139,84.95370686438874,84.95370686448874,84.963560000989,84.96356000108901,84.988063681523,84.988063681623,84.99776920558993,84.99776920568993,85.0267381652574,85.0267381653574,85.04589089145792,85.04589089155792,85.08869852612575,85.08869852622576,85.11700322939319,85.11700322949319,85.14508651386063,85.14508651396063,85.18689776392844,85.18689776402844,85.21453821079587,85.21453821089587,85.2511092159302,85.2511092160302,85.28738499599785,85.28738499609786,85.32336555099884,85.32336555109885,85.35905088093315,85.35905088103316,85.38558423380054,85.38558423390054,85.42946205933507,85.42946205943507,85.4599071443359,85.4599071444359,85.49872924060364,85.49872924070364,85.5500614990717,85.5500614991717,85.59670705960632,85.59670705970632,85.65136060007447,85.65136060017447,85.7013643457425,85.7013643458425,85.75505840474398,85.75505840484398,85.79188773181164,85.79188773191164,85.84868165401319,85.8486816541132,85.89691404928118,85.89691404938118,85.92876145334871,85.92876145344871,85.97610817341668,85.97610817351668,86.03474725228494,86.03474725238495,86.06570898115247,86.06570898125247,86.11940304015393,86.11940304025393,86.17638147802215,86.17638147812215,86.22904224929026,86.22904224939026,86.60918142576732,86.60918142586732,86.66818953596893,86.66818953606894,86.7010333246365,86.7010333247365,86.72631197097051,86.72631197107052,86.74417308750434,86.74417308760434,86.75827008443805,86.75827008453805,86.77210875943844,86.77210875953844,86.77893583910529,86.77893583920529,86.79244238590566,86.79244238600566,86.8023693287726,86.8023693288726,86.80886428023945,86.80886428033945,86.8120379497062,86.8120379498062,86.81834838550637,86.81834838560637,86.82141134557313,86.82141134567313,86.83056332264003,86.83056332274003,86.84263064724037,86.84263064734037,86.84558289790711,86.84558289800711,86.84846134230719,86.84846134240719,86.85130288357394,86.85130288367394,86.8597537011075,86.8597537012075,86.86241072670757,86.86241072680757,86.86761406850772,86.86761406860772,86.87266979777452,86.87266979787452]],"filenames":["genome.ctg.filtered"]}

{"coord\_y":[[83.19471901400732,83.19471901400732,83.71737809142161,83.71737809142161,84.17641616696748,84.17641616696748,84.6230178865797,84.6230178865797,84.8731842274532,84.8731842274532,85.09312690212587,85.09312690212587,85.28886112133122,85.28886112133122,85.47507433213632,85.47507433213632,85.64305739507424,85.64305739507424,85.79546733574507,85.79546733574507,85.93876220248232,85.93876220248232,86.0753037958194,86.0753037958194,86.15959055235503,86.15959055235503,86.24151550835727,86.24151550835727,86.316650287826,86.316650287826,86.38152599622777,86.38152599622777,86.43983294689602,86.43983294689602,86.49467100303086,86.49467100303086,86.54784841816564,86.54784841816564,86.5902501183668,86.5902501183668,86.63180304650128,86.63180304650128,86.66822643910227,86.66822643910227,86.70132854970316,86.70132854970316,86.73384021017073,86.73384021017073,86.76266155730485,86.76266155730485,86.79148290443896,86.79148290443896,86.81853290117304,86.81853290117304,86.8428889691737,86.8428889691737,86.85717048177409,86.85717048177409,86.86580581497434,86.86580581497434,86.87266979777452,86.87266979777452]],"coord\_x":[[0,1,1,2,2,3,3,4,4,5,5,6,6,7,7,8,8,9,9,10,10,11,11,12,12,13,13,14,14,15,15,16,16,17,17,18,18,19,19,20,20,21,21,22,22,23,23,24,24,25,25,26,26,27,27,28,28,29,29,30,30,31]],"filenames":["genome.ctg.filtered"]}

{{ genesInContigs }}

{{ operonsInContigs }}

[{{ num\_contigs }},
{{ Largest\_alignment }},
{{ Total\_aligned\_length }},
{{ num\_misassemblies }},
{{ Misassembled\_contigs\_length }},
{{ num\_mismatches\_per\_100\_kbp }},
{{ num\_indels\_per\_100\_kbp }},
{{ num\_N's\_per\_100\_kbp }},
{{ Genome\_fraction }},
{{ Duplication\_ratio }},
{{ NGA50 }}]

{{ allMisassemblies }}

{{ krona }}

{"list\_of\_GC\_distributions":[[[0.0,1.0,2.0,3.0,4.0,5.0,6.0,7.0,8.0,9.0,10.0,11.0,12.0,13.0,14.0,15.0,16.0,17.0,18.0,19.0,20.0,21.0,22.0,23.0,24.0,25.0,26.0,27.0,28.0,29.0,30.0,31.0,32.0,33.0,34.0,35.0,36.0,37.0,38.0,39.0,40.0,41.0,42.0,43.0,44.0,45.0,46.0,47.0,48.0,49.0,50.0,51.0,52.0,53.0,54.0,55.0,56.0,57.0,58.0,59.0,60.0,61.0,62.0,63.0,64.0,65.0,66.0,67.0,68.0,69.0,70.0,71.0,72.0,73.0,74.0,75.0,76.0,77.0,78.0,79.0,80.0,81.0,82.0,83.0,84.0,85.0,86.0,87.0,88.0,89.0,90.0,91.0,92.0,93.0,94.0,95.0,96.0,97.0,98.0,99.0,100.0],[1,0,0,0,0,0,0,0,0,1,1,0,8,22,32,51,43,63,119,169,291,395,522,707,897,1177,1399,1614,1944,2067,2388,2393,2227,2223,2085,2027,1823,1634,1410,1098,896,745,499,361,300,182,124,90,66,44,45,24,4,8,12,4,3,1,3,2,1,1,0,0,0,0,0,0,0,0,0,0,0,0,0,0,0,0,0,0,0,0,0,0,0,0,0,0,0,0,0,0,0,0,0,1,0,0,0,1,0]],[[0.0,1.0,2.0,3.0,4.0,5.0,6.0,7.0,8.0,9.0,10.0,11.0,12.0,13.0,14.0,15.0,16.0,17.0,18.0,19.0,20.0,21.0,22.0,23.0,24.0,25.0,26.0,27.0,28.0,29.0,30.0,31.0,32.0,33.0,34.0,35.0,36.0,37.0,38.0,39.0,40.0,41.0,42.0,43.0,44.0,45.0,46.0,47.0,48.0,49.0,50.0,51.0,52.0,53.0,54.0,55.0,56.0,57.0,58.0,59.0,60.0,61.0,62.0,63.0,64.0,65.0,66.0,67.0,68.0,69.0,70.0,71.0,72.0,73.0,74.0,75.0,76.0,77.0,78.0,79.0,80.0,81.0,82.0,83.0,84.0,85.0,86.0,87.0,88.0,89.0,90.0,91.0,92.0,93.0,94.0,95.0,96.0,97.0,98.0,99.0,100.0],[0,0,0,0,0,0,0,0,0,0,0,0,4,6,12,21,38,38,63,107,185,271,355,457,549,787,947,1076,1312,1477,1602,1744,1791,1901,1832,1792,1590,1436,1205,1061,851,649,515,376,284,186,131,110,66,59,48,39,23,19,23,18,13,8,7,8,3,2,1,0,0,0,0,0,0,0,0,0,0,0,0,0,0,0,0,0,0,0,0,0,0,0,0,0,0,0,0,0,0,0,0,0,0,0,0,0,0]]],"reference\_index":1,"list\_of\_GC\_contigs\_distributions":[[[0,5,10,15,20,25,30,35,40,45,50,55,60,65,70,75,80,85,90,95,100],[1,0,53,116,740,2560,2409,717,165,85,5,2,0,0,0,0,0,0,0,1,0]]],"lists\_of\_gc\_info":null,"filenames":["genome.ctg.filtered"]}

{"links\_names":["View in Icarus contig browser"],"links":["icarus\_viewers/alignment\_viewer.html"]}

{
"# contigs" : "is the total number of contigs in the assembly.",
"Largest contig" : "is the length of the longest contig in the assembly.",
"Total length" : "is the total number of bases in the assembly.",
"Reference length" : "is the total number of bases in the reference.",
"# contigs (>= 0 bp)" : "is the total number of contigs in the assembly that have size greater than or equal to 0 bp.",
"Total length (>= 0 bp)" : "is the total number of bases in the contigs having size greater than or equal to 0 bp.",
"N50" : "is the contig length such that using longer or equal length contigs produces half (50%) of the bases of the assembly. Usually there is no value that produces exactly 50%, so the technical definition is the maximum length x such that using contigs of length at least x accounts for at least 50% of the total assembly length.",
"NG50" : "is the contig length such that using longer or equal length contigs produces half (50%) of the bases of the reference genome. This metric is computed only if a reference genome is provided.",
"N75" : "is the contig length such that using longer or equal length contigs produces 75% of the bases of the assembly. Usually there is no value that produces exactly 75%, so the technical definition is the maximum length x such that using contigs of length at least x accounts for at least 75% of the total assembly length.",
"NG75" : "is the contig length such that using longer or equal length contigs produces 75% of the bases of the reference genome. This metric is computed only if a reference genome is provided.",
"L50" : "is the minimum number of contigs that produce half (50%) of the bases of the assembly. In other words, it's the number of contigs of length at least N50.",
"LG50" : "is the minimum number of contigs that produce half (50%) of the bases of the reference genome. In other words, it's the number of contigs of length at least NG50. This metric is computed only if a reference genome is provided.",
"L75" : "is the minimum number of contigs that produce 75% of the bases of the assembly. In other words, it's the number of contigs of length at least N75.",
"LG75" : "is the minimum number of contigs that produce 75% of the bases of the reference genome. In other words, it's the number of contigs of length at least NG75. This metric is computed only if a reference genome is provided.",
"NA50" : "is N50 where the lengths of aligned blocks are counted instead of contig lengths. I.e., if a contig has a misassembly with respect to the reference, the contig is broken into smaller pieces. This metric is computed only if a reference genome is provided.",
"NGA50" : "is NG50 where the lengths of aligned blocks are counted instead of contig lengths. I.e., if a contig has a misassembly with respect to the reference, the contig is broken into smaller pieces. This metric is computed only if a reference genome is provided.",
"NA75" : "is N75 where the lengths of aligned blocks are counted instead of contig lengths. I.e., if a contig has a misassembly with respect to the reference, the contig is broken into smaller pieces. This metric is computed only if a reference genome is provided.",
"NGA75" : "is NG75 where the lengths of aligned blocks are counted instead of contig lengths. I.e., if a contig has a misassembly with respect to the reference, the contig is broken into smaller pieces. This metric is computed only if a reference genome is provided.",
"LA50" : "is L50 where aligned blocks are counted instead of contigs. I.e., if a contig has a misassembly with respect to the reference, the contig is broken into smaller pieces.",
"LGA50" : "is LG50 where aligned blocks are counted instead of contigs. I.e., if a contig has a misassembly with respect to the reference, the contig is broken into smaller pieces.",
"LA75" : "is L75 where aligned blocks are counted instead of contigs. I.e., if a contig has a misassembly with respect to the reference, the contig is broken into smaller pieces.",
"LGA75" : "is LG75 where aligned blocks are counted instead of contigs. I.e., if a contig has a misassembly with respect to the reference, the contig is broken into smaller pieces.",
"Average %IDY" : "is the average of alignment identity percent (Nucmer measure of alignment accuracy) among all contigs.",
"# misassemblies" : "is the number of positions in the assembled contigs where the left flanking sequence aligns over 1 kbp away from the right flanking sequence on the reference (*relocation*) or they overlap on more than 1 kbp (*relocation*) or flanking sequences align on different strands (*inversion*) or different chromosomes (*translocation*).",
"# misassembled contigs" : "is the number of contigs that contain misassembly events.",
"Misassembled contigs length" : "is the number of total bases contained in all contigs that have one or more misassemblies.",
"# relocations" : "is the number of relocation events among all misassembly events. Relocation is a misassembly where the left flanking sequence aligns over 1 kbp away from the right flanking sequence on the reference, or they overlap by more than 1 kbp and both flanking sequences align on the same chromosome.",
"# translocations" : "is the number of translocation events among all misassembly events. Translocation is a misassembly where the flanking sequences align on different chromosomes.",
"# interspecies translocations" : "is the number of interspecies translocation events among all misassembly events. Interspecies translocation is a misassembly where the flanking sequences align on different references (based on alignments to the combined reference).",
"# inversions" : "is the number of inversion events among all misassembly events. Inversion is a misassembly where it is not a *relocation* and the flanking sequences align on opposite strands of the same chromosome.",
"# local misassemblies" : "is the number of local misassemblies. We define a local misassembly breakpoint as a breakpoint that satisfies these conditions:

1. Two or more distinct alignments cover the breakpoint.
2. The gap between left and right flanking sequences is less than 1 kbp.
3. The left and right flanking sequences both are on the same strand of the same chromosome of the reference genome.
",
"# scaffold gap size misassemblies" : "is the number of scaffold gap size misassemblies. We define scaffold gap size misassembly as a breakpoint where the flanking sequences combined in scaffold on the wrong distance. These misassemblies are not included in the total number of misassemblies. ",
"# possibly misassembled contigs": "is the number of contigs that contain large unaligned fragment (default min length is 500 bp) and thus could possibly contain interspecies translocation with unknown reference.",
"# possible misassemblies" : "is the number of putative interspecies translocations in possibly misassembled contigs if each large unaligned fragment is supposed to be a fragment of unknown reference.",
"# intergenomic misassemblies" : "is the number of all found and putative (possible) interspecies translocations.",
"# structural variations" : "is the number of misassemblies matched with structural variations.",
"# unaligned mis. contigs" : "is the number of contigs that have the number of unaligned bases more than 50% of contig length and a misassembly event in their aligned fragment. Note that such misassemblies are not counted in *# misassemblies* and other *misassemblies* statistics.",
"# fully unaligned contigs" : "is the number of contigs that have no alignment to the reference sequence.",
"Fully unaligned length" : "is the total number of bases contained in all fully unaligned contigs.",
"# partially unaligned contigs" : "is the number of contigs that have at least one alignment to the reference sequence but also have at least one unaligned fragment of length ≥ *unaligned-part-size threshold*.",
"Partially unaligned length" : "is the total number of unaligned bases in all partially unaligned contigs.",
"# ambiguous contigs" : "is the number of contigs that have reference alignments of equal quality in multiple locations on the reference.",
"Ambiguous contigs length" : "is the total number of bases contained in all ambiguous contigs.",
"Genome fraction (%)" : "is the total number of aligned bases in the reference, divided by the genome size. A base in the reference genome is counted as aligned if there is at least one contig with at least one alignment to this base. Contigs from repeat regions may map to multiple places, and thus may be counted multiple times in this quantity.",
"GC (%)" : "is the total number of G and C nucleotides in the assembly, divided by the total length of the assembly.",
"Reference GC (%)" : "is the total number of G and C nucleotides in the reference, divided by the total length of the reference.",
"# mismatches per 100 kbp" : "is the average number of mismatches per 100000 aligned bases.",
"# mismatches" : "is the number of mismatches in all aligned bases.",
"# indels per 100 kbp" : "is the average number of indels per 100000 aligned bases.",
"# indels" : "is the number of indels in all aligned bases",
"# indels (<= 5 bp)" : "is the number of indels of length less than or equal to 5 bp",
"# indels (> 5 bp)" : "is the number of indels of length greater than 5 bp",
"Indels length" : "is the number of total bases contained in all indels",
"# genes" : "is the number of genes in the assembly (complete and partial), based on a user-provided annotated list of gene positions in the reference genome. A gene counts as 'partially covered' if the assembly contains at least 100 bp of this gene but not the whole gene.",
"# operons" : "is the number of operons in the assembly (complete and partial), based on a user-provided annotated list of operon positions in the reference genome. An operon counts as 'partially covered' if the assembly contains at least 100 bp of this operon but not the whole operon.",
"# predicted genes (unique)" : "is the number of unique genes in the assembly found by a gene prediction tool.",
"# predicted genes (>= 0 bp)" : "is the number of found genes having length greater than or equal to 0 bp.",
"Cumulative length" : "plot shows the growth of assembly contig lengths. On the x-axis, contigs are ordered from largest (contig #1) to smallest. The y-axis gives the size of the x largest contigs in the assembly.",
"Nx" : "plot shows the Nx metric value as x varies from 0 to 100. Nx is the minimum contig length **y** such that using contigs of length at least **y** accounts for at least x% of the total assembly length.",
"NGx" : "plot shows the NGx metric value as x varies from 0 to 100. NGx is the minimum contig length **y** such that using contigs of length at least **y** accounts for at least x% of the bases of the reference genome. This metric is computed only if a reference genome is provided.",
"NAx" : "plot shows the NAx metric value as x varies from 0 to 100. NAx is computed similarly to Nx, but based on lengths of aligned blocks instead of contig lengths. Contigs are broken into aligned blocks at misassembly breakpoints. NAx is the minimum block length **y** such that using blocks of length at least **y** accounts for at least x% of the bases of the assembly. This metric is computed only if a reference genome is provided.",
"NGAx" : "plot shows the NGAx metric value as x varies from 0 to 100.NGAx is computed similarly to NGx, but based on lengths of aligned blocks instead of contig lengths. Contigs are broken at misassembly breakpoints. NGAx is the minimum block length **y** such that using blocks of length at least **y** accounts for at least x% of the bases of the reference genome. This metric is computed only if a reference genome is provided.",
"GC content" : "plot shows the distribution of GC percentage among the contigs, i.e., the total number of bases in contigs with such GC content. Typically, the distribution is approximately Gaussian. However, for some genomes it is not Gaussian. For assembly projects with contaminants, the GC distribution of the contaminants often differs from the reference genome and may give a superposition of multiple curves with different peaks.",
"Duplication ratio" : "is the total number of aligned bases in the assembly (i.e. *Total length* - *Fully unaligned length* - *Partially unaligned length*), divided by the total number of aligned bases in the reference (see the **Genome fraction (%)** metric). If the assembly contains many contigs that cover the same regions of the reference, its *Duplication ratio* may be much larger than 1. This may occur due to overestimating repeat multiplicities and due to small overlaps between contigs, among other reasons.",
"Largest alignment" : "is the length of the largest continuous alignment in the assembly. This metric is always equal to the *Largest contig* metric but it can be smaller if the largest contig of the assembly contains a misassembly event.",
"Total aligned length" : "is the total number of aligned bases in the assembly.",
"Avg contig read support" : "is the average coverage of contigs that have large unique alignments to the reference. Read coverage is extracted from contig names (SPAdes/Velvet naming scheme only).",
"# N's" : "is the total number of uncalled bases (N's) in the assembly.",
"# N's per 100 kbp" : "is the average number of uncalled bases (N's) per 100000 assembly bases.",
"# similar correct contigs" : "is the number of correct contigs similar among > 50% assemblies (see Icarus for visualization).",
"# similar misassembled blocks" : "is the number of misassembled blocks similar among > 50% assemblies (see Icarus for visualization)."
}
