# Supplementary material for: HiMMe: using genetic patterns as a proxy for genome assembly reliability assessment
Source: BMC Genomics. 2017 Sep 5;18:694. doi: 10.1186/s12864-017-3965-2 (PMC5584555; doi:10.1186/s12864-017-3965-2)
Supplement: Supplementary file 3 — ’Supplementary_file3.zip’, Title: ’Data from GAGE’. Description: contains all real data as well as the pertinent results. (ZIP 18,698 kb) [file 12864_2017_3965_MOESM3_ESM.zip › Real data/QUAST/exact/Summary.pdf]

Reference Genome: Staphylococcus Aureus

Assembly	ABySS	Allpaths-LG	Bambus2	MSR-CA	SGA	SOAPdenovo	Velvet
# contigs	5065	51	105	98	6854	182	301
# contigs (>= 1000 bp)	216	43	96	81	764	50	113
# contigs (>= 5000 bp)	173	30	69	68	147	25	81
# contigs (>= 10000 bp)	112	25	54	58	20	20	60
# contigs (>= 25000 bp)	39	22	34	45	0	17	40
# contigs (>= 50000 bp)	6	9	11	22	0	11	17
Total length (>= 0 bp)	3489706	1674547	2277291	2862552	3448095	2631096	2860307
Total length (>= 1000 bp)	3270669	1672792	2271770	2854786	2583186	2594877	2825981
Total length (>= 5000 bp)	3146022	1644977	2207780	2822185	1097906	2522338	2748669
Total length (>= 10000 bp)	2726110	1604982	2103817	2751233	264524	2491844	2603183
Total length (>= 25000 bp)	1587998	1555732	1742250	2512641	0	2439197	2273326
Total length (>= 50000 bp)	511730	1073911	898529	1681932	0	2214210	1447618
Largest contig	125049	234488	147858	139438	16870	518710	169214
Total length	3489706	1674547	2277291	2862552	3448095	2631096	2860307
Reference length	2709797	2709797	2709797	2709797	2709797	2709797	2709797
GC (%)	32.92	32.41	32.55	32.7	31.99	32.46	32.56
Reference GC (%)	32.89	32.89	32.89	32.89	32.89	32.89	32.89
N50	23082	96740	40128	59152	3144	239815	52792
NG50	27917	35088	35491	60489	4236	239815	54353
N75	10535	42963	26883	33499	991	110622	29485
NG75	18805	NA	13763	40318	2307	93229	31403
L50	46	6	17	18	304	4	17
LG50	31	16	22	17	204	4	16
L75	102	13	33	33	767	8	36
LG75	60	NA	49	30	419	9	32
# misassemblies	35	29	32	56	30	57	35
# misassembled contigs	26	13	21	32	30	16	26
Misassembled contigs length	859906	860548	964322	1649852	109966	2209393	1366916
Unaligned length	889,410	288,643	453,863	487,853	1,081,690	496,667	513,759
Aligned length (%)	0.75	0.83	0.80	0.83	0.69	0.81	0.82
Genome fraction (%)	80.298	50.978	67.127	86.865	84.368	77.903	86.246
Duplication ratio	1.195	1.003	1.002	1.009	1.035	1.011	1.004
# mismatches per 100 kbp	1530.4	1520.62	1582.3	1548.98	1427.69	1553.05	1556.89
# indels per 100 kbp	47.89	54.51	63.66	53.15	45.27	52.68	50.92
Largest alignment	82859	135180	95219	135411	16862	198691	134335
Total aligned length	2594392	1384512	1819548	2368119	2354073	2132887	2341411
NA50	13306	41188	31251	31537	1988	44541	31537
NGA50	20628	3926	19589	32179	3099	44140	32222
NGA75	9921	13100	5290	14864	1184	5243	12946
LA50	66	11	25	28	400	14	26
LGA50	42	40	34	26	250	15	24
LGA75	91	NA	68	54	596	47	55
